# Enantioselective titanium-catalyzed cycloadditions of thiophene-*S*,*S*-dioxides with indenes

**DOI:** 10.1039/d6sc01387b

**Published:** 2026-06-23

**Authors:** Peilin Tian, Viktor S. Camara, Margarita Valentine, Andrew P. Tinkler, Agamemnon Crumpton, Edward A. Anderson

**Affiliations:** a Chemistry Research Laboratory, Department of Chemistry, University of Oxford 12 Mansfield Road Oxford OX1 3TA UK edward.anderson@chem.ox.ac.uk; b Department of Physical Chemistry, Institute of Chemistry of São Carlos, University of São Paulo CEP 13560-970 São Carlos Brazil

## Abstract

Asymmetric inverse electron-demand Diels–Alder (IEDDA) cycloadditions of thiophene *S*,*S*-dioxides (TDOs) remain largely unexplored. Here we report the development of highly enantioselective titanium-catalyzed IEDDA reactions between TDOs and indenes which enables the asymmetric construction of complex carbocyclic frameworks. In combination with TiCl_2_(O^*i*^Pr)_2_, two chiral ligand classes proved capable of promoting this transformation, with BINOL-based catalysts offering optimal selectivity. The transformation exhibits broad substrate scope and high functional group tolerance, and is amenable to gram-scale synthesis; the chiral ligand can be readily recovered and recycled with no deterioration in yield or enantioselectivity. The resulting adducts are versatile synthetic intermediates, undergoing facile derivatization *via* reduction, esterification, Suzuki coupling, and further Diels–Alder transformations.

## Abstract 2

Highly enantioselective titanium-catalyzed inverse electron demand Diels–Alder reactions between thiophene *S*,*S*-dioxides and indenes to form enantioenriched carbocyclic frameworks are described.

## Graphical abstract

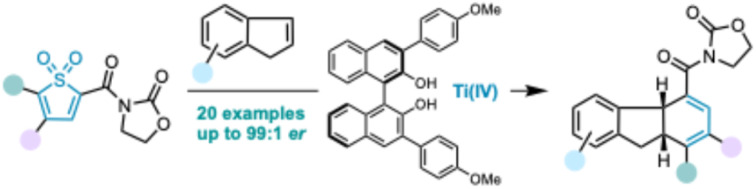

## Introduction

**Fig. 1:**
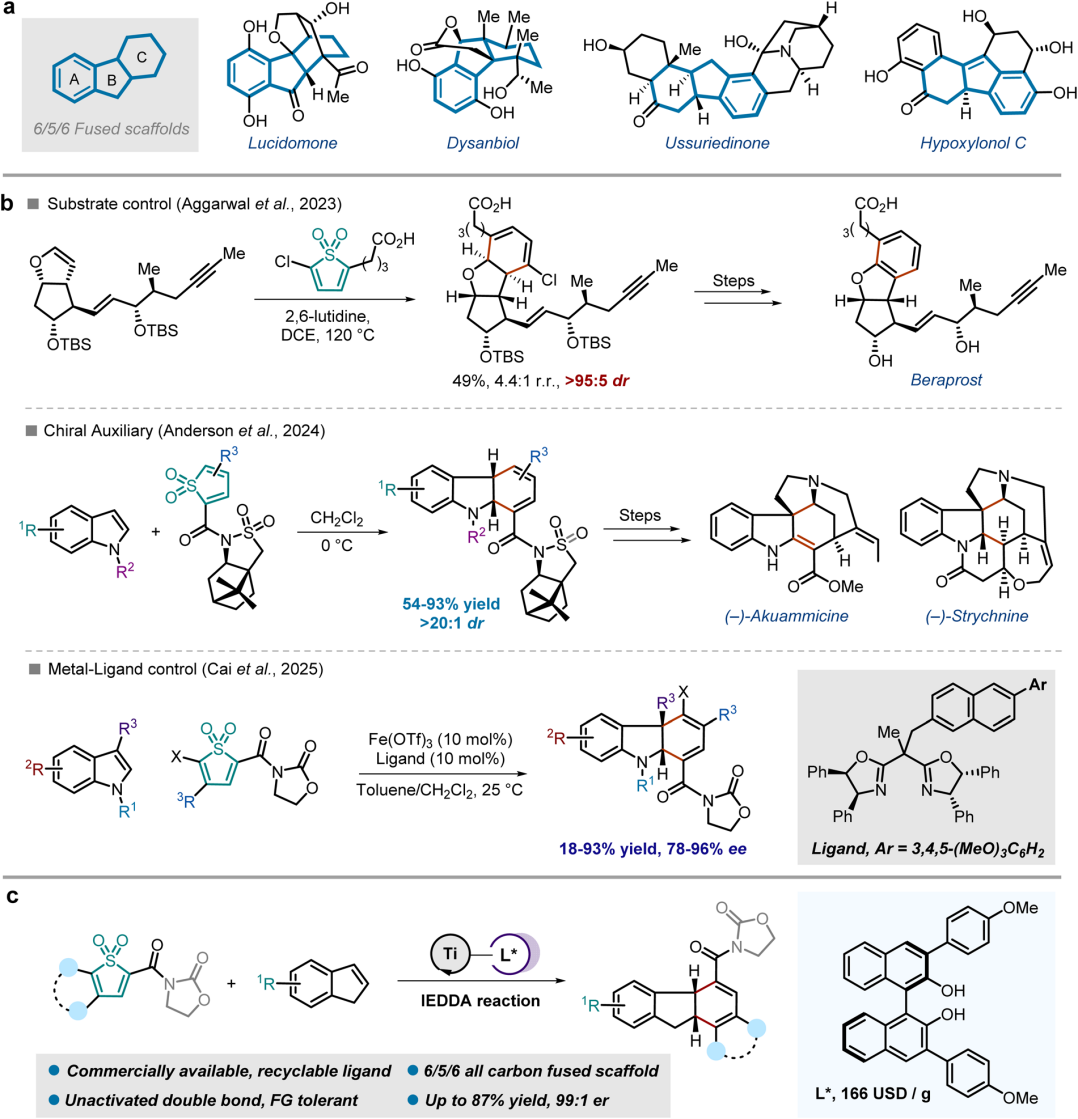
(a) Natural products containing a 6/5/6 all carbon fused scaffolds; (b) examples of asymmetric IEDDA/cheletropic extrusion reactions using TDOs as the diene. (c) Enantioselective titanium/BINOL-catalyzed IEDDA using TDOs and indenes (this work).

Polycyclic molecules are ubiquitous throughout organic chemistry, especially in the context of the complex three-dimensional scaffolds found in bioactive natural products.^1–4^ For example, 6/5/6 all-carbon fused ring systems comprising one benzene ring and two saturated rings are a common structural motif that features in the cores of lucidomone, dysanbiol, ussuriedinone and hypoxylonol C (Fig. 1a).^5–9^ The stereocontrolled construction of such tricyclic frameworks poses a significant synthetic challenge that could be met by the implementation of stereoselective Diels–Alder reactions, due to the ability of such processes to effect the atom-economical formation of six-membered rings with control over multiple stereocenters.^10–12^

Despite the long history of Diels–Alder chemistry, the development of asymmetric inverse-electron demand Diels–Alder (IEDDA) reactions remains less established compared to normal electron-demand variants, with α-pyrones being the most explored substrate class to date.^13–23^ In contrast, thiophene *S*,*S*-dioxides (TDO) have received considerably less attention, despite their established utility as electron-deficient dienes for non-asymmetric IEDDA processes, in which TDOs display high reactivity due to their non-aromatic nature and intrinsically s-*cis* constrained diene. Upon (4+2) cycloaddition, TDOs afford sulfone-bridged adducts that readily undergo cheletropic extrusion of SO_2_,^24,25^ providing an entropic driving force that typically renders the cycloaddition irreversible. Accordingly, TDOs have been applied in cycloadditions with partners such as alkynes, alkenes and furans,^26–32^ and in natural product synthesis contexts for the construction of the aromatic cores of the dictyodendrins^33^ and the illudalane sesquiterpenes.^34^

Despite their promise as dienes for IEDDA chemistry, the use of TDOs in asymmetric transformations remains challenging, with few examples achieving high levels of stereocontrol in the generation of new stereogenic centers. In 2023, Aggarwal *et al.* described the IEDDA reaction of a TDO with a chiral bicyclic dihydrofuran (Fig. 1b), which afforded the corresponding chlorocyclohexadiene (bearing two new stereogenic centers) with excellent diastereoselectivity (>95 : 5 dr), although ultimately these stereocenters were ablated through aromatization.^35^ This transformation nonetheless served as the key step in a synthesis of the antiplatelet pharmaceutical beraprost, with the diastereoselectivity of the cycloaddition being governed by the *cis*-fused-nature of the bicyclic enol ether. More recently, our group reported the development of intermolecular asymmetric (4+2) cycloadditions between indoles and enantioenriched thiophene *S*,*S*-dioxides bearing an inexpensive, readily available camphorsultam sidechain, which produced tricyclic indolines as single diastereomers in high yield.^36^ This functional group tolerant methodology was applied to a wide range of substituted indoles, and was furthermore employed in the total synthesis of eight *Strychnos* alkaloids.

Both of these transformations relied on substrate stereoinduction, as catalytic asymmetric IEDDA/cheletropic extrusions of TDOs had yet to be developed. During the preparation of this manuscript, Cai *et al.* presented a catalytic asymmetric IEDDA between TDOs and indoles using a chiral Fe(iii)/bis-oxazoline catalyst. This elegant reaction could be employed across a range of indoles bearing electron-rich and electron-poor substituents at various positions of the indole ring. However, the methodology was limited to *N*-alkyl indoles, and also required substitution at the indole C3 position; otherwise, a competing Michael addition of the indole to the TDO was observed.^37^ Here, we report a complementary asymmetric IEDDA/cheletropic extrusion of TDOs and indenes, where incorporation of an *N*-acyloxazolidinone moiety onto the TDO scaffold enabled coordination to a chiral titanium Lewis acid–ligand complex that is readily prepared from a common titanium salt and commercially available ligands (Fig. 1c).

## Results and discussion

Our investigations began with the reaction between thiophene *S*,*S*-dioxide 1a and two equivalents of indene in the presence of TiCl_2_(O^*i*^Pr)_2_ (20 mol%) and TADDOL^38–42^ ligand L1 (22 mol%) in toluene at −20 °C (Table 1, Entry 1). The desired cycloadduct 2a was obtained in 37% yield and 63 : 37 er. Encouraged by this result, we examined the influence of the aryl substituents on the TADDOL ‘arms’ (Entries 2–4).^43,44^ Electronic effects exerted only a minor influence on the enantioselectivity, however the introduction of the bulky 1-naphthyl group in L4 offered a pronounced improvement (Entry 4), delivering the product in 90 : 10 er, albeit in only 35% yield. A solvent screen (Entries 5–8) led to an improved 58% yield in chloroform while maintaining 90 : 10 er. The conformation of the five-membered cyclic acetal moiety in the TADDOL ligand has also been found to impact enantioselectivity,^38^ and we thus investigated the influence of the substituents on this ring (L5–L9, Entries 9–13). When both substituents were isopropyl (L6, Entry 10) or phenyl (L7, Entry 11), the reaction reached its highest enantioselectivity (up to 94 : 6 er), although the isolated yields remained modest. Hypothesizing that the moderate yield results from the relatively slow reaction rate and consequently incomplete conversion, we sought to increase the Lewis acidity of the metal center, and therefore evaluated BINOL^15,17,45^ as an alternative ligand. Replacing TADDOL with BINOL L10 (Entry 14) resulted in a marked enhancement of both yield (76%) and enantioselectivity (97 : 3 er). Several metal triflates including Yb(OTf)_3_,^15^ Mg(OTf)_2_ ^46^ and Eu(OTf)_3_ ^47^ were examined, but all afforded only moderate yields and no enantioselectivity. Given the superior performance of the Ti-based conditions, we returned to this system and screened other BINOL derivatives. Bulky substituents (L11, Entry 18 and L12, Entry 19) proved detrimental to enantioselectivity, whereas electron-withdrawing groups (3,5-CF_3_, L13, Entry 20) maintained high selectivity (97 : 3 er). Remarkably, electron-rich aryl substituents such as 3,5-dimethylphenyl (L14, Entry 21) and 4-methoxyphenyl (L15, Entry 22) delivered 2a in 77% and 85% yield respectively, both with 99 : 1 er. Reducing the loading to 15 mol% had minimal impact on enantioselectivity (98 : 2 er) but slightly reduced the yield (75%, Entry 23). Increasing the temperature to −10 °C restored the yield to 81% while maintaining 98 : 2 er (Entry 24). A further reduction to 10% catalyst loading led to a slight erosion in enantioselectivity and yield (Entry 25, 69%, 96 : 4 er). It is worth noting that molecular sieves^48^ were crucial for maintaining high enantioselectivity; in their absence, the enantiomeric ratio dropped dramatically.

**Table 1:** Optimization of asymmetric IEDDA/cheletropic extrusion of 1a and indene

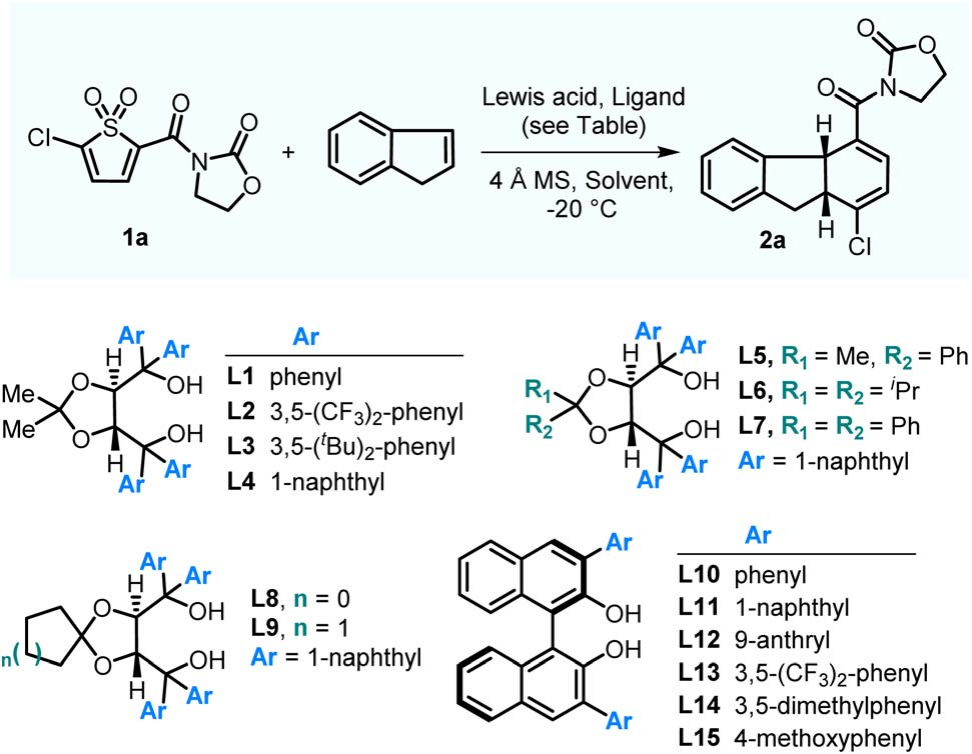					
Entry a	Lewis acid	Ligand	Solvent	Yield (%)	er^b^
1	TiCl_2_(O^*i*^Pr)_2_	L1	Toluene	37	37 63
2	TiCl_2_(O^*i*^Pr)_2_	L2	Toluene	41	20 80
3	TiCl_2_(O^*i*^Pr)_2_	L3	Toluene	27	39 61
4	TiCl_2_(O^*i*^Pr)_2_	L4	Toluene	35	10 90
5	TiCl_2_(O^*i*^Pr)_2_	L4	PhCF_3_	41	16 84
6	TiCl_2_(O^*i*^Pr)_2_	L4	CH_2_Cl_2_	52	18 82
7	TiCl_2_(O^*i*^Pr)_2_	L4	CHCl_3_	58	10 90
8	TiCl_2_(O^*i*^Pr)_2_	L4	Et_2_O	39	16 84
9	TiCl_2_(O^*i*^Pr)_2_	L5	CHCl_3_	51	8 92
10	TiCl_2_(O^*i*^Pr)_2_	L6	CHCl_3_	53	6 94
11	TiCl_2_(O^*i*^Pr)_2_	L7	CHCl_3_	41	6 94
12	TiCl_2_(O^*i*^Pr)_2_	L8	CHCl_3_	37	12 88
13	TiCl_2_(O^*i*^Pr)_2_	L9	CHCl_3_	43	10 90
14	TiCl_2_(O^*i*^Pr)_2_	L10	CHCl_3_	76	97 3
15^c^	Yb(OTf)_3_	L10	CHCl_3_	65	50 50
16^c^	Mg(OTf)_2_	L10	CHCl_3_	72	50 50
17^c^	Eu(OTf)_3_	L10	CHCl_3_	75	50 50
18	TiCl_2_(O^*i*^Pr)_2_	L11	CHCl_3_	67	67 33
19	TiCl_2_(O^*i*^Pr)_2_	L12	CHCl_3_	61	50 50
20	TiCl_2_(O^*i*^Pr)_2_	L13	CHCl_3_	83	97 3
21	TiCl_2_(O^*i*^Pr)_2_	L14	CHCl_3_	77	99 1
22	TiCl_2_(O^*i*^Pr)_2_	L15	CHCl_3_	85	99 1
23^d^	TiCl_2_(O^*i*^Pr)_2_	L15	CHCl_3_	75	98 2
24^d^^,^^e^	TiCl_2_(O^*i*^Pr)_2_	L15	CHCl_3_	81	98 2
25^f^	TiCl_2_(O^*i*^Pr)_2_	L15	CHCl_3_	69	96 4

a Reaction conditions: 1a (0.1 mmol), indene (0.2 mmol), Lewis acid (20 mol%), ligand (22 mol%) in solvent (1.0 mL) at −20 °C.

b er Values were determined by HPLC on a chiral stationary phase.

c The reaction employed 25 mol% of ^i^Pr_2_EtN.

d 15 mol% TiCl_2_(O^*i*^Pr)_2_/16.5 mol% L15.

e The reaction was performed at −10 °C.

f 10 mol% TiCl_2_(O^*i*^Pr)_2_/11 mol% L15.

**Scheme 1:**
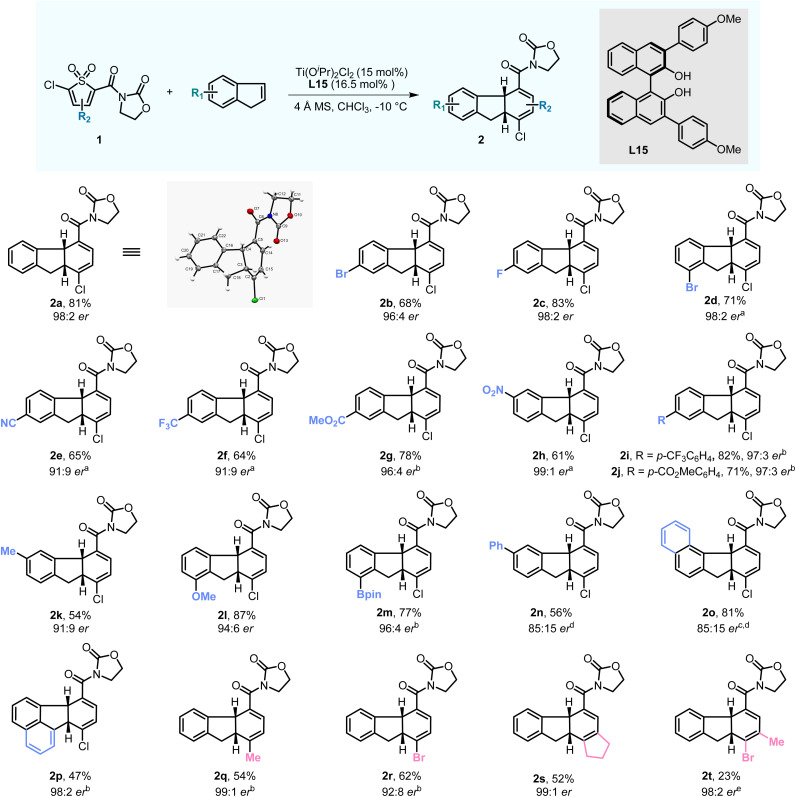
Substrate scope of catalytic asymmetric IEDDA between TDOs and indenes. Reaction conditions: 1a (0.10 mmol), indene (0.20 mmol), TiCl_2_(O^*i*^Pr)_2_ (0.015 mmol), L15 (0.0165 mmol), 4 Å MS (50 mg), dry CHCl_3_ (1 mL, 0.1 M), −10 °C. ^*a*^Reaction performed at 35 °C. ^*b*^Reaction performed at 25 °C. ^*c*^Reaction performed at −20 °C. ^*d*^TiCl_2_(O^*i*^Pr)_2_ (0.025 mmol), L15 (0.0275 mmol) were used. ^*e*^Using L13 as ligand. Er values were determined by HPLC on a chiral stationary phase. The absolute configuration of 2a was determined by X-ray crystallography (CCDC 2512830).

Under the optimized reaction conditions (Table 1, Entry 24), we examined the scope of this transformation (Scheme 1). A series of indene derivatives bearing electron-withdrawing substituents^16^ were well tolerated, affording the desired products in good to excellent yields (2b–2h, 52–83%) with high enantiomeric ratios (91 : 9 to 98 : 2 er), including bromo, fluoro, cyano, trifluoromethyl, ester and nitro groups. Importantly, these substituents were accommodated regardless of their position on the indene ring (C5, C6, or C7). The transformation proved sensitive to temperature: substrates bearing weakly electron-withdrawing groups (Br, F) proceeded efficiently at −10 °C, while more electron deficient indenes (with substituents such as esters, CN, CF_3_ or NO_2_) required elevated temperatures (between room temperature and 35 °C). Electron-donating groups such as methyl and methoxy (2k, 2l) were also tolerated, affording high yields and enantiomeric ratios (54%, 91 : 9 er and 87%, 94 : 6 er respectively); the slightly reduced selectivities compared to the electron-poor substrates could be ascribed to minor levels of non-asymmetric background reaction. A sensitive boronic ester-substituted indene also performed well, affording 2m in 77% yield and 96 : 4 er, enabling further potential derivatization *via* Suzuki cross-coupling. Structurally complex substrates including phenyl-substituted indene, 3*H*-cyclopenta[*a*]naphthalene, and acenaphthylene (2n–2p) were well tolerated, furnishing the corresponding products in moderate to good yields (50–81%) and with good to excellent levels of enantioenrichment (85 : 15 to 98 : 2 er). The successful and selective reaction of acenaphthylene (98 : 2 er) in particular highlights the robustness of the reaction toward sterically and electronically diverse substrates. Finally, the use of other TDOs was examined: methyl substitution on the TDO scaffold afforded 2q in 54% yield and 99 : 1 er, while 5-bromo substitution provided 2r in 62% yield and 92 : 8 er. Pleasingly, a bicyclic TDO substrate reacted smoothly under the optimized conditions, delivering 2s in 52% yield with excellent enantioselectivity (99 : 1 er). A trisubstituted TDO was also tolerated, with 2t isolated with an excellent er of 98 : 2. The absolute configurations of all products were assigned by analogy to the single-crystal X-ray diffraction structure of cycloaddition product 2a (CCDC 2512830).

**Scheme 2:**
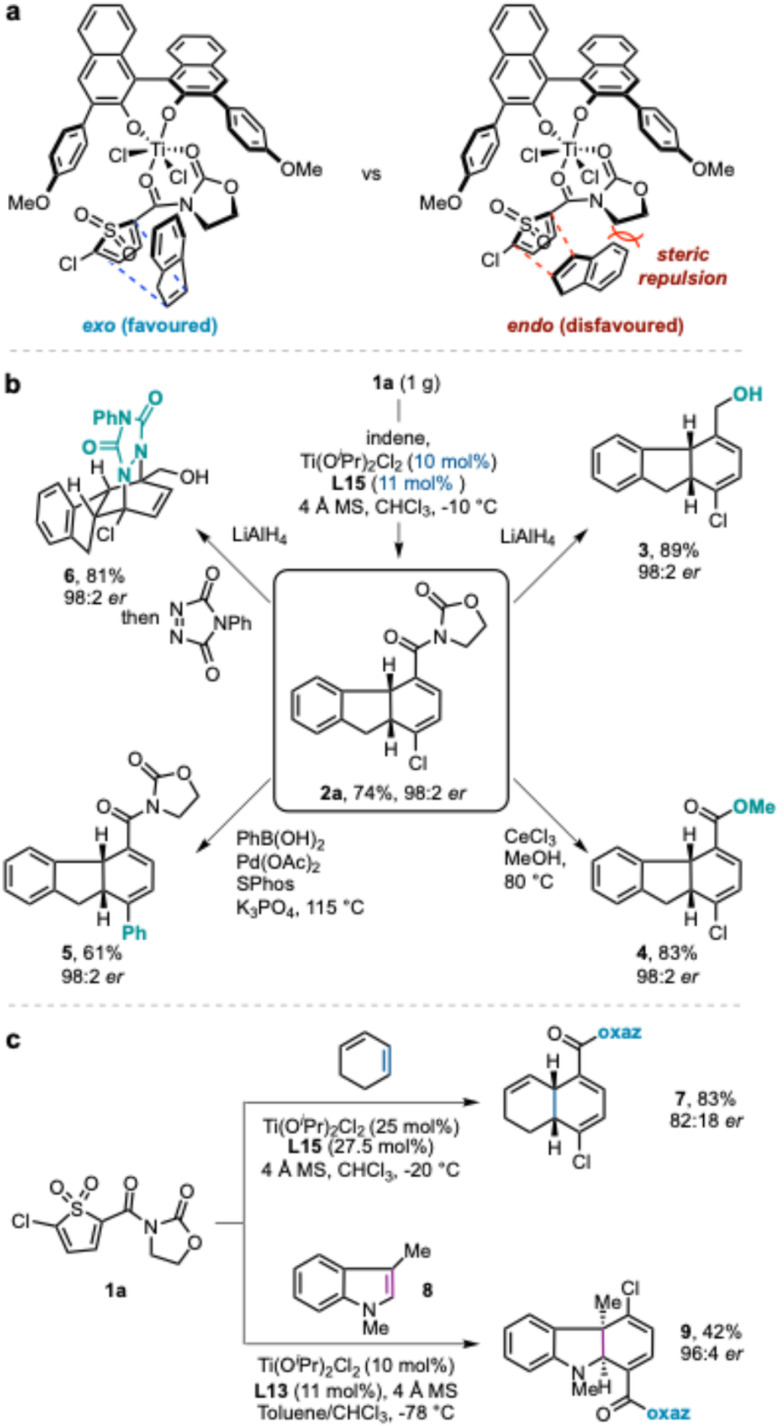
(a) Proposed asymmetric induction model; (b) synthetic transformations; (c) investigation of other dienophile substrates.

To rationalise the stereochemical outcome, we propose the transition state model shown in Scheme 2a, in which enantioselectivity is primarily governed by steric effects. Based on established structures of Ti(iv)(BINOL) complexes,^49,50^ we propose an octahedral geometry where the BINOL and acyloxazolidinone adopt the depicted configuration. The oxazolidone ring is located in a relatively hindered region (proximal to one of the *para*-methoxylphenyl (PMP) BINOL sidechains) and the thiophene *S*,*S*-dioxide rotates to π-stack with the opposing PMP group. This arrangement blocks the ‘back’ face of the TDO (as drawn), such that the indene dienophile approaches from the front face in either an *exo* or *endo* fashion. The conformation of the oxazolidinone is such that it also protrudes towards the front face, which sterically disfavours the *endo* orientation of the indene. This interaction is absent in the *exo* orientation, which is the one that leads to the experimentally observed product.

To demonstrate the practicality of the methodology, a gram-scale synthesis of 2a was carried out (Scheme 2b), which afforded the cycloadduct in 74% yield with 98 : 2 er. On this larger scale, the reaction concentration could be doubled, and the catalyst loading reduced to 10 mol% without compromising the enantioselectivity. Furthermore, 88% of the ligand could be recovered by column chromatography, and the recycled ligand was successfully reused to catalyze the reaction without any loss in enantioselectivity.^51^ With quantities of 2a in hand, further transformations were explored: for example, 2a could be readily reduced to the allylic alcohol 3 in 89% yield, or the oxazolidinone motif could be converted into the corresponding methyl ester 4 in 83% yield under Lewis acidic promotion (CeCl_3_, MeOH).^37^ The chlorine substituent present in 2a provides additional opportunities for derivatization; for example, Suzuki–Miyaura coupling^37^ afforded the arylated diene 5 in 61% yield. Additionally, the diene unit in 2a underwent Diels–Alder reaction with PTAD^52^ (4-phenyl-1,2,4-triazoline-3,5-dione) to furnish the polycyclic bridged adduct 6 in 81% yield.

Finally, to further explore the scope of the asymmetric cycloaddition, other dienophiles were investigated (Scheme 2c). Cyclohexadiene proved a viable substrate,^18^ undergoing asymmetric Diels–Alder reaction with TDO 1a to give the *cis*-fused decalin derivative 7 in 83% yield with 82 : 18 er. It is interesting that cyclohexadiene was observed to act exclusively as a dienophile rather than a diene in this process, which may derive from its ability to engage in an ambimodal transition state, as found for our earlier studies with furan dienophiles.^34^

Inspired by our previous results on auxiliary-controlled asymmetric Diels–Alder reactions of TDOs and indoles, we further sought to develop a catalytic asymmetric variant.^36^ However, attempts using unsubstituted indole predominantly afforded a Michael addition product between C3 of the indole and C3 of the TDO, rather than the desired Diels–Alder adduct. Ultimately, *N*-methyl-3-methylindole 8 was employed as the substrate and, after a brief ligand screen, BINOL ligand L13 (substituted with 3,5-bis(trifluoromethyl) groups) was found to afford the highest enantioselectivity, providing indoline product 9 in 42% yield with 96 : 4 er. Notably, substitution at the N1 and C3 positions of indole is essential to suppress the undesired Michael addition pathway, which is consistent with the findings reported by Cai and co-workers.^37^

## Conclusions

In summary, we have developed a highly enantioselective titanium-catalyzed Diels–Alder reaction of thiophene dioxides with indenes employing commercially-available BINOL derivatives as ligands, providing a broad range of polycyclic products in high yields and excellent enantioselectivities. The reaction exhibits broad substrate scope and functional group tolerance, enabling the asymmetric construction of complex carbocyclic frameworks. The practicality of the methodology was demonstrated by gram-scale synthesis and efficient ligand recycling. Furthermore, the resulting products could be readily diversified through reduction, esterification, Suzuki cross coupling, and Diels–Alder chemistry, and the reaction itself could be expanded to encompass cyclohexadiene and indole derivatives as dienophiles. Overall, this work provides a new catalytic platform for asymmetric inverse electron-demand Diels–Alder reactions based on underexplored TDOs, highlighting the untapped potential of this heterocycle in asymmetric synthesis.

## Author contributions

P. T., V. S. and E. A. conceptualised the project. P. T., V. S., M. V. and A. P. carried out the investigation and developed the methodology. A. C. acquired the X-ray crystal structures of 1a and 2a. All authors conducted the formal analysis and curated the data. E. A. and P. T. supervised the project. E. A. and P. T wrote the original draft and edited the manuscript.

## Conflicts of interest

There are no conflicts to declare.

## Supplementary Material

SC-OLF-D6SC01387B-s001

SC-OLF-D6SC01387B-s002

## Data Availability

CCDC 2512830 and 2512831 contain the supplementary crystallographic data for this paper.^53*a*,*b*^ The data supporting this article has been included as part of the supplementary information (SI). Supplementary information: experimental procedures, NMR data and crystallographic data. See DOI: https://doi.org/10.1039/d6sc01387b.
