# Supplementary material for: Enantioselective titanium-catalyzed cycloadditions of thiophene-S,S-dioxides with indenes
Source: Chem Sci. 2026 Jun 23. Online ahead of print. doi: 10.1039/d6sc01387b (PMC13306190; doi:10.1039/d6sc01387b)
Supplement: SC-OLF-D6SC01387B-s001 [file SC-OLF-D6SC01387B-s001.pdf]

Supplementary Information (SI) for Chemical Science.
This journal is © The Royal Society of Chemistry 2026

Supporting Information

Enantioselective Titanium-Catalyzed Cycloadditions of Thiophene *S,S*-Dioxides with Indenes

Peilin Tian,[‡a] Viktor S. Camara,[‡ a,b] Margarita Valentine,[a] Andrew P. Tinkler,[a] Agamemnon Crumpton[a] and Edward A. Anderson*[a]

[a]Chemistry Research Laboratory, Department of Chemistry, University of Oxford, 12 Mansfield Road, Oxford, OX1 3TA, UK.

[b]Department of Physical Chemistry, Institute of Chemistry of São Carlos, University of São Paulo, CEP 13560-970, São Carlos, Brazil

*Corresponding authors. Email: edward.anderson@chem.ox.ac.uk

Table of contents

1. **Materials and Methods**…………………………………...……………..3
2. **Preparation of Indenes and TADDOLs and Failed Examples**……………....5
3. **General Procedure for Synthesis of Thiophene *S,S*-dioxides**…………….9
4. **General Procedure for Asymmetric Synthesis of 2a–2t**…………………....13
5. **Gram Scale Synthesis and transformations**………………………….....23
6. **Reaction with alternative dienophiles** …………………………………....27
7. **X-Ray data for compound 1a and 2a**……………………..…………......29
8. **NMR Spectra**………………………………..………..……………....32
9. **HPLC Traces**……………………………..………………...…...77
10. **References**……..……………………………….………………….…...103

1. Materials and Methods

NMR Spectroscopy. ^{1}H NMR spectra were recorded at 400, 500 MHz on Bruker AVIII HD 400 and AVII 500 spectrometers. ^{13}C NMR spectra were recorded at 101 or 126 MHz on Bruker AVIII HD 400 and AVII 500 spectrometers. Chemical shifts (δ_H and δ_C) are expressed in parts per million (ppm), with the residual protic solvent signal as the internal reference for ^{1}H NMR spectra [$CDCl_3$ (δ_H = 7.26)] and the deuterated solvent signal as the internal reference for ^{13}C NMR spectra [$CDCl_3$ (δ_C = 77.0)]. Coupling constants (*J*) are reported to the nearest 0.1 Hz. Splitting patterns are described using the following abbreviations: br (broad), s (singlet), d (doublet), t (triplet), q (quartet), quin. (quintet), sept. (septet).

Infrared Spectroscopy. Infrared spectra were recorded on a Bruker Tensor 27 Fourier transform spectrometer, as a thin film on a diamond ATR module. Absorption maxima (ν_{max}) are quoted in wavenumbers (cm^{-1}).

Polarimetry. Optical rotations were recorded using Perkin Elmer 241 Polarimeter (using the sodium D line, 589 nm) with a path length of 1 dm. $[\alpha]_D^T$ are reported in units of 10^{-1} deg cm^2 g^{-1} and the concentrations (c) are reported in g/100 mL

Mass Spectrometry. High Resolution Mass spectrometry was carried out using flow injection analysis and was performed on an ACQUITY I-Class PLUS UPLC System (Waters, Milford, MA, USA) coupled to an ACQUITY RDa mass spectrometer (Waters, Milford, MA, USA) equipped with an ESI probe and a TOF mass analyser. The mass reported is that containing the most abundant isotopes, with each value rounded to 4 decimal places and within 5 ppm of the calculated mass.

X-ray Diffraction. Low temperature single crystal X-ray diffraction data for **1a** and **2a** was collected using a (Rigaku) Oxford Diffraction Supernova A diffractometer. See page 28 and 29 for details.

Reagents, solvents and techniques:

Solvents. Dichloromethane, tetrahydrofuran and toluene were dried by passing through an activated alumina column under argon in a solvent dispenser. All other reagents were used as received. Brine refers to a saturated aqueous solution of NaCl.

Reactions. All reactions were carried out under argon or nitrogen unless otherwise stated. Oven-dried glassware was used for reactions requiring anhydrous conditions.

Heating. For reactions that require heating, a DrySyn heating block or sand bath was employed. The temperature was monitored via a temperature probe plugged into the stirrer plate.

Chromatography. Thin-layer chromatography was performed on Merck aluminium-backed DC 60 F254 0.2 mm precoated plates, which were visualised with UV fluorescence and stained with potassium permanganate or vanillin. Flash column chromatography was performed on MN Kieselgel 60M (particle size 40-63 μm), under a positive pressure of nitrogen, with the solvent system used in parentheses.

2. Preparation of Indenes, TADDOLs and Failed Examples.

Table S1. Structure of indenes.

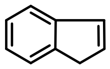

In1

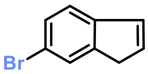

In2

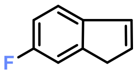

In3

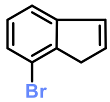

In4

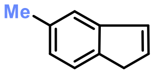

In5

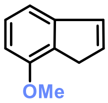

In6

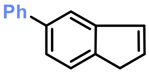

In7

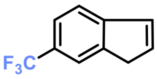

In8

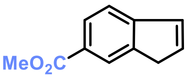

In9

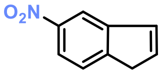

In10

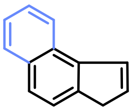

In11

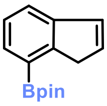

In12

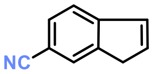

In13

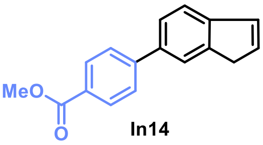

In14

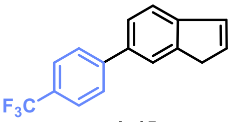

In15

Indenes **In1-In11** were prepared according to the literature[1-3]. Indene **In12-In15** were prepared according to the following procedures.

2-(1*H*-inden-7-yl)-4,4,5,5-tetramethyl-1,3,2-dioxaborolane, In12:

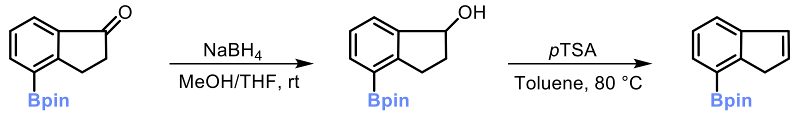

To a stirred solution of indanone **Ind1** (750 mg, 5.00 mmol, 1.00 equiv.) in MeOH (6.0 mL) and THF (3.0 mL) was added $NaBH_4$ (284 mg, 7.50 mmol, 1.50 equiv.) at 0 °C. After stirring at room temperature for 2 h, the reaction was quenched with water (3.0 mL). The resulting mixture was extracted three times with EtOAc. The combined organic layers were dried over Na_2SO_4 and concentrated under reduced pressure. The crude product was redissolved in toluene (22.0 mL) and TsOH·H_2O (*p*TSA, 95.0 mg, 552 μmol, 0.10 equiv.) was added. The reaction mixture was heated at 80 °C for 10 h. After cooling to ambient temperature, K_2CO_3 (10% aq., 15.0 ml) was added and the organic phase was isolated. The organic phase was then washed with brine, dried over Na_2SO_4 and concentrated under reduced pressure. The residue was purified by flash chromatography on silica gel (pentane eluent) to afford **In12** (211 mg, 872 μmol, 30%) as a white solid.

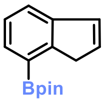

In12

In12: IR (film) ν_{max}: 2979, 1699, 1600, 1419, 1372, 1342, 1220, 1133, 971, 857, 773, 669 cm^{-1}; **^{1}H NMR** (500 MHz, $CDCl_3$) δ 7.64 (dd, *J* = 7.4, 1.2 Hz, 1H), 7.50 (dd, *J* = 7.5, 1.2 Hz, 1H), 7.28 (t, *J* = 7.4 Hz, 1H), 6.88 (dt, *J* = 5.6, 1.9 Hz, 1H), 6.60 (dt, *J* =

5.6, 2.0 Hz, 1H), 3.60 (d, *J* = 2.0 Hz, 2H), 1.36 (s, 12H); **^{13}C NMR** (126 MHz, $CDCl_3$) δ 151.0, 144.1, 134.9, 131.4, 131.2, 125.6, 123.5, 83.5, 40.8, 25.0; **HRMS** calcd. For $C_{15}H_{20}BO_2^+$ [M + H]$^+$ 243.1551, found 243.1544.

1*H*-indene-6-carbonitrile, In13:

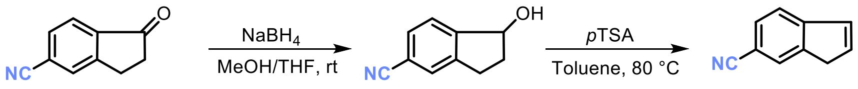

Ind2 **In13**

Following the same procedure for the preparation of compound **In12** from indanone **Ind1**, compound **In13** was obtained from indanone **Ind2** (252 mg, 1.61 mmol, 1.0 equiv.) as a white solid (127 mg, 0.90 mmol, 56%).

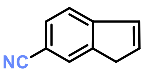

In13

In13: IR (film) ν_{max}: 2223, 1611, 1467, 1422, 1389, 1220, 949, 876, 833, 773, 669 cm^{-1}; **^{1}H NMR** (400 MHz, $CDCl_3$) δ 7.75 – 7.71 (m, 1H), 7.57 (dd, *J* = 7.8, 1.4 Hz, 1H), 7.46 (d, *J* = 7.8 Hz, 1H), 6.95 – 6.89 (m, 1H), 6.81 (dt, *J* = 5.6, 2.0 Hz, 1H), 3.46 (d, *J* = 2.1 Hz, 2H); **^{13}C NMR** (101 MHz, $CDCl_3$) δ 149.2, 144.0, 138.6, 131.7, 130.8, 127.0, 121.5, 119.9, 107.7, 39.1; **HRMS** calcd. For $C_{10}H_7NNa^+$ [M + Na]$^+$ 164.0471, found 164.0470.

Methyl 4-(1*H*-inden-6-yl)benzoate, In14 and 6-(4-(trifluoromethyl)phenyl)-1*H*-indene, In15

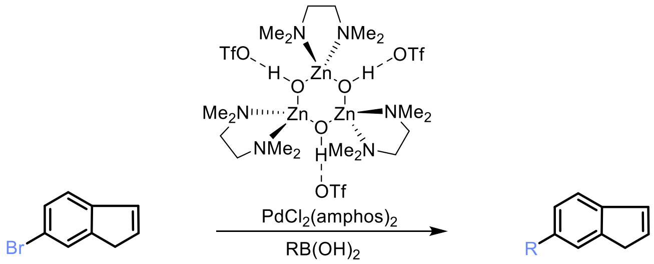

In2

In14: R = *p*-CO$_2$MeC$_6$H$_4$R = *p*-CF$_3$C$_6$H$_4$
In15: R = *p*-CF$_3$C$_6$H$_4$

Indenes **In14** and **In15** were prepared according to the literature.[4] To a stirred solution of **In2** (1.0 equiv.), [(tmeda)Zn(OH)(OTf)]$_3$ (0.78 equiv. per Zn), and $RB(OH)_2$ (1.1 equiv.) in THF (0.2 M) was added $PdCl_2(amphos)_2$ (0.02 equiv.) under Ar. After stirring for 10 h at room temperature, saturated aqueous $NaHCO_3$ was added and the mixture was extracted three times with Et_2O. The organic extracts were dried over Na_2SO_4 and concentrated under reduced pressure. The residue was purified by flash chromatography on silica gel (pentane/Et_2O = 10:1,

v/v) to afford **In14** (151 mg, 0.60 mmol, 66% yield) as a white solid or **In15** (137 mg, 0.53 mmol, 61% yield) as a white solid.

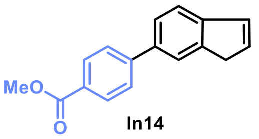

In14

In14: IR (film) ν_{max} (cm^{-1}): 2948, 1720, 1608, 1439, 1287, 774 cm^{-1}; **^{1}H NMR** (500 MHz, $CDCl_3$) δ 8.17 – 8.09 (m, 2H), 7.75 (dt, *J* = 1.8, 0.8 Hz, 1H), 7.73 – 7.68 (m, 2H), 7.56 (dd, *J* = 7.8, 1.7 Hz, 1H), 7.49 (dd, *J* = 7.8, 0.6 Hz, 1H), 6.94 (dtd, *J* = 5.6, 2.0, 0.8 Hz, 1H), 6.64 (dt, *J* = 5.6, 2.0 Hz, 1H), 3.95 (s, 3H), 3.48 (d, *J* = 2.1 Hz, 2H); **^{13}C NMR** (126 MHz, $CDCl_3$) δ 167.0, 146.2, 144.9, 144.4, 136.4, 135.2, 131.7, 130.0, 128.3, 126.9, 125.6, 122.6, 121.2, 52.0, 39.1; **HRMS** Calculated for $C_{17}H_{15}O_2^+$ $[M+H]^+$ 251.1067, found 251.1056.

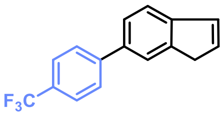

In15

In15: IR (film) ν_{max} (cm^{-1}): 2926, 1692, 1330, 1125, 1074, 1012 cm^{-1}; **^{1}H NMR** (500 MHz, $CDCl_3$) δ 7.77 – 7.68 (m, 5H), 7.57 – 7.50 (m, 2H), 6.96 (dtd, *J* = 4.8, 2.0, 0.7 Hz, 1H), 6.66 (dt, *J* = 5.5, 2.0 Hz, 1H), 3.51 (t, *J* = 2.1 Hz, 2H); **^{13}C NMR** (126 MHz, $CDCl_3$) δ 145.3, 145.0, 144.6, 136.2, 135.3, 131.7, 128.8 (q, J_{C-F} = 32.5 Hz), 127.3, 125.7, 125.6 (q, J_{C-F} = 3.4 Hz), 123.3 (q, J_{C-F} =272.2 Hz), 122.7, 121.3, 39.2; **^{19}F NMR** (377 MHz, $CDCl_3$) δ -62.3. **HRMS** Calculated for $C_{16}H_{11}F_3^+$ $[M+H]^+$ 261.0886, found 261.0882.

Table S2. Structure of TADDOLs

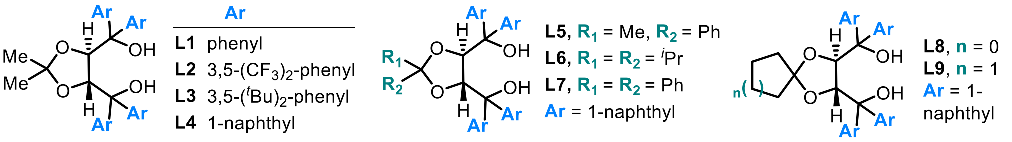

TADDOL ligands **L1** and **L14** were commercially available. **L2-L3**[5], **L5**[6] and **L7-L9**[7-9] were prepared according to the literature. **L6** was prepared according to the following procedure.

((4*R*,5*R*)-2,2-Diisopropyl-1,3-dioxolane-4,5-diyl)bis(di(naphthalen-1-yl)methanol), L6

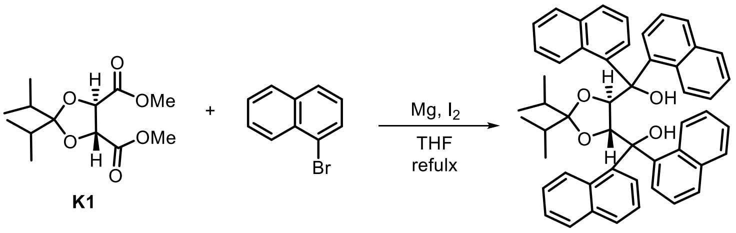

To a stirred suspension of Mg turnings (1.00 g, 41.6 mmol, 5.00 equiv.) and a crystal of I_2 in anhydrous THF (12.0 mL) was added a solution of 1-bromonaphthalene (10.3 g, 49.8 mmol, 6.00 equiv.) in THF (40.0 mL). The mixture was heated at reflux for 2 h and then allowed to cool to room temperature. A solution of ketal **K1**[5] (2.27 g, 8.30 mmol, 1.00 equiv.) in THF (15.0 mL) was added dropwise, and the reaction mixture was heated at reflux for an additional 12 h. After cooling to room temperature, the reaction was quenched by careful addition of saturated aqueous NH_4Cl. The layers were separated, and the aqueous layer was extracted with Et_2O (3 × 50.0 mL). The combined organic extracts were dried over Na_2SO_4, filtered, and concentrated *in vacuo*. The crude product was purified by flash chromatography on silica gel (eluent pentane/Et_2O = 3:1, v/v) to afford **L6** (4.08 g, 5.64 mmol, 68%) as a yellow solid.

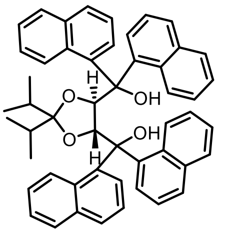

L6

L6: **$[\alpha]_D^{20}$** = −277.9 (c = 0.3, $CHCl_3$); **IR** (film) ν_{max}: 3733, 3710, 3691, 3628, 1868, 1733, 1698, 1663, 1624, 1389, 1218, 775, 669 cm^{-1}; **^{1}H NMR** (500 MHz, DMSO-d_6, 373 K) δ 8.60 – 8.19 (m, 4H), 8.08 (d, J = 7.2 Hz, 2H), 7.95 (d, J = 8.7 Hz, 2H), 7.91 (d, J = 8.2 Hz, 2H), 7.87 (d, J = 8.1 Hz, 2H), 7.79 (d, J = 8.2 Hz, 2H), 7.73 (d, J = 8.2 Hz, 2H), 7.57 (t, J = 7.7 Hz, 4H), 7.22 (t, J = 7.4 Hz, 2H), 7.13 (t, J = 7.2 Hz, 2H), 6.92 (t, J = 7.8 Hz, 2H), 6.75 (s, 2H), 5.23 (s, 2H), 1.23 (dq, J = 14.2, 7.3 Hz, 3H), 0.28 (s, 6H), -0.06 (s, 6H); **^{13}C NMR** (126 MHz, DMSO-d_6, 373 K) δ 142.6, 139.9, 133.9, 133.5, 132.2, 131.2, 128.2, 128.1, 127.7, 127.4, 127.1, 126.3, 125.3, 124.3, 124.2, 123.8, 123.7, 123.4, 123.0, 113.9, 80.7, 78.5, 33.1, 16.9, 14.5; **HRMS** calcd. For $C_{18}H_{18}NO_3^+$ not found.

Table S3. Failed Examples

The following dienophiles and one thiophene *S*,*S*-dioxides were unsuccessful, either undergoing no reaction, or giving complex mixtures / poor selectivity, or conjugate addition products.

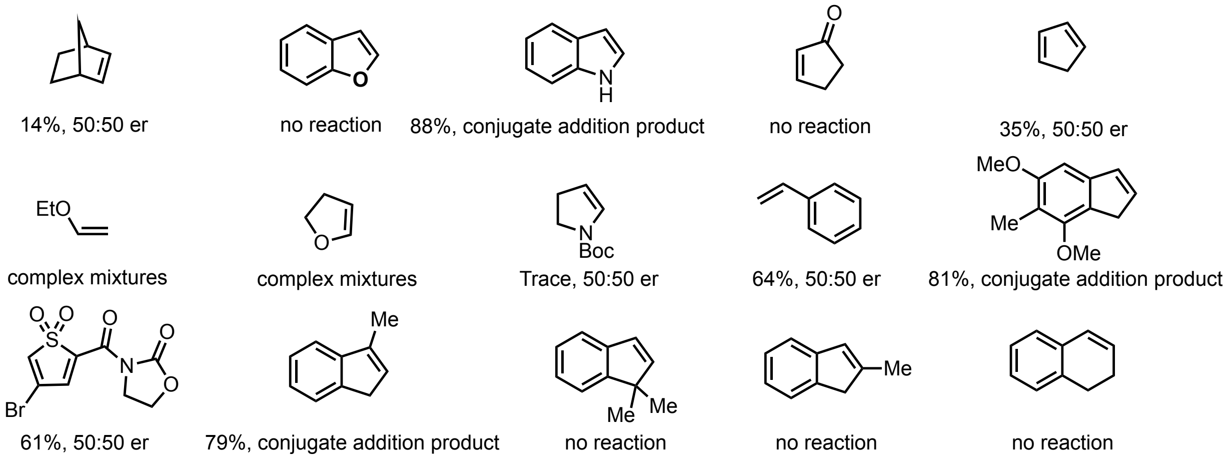

3. General Procedure for Synthesis of Thiophene *S*,*S*-dioxides

Procedure A:

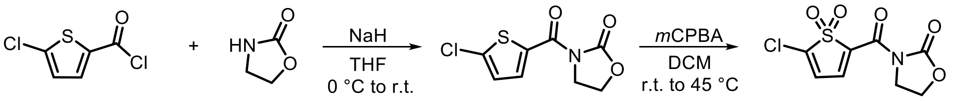

S1 **1a**

Step 1: To a stirred solution of 2-oxazolidone (4.35 g, 50.0 mmol, 1.00 equiv.) in anhydrous THF (200 mL) at 0 °C was added NaH (60% in oil, 3.00 g, 75.0 mmol, 1.50 equiv.). The mixture was stirred at 0 °C for 1 h before 5-chlorothiophene-2-carbonyl chloride (12.1 mL, 100 mmol, 2.00 equiv.) was added. The reaction was then allowed to warm to room temperature and stirred for 14 h, after which it was quenched by the slow addition of water (100 mL). The organic layer was isolated and the aqueous layer was extracted with Et_2O (3 × 100 mL), the combined organic layers were washed with water (150 mL), brine (150 mL), dried over Na_2SO_4, and concentrated *in vacuo*. The residue was purified by column chromatography on silica gel (eluent pentane/Et_2O = 3:1, v/v) to afford **S1** (11.1 g, 48.1 mmol, 96%) as a white solid.

3-(5-Chlorothiophene-2-carbonyl)oxazolidin-2-one, S1:

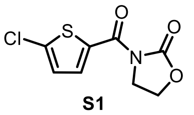

S1

S1: **IR** (film) ν_{max}: 1780, 1652, 1334, 1198, 757 cm^{-1}; **^{1}H NMR** (500 MHz, $CDCl_3$) δ 7.82 (d, *J* = 4.2 Hz, 1H), 6.93 (d, *J* = 4.2 Hz, 1H), 4.48 (t, *J* = 7.8 Hz, 2H), 4.18 – 4.10 (m, 2H); **^{13}C NMR** (126 MHz, $CDCl_3$) δ 160.9, 153.1, 139.5, 135.4, 133.0, 126.9, 62.5, 44.2; **HRMS** calcd. For $C_8H_7ClNO_3S^+$ $[M + H]^+$ 231.9830, found 231.9836.

Step 2: To a stirred solution of thiophene **S1** (2.31 g, 10.0 mmol, 1.00 equiv.) in anhydrous CH_2Cl_2 (50 mL) at room temperature was added *m*-CPBA (≥98% purity, 6.10 g, 35.4 mmol, 3.50 equiv) The reaction mixture was fitted with a reflux condenser and heated to 45 °C for 60 h. After cooling to room temperature, the resulting mixture was filtered to afford a solid, which was washed with Et_2O to give **1a** as a pale yellow powder (973 mg, 3.70 mmol, 37%). The filtrate was concentrated under reduced pressure, and the residue was washed with Et_2O to afford an additional portion of **1a** as a yellow solid (473 mg, 1.80 mmol, 18%).

3-(5-Chloro-1,1-dioxidothiophene-2-carbonyl)oxazolidin-2-one, 1a:

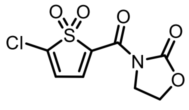

1a

1a: **IR** (film) ν_{max}: 1775, 1655, 1323, 1146, 1101, 760 cm^{-1}; **^{1}H NMR** (500 MHz, $CDCl_3$) δ 8.14 (d, *J* = 5.4 Hz, 1H), 6.80 (d, *J* = 5.4 Hz, 1H), 4.54 (t, *J* = 7.9 Hz, 2H), 4.19 (t, *J* = 7.9 Hz, 2H); **^{13}C NMR** (126 MHz, $CDCl_3$) δ 156.9, 152.4, 139.4, 137.0, 132.1, 120.9, 63.0, 43.7; **HRMS** calcd. For $C_8H_6ClNO_5SNa^+$ $[M + Na]^+$ 285.9547, found 285.9538;

Procedure B:

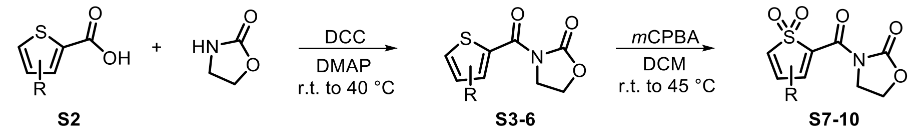

Step 1: Under an N_2 atmosphere, thiophene-2-carboxylic acid **S2** (1.10 equiv.) and DMAP (0.3 equiv.) were dissolved in anhydrous CH_2Cl_2 (0.2 M) at 0 °C. DCC (1.10 equiv.) was then added and the mixture was stirred for 10 minutes before the addition of 2-oxazolidone (1.00 equiv.). The mixture was then stirred overnight at 40 °C. After, the solvent was removed *in vacuo*, the crude reaction mixture was purified by column chromatography on silica gel (CH_2Cl_2: pentane= 5:10, v/v] to afford **S3-6**.

3-(5-Methylthiophene-2-carbonyl)oxazolidin-2-one, S3:

Following procedure B, compound **S3** was obtained from **2-oxazolidone** (435 mg, 5.00 mmol, 1.0 equiv.) as a white solid (721 mg, 3.42 mmol, 68%).

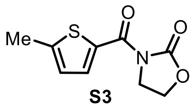

S3

S3: **IR** (film) ν_{max}: 1789, 1646, 1320, 1268, 755 cm^{-1}; **^{1}H NMR** (400 MHz, $CDCl_3$) δ 7.78 (d, *J* = 3.9 Hz, 1H), 6.79 (dd, *J* = 3.9, 1.0 Hz, 1H), 4.47 (t, *J* = 7.9 Hz, 2H), 4.14 (t, *J* = 7.5 Hz, 2H), 2.53 (d, *J* = 1.0 Hz, 3H); **^{13}C NMR** (101 MHz, $CDCl_3$) δ 161.9, 153.3, 149.9, 136.1, 132.2, 126.3, 62.4, 44.3, 15.8; **HRMS** calcd. For $C_9H_{10}NO_3S^+$ $[M + H]^+$ 212.0376, found 212.0382.

3-(5-Bromothiophene-2-carbonyl)oxazolidin-2-one, S4:

Following procedure B, compound **S3** was obtained from **2-oxazolidone** (1.98 g, 22.7 mmol, 1.0 equiv.) as a white solid (4.30 g, 15.6 mmol, 69%).

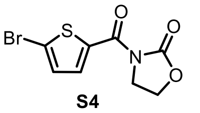

S4

S4: **IR** (film) ν_{max}: 1786, 1651, 1416, 1334, 1199, 967, 740 cm^{-1}; **^{1}H NMR** (400 MHz, $CDCl_3$) δ 7.76 (d, J = 4.2 Hz, 1H), 7.07 (d, *J* = 4.2 Hz, 1H), 4.48 (t, *J* = 7.8 Hz, 2H), 4.14 (t, *J* = 7.8 Hz, 2H); **^{13}C NMR** (101 MHz, $CDCl_3$) δ 160.7, 153.0, 136.0, 130.6, 122.7, 62.5, 44.2; **HRMS** calcd. For $C_8H_7BrNO_3S^+$ $[M + H]^+$ 275.9325, found 275.9316.

3-(5-Bromo-4-methylthiophene-2-carbonyl)oxazolidin-2-one, S5:

Following procedure B, compound **S3** was obtained from **2-oxazolidone** (75.0 mg, 861 μmol, 1.0 equiv.) as a white solid (224 mg, 775 μmol, 90%).

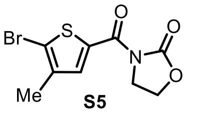

S5

S5: **IR** (film) ν_{max}: 1788, 1656, 1381, 1321, 1207, 1037, 756 cm^{-1}; **^{1}H NMR** (400 MHz, $CDCl_3$) δ 7.68 (s, 1H), 4.48 (t, *J* = 7.8 Hz, 2H), 4.13 (t, *J* = 7.8 Hz, 2H), 2.20 (s, 3H); **^{13}C NMR** (101 MHz, $CDCl_3$) δ 161.0, 153.1, 138.1, 137.1, 133.6, 120.5, 62.5, 44.2, 15.3; **HRMS** calcd. For $C_9H_9BrNO_3S^+$ $[M + H]^+$ 289.9481, found 289.9508.

3-(5,6-Dihydro-4*H*-cyclopenta[*b*]thiophene-2-carbonyl)oxazolidin-2-one, S6:

Following procedure B, compound **S3** was obtained from **2-oxazolidone** (377 mg, 4.33 mmol, 1.0 equiv.) as a white solid (450 mg, 1.90 mmol, 44%).

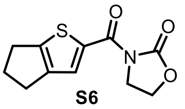

S6

S6: **IR** (film) ν_{max}: 1778, 1649, 1305, 1186, 1039, 759 cm^{-1}; **^{1}H NMR** (500 MHz, $CDCl_3$) δ 7.68 (s, 1H), 4.47 (t, *J* = 7.8 Hz, 2H), 4.14 (t, *J* = 7.8 Hz, 2H), 2.94 (t, *J* = 7.3 Hz, 2H), 2.76 (t, *J* = 7.3 Hz, 2H), 2.45 (p, *J* = 7.3 Hz, 2H); **^{13}C NMR** (126 MHz, $CDCl_3$) δ 162.3, 153.4, 153.3, 147.3, 136.9, 131.0, 62.4, 44.4, 29.6, 29.1, 28.1; **HRMS** calcd. For $C_{11}H_{12}NO_3S^+$ $[M + H]^+$ 238.0532, found 238.0535.

3-(5-Methyl-1,1-dioxidothiophene-2-carbonyl)oxazolidin-2-one, 1b:

Following the same procedure for preparation of compound **1a** from **S1**, compound **1b** was obtained from **S3** (150 mg, 710 μmol, 1.0 equiv.) as a white solid (146 mg, 590 μmol, 83%).

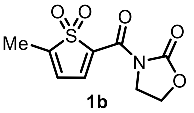

1b

1b: **IR** (film) ν_{max}: 1783, 1659, 1386, 1335, 1309, 1200, 1128, 1040, 741 cm^{-1}; **^{1}H NMR** (400 MHz, $CDCl_3$) δ 7.99 (d, *J* = 4.9 Hz, 1H), 6.52 (dd, *J* = 4.9, 2.1 Hz, 1H), 4.51 (t, *J* = 7.9 Hz, 2H), 4.18 (t, *J* = 7.9 Hz, 2H), 2.24 (d, *J* = 1.9 Hz, 3H); **^{13}C NMR** (101 MHz, $CDCl_3$) δ 157.4, 152.5, 146.6, 138.1, 132.5, 120.6, 62.8, 43.7, 10.1; **HRMS** calcd. For $C_9H_{10}NO_5S^+$ $[M + H]^+$ 244.0274, found 244.0269.

3-(5-Bromo-1,1-dioxidothiophene-2-carbonyl)oxazolidin-2-one, 1c:

Following the same procedure for preparation of compound **1a** from **S1**, compound **1c** was obtained from **S4** (400 mg, 1.45 mmol, 1.0 equiv.) as a off-white solid (240 mg, 779 μmol, 54%).

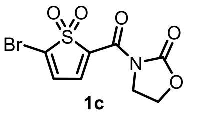

1c

1c: **IR** (film) ν_{max}: 1784, 1389, 1333, 1217, 1153, 913, 749 cm^{-1}; **^{1}H NMR** (400 MHz, $CDCl_3$) δ 8.07 (d, *J* = 5.3 Hz, 1H), 7.01 (d, *J* = 5.3 Hz, 1H), 4.54 (t, *J* = 7.9 Hz, 2H), 4.19 (t, *J* = 7.9 Hz, 2H); **^{13}C NMR** (126 MHz, $CDCl_3$) δ 156.9, 152.4, 137.5, 133.4, 128.3, 125.4, 62.9, 43.7; **HRMS** calcd. For $C_8H_7BrNO_5S^+$ $[M + H]^+$ 307.9223, found 307.9229.

3-(5-Bromo-4-methyl-1,1-dioxidothiophene-2-carbonyl)oxazolidin-2-one, 1d:

Following the same procedure for preparation of compound **1a** from **S1**, compound **1d** was obtained from **S5** (450 mg, 1.55 mmol, 1.0 equiv.) as a white solid (425 mg, 1.32 mmol, 85%).

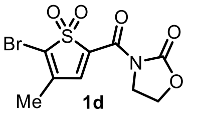

1d

1d: **IR** (film) ν_{max}: 1788, 1729, 1331, 1214, 1155, 746 cm^{-1}; **^{1}H NMR** (500 MHz, $CDCl_3$) δ 7.95 (s, 1H), 4.53 (t, *J* = 7.9 Hz, 2H), 4.18 (t, *J* = 7.9 Hz, 2H), 2.14 (s, 3H); **^{13}C NMR** (126 MHz, $CDCl_3$) δ 156.9, 152.5, 140.7, 135.0, 133.5, 122.9, 62.9, 43.7, 16.3; **HRMS** calcd. For $C_9H_9BrNO_5S^+$ $[M + H]^+$ 321.9379, found 321.9374.

3-(1,1-Dioxido-5,6-dihydro-4*H*-cyclopenta[*b*]thiophene-2-carbonyl)oxazolidin-2-one, S10:

Following the same procedure for preparation of compound **1a** from **S1**, compound **1d** was obtained from **S6** (450 mg, 1.90 mmol, 1.0 equiv.) as a yellow solid (266 mg, 989 μmol, 52%).

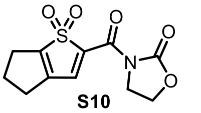

S10

S10: **IR** (film) ν_{max}: 1783, 1651, 1387, 1128, 1038, 741 cm^{-1}; **^{1}H NMR** (500 MHz, $CDCl_3$) δ 7.90 (s, 1H), 4.51 (t, *J* = 7.9 Hz, 2H), 4.17 (t, *J* = 7.9 Hz, 2H), 2.82 (tt, *J* = 7.2, 3.1 Hz, 2H), 2.69 (tt, *J* = 6.4, 3.1 Hz, 2H), 2.45 (p, *J* = 7.5 Hz, 2H); **^{13}C NMR** (126 MHz, $CDCl_3$) δ 157.5, 152.5, 147.1, 146.6, 137.9, 135.2, 62.8, 43.7, 29.2, 26.8, 26.5; **HRMS** calcd. For $C_{11}H_{12}NO_5S^+$ $[M + H]^+$ 270.0431, found 270.0420.

4. General Procedure for Asymmetric Synthesis of 2a–2t

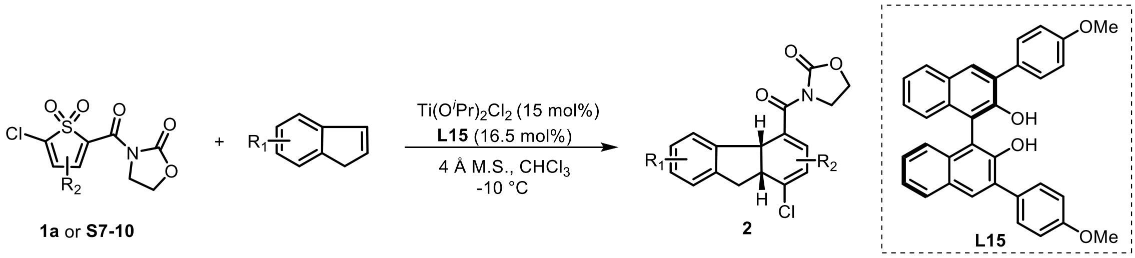

To a stirred solution of $Ti(O^iPr)_2Cl_2$ (3.5 mg, 14.6 μmol) and freshly activated 4 Å molecular sieves (50 mg) in anhydrous $CHCl_3$ (1.0 mL) at room temperature was added **L15** (8.2 mg, 16.1 μmol). The reaction mixture was stirred at room temperature for 2 h, then thiophene *S,S*-dioxide (100 μmol) was added, and the mixture was stirred for an additional 30 min at room temperature. The reaction mixture was then cooled to the specified temperature, followed by the addition of indene (200 μmol). After stirring for 48 h, the crude mixture was directly purified by column chromatography to afford cycloadduct **2**.

3-((4a*R*,9a*R*)-1-Chloro-4a,9a-dihydro-9*H*-fluorene-4-carbonyl)oxazolidin-2-one, 2a:

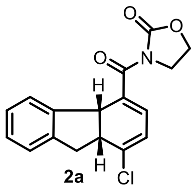

2a

Compound **2a** was prepared following the general procedure at a temperature of –10 °C. **2a**: White foam (25.5 mg, 81.0 μmol, 81%); $[\alpha]_D^{20}$ = −81.3 (c = 1.0, $CHCl_3$); **IR** (film) ν_{max}: 1784, 1671, 1383, 1311, 1097, 759 cm^{-1}; **^{1}H NMR** (400 MHz, $CDCl_3$) δ 7.26 – 7.23 (m, 1H), 7.21 – 7.11 (m, 3H), 6.41 (d, J = 6.4 Hz, 1H), 6.13 (dd, J = 6.4, 1.4 Hz, 1H), 4.54 (d, J = 9.8 Hz, 1H), 4.46 – 4.35 (m, 2H), 4.13 (ddd, J = 10.8, 9.2, 8.5 Hz, 1H), 4.02 – 3.93 (m, 1H), 3.62 – 3.54 (m, 1H), 3.42 (dd, J = 15.5, 5.3 Hz, 1H), 3.35 (dd, J = 16.0, 7.7 Hz, 1H); **^{13}C NMR** (101 MHz, $CDCl_3$) δ 169.6, 153.0, 143.0, 142.5, 141.0, 129.4, 128.8, 127.2, 126.8, 124.2, 124.0, 120.6, 62.2, 46.7, 45.9, 43.3, 38.3; **HRMS** calcd. For $C_{17}H_{15}ClNO_3^+$ $[M + H]^+$ 316.0735, found 316.0741; **HPLC**: CHIRALCEL® OD-H column, *n*-hexane/^{i}PrOH, 85:15 v/v, v = 1.0 mL/min, λ = 280 nm, t_R(major) = 37.3 min, t_R(minor) = 44.6 min, er = 98:2.

3-((4a*R*,9a*R*)-7-Bromo-1-chloro-4a,9a-dihydro-9*H*-fluorene-4-carbonyl)oxazolidin-2-one, 2b:

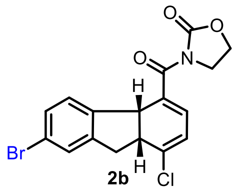

2b

Compound **2b** was prepared following the general procedure at a temperature of –10 °C. **2b**: White foam (26.7 mg, 67.9 μmol, 68% yield); $[\alpha]_D^{25}$ = −57.8 (c = 0.3, $CHCl_3$); **IR** (film) ν_{max} : 2959, 2918, 2852, 1782, 1670, 1309, 1256 cm^{-1}; **^{1}H NMR** (500 MHz, $CDCl_3$) δ 7.43 – 7.35 (m, 1H),

7.31 – 7.24 (m, 1H), 7.07 (dd, *J* = 8.1, 1.1 Hz, 1H), 6.45 (d, *J* = 6.4 Hz, 1H), 6.17 (dd, *J* = 6.4, 1.4 Hz, 1H), 4.51 – 4.40 (m, 3H), 4.15 (dt, *J* = 10.8, 8.8 Hz, 1H), 4.02 (ddd, *J* = 10.8, 8.5, 5.2 Hz, 1H), 3.64 – 3.55 (m, 1H), 3.43 (dd, *J* = 15.8, 5.2 Hz, 1H), 3.34 (dd, *J* = 15.9, 7.6 Hz, 1H); **^{13}C NMR** (126 MHz, $CDCl_3$) δ 169.5, 153.1, 143.5, 142.8, 141.7, 130.1, 130.0, 128.1, 127.2, 125.9, 121.1, 120.8, 62.2, 47.0, 45.5, 43.3, 38.1; **HRMS**: Calculated for $C_{17}H_{13}BrClNO_3Na$ [M + Na]$^+$ 415.9660, found 415.9664; **HPLC**: CHIRALPAK IA column, *n*-hexane/^{i}PrOH, 85:15 v/v, v = 1.0 mL/min, λ = 230 nm, t_R(major) = 24.6 min, t_R(minor) = 18.3 min, er = 96:4.

3-((4a*R*,9a*R*)-1-Chloro-7-fluoro-4a,9a-dihydro-9*H*-fluorene-4-carbonyl)oxazolidin-2-one, 2c:

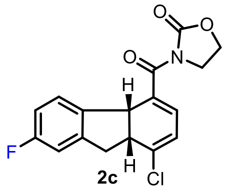

Compound **2c** was prepared following the general procedure at a temperature of –10 °C. **2c**: White foam (27.5 mg, 82.6 μmol, 83% yield); **[α]$_D$^{25}** = −64.1 (*c* = 0.3, $CHCl_3$); **IR** (film) $ν_{max}$: 3016, 2970, 2944, 1784, 1738, 1366, 1216 cm^{-1}; **^{1}H NMR** (500 MHz, $CDCl_3$) δ 7.13 (dd, *J* = 8.4, 5.1 Hz, 1H), 6.96 (dd, *J* = 8.7, 2.5 Hz, 1H), 6.85 (td, *J* = 8.8, 2.5 Hz, 1H), 6.44 (d, *J* = 6.4 Hz, 1H), 6.16 (dd, *J* = 6.3, 1.4 Hz, 1H), 4.51 – 4.48 (m, 1H), 4.48 – 4.40 (m, 2H), 4.15 (dt, *J* = 10.8, 8.9 Hz, 1H), 4.01 (ddd, *J* = 10.9, 8.5, 5.2 Hz, 1H), 3.61 (dddd, *J* = 9.5, 7.1, 5.3, 1.4 Hz, 1H), 3.43 (dd, *J* = 15.9, 5.4 Hz, 1H), 3.34 (dd, *J* = 15.9, 7.6 Hz, 1H); **^{13}C NMR** (126 MHz, $CDCl_3$) δ 169.6, 162.4 (d, $J_{C\text{-}F}$ = 244.6 Hz), 153.1, 143.4 (d, $J_{C\text{-}F}$ = 8.6 Hz), 142.8, 139.0 (d, $J_{C\text{-}F}$ = 2.5 Hz), 129.7, 128.5, 125.4 (d, $J_{C\text{-}F}$ = 8.9 Hz), 120.7, 113.8 (d, $J_{C\text{-}F}$ = 22.4 Hz), 111.2 (d, $J_{C\text{-}F}$ = 22.4 Hz), 62.2, 47.3, 45.2, 43.3, 38.4 (d, $J_{C\text{-}F}$ = 2.2 Hz); **^{19}F NMR** (377 MHz, $CDCl_3$) δ -115.97 (td, *J* = 8.9, 5.2 Hz); **HRMS**: Calculated for $C_{17}H_{13}FClNO_3Na$ [M + Na]$^+$ 356.0460, found 356.0468; **HPLC**: CHIRALPAK IA column, *n*-hexane/^{i}PrOH, 85:15 v/v, v = 1.0 mL/min, λ = 280 nm, t_R(major) = 19.6 min, t_R(minor) = 15.4 min, er = 98:2.

3-((4a*R*,9a*R*)-8-Bromo-1-chloro-4a,9a-dihydro-9*H*-fluorene-4-carbonyl)oxazolidin-2-one, 2d:

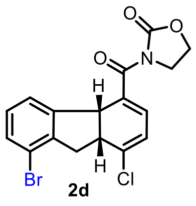

Compound **2d** was prepared following the general procedure at a temperature of 35 °C. **2d**: White foam (27.9 mg, 71.2 μmol, 71%); **[α]$_D$^{20}** = −37.2 (*c* = 1.0, $CHCl_3$); **IR** (film) $ν_{max}$: 1783, 1670, 1567, 1384, 1219, 770 cm^{-1}; **^{1}H NMR** (500 MHz, $CDCl_3$) δ 7.33 (dt, *J* = 7.9, 0.9 Hz, 1H), 7.11 – 7.06 (m, 1H), 7.04 – 6.98 (m, 1H), 6.45 (dt, *J* = 6.5, 0.8 Hz, 1H), 6.15 (dd, *J* = 6.4, 1.5 Hz, 1H), 4.63 (dd, *J* = 10.1, 1.0 Hz, 1H), 4.48 – 4.38 (m, 2H), 4.14 (dt, *J* = 10.9, 8.9 Hz, 1H), 3.98 (ddd, *J* = 10.8, 8.5, 5.1 Hz, 1H),

3.60 (d, *J* = 5.0 Hz, 1H), 3.51 – 3.44 (m, 1H), 3.38 (dd, *J* = 16.4, 7.8 Hz, 1H); **^{13}C NMR** (126 MHz, $CDCl_3$) δ 169.4, 153.0, 144.5, 142.9, 141.6, 130.5, 130.2, 128.7, 128.2, 123.2, 120.8, 119.6, 62.3, 46.8, 45.7, 43.4, 39.8; **HRMS** calcd. For $C_{17}H_{14}ClBrNO_3^+$ $[M + H]^+$ 393.9840, found 393.9842; **HPLC**: CHIRALPAK IA column, *n*-hexane/^{i}PrOH, 70:30 v/v, v = 1.0 mL/min, λ = 254 nm, t_R(major) = 11.0 min, t_R(minor) = 8.7 min, er = 98:2.

(4b*R*,8a*R*)-8-Chloro-5-(2-oxooxazolidine-3-carbonyl)-4b,8a-dihydro-9*H*-fluorene-2-carbonitrile, 2e:

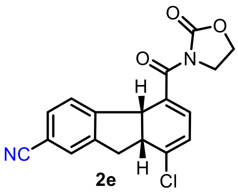

Compound **2e** was prepared following the general procedure at a temperature of 35 °C. **2e**: White foam (22.1 mg, 65.0 µmol, 65%); **$[\alpha]_D^{20}$** = −44.3 (*c* = 0.6, $CHCl_3$); **IR** (film) ν_{max}: 1783, 1671, 1384, 1219, 771 cm^{-1}; **^{1}H NMR** (500 MHz, $CDCl_3$) δ 7.53 (s, 1H), 7.45 (dd, *J* = 8.0, 1.5 Hz, 1H), 7.29 (d, *J* = 7.9 Hz, 1H), 6.50 (d, *J* = 6.5 Hz, 1H), 6.17 (dd, *J* = 6.4, 1.5 Hz, 1H), 4.53 (d, *J* = 9.6 Hz, 1H), 4.51 – 4.40 (m, 2H), 4.14 (dt, *J* = 10.8, 8.7 Hz, 1H), 4.02 (ddd, *J* = 10.9, 8.5, 5.4 Hz, 1H), 3.67 – 3.59 (m, 1H), 3.53 – 3.45 (m, 1H), 3.37 (dd, *J* = 16.0, 7.6 Hz, 1H); **^{13}C NMR** (101 MHz, $CDCl_3$) δ 169.3, 153.2, 148.3, 142.8, 142.3, 131.3, 131.0, 127.5, 127.2, 125.3, 121.2, 119.1, 111.1, 62.3, 46.7, 46.1, 43.4, 37.7; **HRMS** calcd. For $C_{18}H_{14}ClN_2O_3^+$ $[M + H]^+$ 341.0687, found 341.0686; **HPLC**: CHIRALPAK IA column, *n*-hexane/^{i}PrOH, 70:30 v/v, v = 1.0 mL/min, λ = 280 nm, t_R(major) = 20.2 min, t_R(minor) = 14.9 min, er = 91:9.

3-((4a*R*,9a*R*)-1-Chloro-7-(trifluoromethyl)-4a,9a-dihydro-9*H*-fluorene-4-carbonyl) oxazolidin-2-one, 2f:

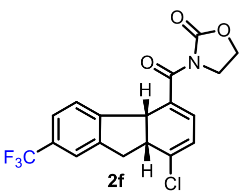

Compound **2f** was prepared following the general procedure at a temperature of 35 °C. **2f**: White foam (24.5 mg, 64.0 µmol, 64%); **$[\alpha]_D^{20}$** = −37.4 (*c* = 0.9, $CHCl_3$); **IR** (film) ν_{max}: 1783, 1699, 1387, 1219, 1120, 771 cm^{-1}; **^{1}H NMR** (500 MHz, $CDCl_3$) δ 7.49 (s, 1H), 7.40 (dd, *J* = 8.0, 1.6 Hz, 1H), 7.28 (d, *J* = 7.9 Hz, 1H), 6.48 (d, *J* = 6.5 Hz, 1H), 6.16 (dd, *J* = 6.4, 1.5 Hz, 1H), 4.58 – 4.52 (m, 1H), 4.49 – 4.39 (m, 2H), 4.14 (dt, *J* = 10.8, 8.8 Hz, 1H), 4.01 (ddd, *J* = 10.8, 8.5, 5.3 Hz, 1H), 3.67 – 3.60 (m, 1H), 3.49 (dd, *J* = 15.9, 4.9 Hz, 1H), 3.38 (dd, *J* = 15.9, 7.6 Hz, 1H); **^{13}C NMR** (126 MHz, $CDCl_3$) δ 169.4, 153.1, 146.8, 142.9, 141.9, 130.6, 129.7 (q, $J_{C\text{-}F}$ = 32.0 Hz), 127.7, 125.3 (q, $J_{C\text{-}F}$ = 272.2 Hz), 124.7, 124.2 (q, $J_{C\text{-}F}$ = 3.9 Hz), 121.0, 120.8 (q, $J_{C\text{-}F}$ = 3.8 Hz), 62.3, 46.9, 45.8, 43.4, 37.9; **^{19}F NMR** (377 MHz, $CDCl_3$) δ -62.1; **HRMS** calcd. For $C_{18}H_{14}ClF_3NO_3^+$ $[M + H]^+$ 384.0609, found 384.0619; **HPLC**: CHIRALPAK IA column, *n*-

hexane/^{i}PrOH, 70:30 v/v, v = 1.0 mL/min, λ = 254 nm, t_R(major) = 10.5 min, t_R(minor) = 8.3 min, er = 91:9.

Methyl (4b*R*,8a*R*)-8-chloro-5-(2-oxooxazolidine-3-carbonyl)-4b,8a-dihydro-9*H*-fluorene-2-carboxylate, 2g:

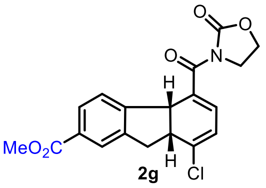

Compound **2g** was prepared following the general procedure at a temperature of 25 °C. **2g**: White foam (29.1 mg, 78.0 µmol, 78%); **[α]$_D$^{20}** = −71.9 (c = 1.0, $CHCl_3$); **IR** (film) ν_{max}: 1783, 1717, 1438, 1386, 1202, 767 cm^{-1}; **^{1}H NMR** (500 MHz, $CDCl_3$) δ 7.91 (d, J = 1.5 Hz, 1H), 7.84 (dd, J = 8.0, 1.6 Hz, 1H), 7.23 (d, J = 8.0 Hz, 1H), 6.45 (d, J = 6.4 Hz, 1H), 6.15 (dd, J = 6.4, 1.4 Hz, 1H), 4.54 (d, J = 9.8 Hz, 1H), 4.49 – 4.38 (m, 2H), 4.14 (dt, J = 10.8, 8.8 Hz, 1H), 4.01 (ddd, J = 10.8, 8.4, 5.3 Hz, 1H), 3.89 (s, 3H), 3.66 – 3.57 (m, 1H), 3.50 – 3.44 (m, 1H), 3.37 (dd, J = 15.8, 7.6 Hz, 1H); **^{13}C NMR** (126 MHz, $CDCl_3$) δ 169.5, 167.1, 153.1, 148.1, 143.0, 141.5, 130.3, 129.4, 128.7, 127.9, 125.1, 124.3, 120.9, 62.3, 52.1, 47.0, 46.0, 43.4, 37.9; **HRMS** calcd. For $C_{19}H_{16}ClNO_5Na^+$ $[M + Na]^+$ 396.0609, found 396.0622; **HPLC**: CHIRALPAK IC column, n-hexane/^{i}PrOH, 60:40 v/v, v = 1.0 mL/min, λ = 280 nm, t_R(major) = 18.9 min, t_R(minor) = 25.2 min, er = 96:4.

3-((4a*R*,9a*R*)-1-Chloro-6-nitro-4a,9a-dihydro-9*H*-fluorene-4-carbonyl)oxazolidin-2-one, 2h:

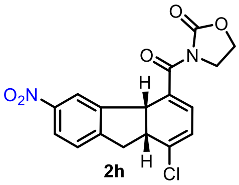

Compound **2h** was prepared following the general procedure at a temperature of 35 °C. **2h**: White foam (22.0 mg, 61.1 µmol, 61%); **[α]$_D$^{20}** = −62.7 (c = 0.3, $CHCl_3$); **IR** (film) ν_{max}: 1791, 1671, 1521, 1219, 771, 669 cm^{-1}; **^{1}H NMR** (500 MHz, $CDCl_3$) δ 8.07 (dd, J = 8.1, 2.2 Hz, 1H), 8.04 (s, 1H), 7.38 (d, J = 8.2 Hz, 1H), 6.51 (d, J = 6.4 Hz, 1H), 6.18 (dd, J = 6.4, 1.4 Hz, 1H), 4.57 (d, J = 9.7 Hz, 1H), 4.50 – 4.44 (m, 2H), 4.20 – 4.11 (m, 1H), 4.05 (ddd, J = 10.8, 7.7, 6.6 Hz, 1H), 3.70 – 3.61 (m, 1H), 3.49 (dd, J = 16.6, 5.3 Hz, 1H), 3.41 (dd, J = 16.6, 7.6 Hz, 1H); **^{13}C NMR** (126 MHz, $CDCl_3$) δ 169.1, 153.2, 148.8, 147.7, 144.6, 142.4, 130.8, 127.2, 124.4, 123.1, 121.1, 120.0, 62.4, 47.0, 45.6, 43.4, 38.2; **HRMS** calcd. For $C_{17}H_{14}ClN_2O_5^+$ $[M + H]^+$ 361.0586, found 361.0589; **HPLC**: CHIRALPAK IA column, n-hexane/^{i}PrOH, 70:30 v/v, v = 1.0 mL/min, λ = 254 nm, t_R(major) = 14.3 min, t_R(minor) = 17.6 min, er = 99:1.

3-((4a*R*,9a*R*)-1-Chloro-7-(4-(trifluoromethyl)phenyl)-4a,9a-dihydro-9*H*-fluorene-4-carbonyl)oxazolidin-2-one, 2i:

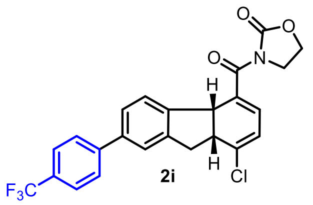

2i

Compound **2i** was prepared following the general procedure at a temperature of 25 °C. **2i**: Yellow oil (37.6 mg, 81.9 μmol, 82%); **[α]$_D$^{20}** = −58.4 (c = 1.0, $CHCl_3$); **IR** (film) ν_{max}: 1784, 1671, 1326, 1219, 1122, 768 cm^{-1}; **^{1}H NMR** (500 MHz, $CDCl_3$) δ 7.70 – 7.62 (m, 4H), 7.47 (d, J = 1.7 Hz, 1H), 7.37 (dd, J = 7.9, 1.8 Hz, 1H), 7.26 (d, J = 7.8 Hz, 1H), 6.47 (d, J = 6.4 Hz, 1H), 6.17 (dd, J = 6.4, 1.4 Hz, 1H), 4.58 (d, J = 9.8 Hz, 1H), 4.49 – 4.39 (m, 2H), 4.17 (dt, J = 10.9, 8.9 Hz, 1H), 4.02 (ddd, J = 10.8, 8.4, 5.2 Hz, 1H), 3.68 – 3.60 (m, 1H), 3.50 (dd, J = 15.8, 4.9 Hz, 1H), 3.41 (dd, J = 15.8, 7.6 Hz, 1H); **^{13}C NMR** (126 MHz, $CDCl_3$) δ δ 169.6, 153.1, 144.7, 143.1, 142.9, 142.1, 139.0, 130.1, 129.2 (q, $J_{C\text{-}F}$ = 32.4 Hz), 128.4, 127.4, 126.2, 125.6 (q, $J_{C\text{-}F}$ = 3.8 Hz), 125.4 (q, $J_{C\text{-}F}$ = 273.4 Hz), 124.8, 122.9, 120.8, 62.3, 47.0, 45.7, 43.4, 38.3; **^{19}F NMR** (471 MHz, $CDCl_3$) δ -62.3; **HRMS** calcd. For $C_{24}H_{18}ClF_3NO_3^+$ $[M + H]^+$ 460.0922, found 460.0917; **HPLC**: CHIRALPAK IA column, n-hexane/^{i}PrOH, 80:20 v/v, v = 1.0 mL/min, λ = 280 nm, t_R(major) = 37.1 min, t_R(minor) = 27.4 min, er = 97:3.

Methyl 4-((4b*R*,8a*R*)-8-chloro-5-(2-oxooxazolidine-3-carbonyl)-4b,8a-dihydro-9*H*-fluoren-2-yl)benzoate, 2j:

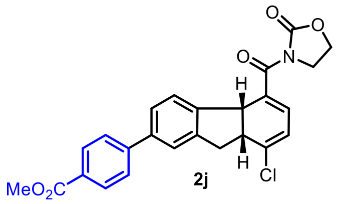

2j

Compound **2j** was prepared following the general procedure at a temperature of 25 °C. **2j**: White foam (31.8 mg, 70.8 μmol, 71%); **[α]$_D$^{20}** = −75.3 (c = 1.0, $CHCl_3$); **IR** (film) ν_{max}: 1792, 1678, 1474, 1396, 1219, 772 cm^{-1}; **^{1}H NMR** (500 MHz, $CDCl_3$) δ 8.08 (d, J = 8.4 Hz, 2H), 7.62 (d, J = 8.4 Hz, 2H), 7.50 (d, J = 1.7 Hz, 1H), 7.40 (dd, J = 7.9, 1.8 Hz, 1H), 7.24 (s, 1H), 6.47 (d, J = 6.4 Hz, 1H), 6.16 (dd, J = 6.4, 1.4 Hz, 1H), 4.58 (d, J = 9.8 Hz, 1H), 4.50 – 4.39 (m, 2H), 4.17 (dt, J = 10.9, 8.9 Hz, 1H), 4.02 (ddd, J = 10.8, 8.4, 5.2 Hz, 1H), 3.93 (s, 3H), 3.67 – 3.60 (m, 1H), 3.49 (dd, J = 15.7, 5.1 Hz, 1H), 3.41 (dd, J = 15.7, 7.7 Hz, 1H); **^{13}C NMR** (126 MHz, $CDCl_3$) δ 169.6, 167.0, 153.1, 145.6, 143.2, 142.8, 142.0, 139.3, 130.0, 130.0, 128.8, 128.4, 127.0, 126.2, 124.8, 122.9, 120.8, 62.3, 52.1, 47.0, 45.7, 43.4, 38.3; **HRMS** calcd. For $C_{25}H_{21}ClNO_5^+$ $[M + H]^+$ 450.1103, found 450.1118; **HPLC**: CHIRALPAK IA column, n-hexane/^{i}PrOH, 80:20 v/v, v = 1.0 mL/min, λ = 280 nm, t_R(major) = 48.7 min, t_R(minor) = 43.4 min, er = 97:3.

3-((4a*R*,9a*R*)-1-Chloro-6-methyl-4a,9a-dihydro-9*H*-fluorene-4-carbonyl)oxazolidin-2-one, 2k:

Compound **2k** was prepared following the general procedure at a temperature of −10 °C. **2k**: White foam (17.7 mg, 53.8 μmol, 54%); **[α]$_D$^{20}** = −56.3 (c = 0.3, $CHCl_3$); **IR** (film) ν_{max}: 1792,

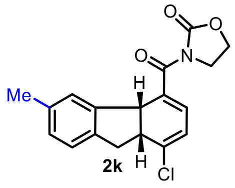

1684, 1559, 1457, 1219, 771 cm^{-1}; **^{1}H NMR** (500 MHz, $CDCl_3$) δ 7.13 (d, *J* = 7.6 Hz, 1H), 7.02 – 6.97 (m, 1H), 6.95 (s, 1H), 6.44 (d, *J* = 6.4 Hz, 1H), 6.13 (dd, *J* = 6.4, 1.5 Hz, 1H), 4.51 – 4.36 (m, 3H), 4.15 (dt, *J* = 10.8, 8.9 Hz, 1H), 4.00 (ddd, *J* = 10.8, 8.4, 5.2 Hz, 1H), 3.58 (tdd, *J* = 9.4, 5.4, 1.5 Hz, 1H), 3.39 (dd, *J* = 15.5, 4.7 Hz, 1H), 3.29 (dd, *J* = 15.5, 7.6 Hz, 1H), 2.29 (s, 3H); **^{13}C NMR** (126 MHz, $CDCl_3$) δ 169.7, 153.1, 143.4, 142.7, 138.1, 136.5, 129.7, 129.0, 128.0, 124.8, 123.6, 120.8, 62.2, 47.1, 45.9, 43.4, 37.8, 21.3; **HRMS** calcd. For $C_{18}H_{17}ClNO_3^+$ $[M + H]^+$ 330.0891, found 330.0885; **HPLC**: CHIRALPAK IA column, *n*-hexane/^{i}PrOH, 90:10 v/v, v = 1.0 mL/min, λ = 280 nm, t_R(major) = 20.2 min, t_R(minor) = 17.5 min, er = 91:9.

3-((4a*R*,9a*R*)-1-Chloro-8-methoxy-4a,9a-dihydro-9*H*-fluorene-4-carbonyl)oxazolidin-2-one, 2l:

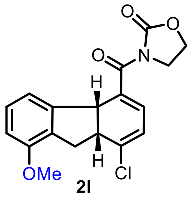

Compound **2l** was prepared following the general procedure at a temperature of –10 °C. **2l**: White foam (30.1 mg, 87.2 μmol, 87% yield); **[α]$_D$^{25}** = −55.2 (*c* = 0.4, $CHCl_3$); **IR** (film) ν_{max}: 3004, 2926, 2851, 1785, 1738, 1365, 1205 cm^{-1}; **^{1}H NMR** (500 MHz, $CDCl_3$) δ 7.12 (t, *J* = 7.9 Hz, 1H), 6.73 (d, *J* = 7.6 Hz, 1H), 6.70 (d, *J* = 8.1 Hz, 1H), 6.40 (d, *J* = 6.4 Hz, 1H), 6.12 (dd, *J* = 6.4, 1.5 Hz, 1H), 4.57 (d, *J* = 10.1 Hz, 1H), 4.47 – 4.37 (m, 2H), 4.15 (dt, *J* = 10.8, 9.0 Hz, 1H), 3.98 (ddd, *J* = 10.8, 8.4, 4.9 Hz, 1H), 3.82 (s, 3H), 3.64 – 3.54 (m, 1H), 3.38 (dd, *J* = 16.1, 5.0 Hz, 1H), 3.31 (dd, *J* = 16.1, 7.9 Hz, 1H); **^{13}C NMR** (126 MHz, $CDCl_3$) δ 169.7, 155.7, 153.1, 144.6, 143.3, 129.5, 128.9, 128.9, 128.5, 120.4, 116.4, 109.0, 62.3, 55.3, 46.5, 46.3, 43.4, 35.4; **HRMS**: Calculated for $C_{18}H_{16}ClNO_4Na$ $[M + Na]^+$ 368.0660, found 368.0667; **HPLC**: CHIRALPAK IA column, *n*-hexane/^{i}PrOH, 85:15 v/v, v = 1.0 mL/min, λ = 280 nm, t_R(major) = 20.9 min, t_R(minor) = 16.8 min, er = 94:6.

3-((4a*R*,9a*R*)-1-Chloro-8-(4,4,5,5-tetramethyl-1,3,2-dioxaborolan-2-yl)-4a,9a-dihydro-9*H*-fluorene-4-carbonyl)oxazolidin-2-one, 2m:

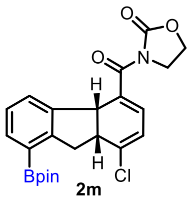

Compound **2m** was prepared following the general procedure at a temperature of 25 °C. **2m**: White foam (34.0 mg, 77.1 μmol, 77% yield) **[α]$_D$^{25}** = −12.9 (*c* = 0.2, $CHCl_3$) **IR** (film) ν_{max}: 2979, 2924, 1785, 1671, 1356, 1312 cm^{-1}; **^{1}H NMR** (400 MHz, $CDCl_3$) δ 7.68 – 7.60 (m, 1H), 7.30 – 7.23 (m, 1H), 7.16 (t, *J* = 7.5 Hz, 1H), 6.45 (dd, *J* = 6.5, 1.0 Hz, 1H), 6.14 (dd, *J* = 6.4, 1.0 Hz, 1H), 4.59 – 4.52 (m, 1H), 4.50 – 4.37 (m, 2H), 4.24 – 4.10 (m, 1H), 4.01 (ddd, *J* = 10.8, 8.3, 5.3 Hz, 1H), 3.74 – 3.64 (m, 1H),

3.61 – 3.49 (m, 2H), 1.36 (s, 12H). **^{13}C NMR** (101 MHz, $CDCl_3$) δ 169.7, 153.1, 148.2, 143.9, 142.1, 134.3, 129.9, 128.9, 127.0, 126.2, 120.4, 83.6, 62.3, 46.6, 45.7, 43.4, 39.3, 25.0, 24.9. **HRMS**: Calculated for $C_{23}H_{25}BClNO_5Na$ $[M + Na]^+$ 464.1407, found 464.1425.); **HPLC**: CHIRALPAK IA column, *n*-hexane/^{i}PrOH, 85:15 v/v, v = 1.0 mL/min, λ = 280 nm, t_R(major) = 11.5 min, t_R(minor) = 9.4 min, er = 96:4.

3-((4a*R*,9a*R*)-1-Chloro-6-phenyl-4a,9a-dihydro-9*H*-fluorene-4-carbonyl)oxazolidin-2-one, 2n:

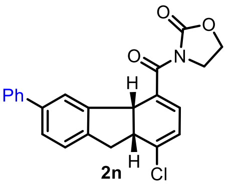

Compound **2n** was prepared following the general procedure at a temperature of –10 °C. **2n**: Yellow foam (21.9 mg, 56.0 μmol, 56%); **$[\alpha]_D^{20}$** = −53.3 (*c* = 0.3, $CHCl_3$); **IR** (film) ν_{max}: 1792, 1698, 1541, 1219, 770 cm^{-1}; **^{1}H NMR** (400 MHz, $CDCl_3$) δ 7.56 – 7.51 (m, 2H), 7.45 – 7.36 (m, 4H), 7.35 – 7.29 (m, 2H), 6.45 (d, *J* = 6.4 Hz, 1H), 6.16 (dd, *J* = 6.4, 1.4 Hz, 1H), 4.58 (d, *J* = 9.7 Hz, 1H), 4.46 – 4.34 (m, 2H), 4.12 (ddd, *J* = 10.8, 9.2, 8.4 Hz, 1H), 3.97 (ddd, *J* = 10.8, 8.2, 5.4 Hz, 1H), 3.68 – 3.57 (m, 1H), 3.50 – 3.42 (m, 1H), 3.38 (dd, *J* = 15.8, 7.6 Hz, 1H); **^{13}C NMR** (101 MHz, $CDCl_3$) δ 169.6, 153.1, 143.3, 143.1, 141.3, 140.4, 140.2, 129.8, 128.7, 128.7, 127.1, 127.0, 126.4, 124.2, 123.2, 120.8, 62.2, 47.1, 46.0, 43.4, 38.1; **HRMS** calcd. For $C_{23}H_{19}ClNO_3^+$ $[M + H]^+$ 392.1048, found 392.1065; **HPLC**: CHIRALPAK IB column, *n*-hexane/^{i}PrOH, 90:10 v/v, v = 1.0 mL/min, λ = 254 nm, t_R(major) = 37.9 min, t_R(minor) = 34.5 min, er = 85:15.

3-((7a*R*,11a*R*)-8-Chloro-7a,11a-dihydro-7*H*-benzo[*c*]fluorene-11-carbonyl)oxazolidin-2-one, 2o:

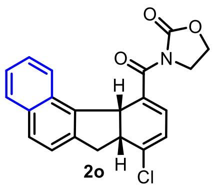

Compound **2o** was prepared following the general procedure at a temperature of –20 °C. **2o**: Yellow foam (29.5 mg, 80.8 μmol, 81%); **$[\alpha]_D^{20}$** = −551.7 (*c* = 1.0, $CHCl_3$); **IR** (film) ν_{max}: 1791, 1683, 1508, 1361, 1220, 770 cm^{-1}; **^{1}H NMR** (500 MHz, $CDCl_3$) δ 7.86 (dd, *J* = 8.0, 1.3 Hz, 1H), 7.76 (dd, *J* = 8.5, 1.2 Hz, 1H), 7.72 (d, *J* = 8.3 Hz, 1H), 7.49 – 7.43 (m, 2H), 7.40 (ddd, *J* = 8.0, 6.7, 1.3 Hz, 1H), 6.16 (d, *J* = 6.2 Hz, 1H), 5.97 (dd, *J* = 6.2, 2.8 Hz, 1H), 5.11 (dd, *J* = 9.3, 2.8 Hz, 1H), 4.02 – 3.91 (m, 1H), 3.53 – 3.38 (m, 4H), 3.34 – 3.24 (m, 2H); **^{13}C NMR** (126 MHz, $CDCl_3$) δ 169.5, 151.9, 141.7, 138.8, 136.6, 132.6, 130.2, 129.0, 128.9, 127.9, 125.9, 124.7, 123.7, 123.1, 123.0, 118.9, 61.8, 47.9, 44.9, 42.4, 40.3; **HRMS** calcd. For $C_{21}H_{17}ClNO_3^+$ $[M + H]^+$ 366.0891, found

366.0895; **HPLC**: CHIRALCEL® OD-H column, *n*-hexane/^{i}PrOH, 85:15 v/v, v = 1.0 mL/min, λ = 280 nm, t_R(major) = 42.6 min, t_R(minor) = 26.8 min, er = 85:15.

3-((6b*R*,10a*S*)-10-Chloro-6b,10a-dihydrofluoranthene-7-carbonyl)oxazolidin-2-one, 2p:

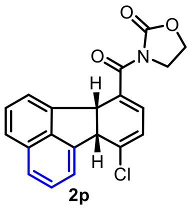

2p

Compound **2p** was prepared following the general procedure at a temperature of 25 °C. **2p**: White foam (16.5 mg, 47.0 μmol, 47% yield); **[α]$_D^{25}$** = +240.0 (*c* = 0.2, $CHCl_3$). **IR** (film) $ν_{max}$: 3001, 2970, 2926, 1782, 1738, 1373, 1217 cm^{-1}; **^{1}H NMR** (400 MHz, $CDCl_3$) δ 7.81 (d, *J* = 7.1 Hz, 1H), 7.72 (d, *J* = 8.2 Hz, 1H), 7.67 (d, *J* = 8.3 Hz, 1H), 7.54 (dd, *J* = 8.2, 7.1 Hz, 1H), 7.45 (dd, *J* = 8.2, 7.0 Hz, 1H), 7.17 – 7.12 (m, 1H), 6.45 (ddd, *J* = 6.6, 1.3, 0.6 Hz, 1H), 6.14 (dd, *J* = 6.6, 1.7 Hz, 1H), 5.33 (dd, *J* = 11.5, 1.3 Hz, 1H), 4.89 (d, *J* = 11.5 Hz, 1H), 4.60 – 4.50 (m, 1H), 4.50 – 4.42 (m, 1H), 4.32 (dt, *J* = 10.8, 9.3 Hz, 1H), 4.07 (ddd, *J* = 10.8, 8.4, 4.1 Hz, 1H). **^{13}C NMR** (101 MHz, $CDCl_3$) δ 169.7, 153.0, 145.0, 143.5, 140.4, 136.8, 131.7, 129.8, 128.9, 128.0, 128.0, 123.9, 123.6, 121.6, 119.7, 119.2, 62.4, 49.9, 45.8, 43.6. **HRMS**: Calculated for $C_{20}H_{14}ClNO_3Na$ $[M + Na]^+$ 374.0554, found 374.0550. **HPLC**: CHIRALPAK IC column, *n*-hexane/^{i}PrOH, 80:20 v/v, v = 1.0 mL/min, λ = 280 nm, t_R(major) = 21.4 min, t_R(minor) = 33.8 min, er = 98:2.

3-((4a*R*,9a*R*)-1-Methyl-4a,9a-dihydro-9*H*-fluorene-4-carbonyl)oxazolidin-2-one, 2q:

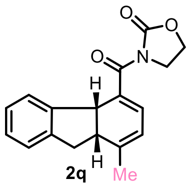

2q

Compound **2q** was prepared following the general procedure at a temperature of 25 °C. **2q**: White foam, (16.0 mg, 54.2 μmol, 54%); **[α]$_D^{25}$** = −56.0 (*c* = 0.4, $CHCl_3$); **IR** (film) $ν_{max}$: 1781, 1669, 1384, 1304, 1197, 1085, 1039, 753 cm^{-1}; **^{1}H NMR** (500 MHz, $CDCl_3$) δ 7.24 – 7.20 (m, 2H), 7.17 – 7.07 (m, 2H), 6.47 (dd, *J* = 6.0, 1.5 Hz, 1H), 5.80 (dd, *J* = 5.9, 1.6 Hz, 1H), 4.45 – 4.41 (m, 1H), 4.40 – 4.33 (m, 2H), 4.14 – 4.06 (m, 1H), 3.93 (ddd, *J* = 10.7, 8.2, 5.1 Hz, 1H), 3.28 – 3.19 (m, 2H), 3.10 (dq, *J* = 9.9, 4.3 Hz, 1H), 1.92 (s, 3H); **^{13}C NMR** (126 MHz, $CDCl_3$) δ 170.3, 153.1, 145.6, 143.8, 142.2, 131.6, 127.0, 126.6, 126.5, 124.8, 123.7, 118.1, 62.1, 45.5, 44.2, 43.4, 38.5, 22.4; **HRMS** calcd. For $C_{18}H_{18}NO_3^+$ $[M + H]^+$ 296.1281, found 296.1283; **HPLC**: CHIRALCEL® OD-H column, *n*-hexane/^{i}PrOH, 90:10 v/v, v = 1.0 mL/min, λ = 280 nm, t_R(major) = 43.2 min, t_R(minor) = 56.9 min, er = 99:1.

3-((4a*R*,9a*R*)-1-Bromo-4a,9a-dihydro-9*H*-fluorene-4-carbonyl)oxazolidin-2-one, 2r:

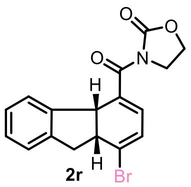

Compound **2r** was prepared following the general procedure at a temperature of 25 °C. **2r**: Off-white foam, (22.1 mg, 61.6 μmol, 62%); **[α]$_D$^{25}** = −67.0 (c = 0.81, $CHCl_3$); **IR** (film) ν_{max}: 1786, 1675, 1382, 1316, 1216, 1042, 914, 755 cm^{-1}; **^{1}H NMR** (500 MHz, $CDCl_3$) δ 7.25 (s, 1H), 7.20 – 7.13 (m, 3H), 6.37 (dd, J = 6.4, 1.3 Hz, 1H), 6.27 (dd, J = 6.4, 1.2 Hz, 1H), 4.51 (d, J = 9.9 Hz, 1H), 4.46 – 4.37 (m, 2H), 4.13 (dt, J = 10.9, 9.0 Hz, 1H), 3.98 (ddd, J = 10.8, 8.4, 5.1 Hz, 1H), 3.69 – 3.63 (m, 1H), 3.43 – 3.32 (m, 2H); **^{13}C NMR** (126 MHz, $CDCl_3$) δ 169.7, 152.9, 142.4, 141.1, 135.1, 129.3, 129.0, 127.2, 126.8, 124.6, 124.4, 124.1, 62.2, 48.6, 46.0, 43.2, 39.7; **HRMS** calcd. For $C_{17}H_{15}BrNO_3^+$ [M + H]$^+$ 360.0230, found 360.0231; CHIRALPAK IA column, n-hexane/^{i}PrOH, 85:15 v/v, v = 1.0 mL/min, λ = 280 nm, t_R(major) = 18.5 min, t_R(minor) = 14.6 min, er = 92:8.

3-((5a*R*,10a*R*)-1,2,3,5a,10,10a-Hexahydrocyclopenta[*a*]fluorene-5-carbonyl)oxazolidin-2-one, 2s:

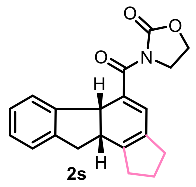

Compound **2s** was prepared following the general procedure at a temperature of −10 °C. **2s**: White foam (16.7 mg, 52.0 μmol, 52%); **[α]$_D$^{20}** = +73.5 (c = 0.5, $CHCl_3$); **IR** (film) ν_{max}: 1773, 1698, 1541, 1387, 1218, 771 cm^{-1}; **^{1}H NMR** (500 MHz, $CDCl_3$) δ 7.23 – 7.18 (m, 1H), 7.18 – 7.09 (m, 3H), 6.69 (s, 1H), 4.50 – 4.39 (m, 2H), 4.37 (d, J = 9.2 Hz, 1H), 4.17 (q, J = 8.7 Hz, 1H), 4.04 (ddd, J = 10.7, 8.3, 5.5 Hz, 1H), 3.54 – 3.45 (m, 1H), 3.27 (dd, J = 15.4, 7.6 Hz, 1H), 3.09 (dd, J = 15.5, 2.8 Hz, 1H), 2.53 – 2.43 (m, 1H), 2.43 – 2.28 (m, 3H), 1.98 – 1.86 (m, 1H), 1.87 – 1.76 (m, 1H); **^{13}C NMR** (126 MHz, $CDCl_3$) δ 170.7, 153.6, 147.4, 144.2, 141.7, 132.9, 132.6, 128.9, 126.7, 126.6, 124.0, 123.7, 62.3, 45.1, 43.8, 42.1, 37.4, 34.1, 33.2, 22.2; **HRMS** calcd. For $C_{20}H_{20}NO_3^+$ [M + H]$^+$ 322.1438, found 322.1456; **HPLC**: CHIRALPAK IC column, n-hexane/^{i}PrOH, 80:20 v/v, v = 1.0 mL/min, λ = 210 nm, t_R(major) = 21.6 min, t_R(minor) = 29.4 min, er = 99:1.

3-((4a*R*,9a*R*)-1-Bromo-2-methyl-4a,9a-dihydro-9*H*-fluorene-4-carbonyl)oxazolidin-2-one, 2t:

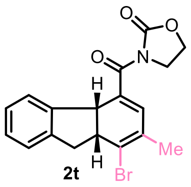

Compound **2c** was prepared following the general procedure at a temperature of 25 °C and **L13** was used instead of **L15**. **2t**: White solid, (8.40 mg, 22.5 μmol, 23%); **[α]$_D$^{25}** = −97.0 (c = 0.22, $CHCl_3$); **IR** (film) ν_{max}: 1783, 1674, 1383, 1312, 1196, 1039, 911, 764 cm^{-1}; **^{1}H NMR** (400 MHz, $CDCl_3$) δ 7.24 (d, J = 6.9 Hz, 1H), 7.21 – 7.08 (m, 3H), 6.24 (d, J = 1.6 Hz, 1H), 4.50 (d, J = 10.0 Hz, 1H), 4.45 – 4.35 (m, 2H), 4.11 (dt, J = 10.8, 9.1 Hz, 1H), 3.94 (ddd, J = 10.8, 8.0, 5.3 Hz, 1H), 3.70 – 3.61 (m, 1H), 3.35 (d, J = 7.4 Hz, 2H), 1.92 (d, J = 1.7 Hz, 3H); **^{13}C NMR** (101 MHz, $CDCl_3$) δ 169.6, 152.8,

142.5, 141.5, 132.5, 130.8, 129.2, 128.3, 127.1, 126.7, 124.5, 124.1, 77.2, 62.2, 49.7, 45.9, 43.1, 40.1, 21.8. **HRMS** calcd. For $C_{18}H_{17}BrNO_3^+$ [M + H]$^+$ 374.0386, found 374.0391; **HPLC**: CHIRALCEL® OD-H column, *n*-hexane/^{i}PrOH, 90:10 v/v, v = 1.0 mL/min, λ = 280 nm, t_R(major) = 35.8 min, t_R(minor) = 42.2 min.

5. Gram Scale Synthesis and transformations.

3-((4a*R*,9a*R*)-1-Chloro-4a,9a-dihydro-9*H*-fluorene-4-carbonyl)oxazolidin-2-one, 2a:

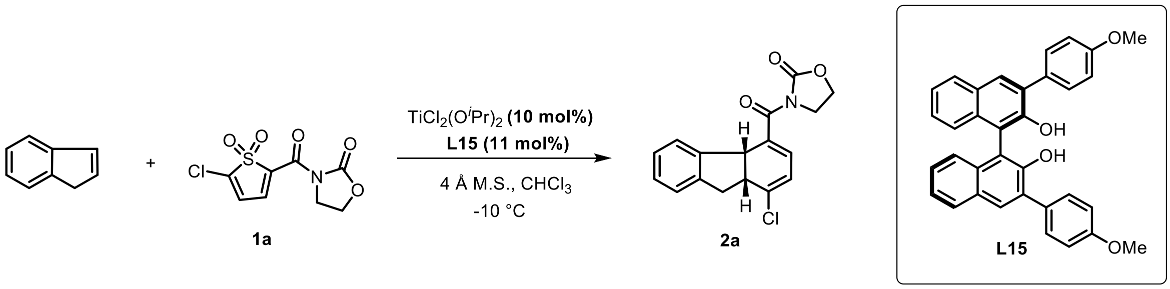

To a stirred solution of Ti(O^{i}Pr)$_2$Cl$_2$ (91.7 mg, 384 µmol) and fresh activated 4 Å molecular sieves (2.0 g) in anhydrous $CHCl_3$ (19.0 mL) at room temperature was added **L15** (212 mg, 426 µmol). After the reaction mixture was stirred at room temperature for 2 h, thiophene *S,S*-dioxide (1.02 g, 3.87 mmol) was added, and the mixture was stirred for an additional 30 min at room temperature. The reaction mixture was then cooled to –10 °C, followed by the addition of indene (0.9 mL, 7.74 mmol). After stirring for 72 h, the crude mixture was purified directly by column chromatography on silica gel (pentane: Et_2O 10:1→1:1, v/v) to afford **2a** (903 mg, 2.86 mmol, 74%) as a white foam and 186 mg (88%, 373 µmol) **L15** was recovered.

((4a*R*,9a*R*)-1-Chloro-4a,9a-dihydro-9*H*-fluoren-4-yl)methanol, 3:

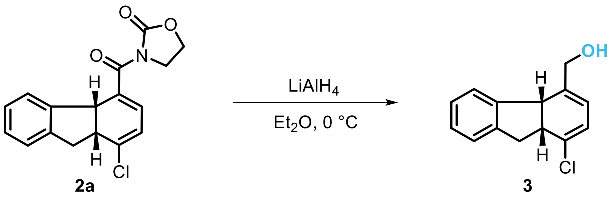

To a stirred solution of **2a** (31.5 mg, 100 µmol, 1.00 equiv.) in anhydrous Et_2O (1.0 mL) at 0 °C was added $LiAlH_4$ (11.4 mg, 300 µmol, 3.00 equiv.). The resultant mixture was warmed to room temperature and stirred for a further 3 hours before cooling to 0 °C and diluting with Et_2O (3.0 mL). The reaction was then quenched with sequential addition of water (0.1 mL), 1 M sodium hydroxide (0.1 mL), and water (0.1 mL), then stirred for 15 min. Anhydrous magnesium sulfate was added and the resultant mixture was stirred for a further 15 min at rt, before the mixture was filtered and concentrated in vacuo. The crude residue was purified by flash chromatography on silica gel (pentane: Et_2O 10:1→3:1, v/v) to afford **3** (20.6 mg, 88.8 µmol, 89%) as a white solid.
3: **$[\alpha]_D^{20}$** = −144.3 (c = 0.3, $CHCl_3$); **IR** (film) ν_{max}: 3345, 1717, 1671, 1558, 1339, 1219, 772 cm^{-1}; **^{1}H NMR** (500 MHz, $CDCl_3$) δ 7.40 (dd, J = 5.3, 3.5 Hz, 1H), 7.29 – 7.24 (m, 1H), 7.20 (dd, J = 5.6, 3.2 Hz, 2H), 6.01 (d, J = 6.2 Hz, 1H), 5.86 (dd, J = 6.2, 1.7 Hz, 1H), 4.29 – 4.12 (m, 3H), 3.45 – 3.28 (m, 2H), 3.17 (dd, J = 14.4, 7.5 Hz, 1H); **^{13}C NMR** (126 MHz, $CDCl_3$) δ 143.1,

141.3, 136.2, 135.0, 127.0, 126.8, 125.1, 124.0, 120.2, 118.5, 77.3, 77.0, 76.7, 64.8, 46.7, 46.6, 39.2; **HRMS** calcd. For $C_{14}H_{13}ClONa^+$ $[M + Na]^+$ 255.0549, found 255.0549; **HPLC**: CHIRALCEL® OD-H column, *n*-hexane/^{i}PrOH, 92:8 v/v, v = 1.0 mL/min, λ = 280 nm, t_R(major) = 12.6 min, t_R(minor) = 17.2 min, er = 98:2.

Methyl (4a*R*,9a*R*)-1-chloro-4a,9a-dihydro-9*H*-fluorene-4-carboxylate, 4:

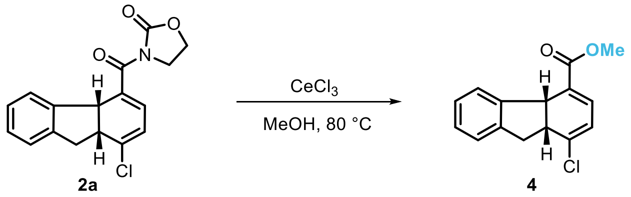

To a stirred solution of **2a** (31.5 mg, 100 μmol, 1.00 equiv.) in anhydrous MeOH (2.0 mL) was added anhydrous $CeCl_3$ (2.5 mg, 10.0 μmol, 0.10 equiv.) at room temperature. The reaction mixture was then heated to 80 °C and stirred for 1 h. After completion, the reaction mixture was concentrated *in vacuo*. The residue was purified by column chromatography on silica gel (pentane: Et_2O 10:1→3:1, v/v) to afford **4** (21.6 mg, 83.1 μmol, 83% yield) as a colorless oil.

4: $[\alpha]_D^{20}$ = +87.9 (*c* = 1.0, $CHCl_3$); **IR** (film) ν_{max}: 1707, 1570, 1436, 1256, 1100, 772 cm^{-1}; **^{1}H NMR** (500 MHz, $CDCl_3$) δ 7.27 – 7.24 (m, 1H), 7.18 (dtt, *J* = 16.8, 7.3, 1.2 Hz, 2H), 7.10 (dt, *J* = 7.9, 1.3 Hz, 1H), 6.98 (dd, *J* = 6.4, 0.8 Hz, 1H), 6.14 (dd, *J* = 6.5, 2.2 Hz, 1H), 4.48 (dd, *J* = 9.5, 1.1 Hz, 1H), 3.88 (s, 3H), 3.63 – 3.52 (m, 2H), 3.35 (dd, *J* = 16.0, 7.6 Hz, 1H); **^{13}C NMR** (126 MHz, $CDCl_3$) δ 167.7, 144.6, 143.0, 140.6, 131.6, 127.2, 127.0, 126.9, 123.8, 123.7, 122.0, 52.0, 46.3, 44.4, 37.7; **HRMS** calcd. For $C_{15}H_{14}ClO_2^+$ $[M + H]^+$ 261.0677, found 261.0669; **HPLC**: CHIRALCEL® OD-H column, *n*-hexane/^{i}PrOH, 95:5 v/v, v = 1.0 mL/min, λ = 280 nm, t_R(major) = 8.2 min, t_R(minor) = 12.2 min, er = 98:2.

Methyl (4a*R*,9a*R*)-1-phenyl-4a,9a-dihydro-9*H*-fluorene-4-carboxylate, 5:

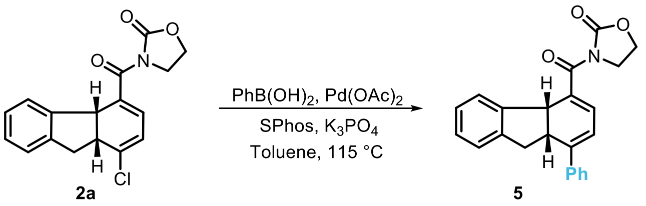

To a stirred solution of **2a** (31.5 mg, 100 μmol, 1.00 equiv.), $Pd(OAc)_2$ (2.3 mg, 10.0 μmol, 0.10 equiv.) and SPhos (10.3 mg, 25.0 μmol, 0.25 equiv.) in anhydrous toluene (1.5 mL) was added phenylboronic acid (18.3 mg, 150 μmol, 1.50 equiv.) and K_3PO_4 (42.5 mg, 200 μmol, 2.00 equiv.) at room temperature. The reaction mixture was then heated to 110 °C. After stirring for

15 h, the reaction mixture was purified directly by column chromatography on silica gel (pentane: Et_2O 20:1→1:1, v/v) to afford **5** (21.7 mg, 60.8 μmol, 61% yield) as a yellow solid.

5: $[\alpha]_D^{20}$ = −457.2 (c = 0.5, $CHCl_3$); **IR** (film) ν_{max}: 2924, 1829, 1784, 1653, 1541, 1220, 1039, 772, 669 cm^{-1}; **^{1}H NMR** (400 MHz, $CDCl_3$) δ 7.58 – 7.51 (m, 2H), 7.44 – 7.27 (m, 5H), 7.21 – 7.10 (m, 3H), 6.60 (dd, J = 6.2, 2.1 Hz, 1H), 6.43 (d, J = 6.2 Hz, 1H), 4.62 (dd, J = 9.8, 2.0 Hz, 1H), 4.42 – 4.34 (m, 2H), 4.11 (dt, J = 10.8, 9.3 Hz, 1H), 3.95 – 3.89 (m, 1H), 3.89 – 3.81 (m, 1H), 3.29 (dd, J = 15.3, 7.5 Hz, 1H), 3.14 (dd, J = 15.3, 9.1 Hz, 1H); **^{13}C NMR** (126 MHz, $CDCl_3$) δ 169.9, 152.9, 145.3, 143.2, 142.9, 139.1, 130.4, 128.7, 128.5, 128.3, 126.7, 126.5, 125.7, 125.1, 123.9, 118.2, 62.1, 44.8, 43.2, 42.8, 40.3; **HRMS** calcd. For $C_{23}H_{20}NO_3^+$ $[M + H]^+$ 358.1438, found 358.1436; **HPLC**: CHIRALCEL® OD-H column, n-hexane/^{i}PrOH, 85:15 v/v, v = 1.0 mL/min, λ = 360 nm, t_R(major) = 34.4 min, t_R(minor) = 31.2 min, er = 98:2.

Methyl (4a*R*,9a*R*)-1-phenyl-4a,9a-dihydro-9*H*-fluorene-4-carboxylate, 6:

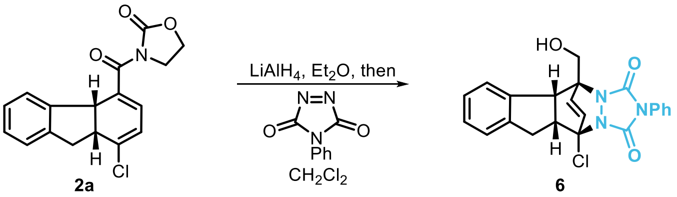

To a stirred solution of **2a** (31.5 mg, 100 μmol, 1.00 equiv.) in anhydrous Et_2O (2.0 mL) was added $LiAlH_4$ (11.4 mg, 300 μmol, 3.00 equiv.) at 0 °C. The reaction mixture was allowed to warm to room temperature and stirred for 1 h before cooling to 0 °C and diluting with Et_2O (5.0 mL). The reaction was quenched with sequential addition of water (0.02 mL), 1 M sodium hydroxide (0.02 mL) and water (0.06 mL), then stirred for 15 min. Anhydrous magnesium sulfate was added and the resultant mixture was stirred for a further 15 min at room temperature, before the mixture was filtered and concentrated *in vacuo*. The crude residue was redissolved in CH_2Cl_2 (2.0 mL) and 4-Phenyl-1,2,4-triazoline-3,5-dione (26.3 mg, 150 μmol, 1.50 equiv.) was added at room temperature. The reaction mixture was stirred at that temperature for 15 min before being concentrated *in vacuo*. The crude residue was purified by column chromatography on (silica gel, pentane: Et_2O 10:1→1:1, v/v) to afford **6** (32.8 mg, 80.6 μmol, 81% yield) as a white solid.

6: $[\alpha]_D^{20}$ = −120.3 (c = 1.0, $CHCl_3$); **IR** (film) ν_{max}: 3734, 3649, 1771, 1699, 1558, 1457, 1219, 1081, 771 cm^{-1}; **^{1}H NMR** (500 MHz, $CDCl_3$) δ 7.51 – 7.43 (m, 5H), 7.42 – 7.37 (m, 1H), 7.26 – 7.15 (m, 3H), 6.51 (dd, J = 8.5, 1.1 Hz, 1H), 6.00 (d, J = 8.5 Hz, 1H), 4.64 (dd, J = 13.7, 5.7 Hz, 1H), 4.38 – 4.20 (m, 2H), 4.13 (dd, J = 10.1, 5.7 Hz, 1H), 3.67 – 3.59 (m, 1H), 3.28 (dd, J = 17.7, 10.4 Hz, 1H), 3.00 (dd, J = 17.7, 5.8 Hz, 1H); **^{13}C NMR** (126 MHz, $CDCl_3$) δ 151.5, 150.8,

142.5, 138.6, 134.8, 131.6, 130.8, 129.1, 128.6, 128.5, 127.0, 126.7, 126.0, 124.8, 80.1, 66.1, 60.3, 51.4, 49.8, 35.4; **HRMS** calcd. For $C_{22}H_{19}ClN_3O_3^+$ $[M + H]^+$ 408.1109, found 408.1099; **HPLC**: CHIRALPAK IB column, *n*-hexane/^{i}PrOH, 60:40 v/v, v = 1.0 mL/min, λ = 254 nm, t_R(major) = 29.4 min, t_R(minor) = 11.1 min, er = 98:2.

6. Reaction with alternative dienophiles.

3-((4a*R*,8a*S*)-4-Chloro-4a,5,6,8a-tetrahydronaphthalene-1-carbonyl)oxazolidin-2-one, 7

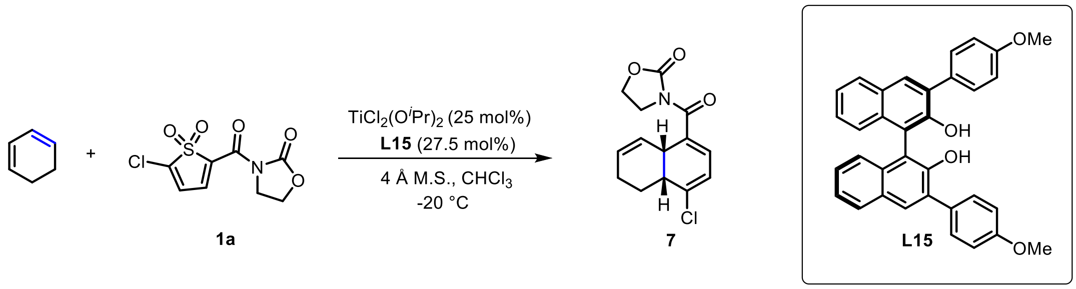

To a stirred solution of $Ti(O^iPr)_2Cl_2$ (6.0 mg, 25.0 μmol, 0.25 equiv.) and fresh activated 4 Å molecular sieves (50.0 mg) in anhydrous $CHCl_3$(1.0 mL) at room temperature was added **L15** (13.7 mg, 27.5 μmol, 0.28 equiv.). After the reaction mixture was stirred at the room temperature for 2 h, thiophene *S,S*-dioxide (26.3 mg, 100 μmol, 1.00 equiv.) **1a** was added, and the mixture was stirred for an additional 30 min at room temperature. The reaction mixture was then cooled to –20 °C, followed by the addition of cyclohexa-1,3-diene (0.02 mL, 200 μmol, 2.00 equiv.). After stirring for 48 h, the crude mixture was purified directly by column chromatography on silica gel (pentane: Et_2O 10:1→2:1, v/v) to afford **7** (23.1 mg, 82.8 μmol, 83%) as a colorless oil.

7: $[\alpha]_D^{20}$ = −10.2 (c = 0.4, $CHCl_3$); **IR** (film) ν_{max}: 1792, 1717, 1558, 1473, 1387, 1219, 771 cm^{-1}; **^{1}H NMR** (500 MHz, $CDCl_3$) δ 6.26 (dd, J = 6.2, 2.0 Hz, 1H), 6.10 (dd, J = 6.1, 0.9 Hz, 1H), 5.82 (ddd, J = 9.8, 3.8, 2.2 Hz, 1H), 5.66 – 5.55 (m, 1H), 4.49 – 4.35 (m, 2H), 4.10 (dt, J = 10.9, 8.9 Hz, 1H), 3.96 (ddd, J = 10.8, 8.5, 5.2 Hz, 1H), 3.75 – 3.63 (m, 1H), 2.78 (td, J = 8.8, 3.1 Hz, 1H), 2.22 – 1.99 (m, 3H), 1.85 – 1.70 (m, 1H); **^{13}C NMR** (126 MHz, $CDCl_3$) δ 169.0, 153.1, 143.2, 131.1, 129.3, 129.0, 123.1, 120.5, 62.2, 43.2, 40.5, 36.2, 23.1, 21.2; **HRMS** calcd. For $C_{14}H_{15}ClNO_3^+$ $[M + H]^+$ 280.0735, found 280.0724; **HPLC**: CHIRALCEL® OD-H column, *n*-hexane/^{i}PrOH, 75:25 v/v, v = 1.0 mL/min, λ = 280 nm, t_R(major) = 17.9 min, t_R(minor) = 21.2 min, er = 82:18.

3-((4a*R*,9a*R*)-4-Chloro-4a,9-dimethyl-4a,9a-dihydro-9*H*-carbazole-1-carbonyl)oxazolidin-2-one, 9

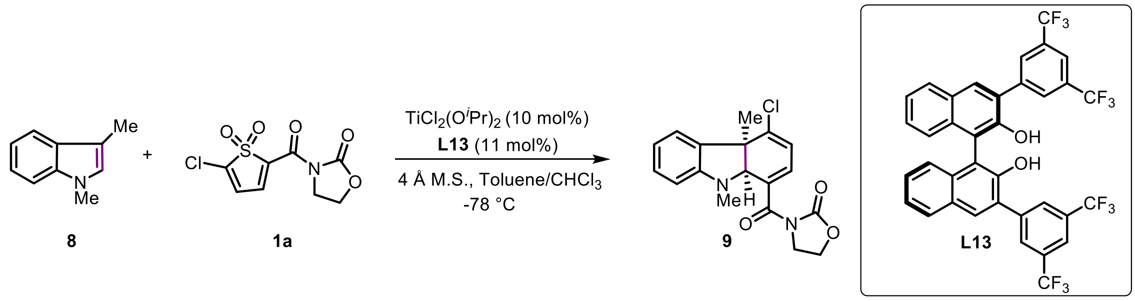

To a stirred solution of Ti(O^{i}Pr)$_2$Cl$_2$ (2.4 mg, 10.0 µmol, 0.10 equiv.) and fresh activated 4 Å molecular sieves (50.0 mg) in anhydrous CHCl$_3$(1.0 mL) at room temperature was added **L13** (5.5 mg, 11.0 µmol, 0.11 equiv.). After the reaction mixture was stirred at room temperature for 2 h, thiophene *S,S*-dioxide (26.3 mg, 100 µmol, 1.00 equiv.) **1a** was added, and the mixture was stirred for an additional 30 min at room temperature. The reaction mixture was then cooled to – 78 °C, followed by the addition of **8** (29.0 mg, 200 µmol, 2.00 equiv.). After stirring for 72 h, the crude mixture was purified directly by column chromatography on silica gel (pentane: Et$_2$O 10:1→1:2, v/v) to afford **9** (14.4 mg, 42.0 µmol, 42%) as a yellow foam.

9: **[α]$_D$^{20}** = +951.2 (*c* = 0.5, CHCl$_3$); **IR** (film) ν_{max}: 2987, 1780, 1670, 1568, 1517, 1308, 1196, 912, 735, 669 cm^{-1}; **^{1}H NMR** (500 MHz, CDCl$_3$) δ 7.36 (d, *J* = 7.4 Hz, 1H), 7.17 (t, *J* = 7.6 Hz, 1H), 6.88 – 6.77 (m, 2H), 6.57 (d, *J* = 7.9 Hz, 1H), 6.29 (d, *J* = 6.5 Hz, 1H), 4.51 (td, *J* = 8.9, 4.1 Hz, 1H), 4.44 (q, *J* = 8.8 Hz, 1H), 4.28 – 4.17 (m, 1H), 4.12 (s, 1H), 4.03 – 3.92 (m, 1H), 2.67 (s, 3H), 1.54 (s, 3H); **^{13}C NMR** (126 MHz, CDCl$_3$) δ 169.8, 153.4, 151.8, 147.3, 135.4, 130.9, 128.3, 126.1, 124.2, 119.9, 118.8, 107.4, 71.7, 62.4, 50.3, 43.8, 34.2, 21.6; **HRMS** calcd. For $C_{18}H_{17}ClN_2O_3Na^+$ [M + Na]$^+$ 367.0820, found 367.0817; **HPLC**: CHIRALPAK IB column, *n*-hexane/^{i}PrOH, 80:20 v/v, v = 1.0 mL/min, λ = 254 nm, t$_R$(major) = 24.0 min, t$_R$(minor) = 22.0 min, er = 96:4.

7. X-Ray data for compound 1a and 2a

Single-crystal X-ray diffraction data were collected on a (Rigaku) Oxford Diffraction SuperNova diffractometer equipped with a Cu Kα microfocus source (λ = 1.5418 Å) and an Atlas CCD area detector. Individual crystals were selected under Paratone-N oil, mounted on MiTeGen loops, and cooled to 150 K using an Oxford Cryosystems nitrogen-flow device.[10]

Data acquisition, indexing, integration, and multi-scan scaling were performed with CrysAlisPro. Unit-cell constants were refined against all suitable reflections during processing.

Structure solution methods were selected according to data quality: compound 1a was solved using SHELXT,[11] while compound 2a was solved using SUPERFLIP.[12] Final refinements for all structures were carried out against F^2 using the CRYSTALS refinement suite.[13,14] Complete crystallographic information, including refinement details and atomic coordinates, is provided in the accompanying CIF files.

Crystallographic data have been deposited with the Cambridge Crystallographic Data Centre under deposition numbers CCDC 2512830 (for compound **2a**) and CCDC 2512831 (for compound **1a**). These files are available free of charge from the CCDC via www.ccdc.cam.ac.uk/data_request/cif.

Figure S1. Solid state structure of **1a**. Displacement ellipsoid plots are drawn at 50% probability.

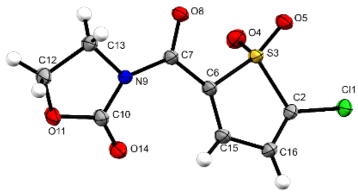

Table S4. Crystal data and structure refinement for 1a.

CCDC code	2512831	
Empirical formula	C8 H6 Cl N O5 S	
Formula weight	263.66	
Temperature	100 K	
Wavelength	1.54184 Å	
Crystal system	Monoclinic	
Space group	P 21/n	
Unit cell dimensions	a = 10.97250(10) Å	a= 90°.
	b = 6.52180(10) Å	b= 95.9999(8)°.
	c = 14.02240(10) Å	g = 90°.
Volume	997.952(19) $Å^3$	
Z	4	
Density (calculated)	1.755 Mg/m^3	
Absorption coefficient	5.462 mm^{-1}	
F(000)	536	
Crystal size	0.272 x 0.120 x 0.087 mm^3	
Theta range for data collection	4.878 to 75.771°.	
Index ranges	-13<=h<=12, -8<=k<=8, -17<=l<=17	
Reflections collected	31025	
Independent reflections	2038 [R(int) = 0.042]	
Completeness to theta = 73.497°	99.9 %	
Absorption correction	Semi-empirical from equivalents	
Max. and min. transmission	0.62 and 0.32	
Refinement method	Full-matrix least-squares on F^2	
Data / restraints / parameters	2038 / 0 / 145	
Goodness-of-fit on F^2	1.0031	
Final R indices [I>2sigma(I)]	R1 = 0.0341, wR2 = 0.0853	
R indices (all data)	R1 = 0.0350, wR2 = 0.0860	
Largest diff. peak and hole	0.69 and -0.65 e.$Å^{-3}$	

Figure S2. Solid state structure of **2a**. Displacement ellipsoid plots are drawn at 50% probability.

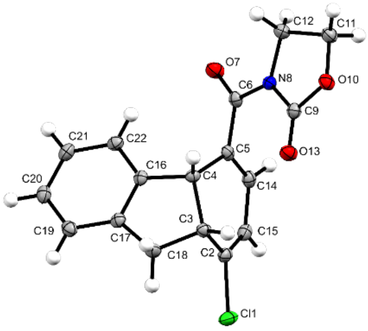

Table S5. Crystal data and structure refinement for 2a.

CCDC code	2512830	
Empirical formula	C17 H14 Cl N O3	
Formula weight	315.76	
Temperature	100 K	
Wavelength	1.54184 Å	
Crystal system	Orthorhombic	
Space group	P 21 21 21	
Unit cell dimensions	a = 7.24530(4) Å	a= 90°.
	b = 11.54662(6) Å	b= 90°.
	c = 17.00452(9) Å	g = 90°.
Volume	1422.576(14) Å^3	
Z	4	
Density (calculated)	1.474 Mg/m^3	
Absorption coefficient	2.492 mm^{-1}	
F(000)	655.997	
Crystal size	0.136 x 0.092 x 0.075 mm^3	
Theta range for data collection	4.629 to 76.011°.	
Index ranges	-9<=h<=9, -14<=k<=14, -21<=l<=20	
Reflections collected	52669	
Independent reflections	2940 [R(int) = 0.039]	
Completeness to theta = 73.731°	99.7 %	
Absorption correction	Semi-empirical from equivalents	
Max. and min. transmission	0.83 and 0.64	
Refinement method	Full-matrix least-squares on F^2	
Data / restraints / parameters	2940 / 85 / 256	
Goodness-of-fit on F^2	1.0089	
Final R indices [I>2sigma(I)]	R1 = 0.0223, wR2 = 0.0567	
R indices (all data)	R1 = 0.0228, wR2 = 0.0571	
Absolute structure parameter	0.002(3)	
Largest diff. peak and hole	0.14 and -0.14 e.Å^{-3}	

8. NMR Spectra

2-(1*H*-Inden-7-yl)-4,4,5,5-tetramethyl-1,3,2-dioxaborolane, In12

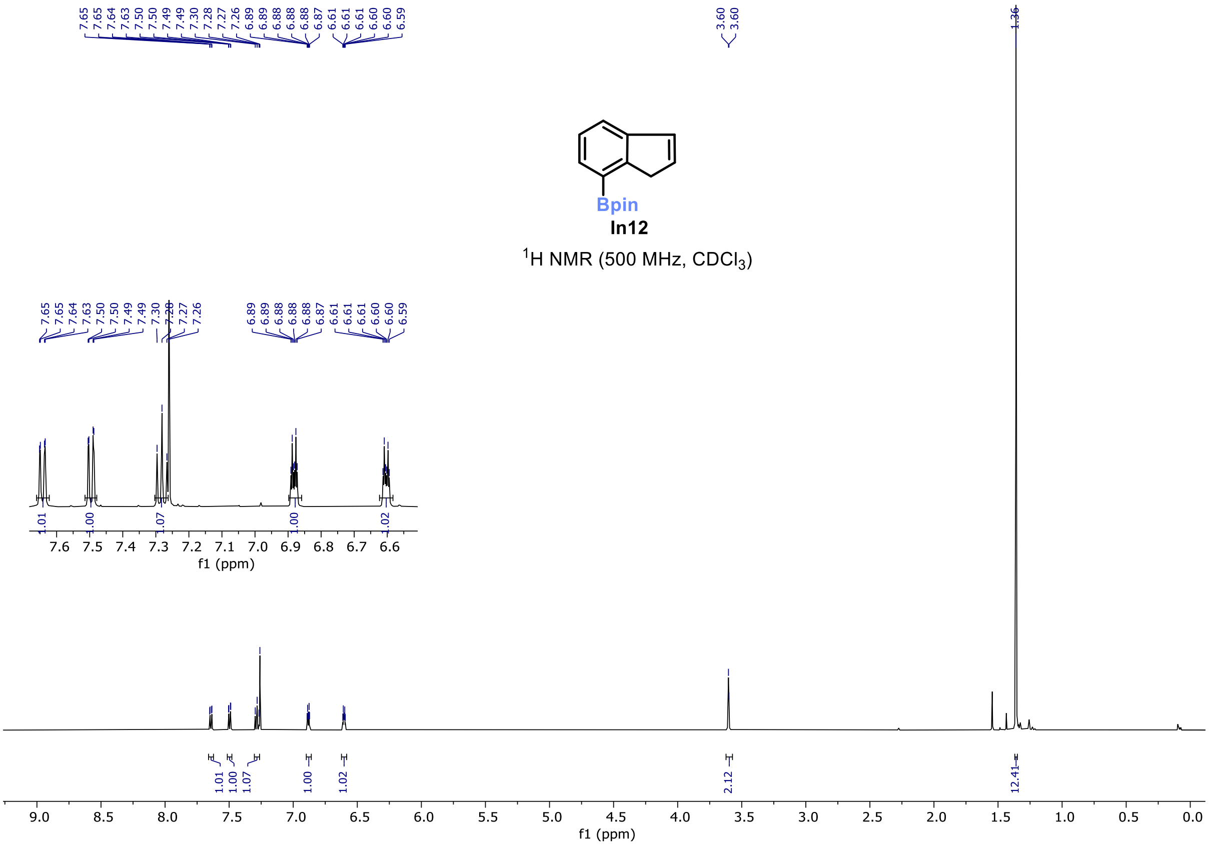

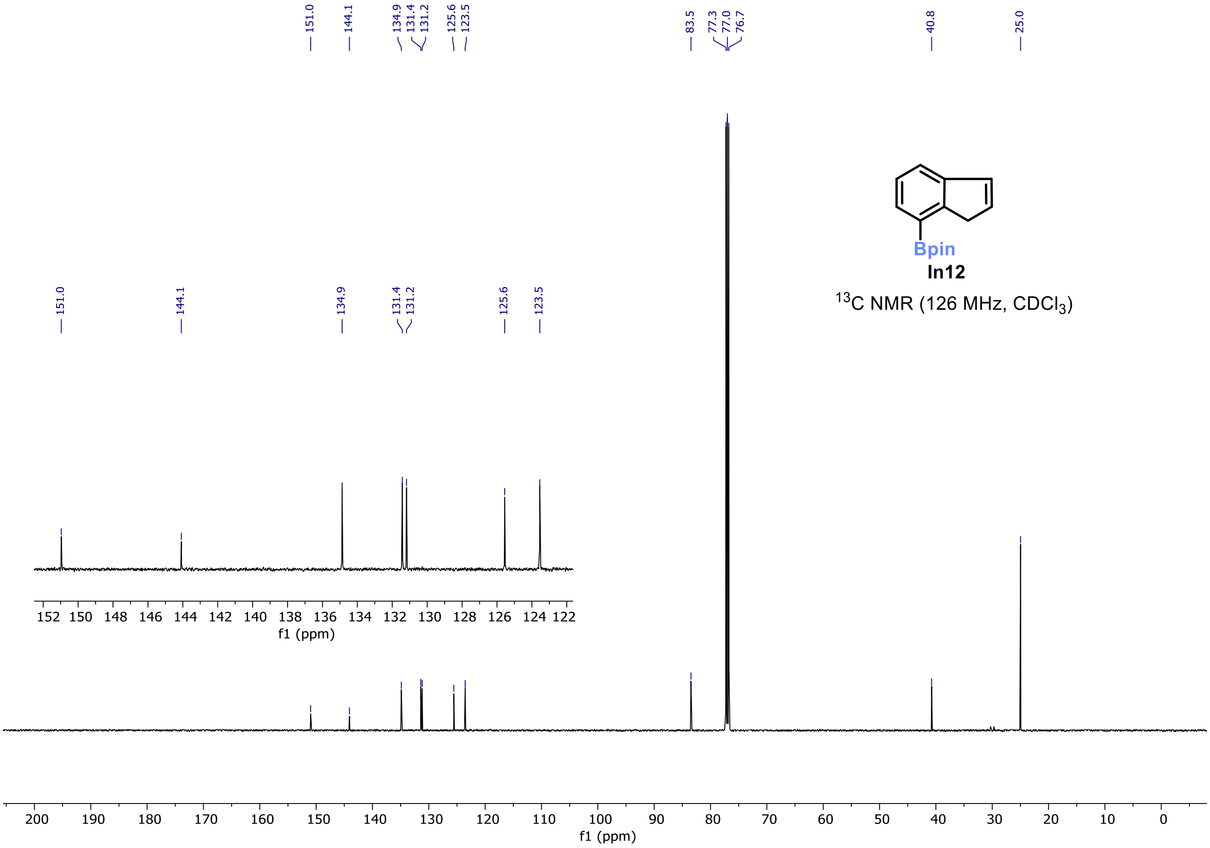

1*H*-Indene-6-carbonitrile, In13

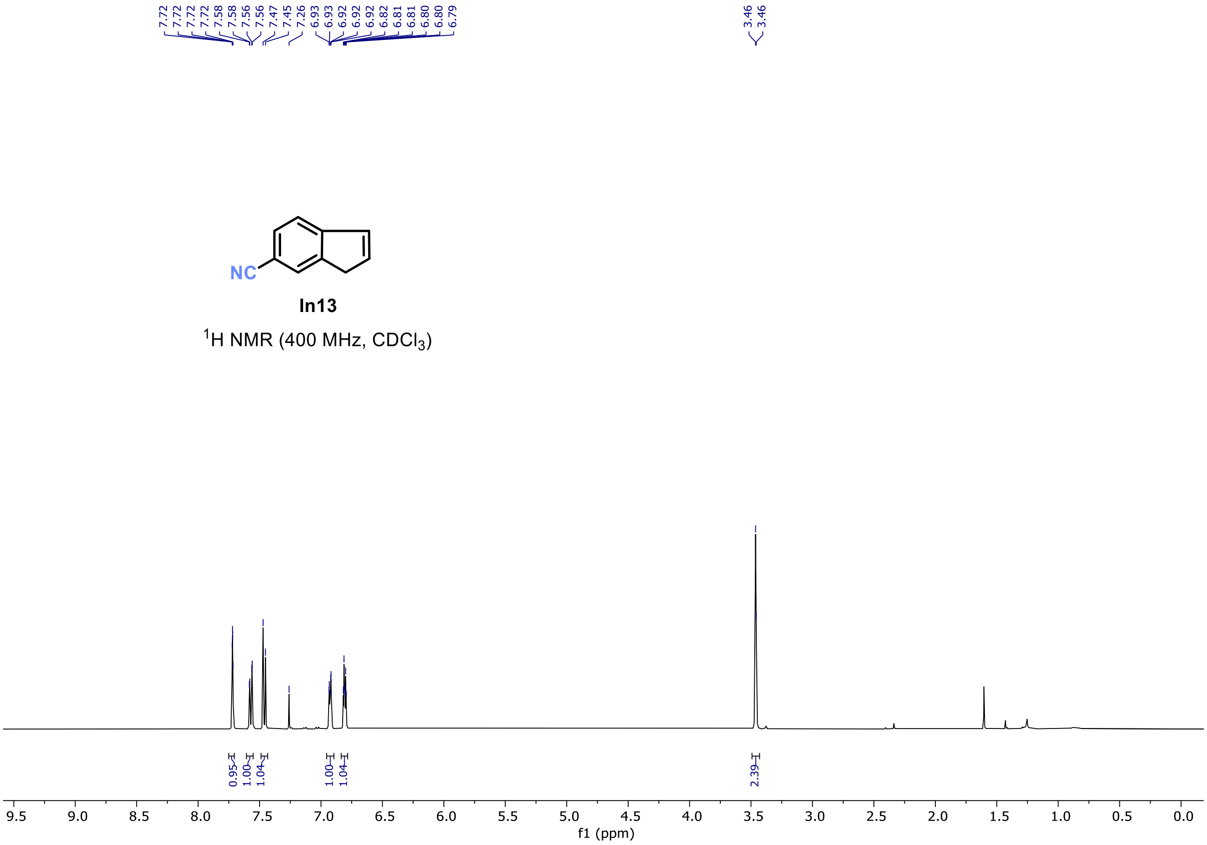

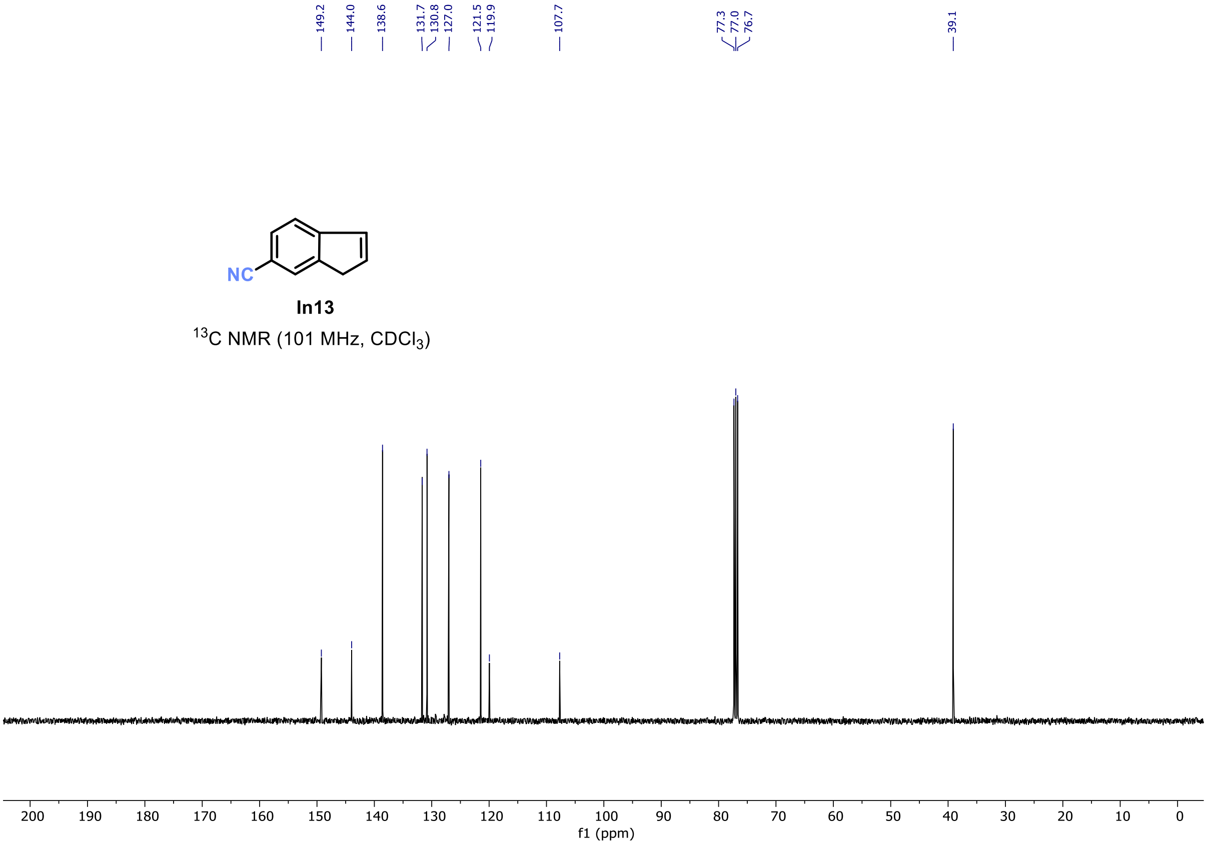

Methyl 4-(1*H*-inden-6-yl)benzoate, In14

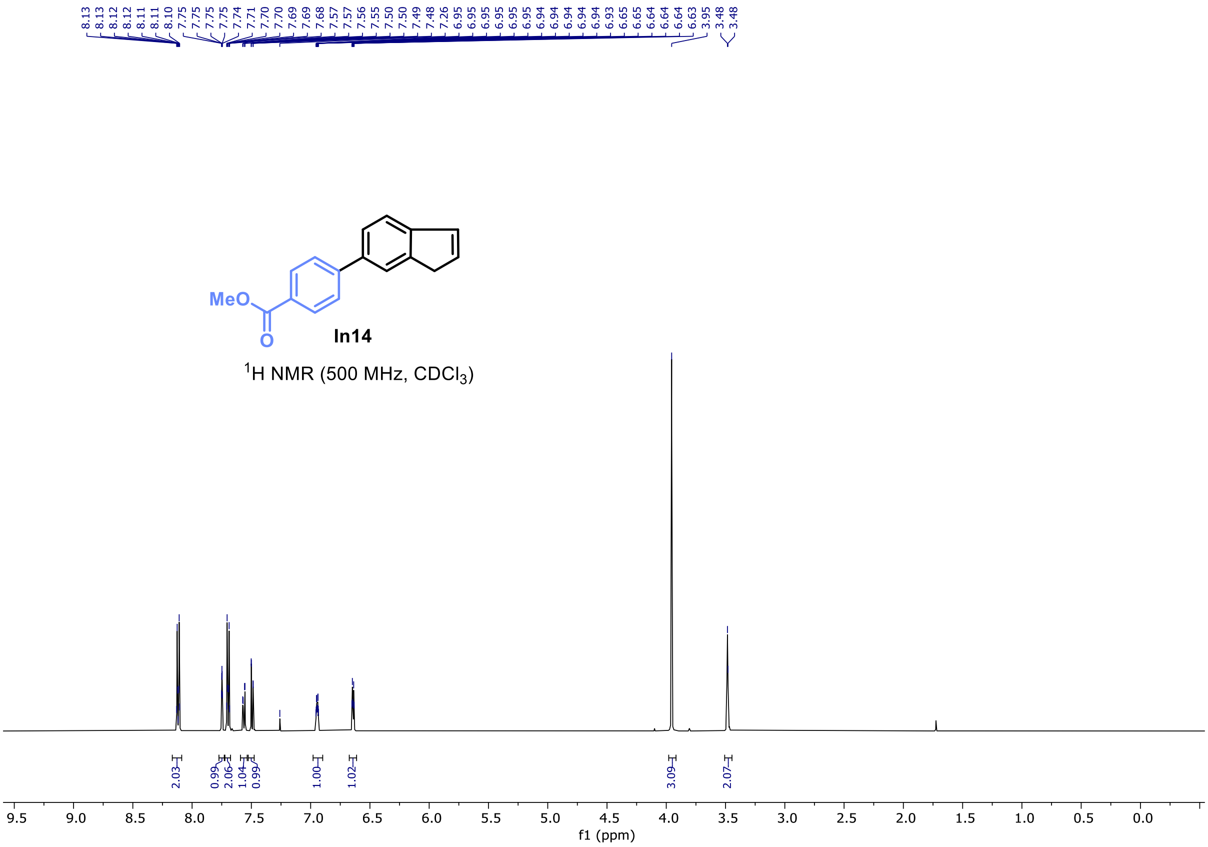

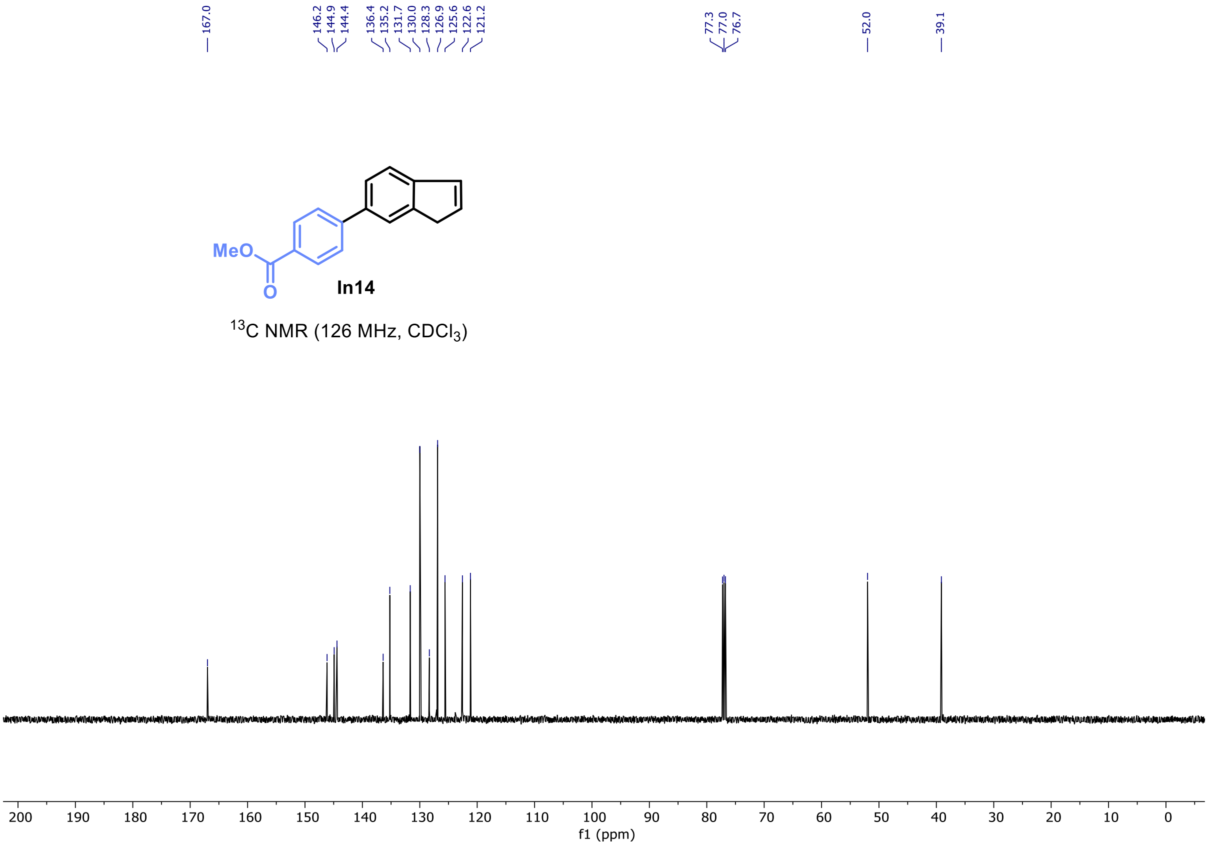

6-(4-(Trifluoromethyl)phenyl)-1*H*-indene, In15

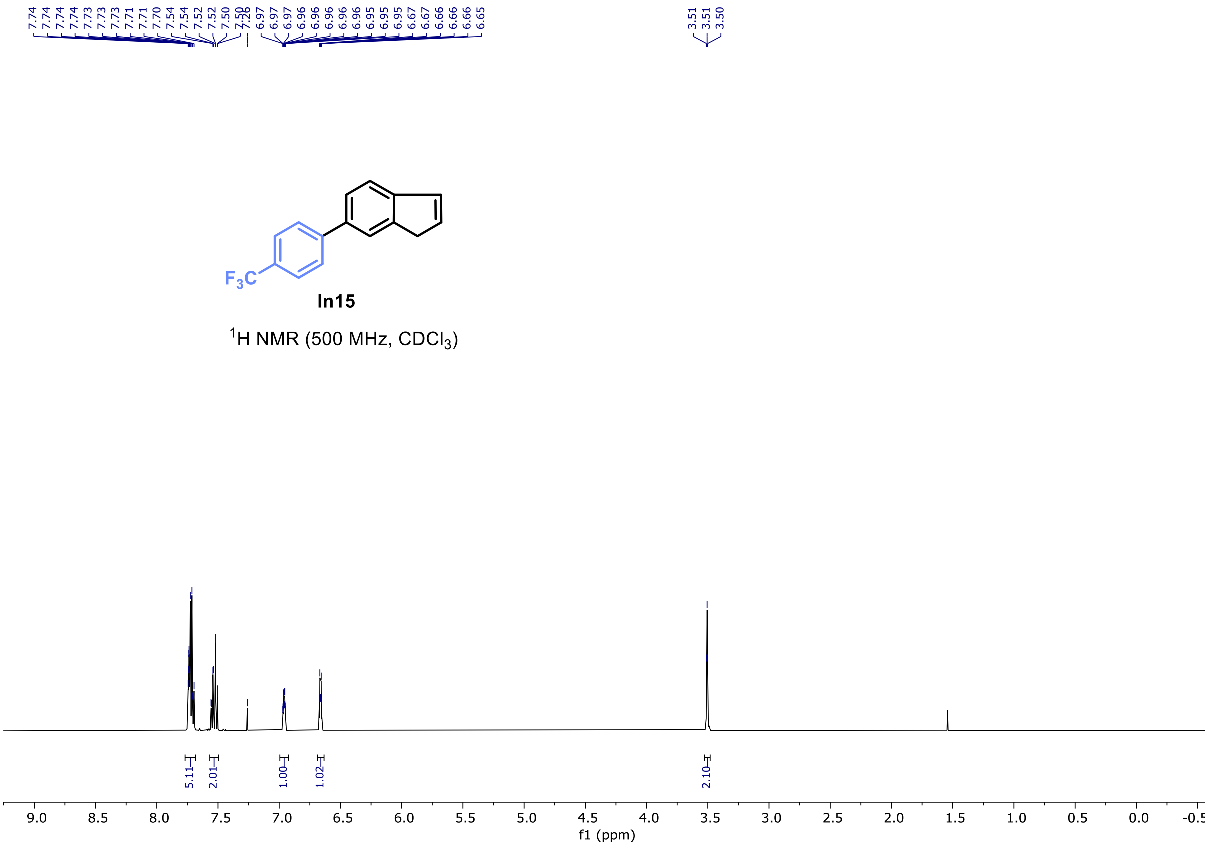

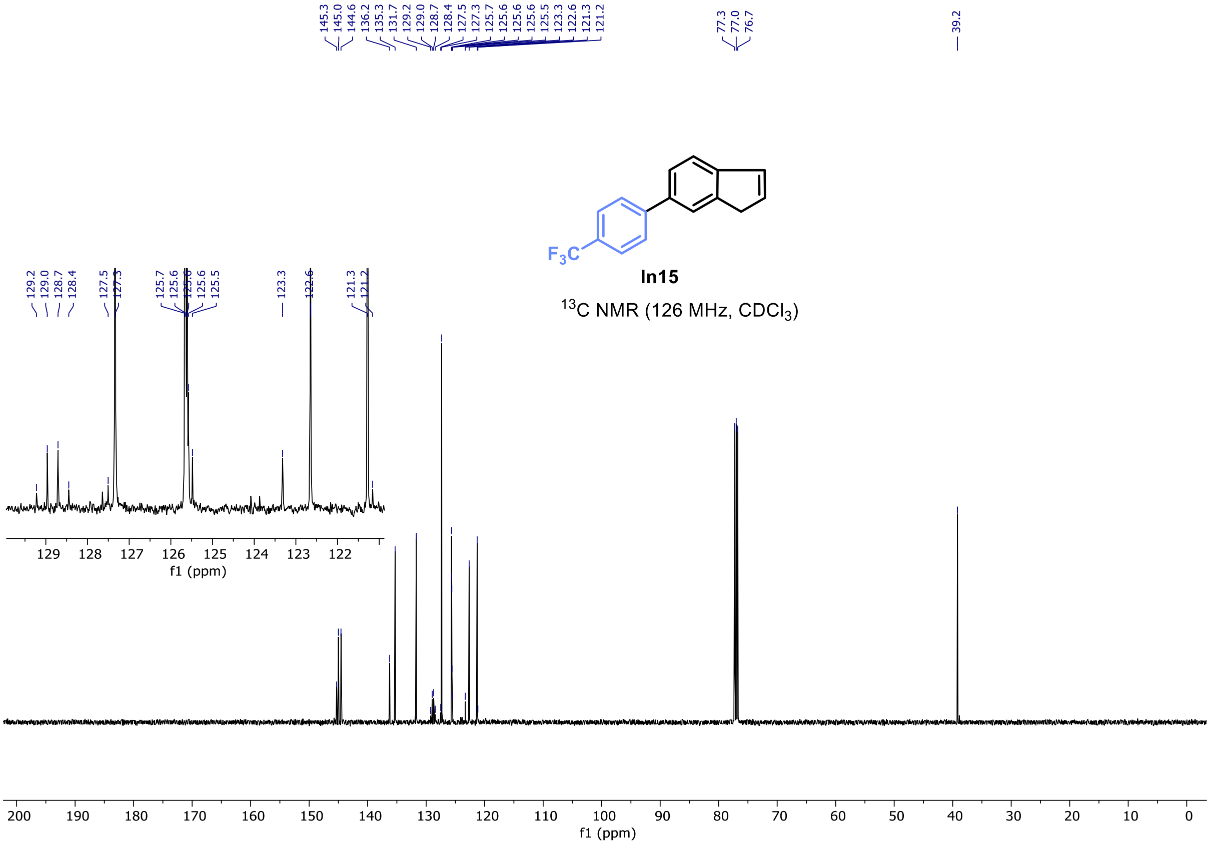

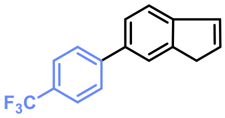

In15

^{19}F NMR (377 MHz, $CDCl_3$)

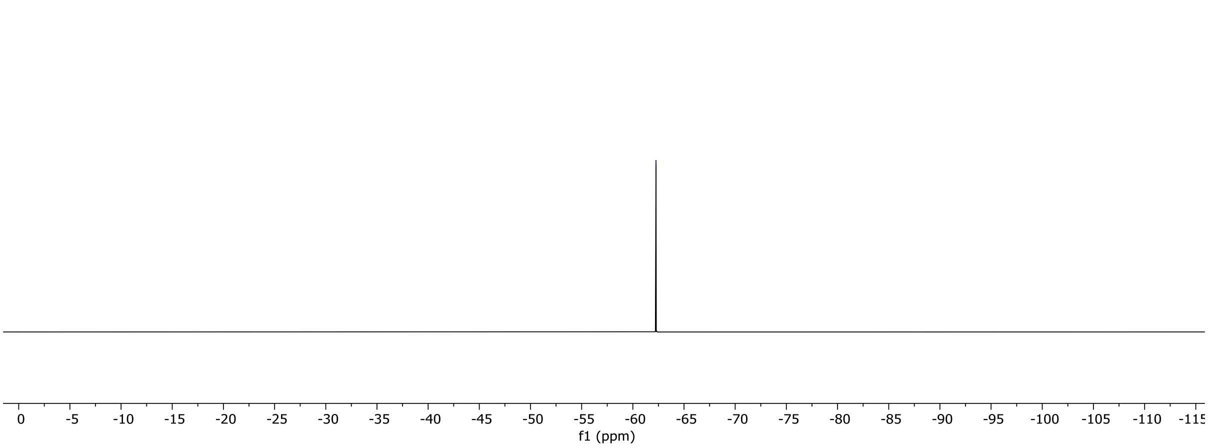

((4*R*,5*R*)-2,2-Diisopropyl-1,3-dioxolane-4,5-diyl)bis(di(naphthalen-1-yl)methanol), L6

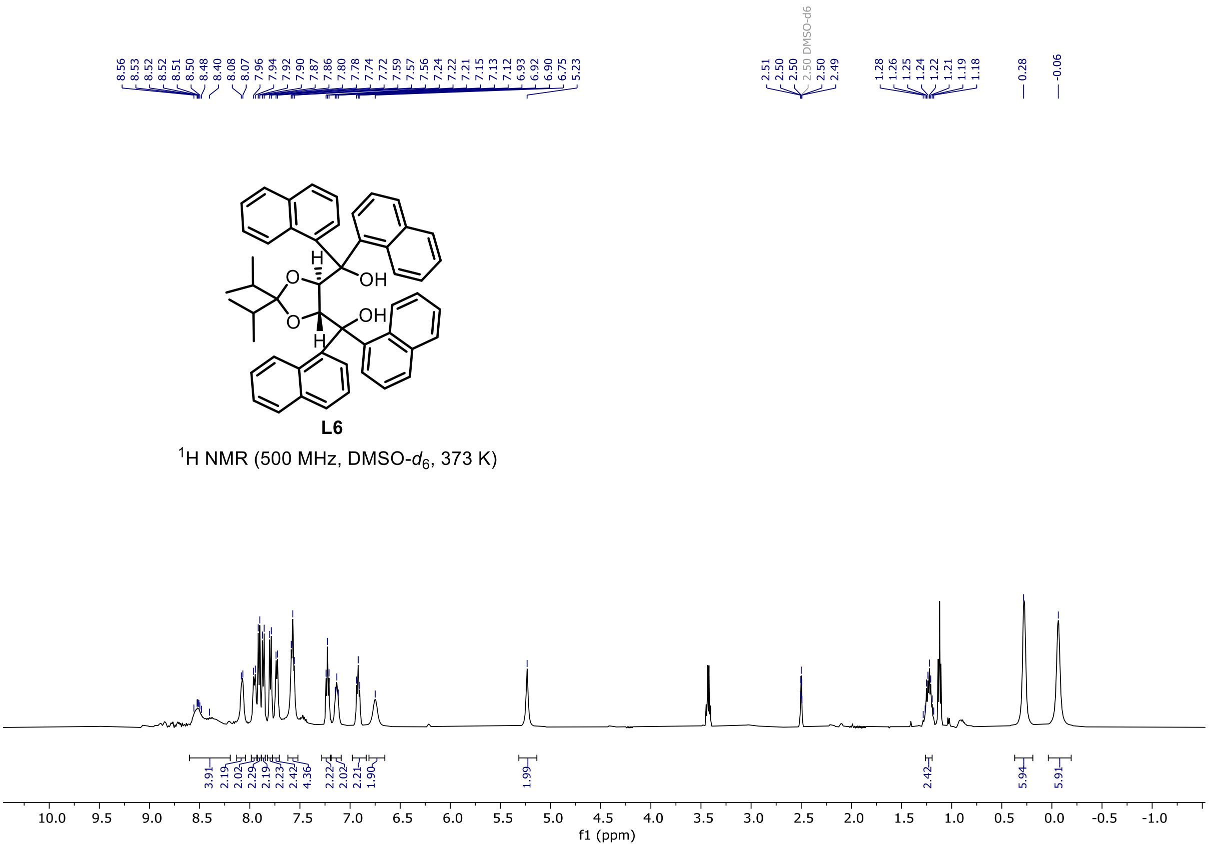

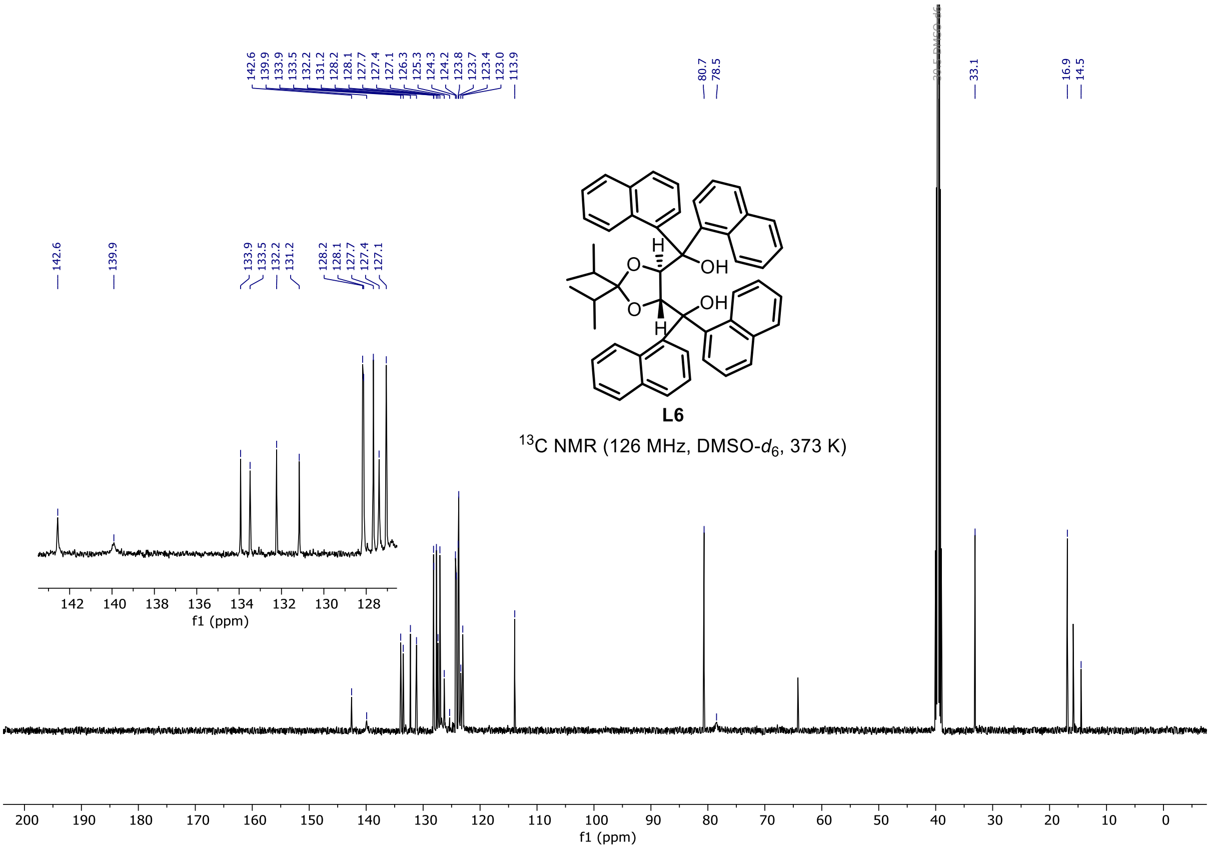

3-(5-Chlorothiophene-2-carbonyl)oxazolidin-2-one, S1

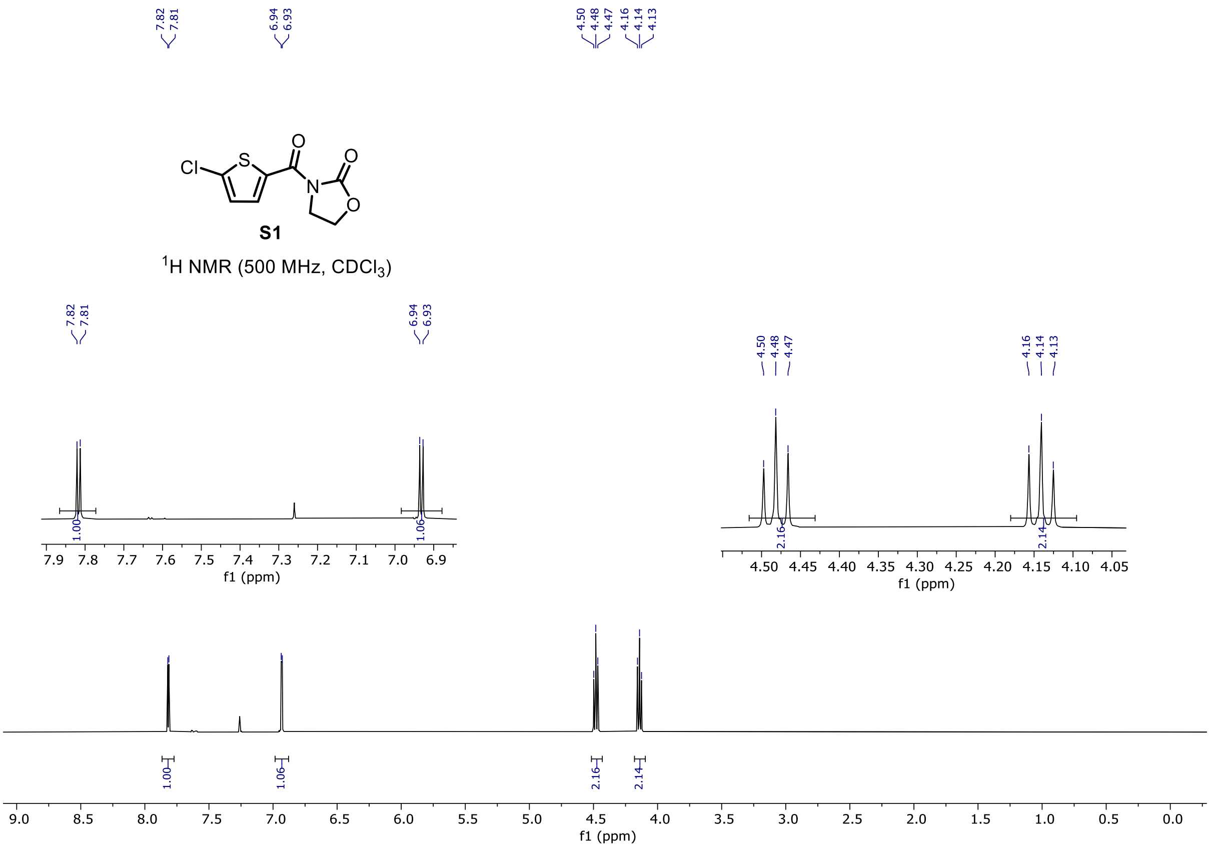

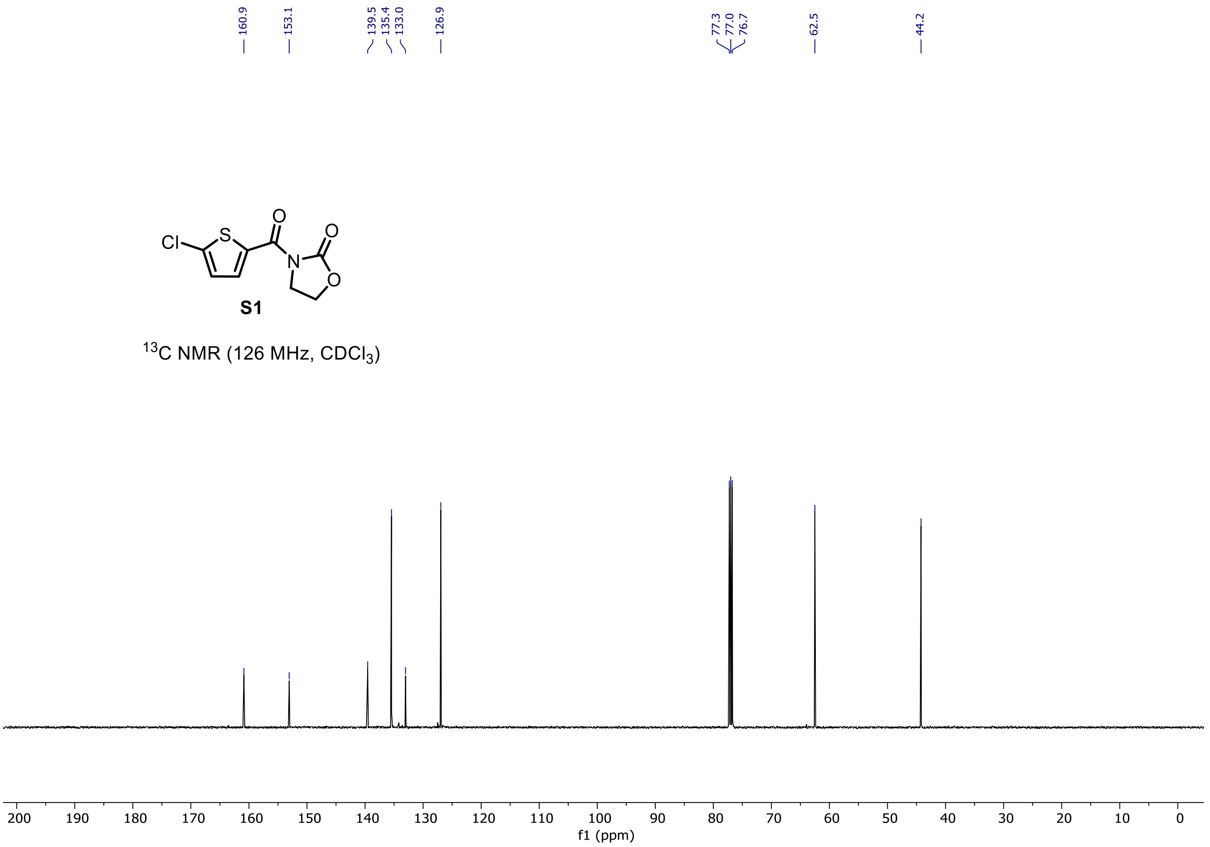

3-(5-Chloro-1,1-dioxidothiophene-2-carbonyl)oxazolidin-2-one, 1a

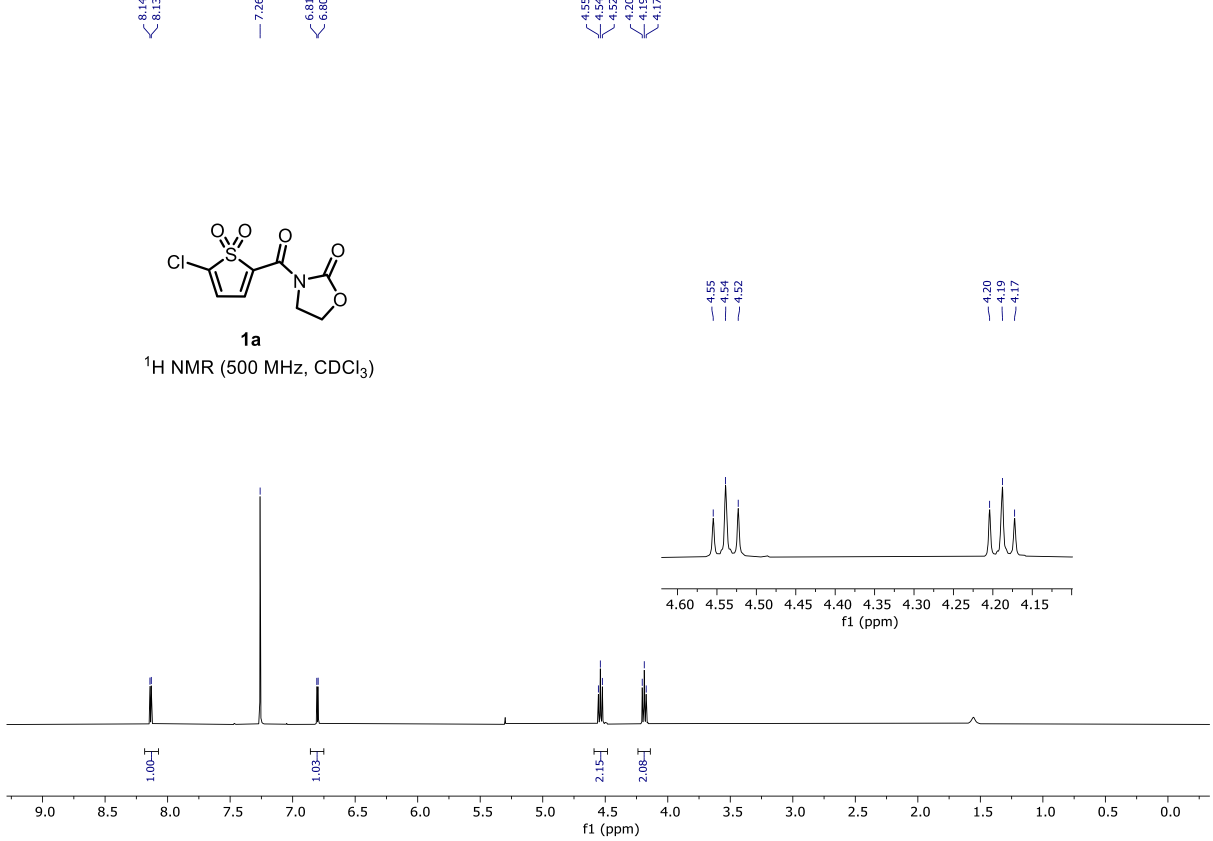

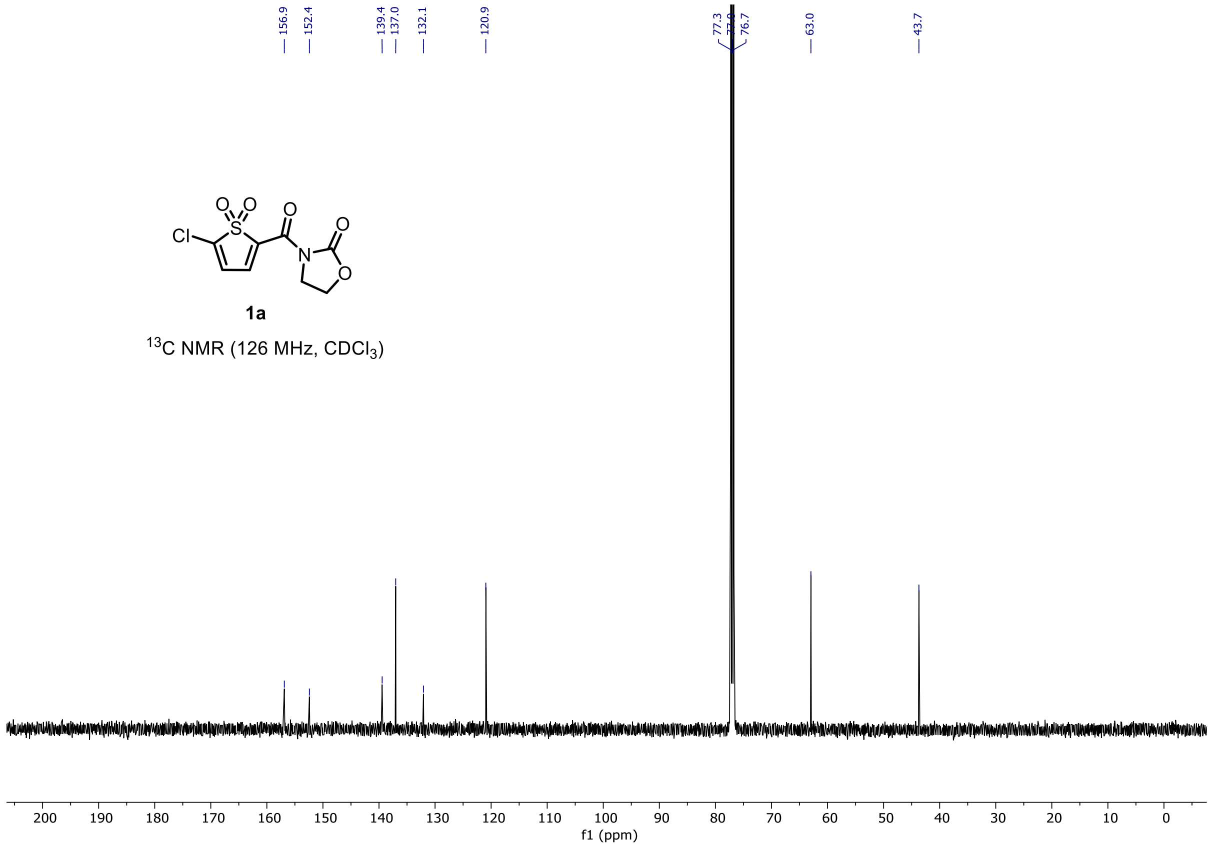

3-(5-Methylthiophene-2-carbonyl)oxazolidin-2-one, S3

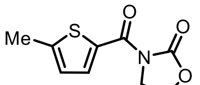

S3

^{1}H NMR (400 MHz, $CDCl_3$)

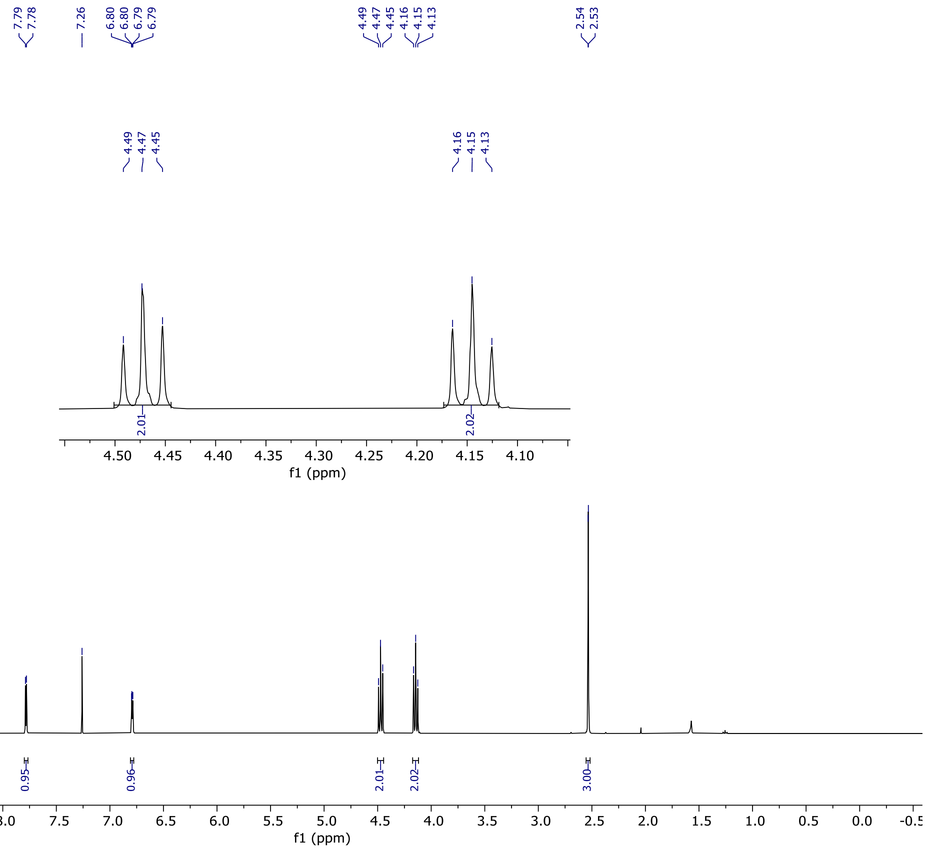

S3

^{13}C NMR (101 MHz, $CDCl_3$)

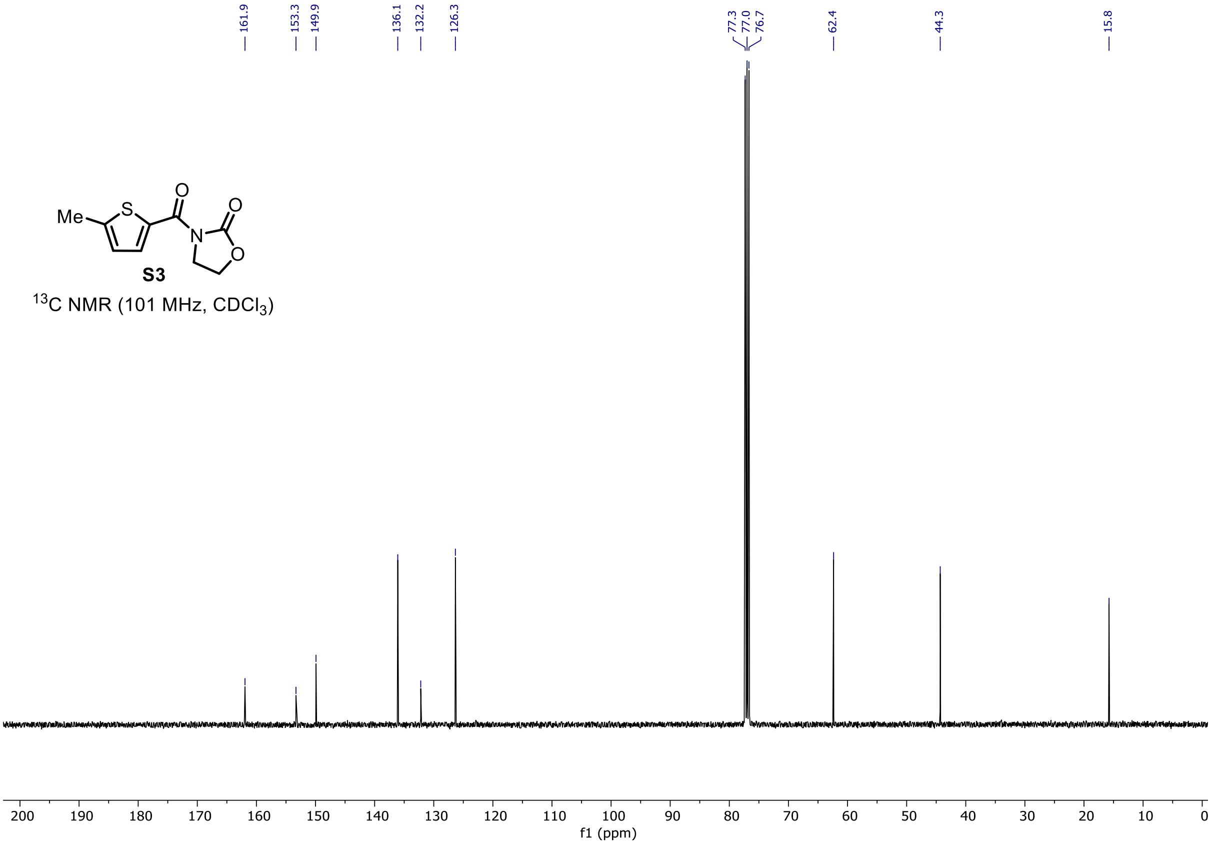

3-(5-Bromothiophene-2-carbonyl)oxazolidin-2-one, S4

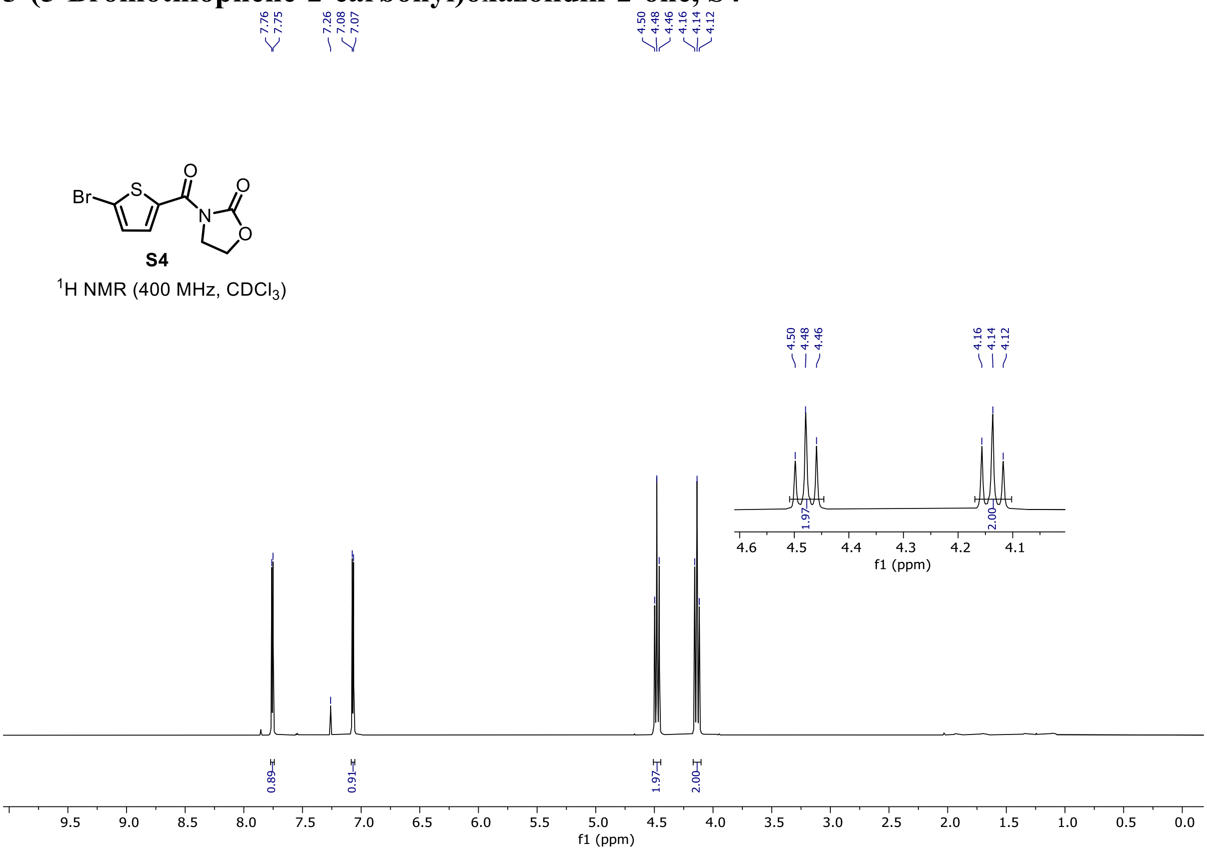

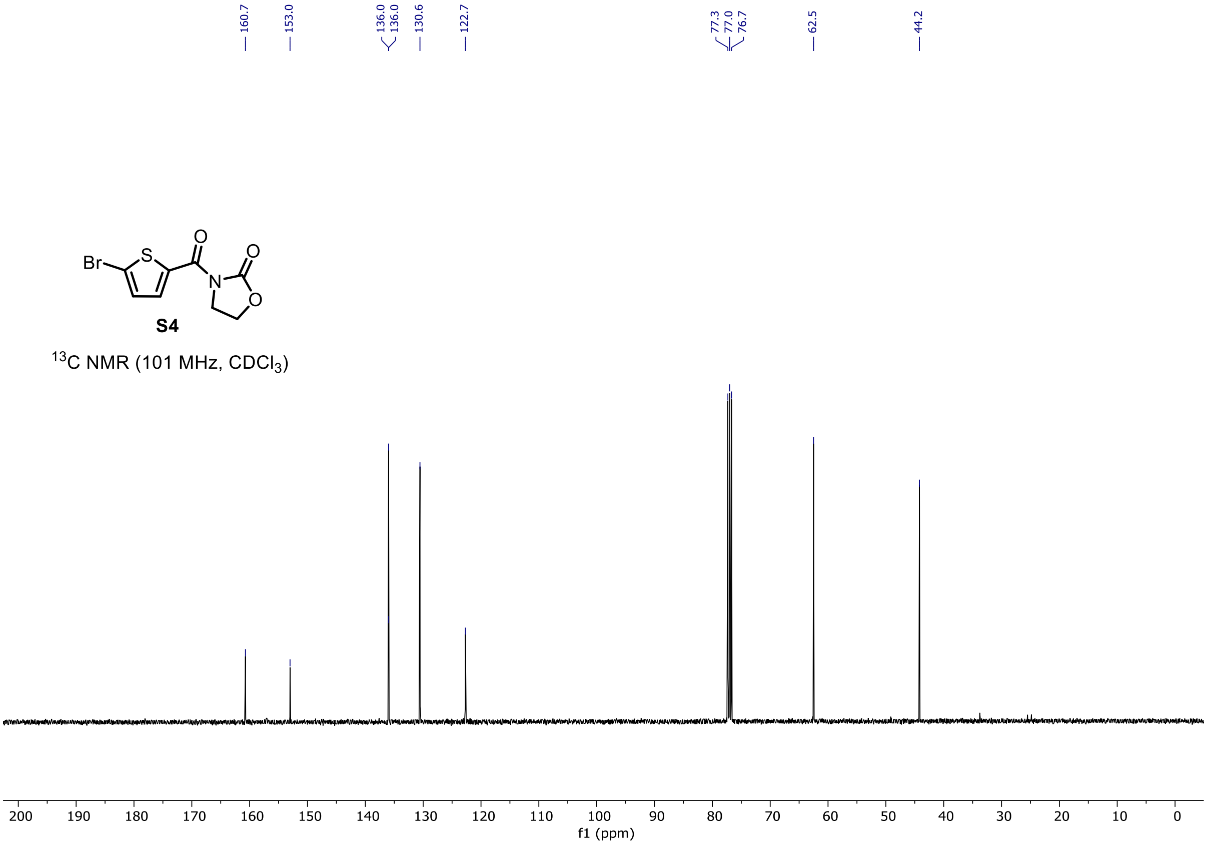

3-(5-Bromo-4-methylthiophene-2-carbonyl)oxazolidin-2-one, S5

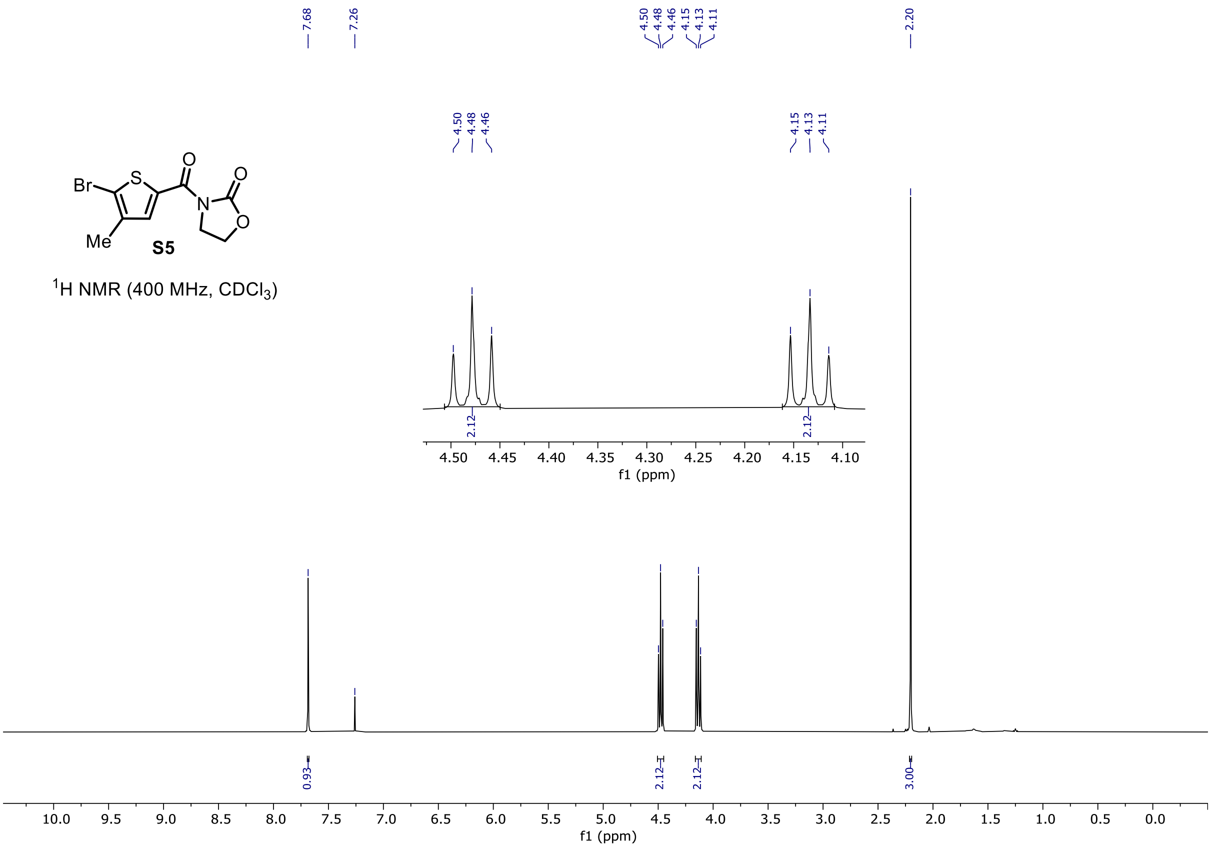

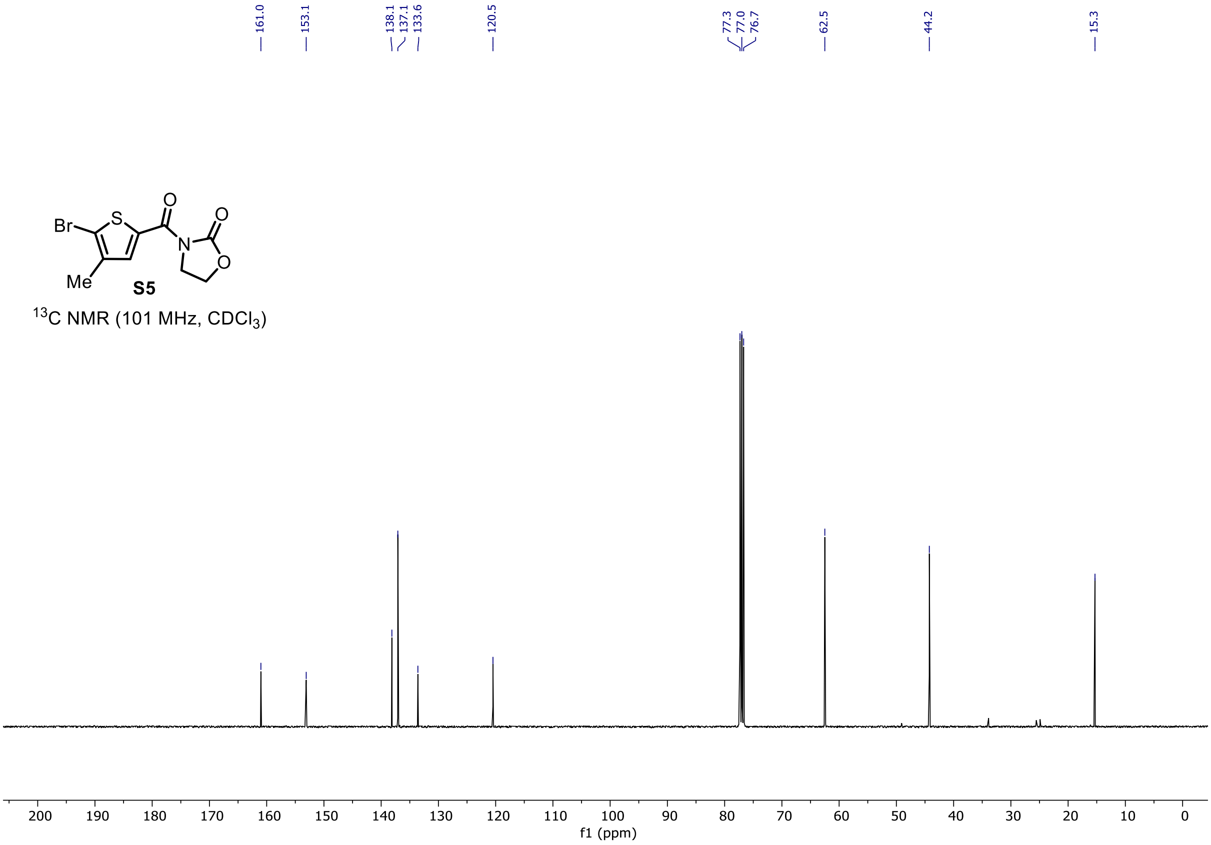

3-(5,6-Dihydro-4*H*-cyclopenta[*b*]thiophene-2-carbonyl)oxazolidin-2-one, S6

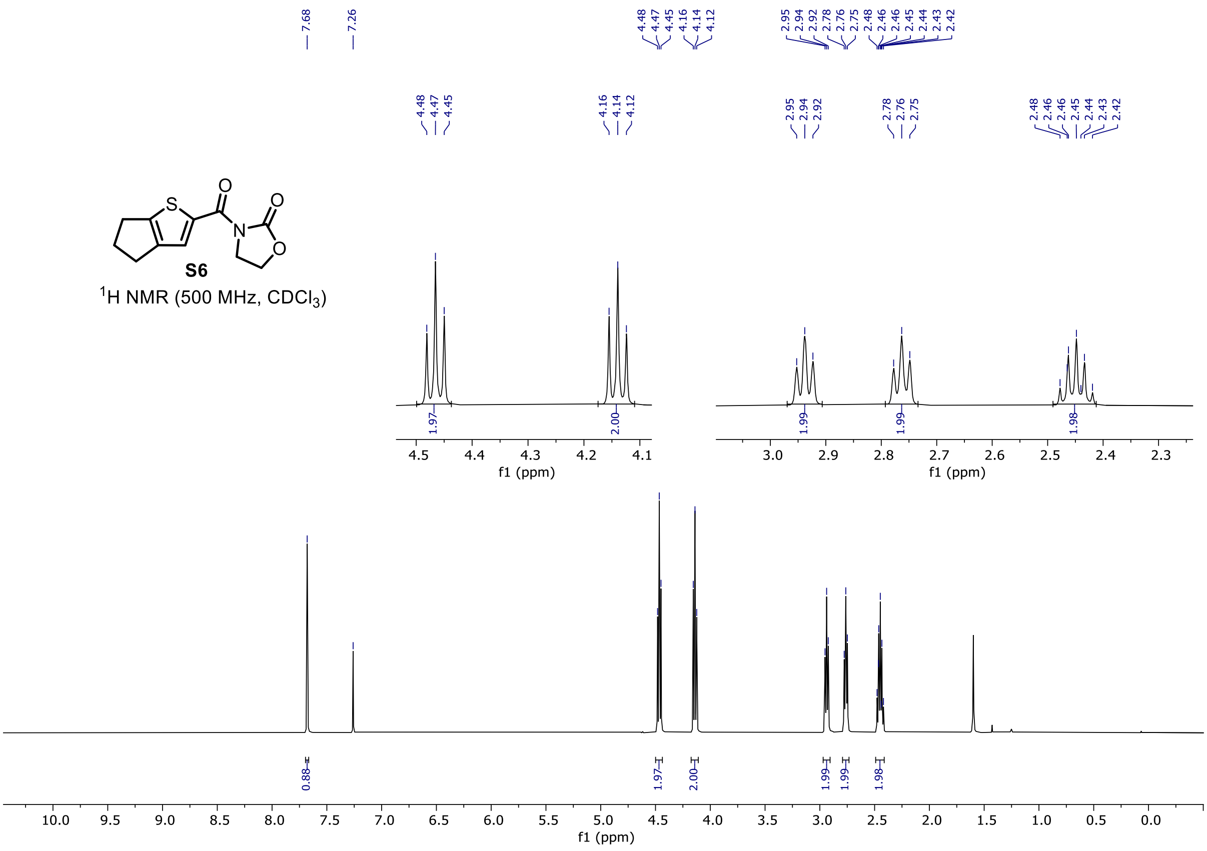

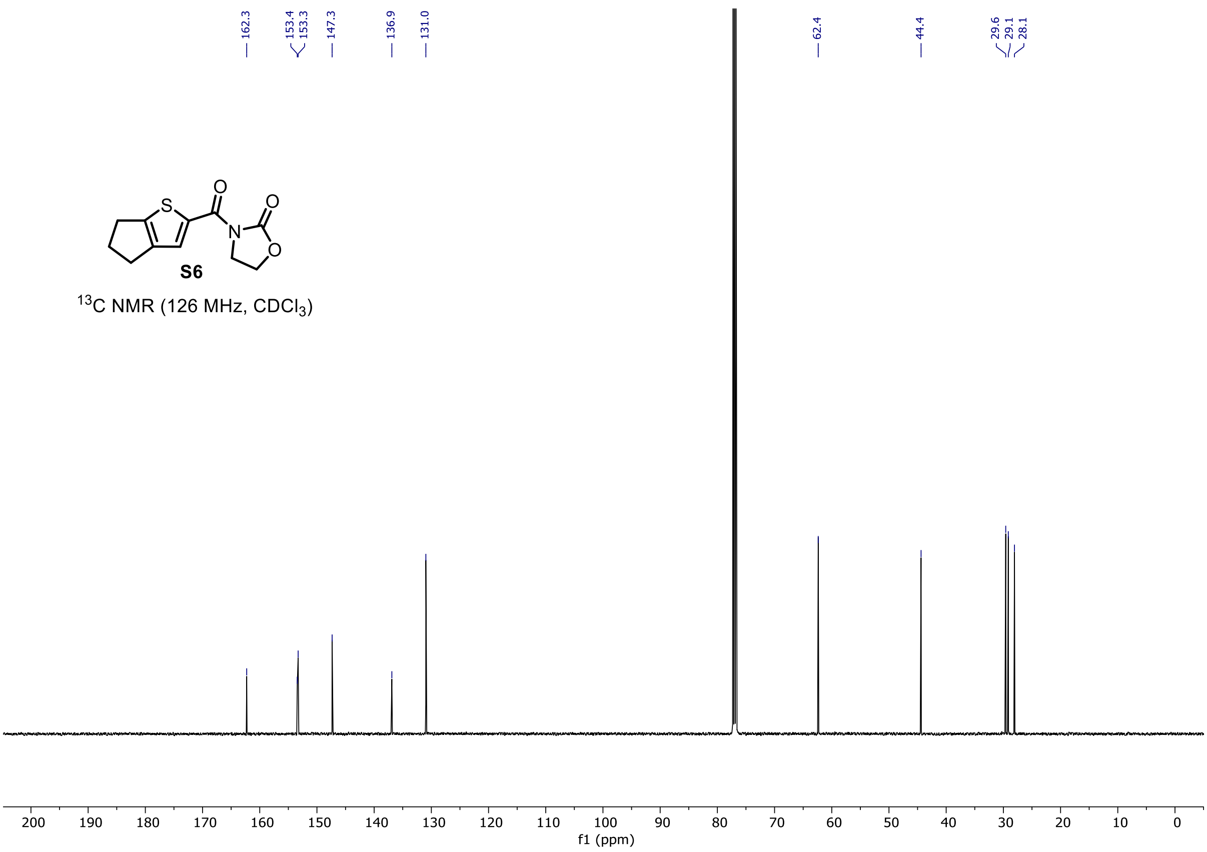

3-(5-Methyl-1,1-dioxidothiophene-2-carbonyl)oxazolidin-2-one, 1b

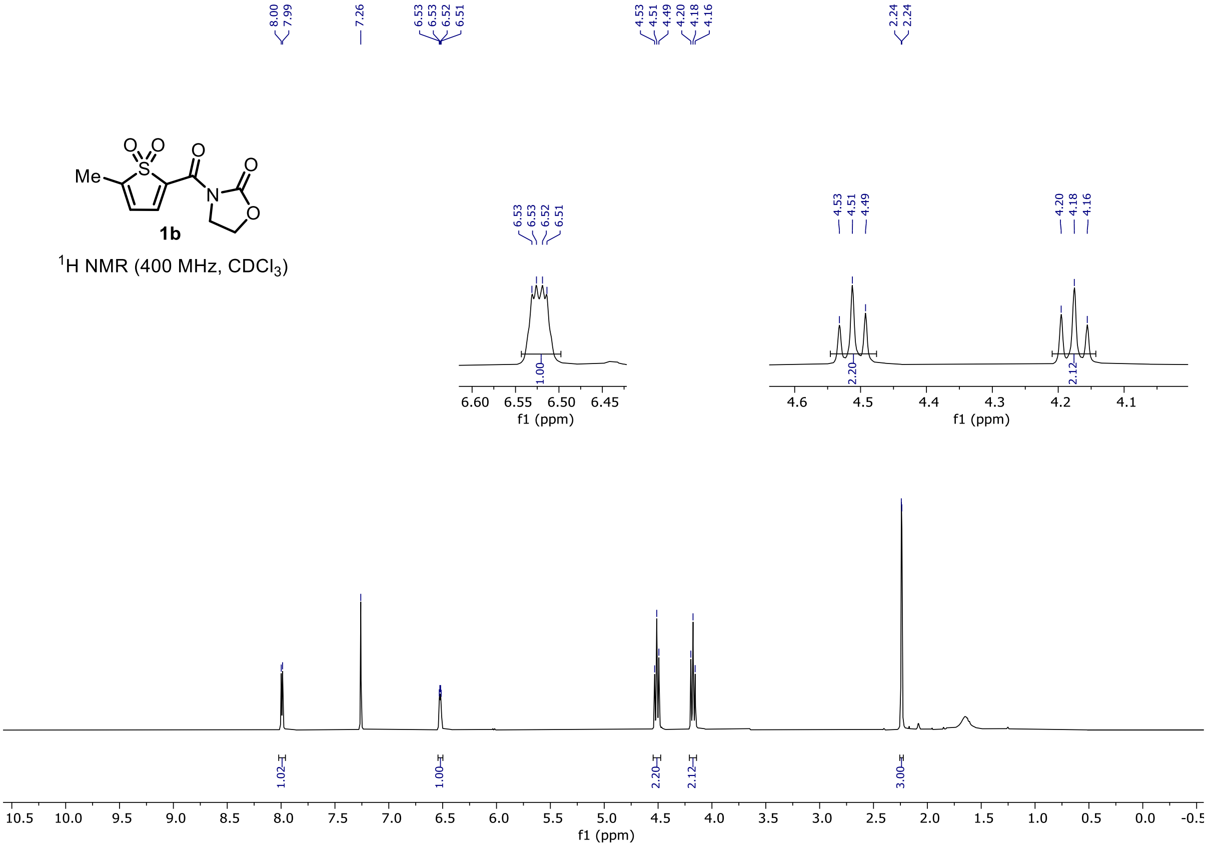

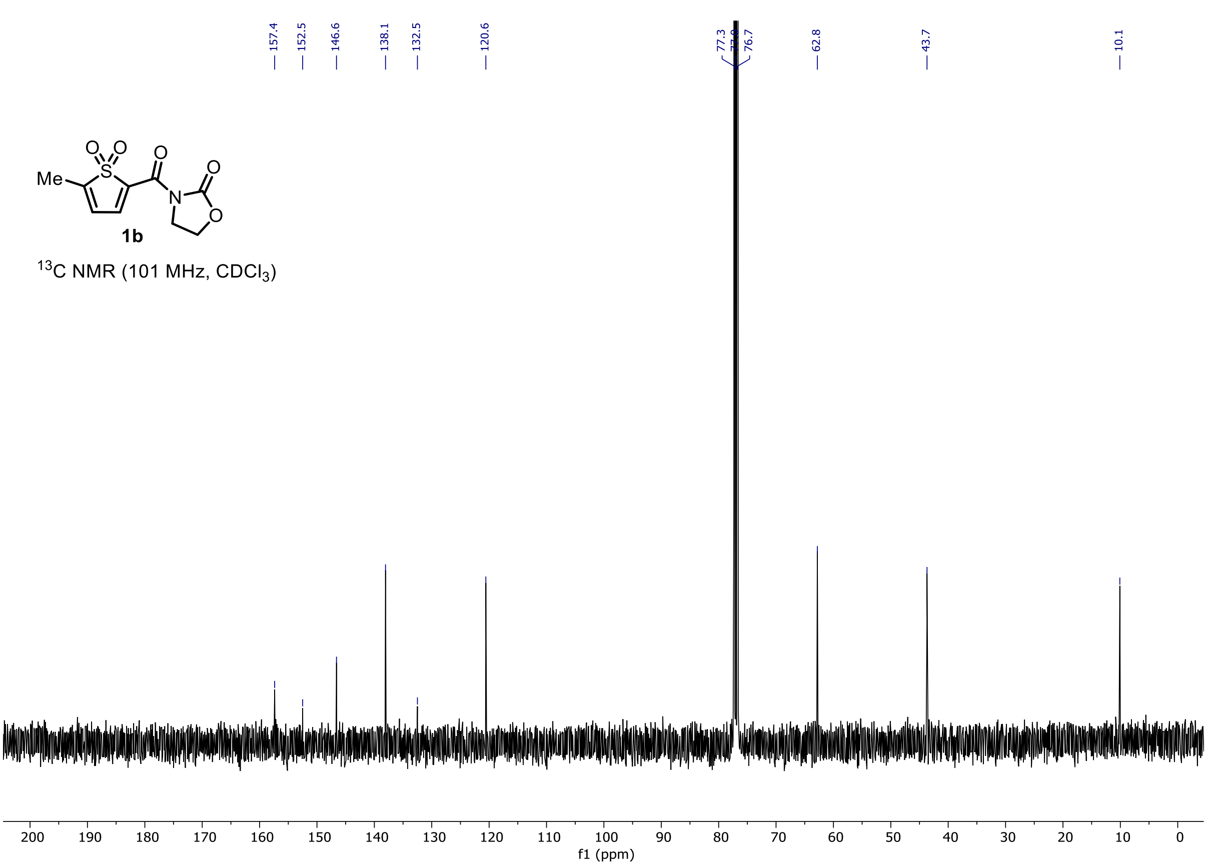

3-(5-Bromo-1,1-dioxidothiophene-2-carbonyl)oxazolidin-2-one, 1c

1c

^{1}H NMR (400 MHz, $CDCl_3$)

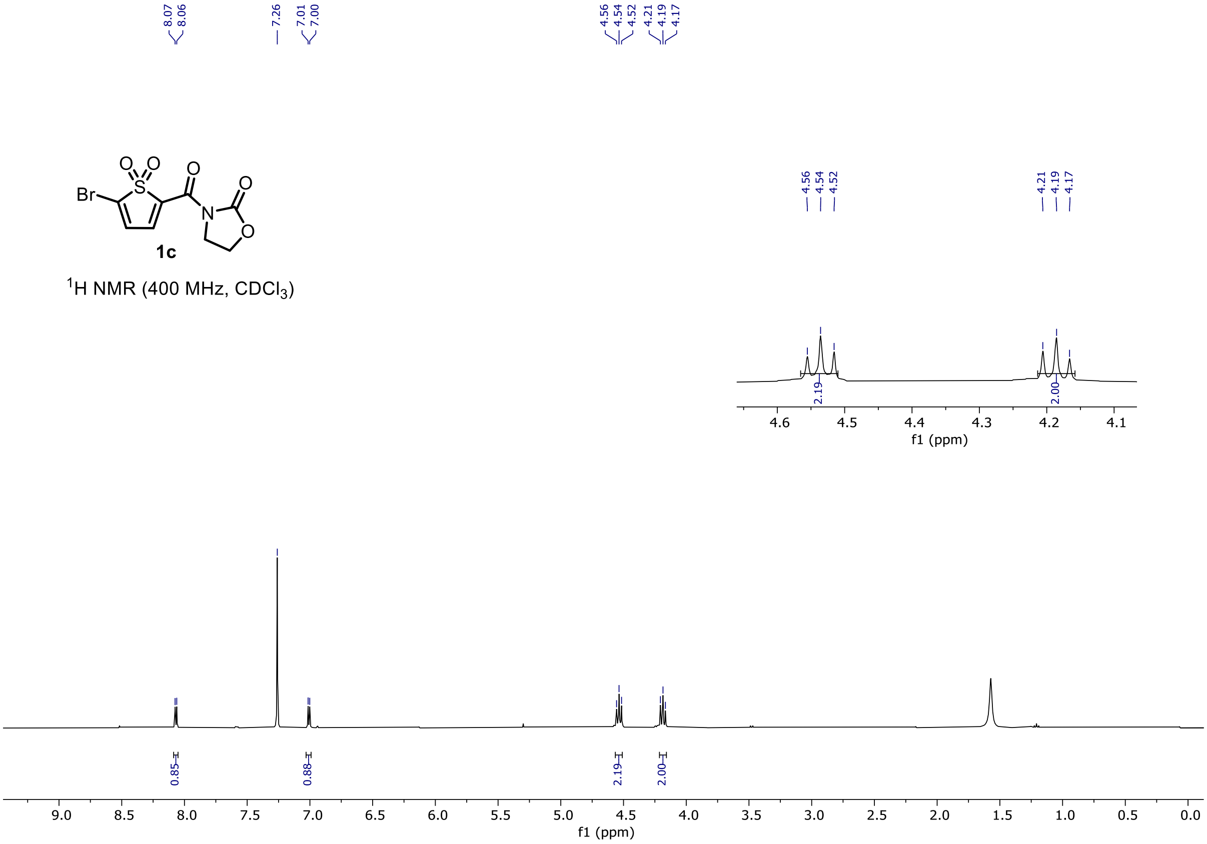

1c

^{13}C NMR (101 MHz, $CDCl_3$)

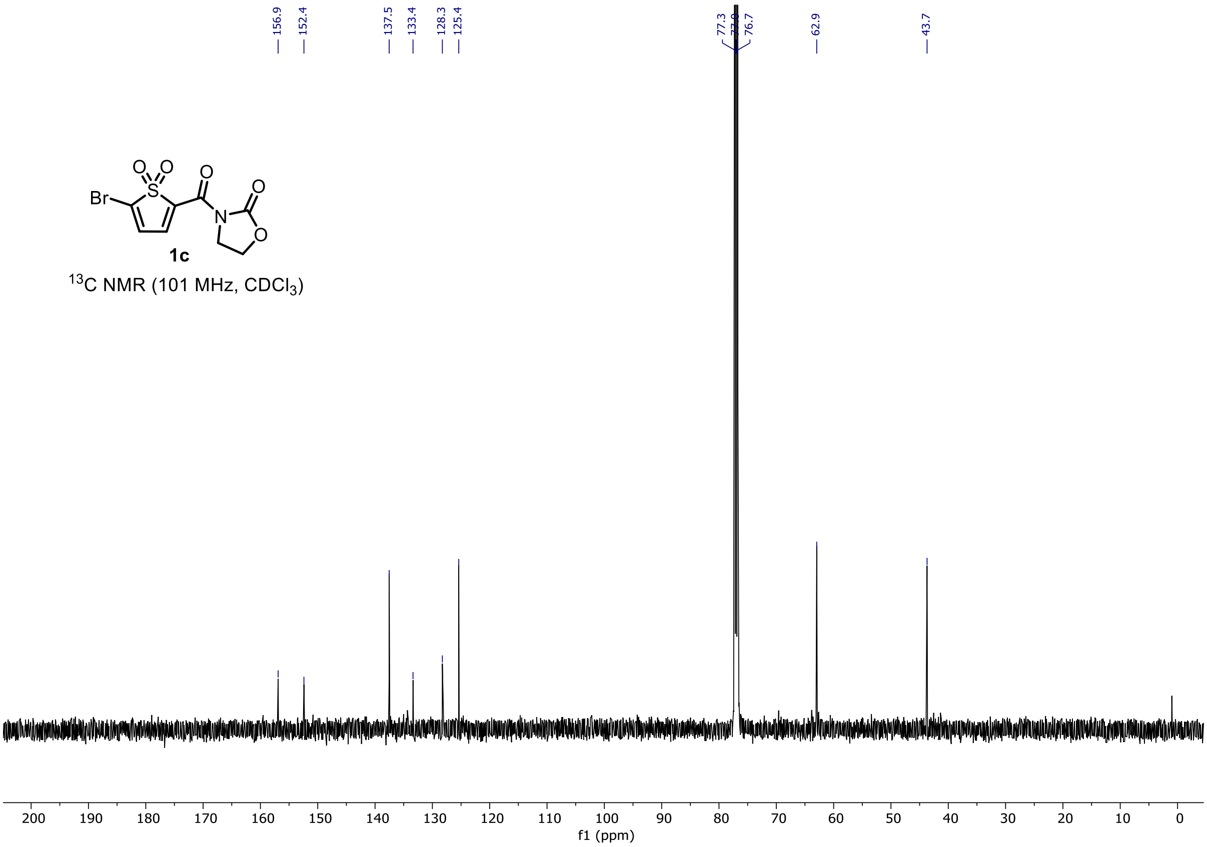

3-(5-Bromo-4-methyl-1,1-dioxidothiophene-2-carbonyl)oxazolidin-2-one, 1d

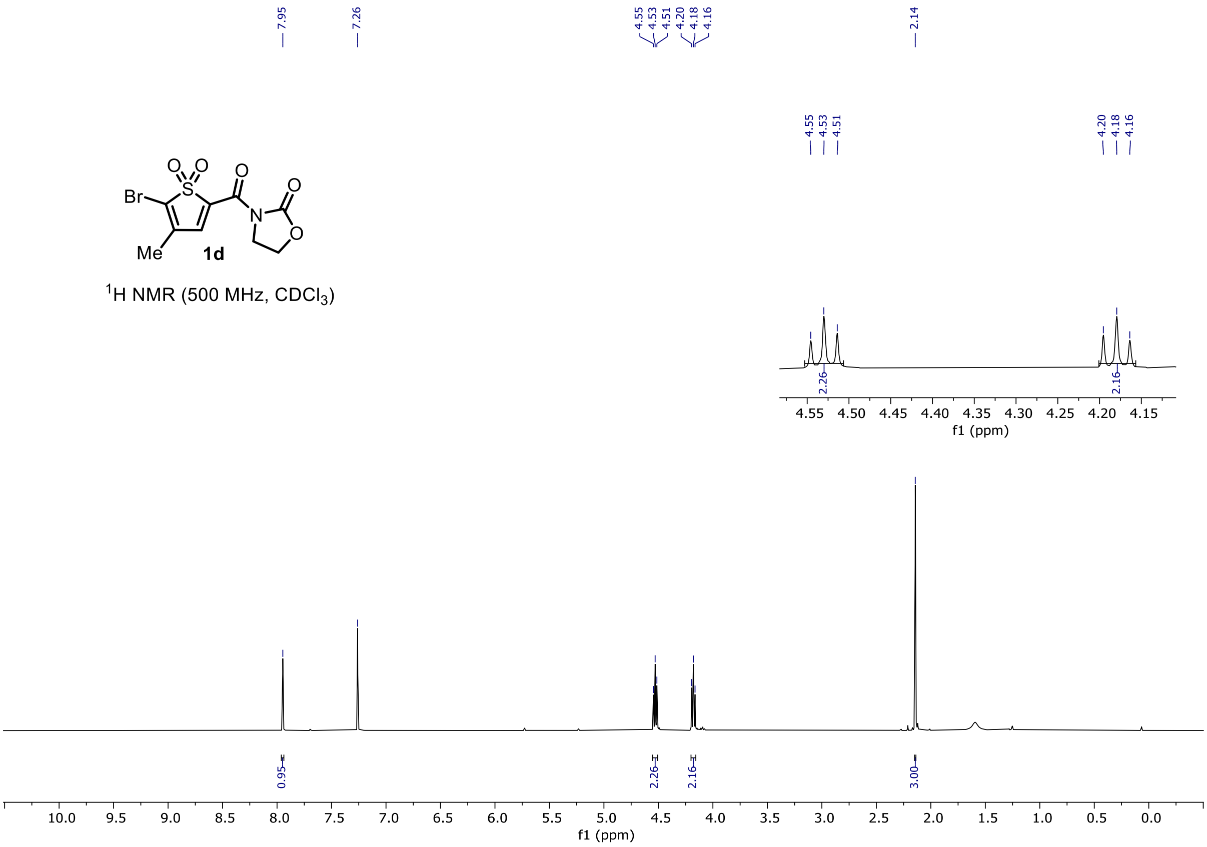

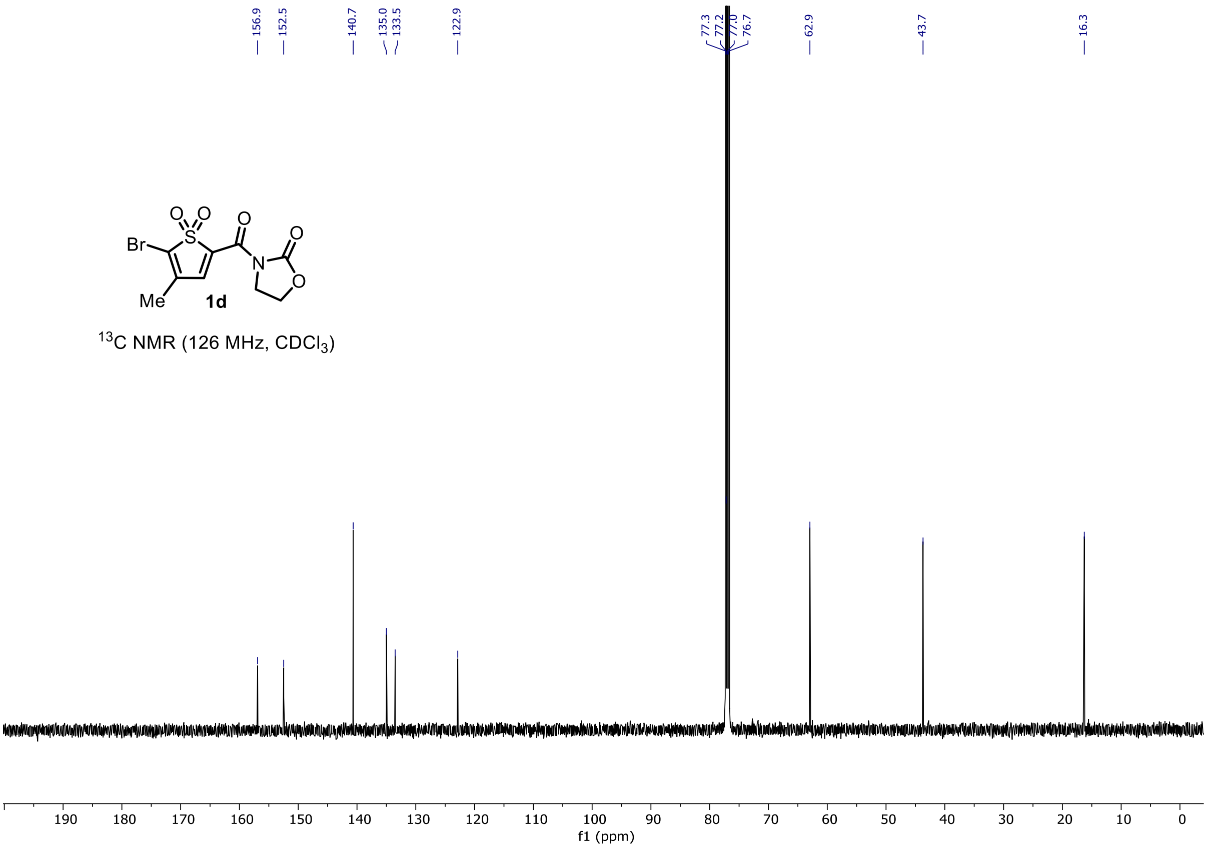

3-(1,1-Dioxido-5,6-dihydro-4*H*-cyclopenta[*b*]thiophene-2-carbonyl)oxazolidin-2-one, 1e

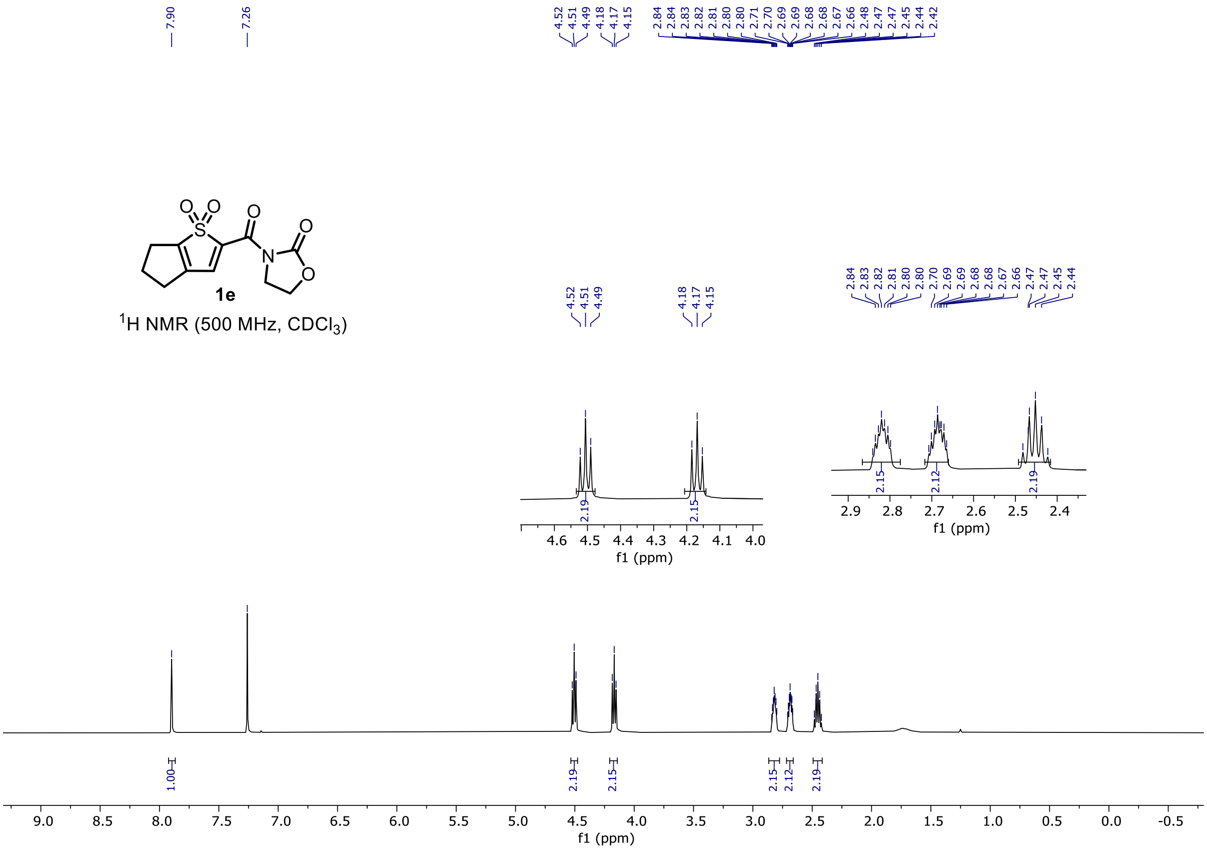

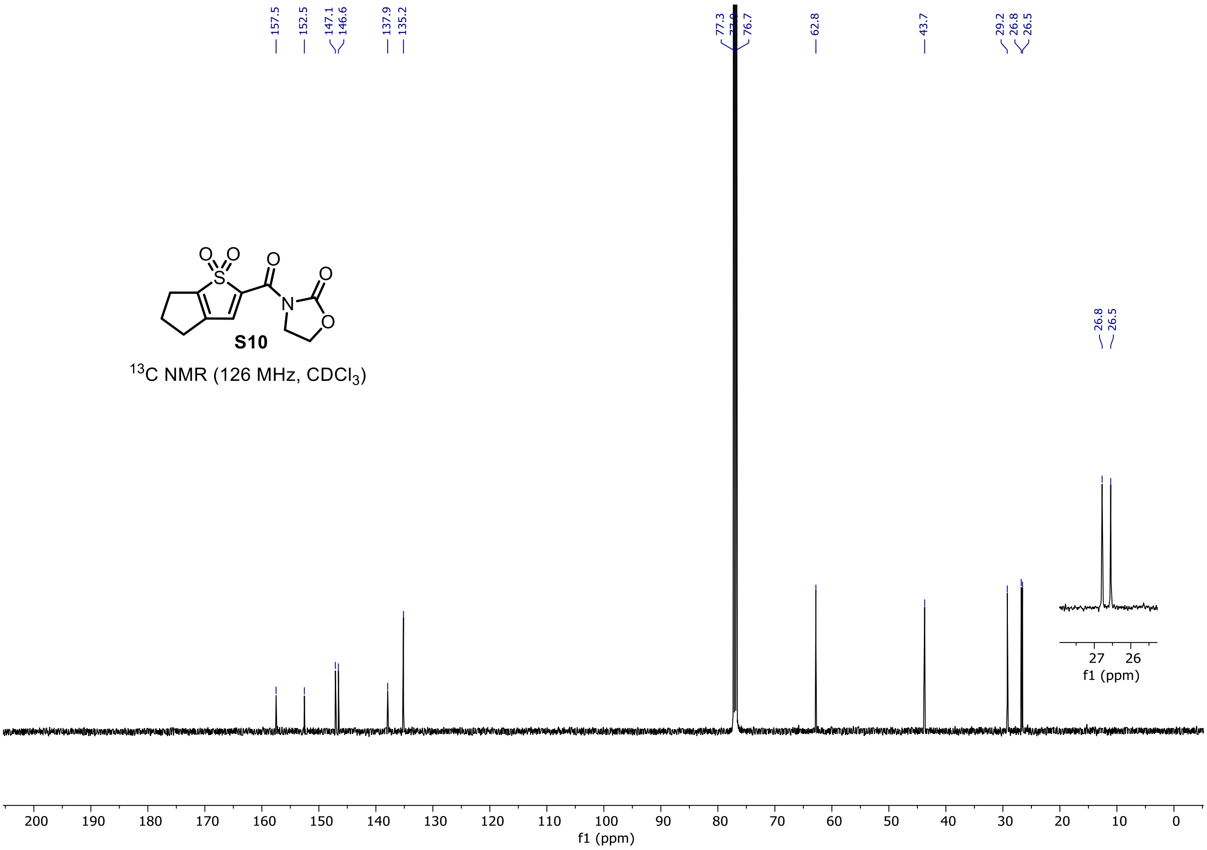

3-((4a*R*,9a*R*)-1-Chloro-4a,9a-dihydro-9*H*-fluorene-4-carbonyl)oxazolidin-2-one, 2a

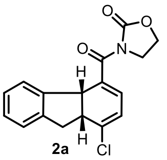

2a

^{1}H NMR (400 MHz, $CDCl_3$)

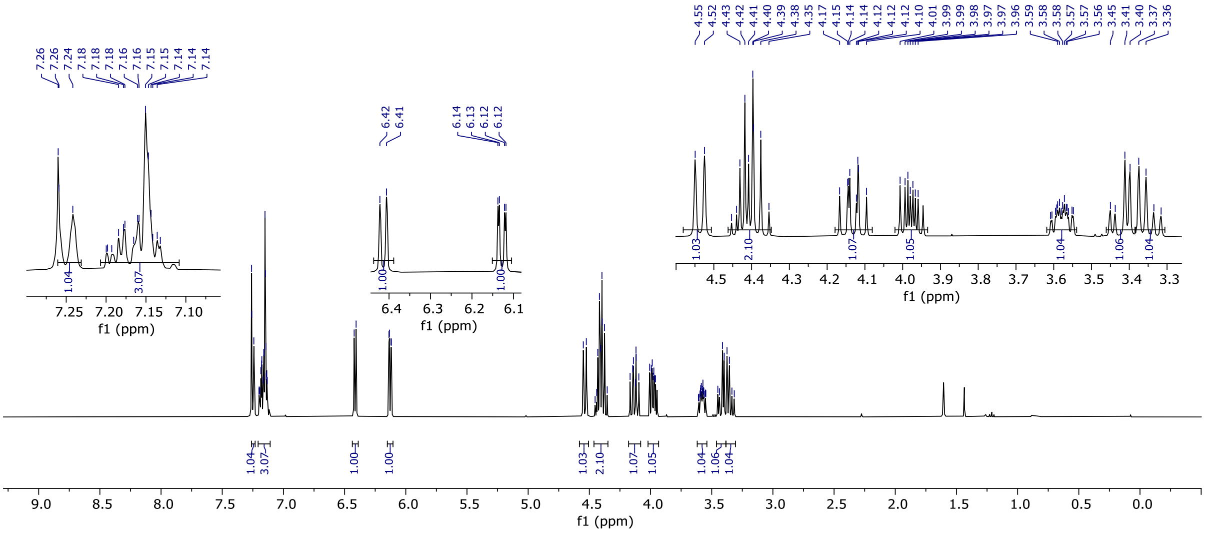

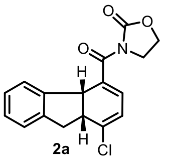

2a

^{13}C NMR (101 MHz, $CDCl_3$)

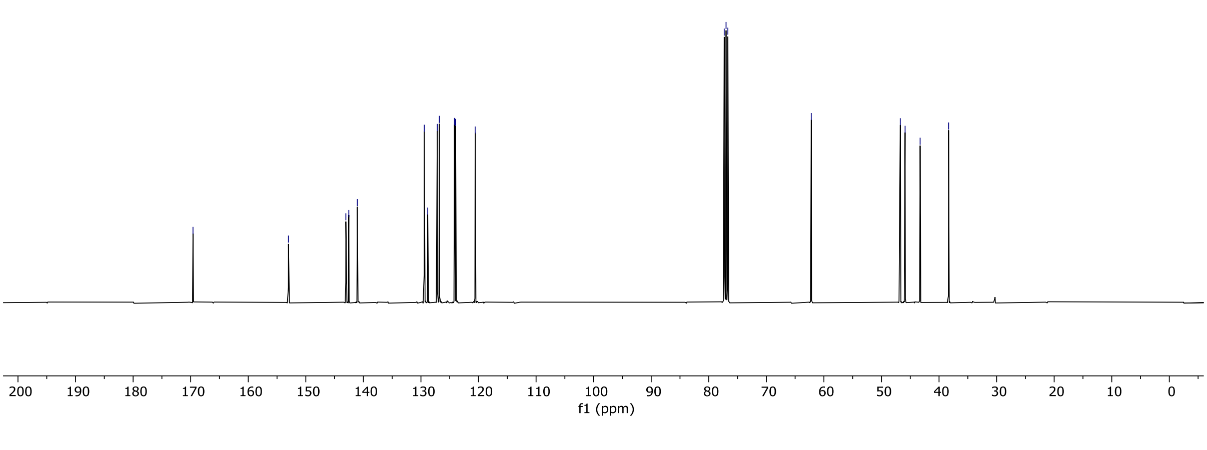

3-((4a*R*,9a*R*)-7-Bromo-1-chloro-4a,9a-dihydro-9*H*-fluorene-4-carbonyl)oxazolidin-2-one, 2b

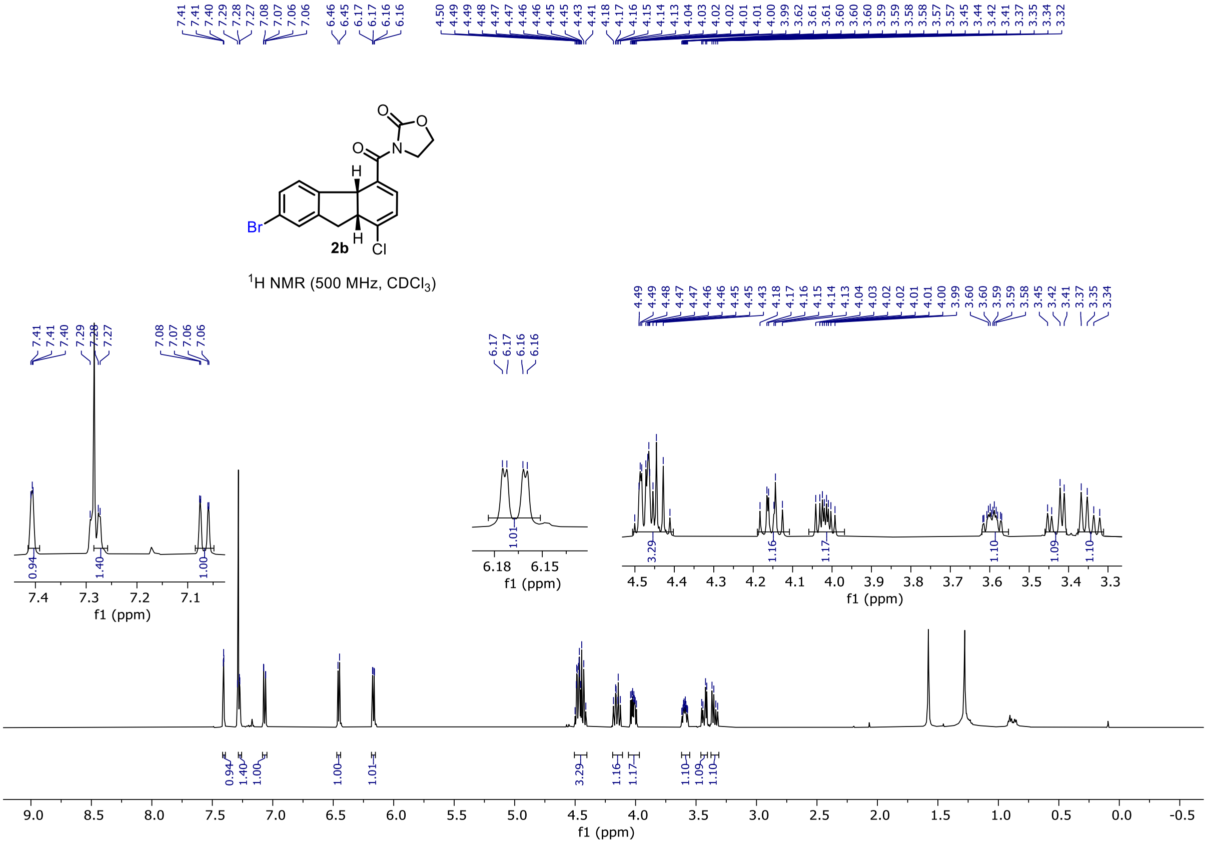

^{1}H NMR (500 MHz, $CDCl_3$)

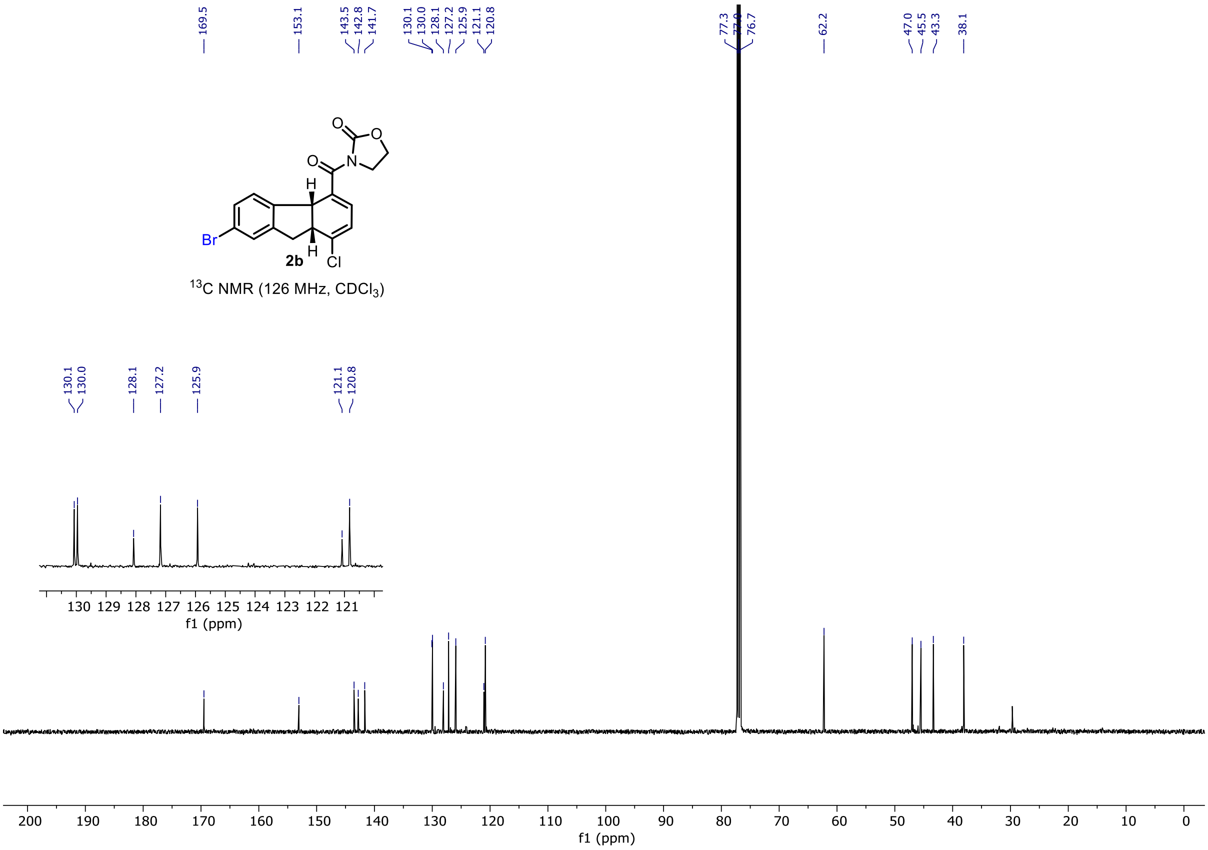

^{13}C NMR (126 MHz, $CDCl_3$)

3-((4a*R*,9a*R*)-1-Chloro-7-fluoro-4a,9a-dihydro-9*H*-fluorene-4-carbonyl)oxazolidin-2-one, 2c

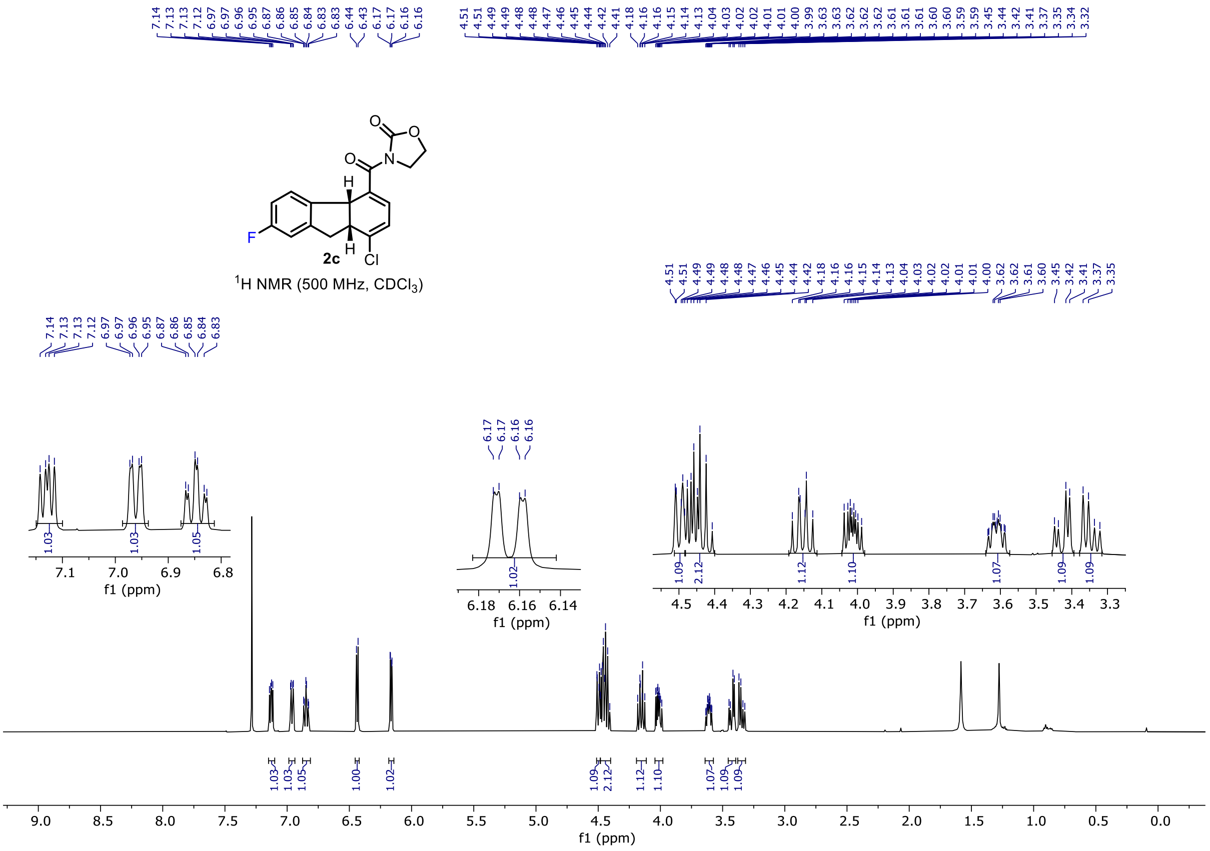

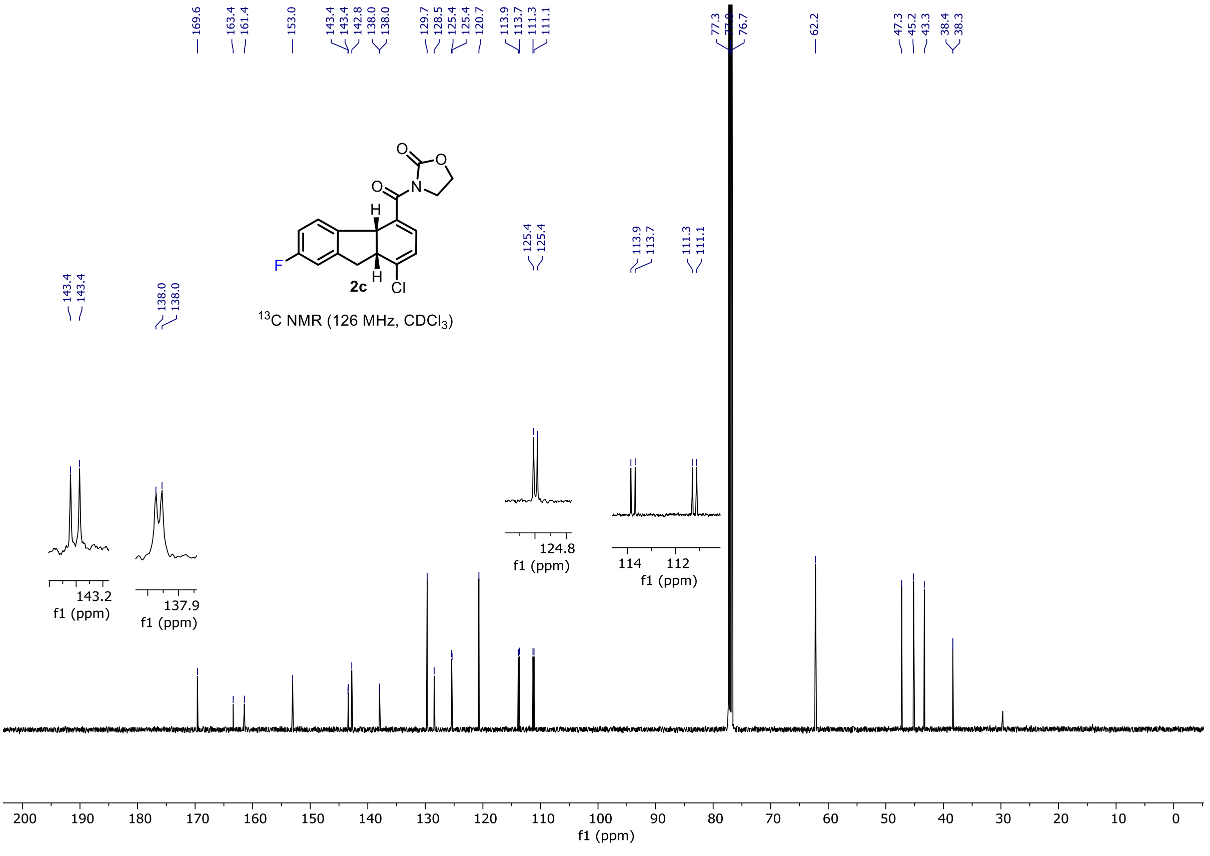

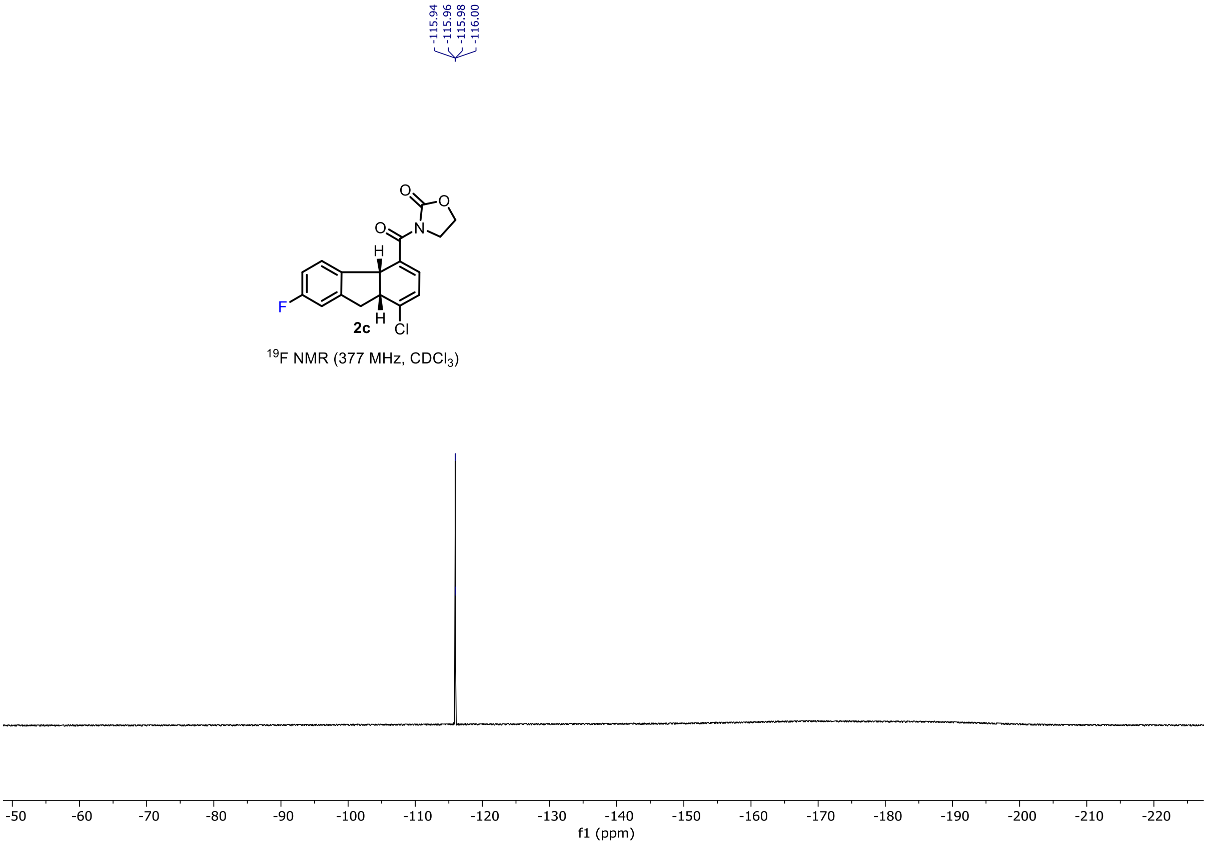
-115.94
-115.96
-115.98
-116.00
2c
^{19}F NMR (377 MHz, CDCl$_3$)
f1 (ppm)

3-((4a*R*,9a*R*)-8-Bromo-1-chloro-4a,9a-dihydro-9*H*-fluorene-4-carbonyl)oxazolidin-2-one, 2d

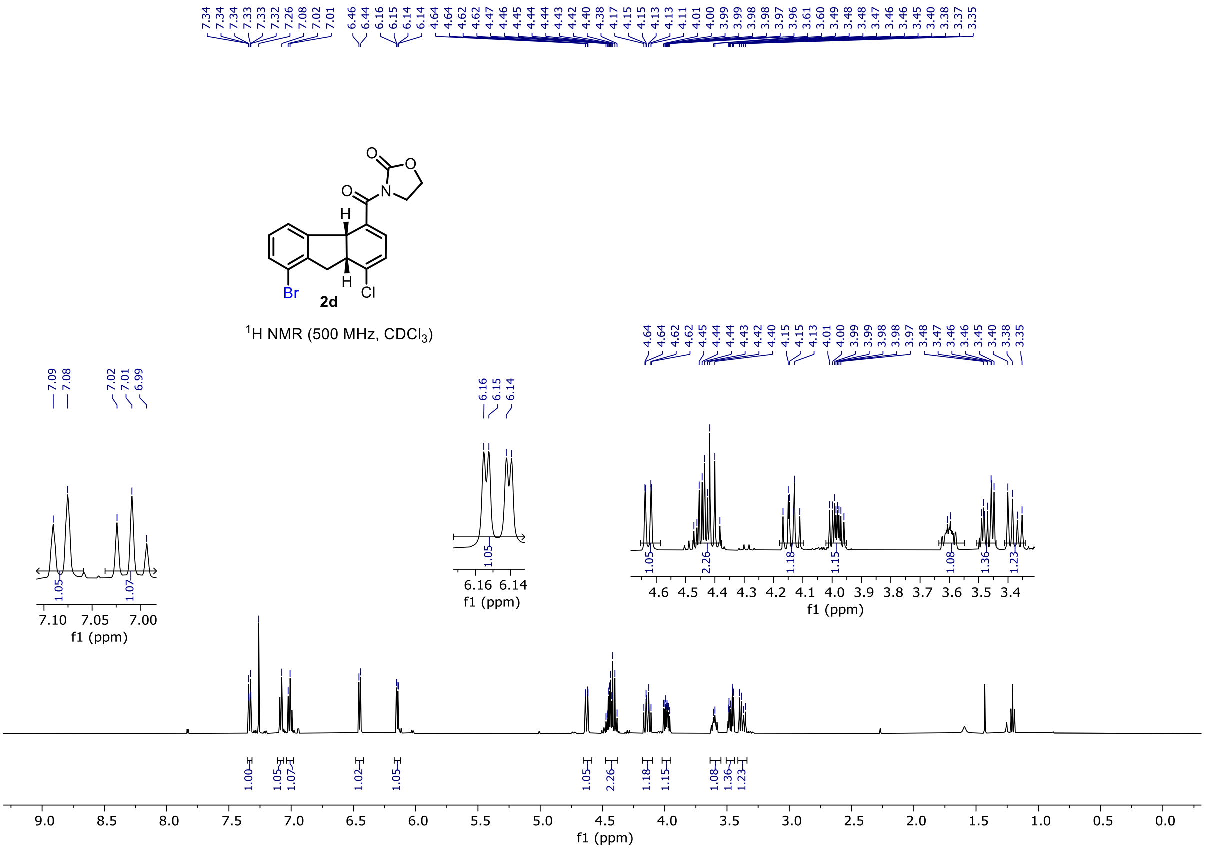

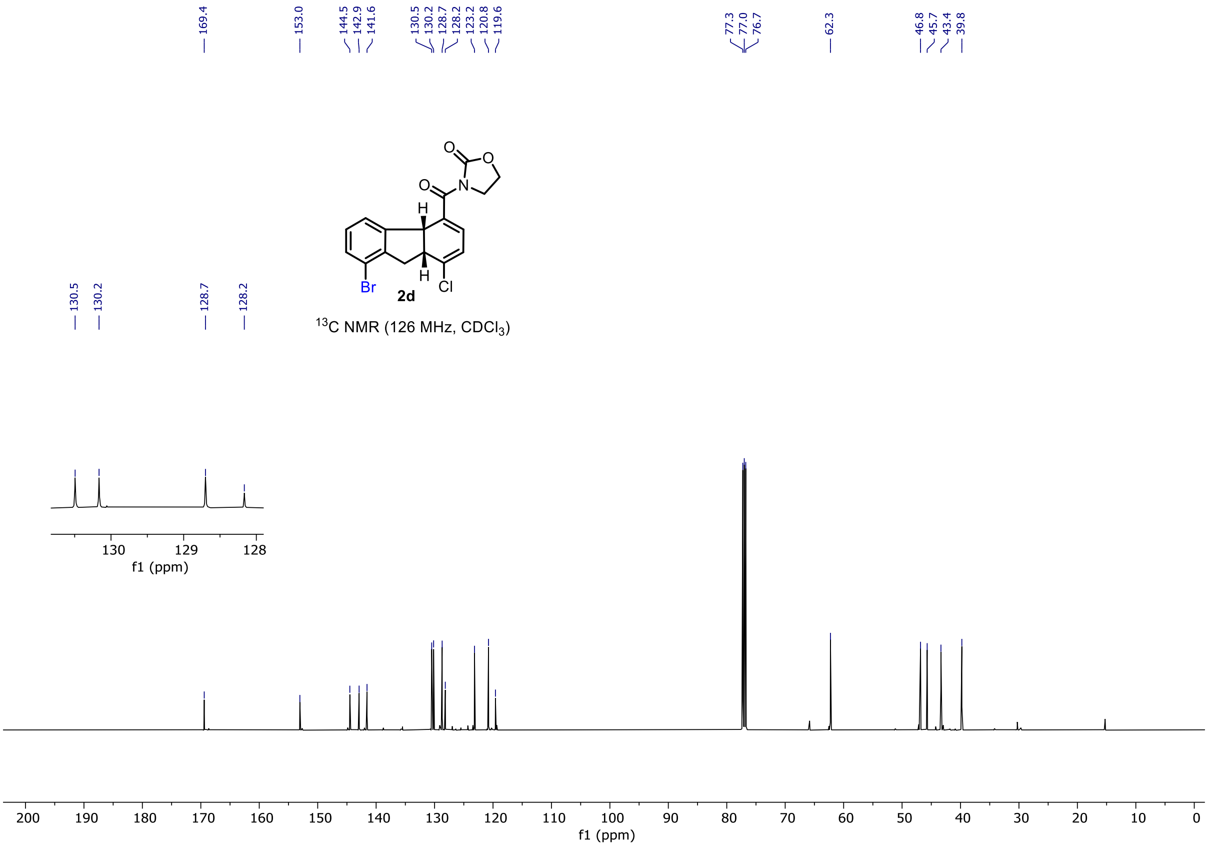

(4b*R*,8a*R*)-8-Chloro-5-(2-oxooxazolidine-3-carbonyl)-4b,8a-dihydro-9*H*-fluorene-2-carbonitrile, 2e

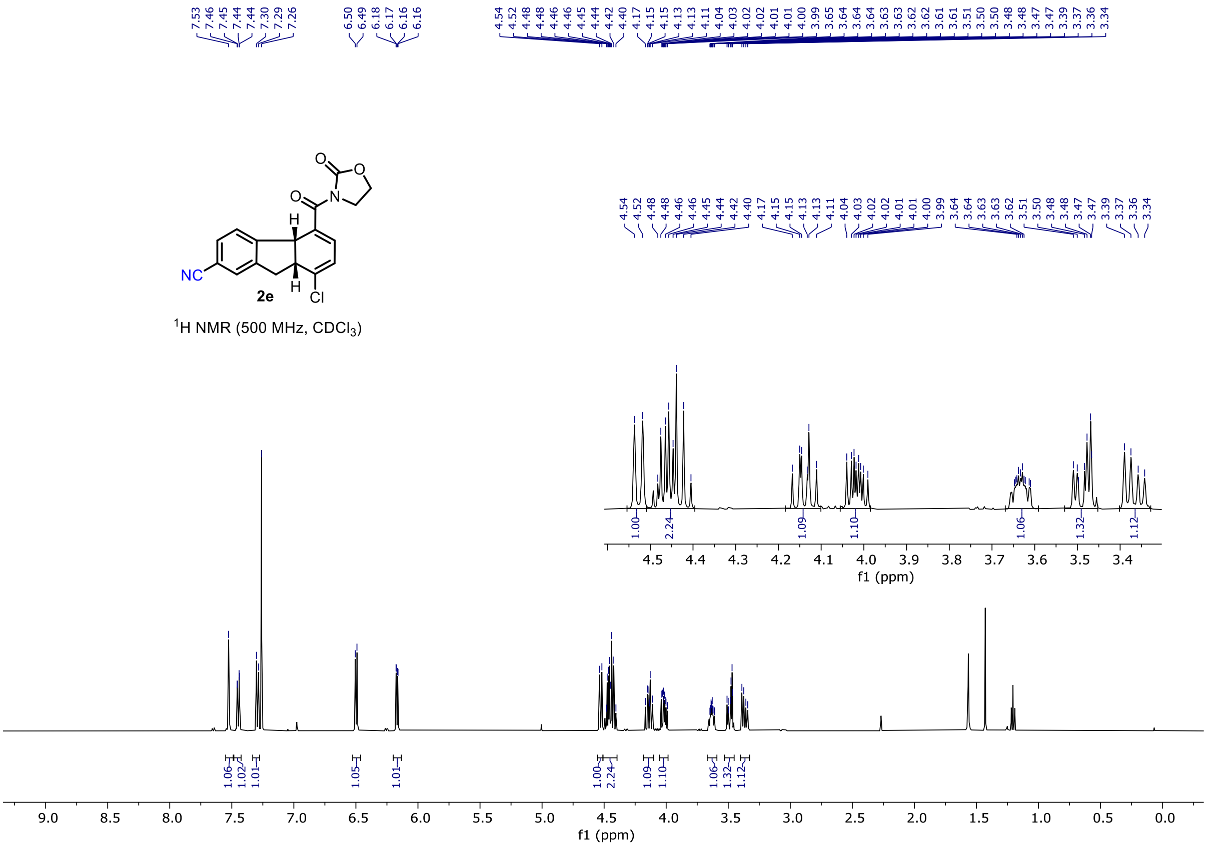

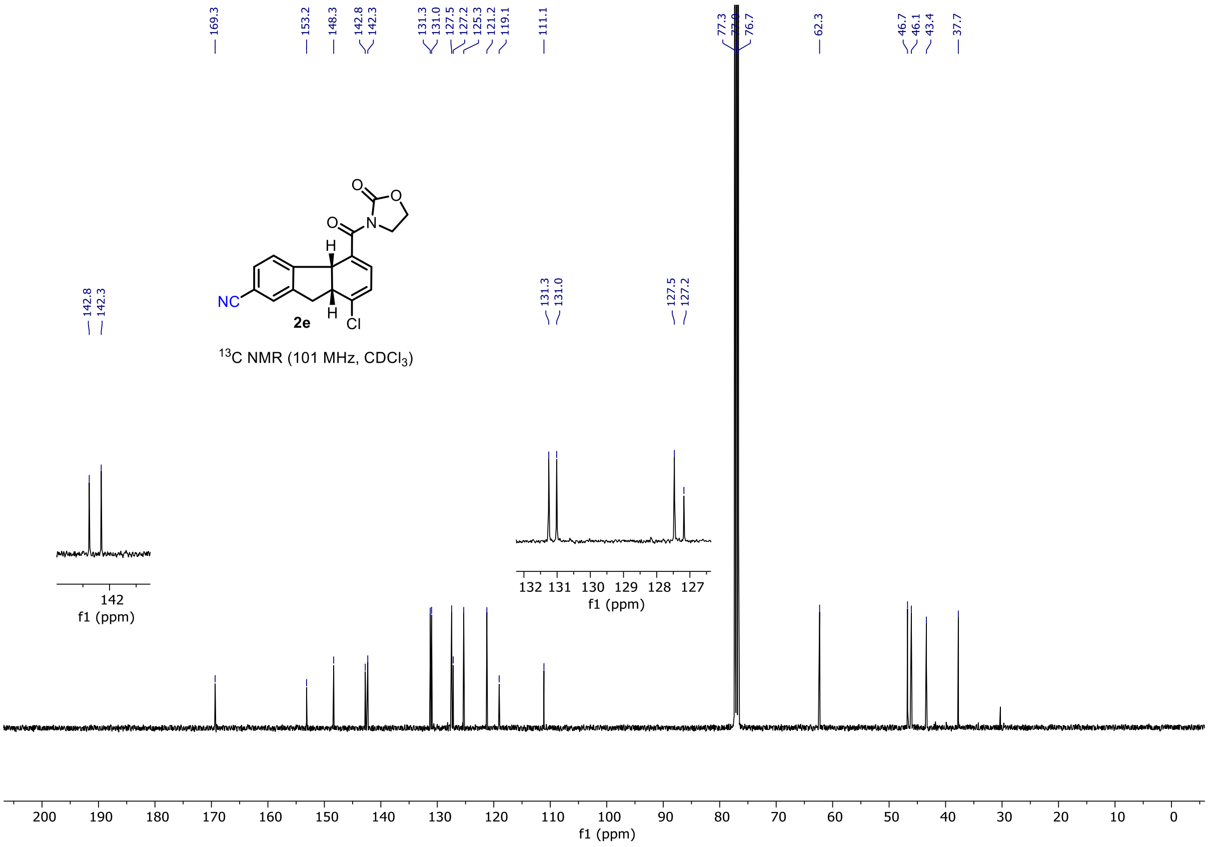

3-((4a*R*,9a*R*)-1-Chloro-7-(trifluoromethyl)-4a,9a-dihydro-9*H*-fluorene-4-carbonyl) oxazolidin-2-one, 2f

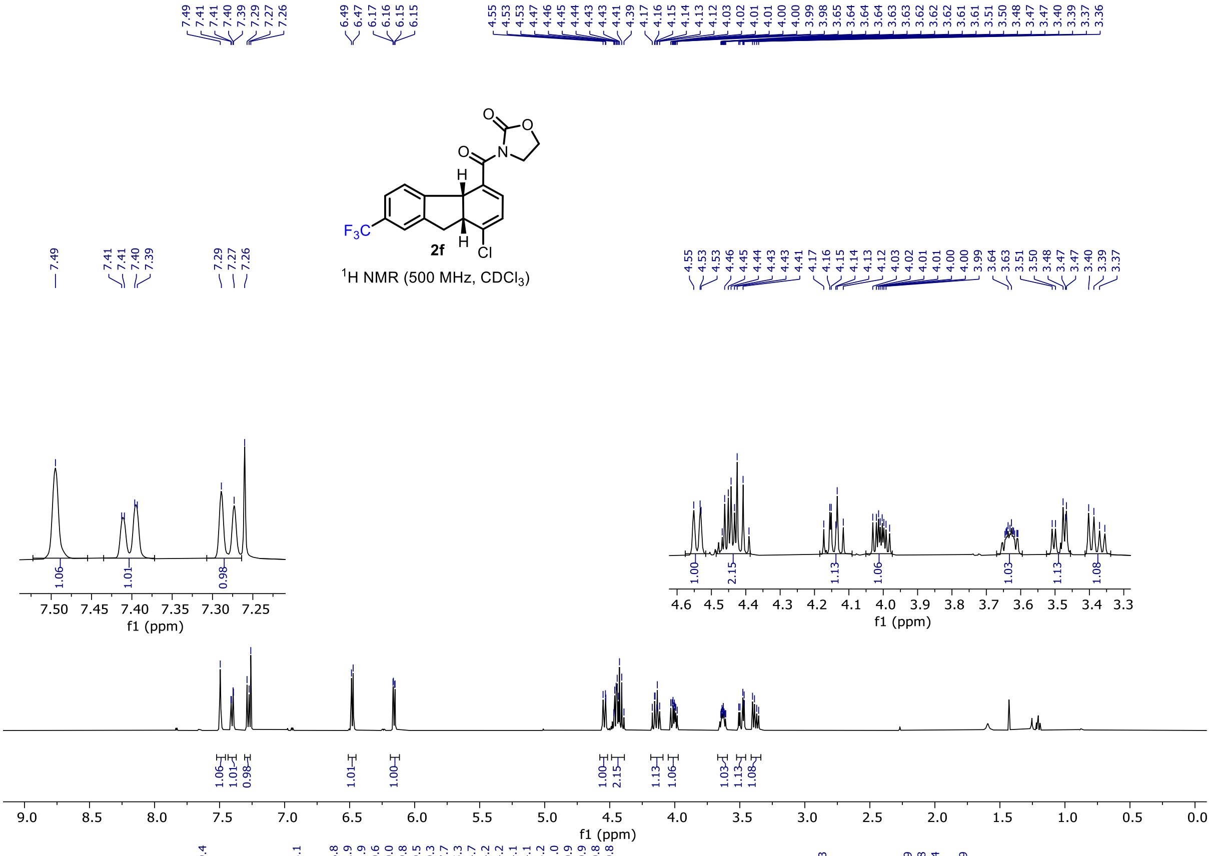

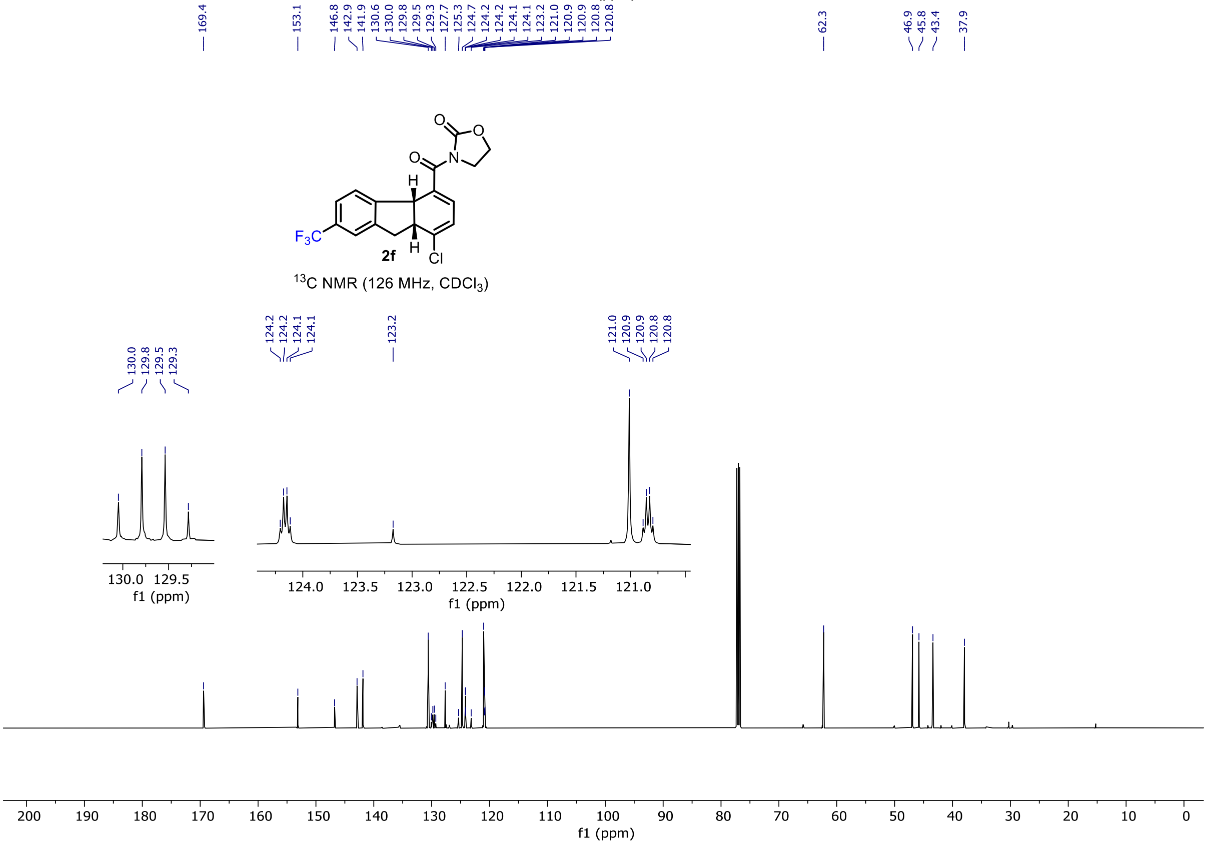

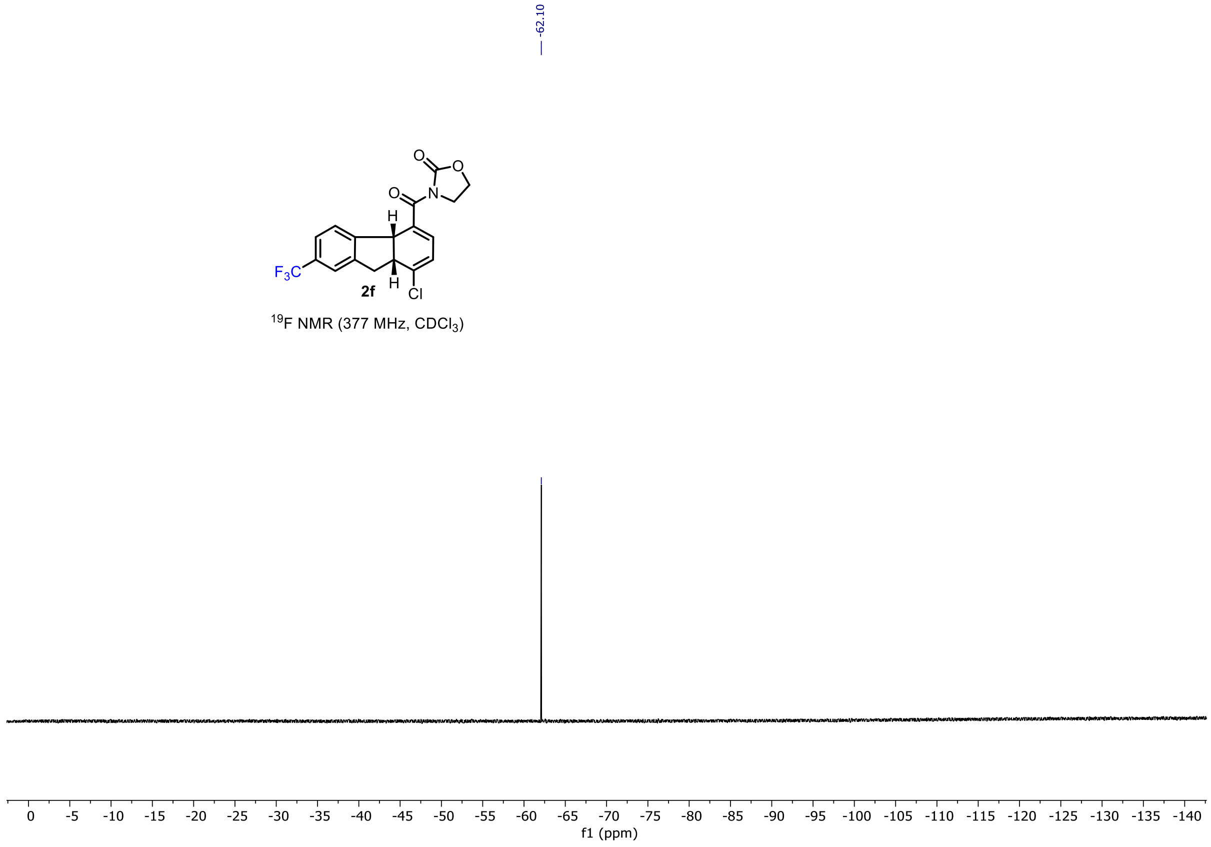
-62.10
2f
F3C
Cl
^{19}F NMR (377 MHz, CDCl$_3$)
0 -5 -10 -15 -20 -25 -30 -35 -40 -45 -50 -55 -60 -65 -70 -75 -80 -85 -90 -95 -100 -105 -110 -115 -120 -125 -130 -135 -140
f1 (ppm)

Methyl (4b*R*,8a*R*)-8-chloro-5-(2-oxooxazolidine-3-carbonyl)-4b,8a-dihydro-9*H*-fluorene-2-carboxylate, 2g

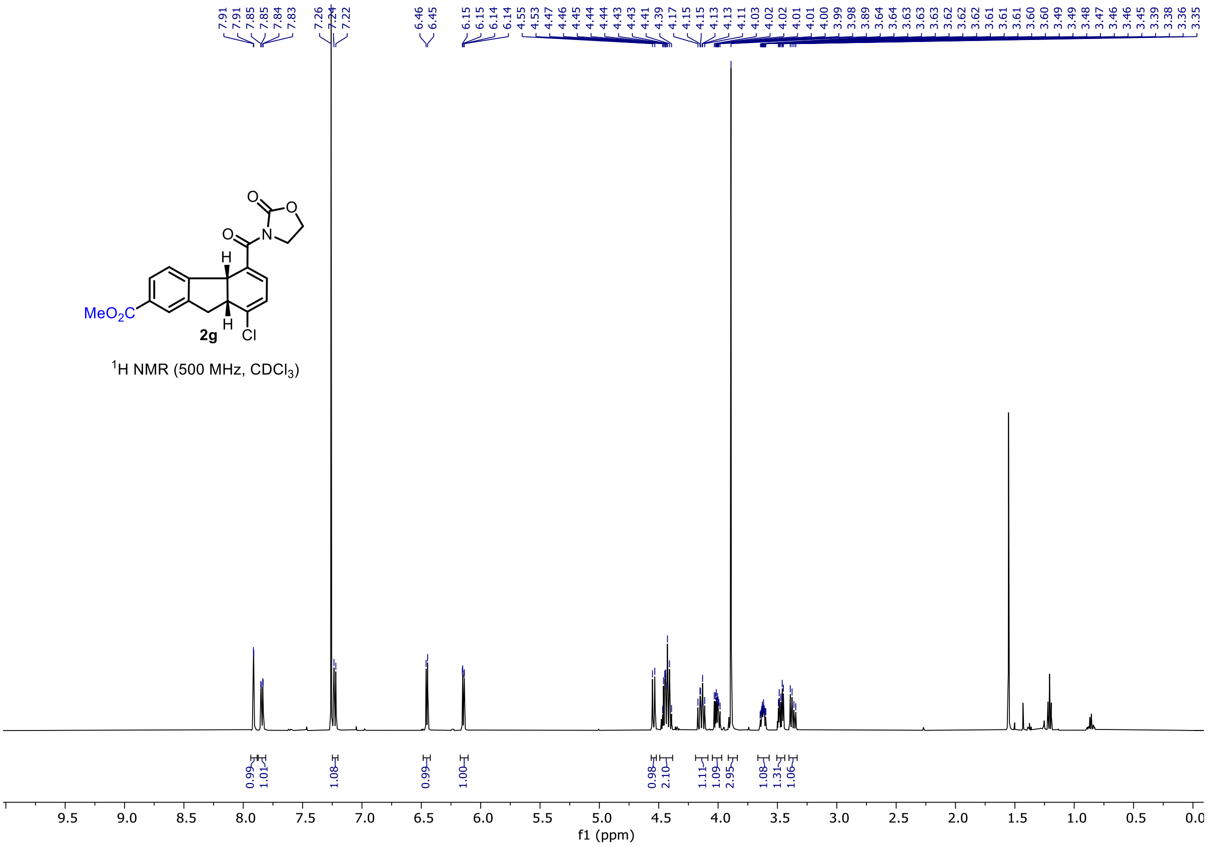

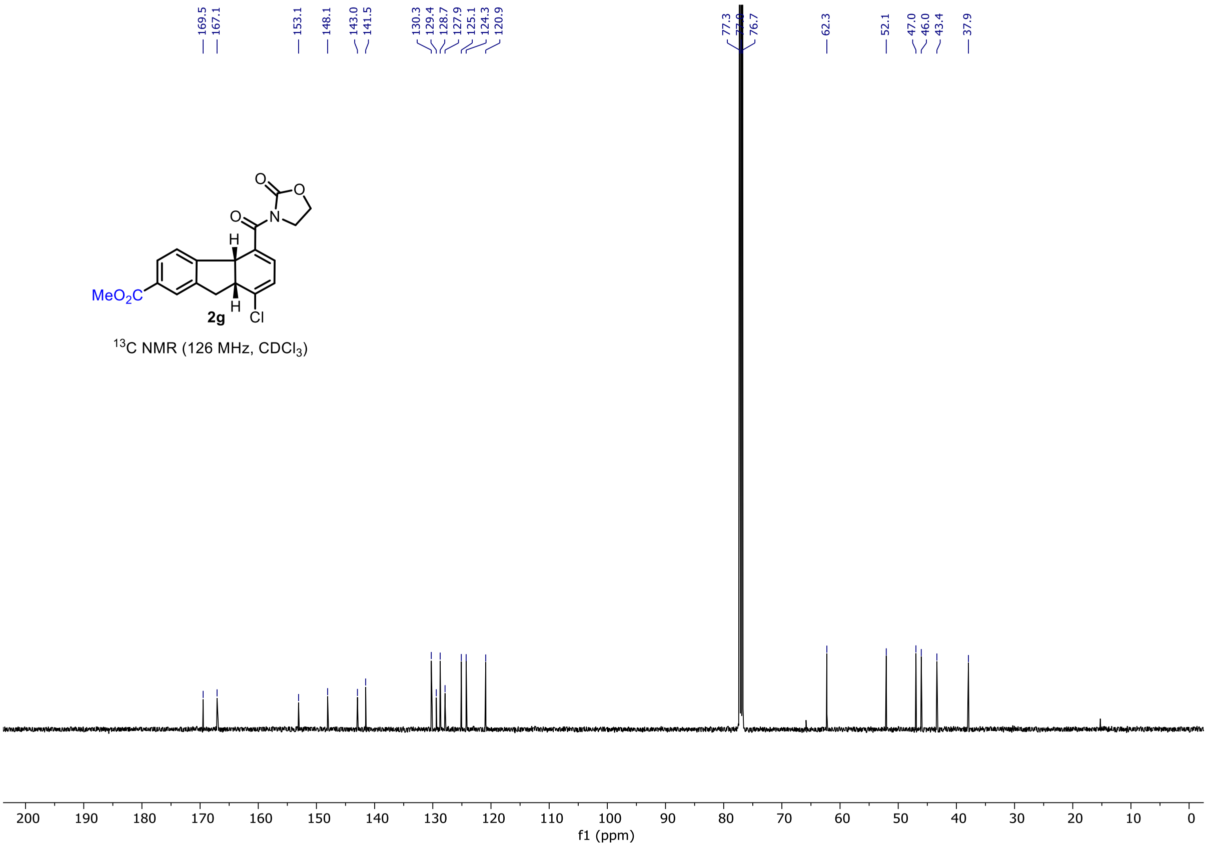

3-((4a*R*,9a*R*)-1-Chloro-6-nitro-4a,9a-dihydro-9*H*-fluorene-4-carbonyl)oxazolidin-2-one, 2h

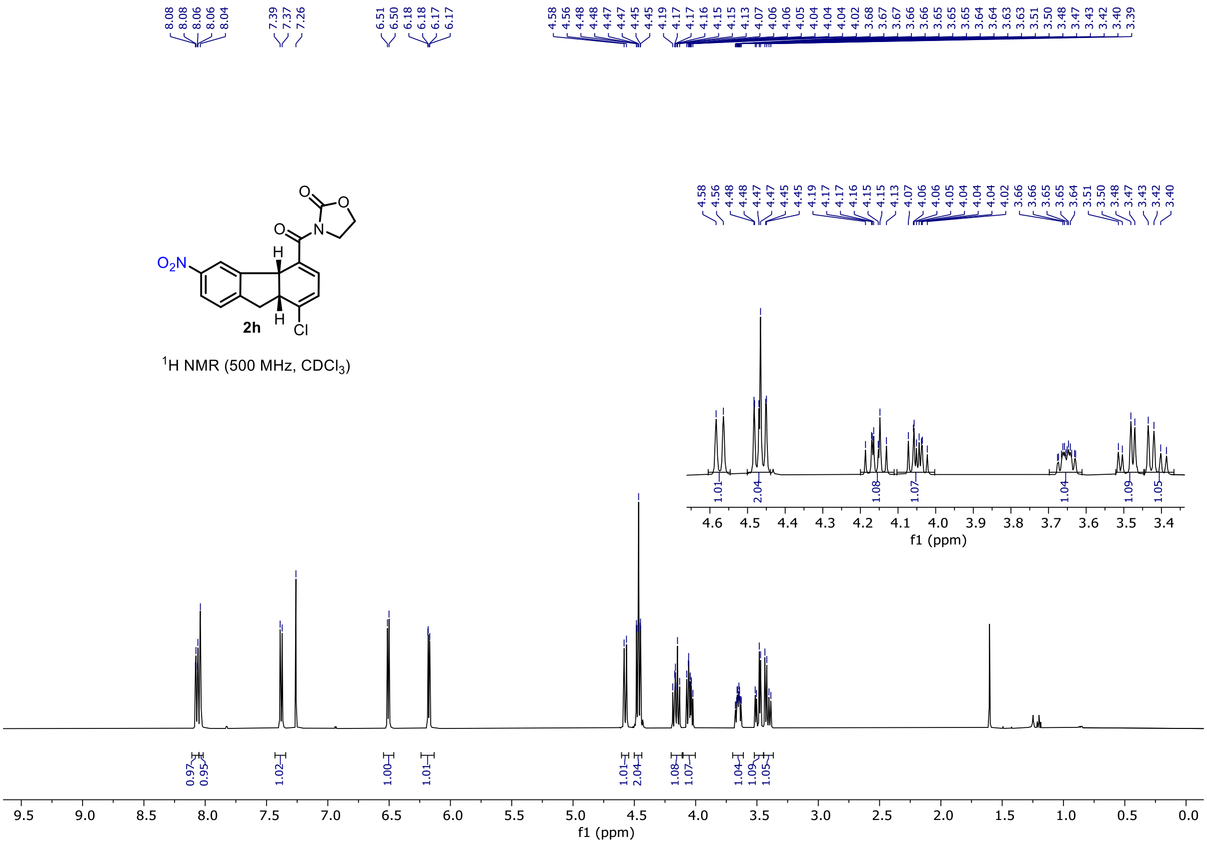

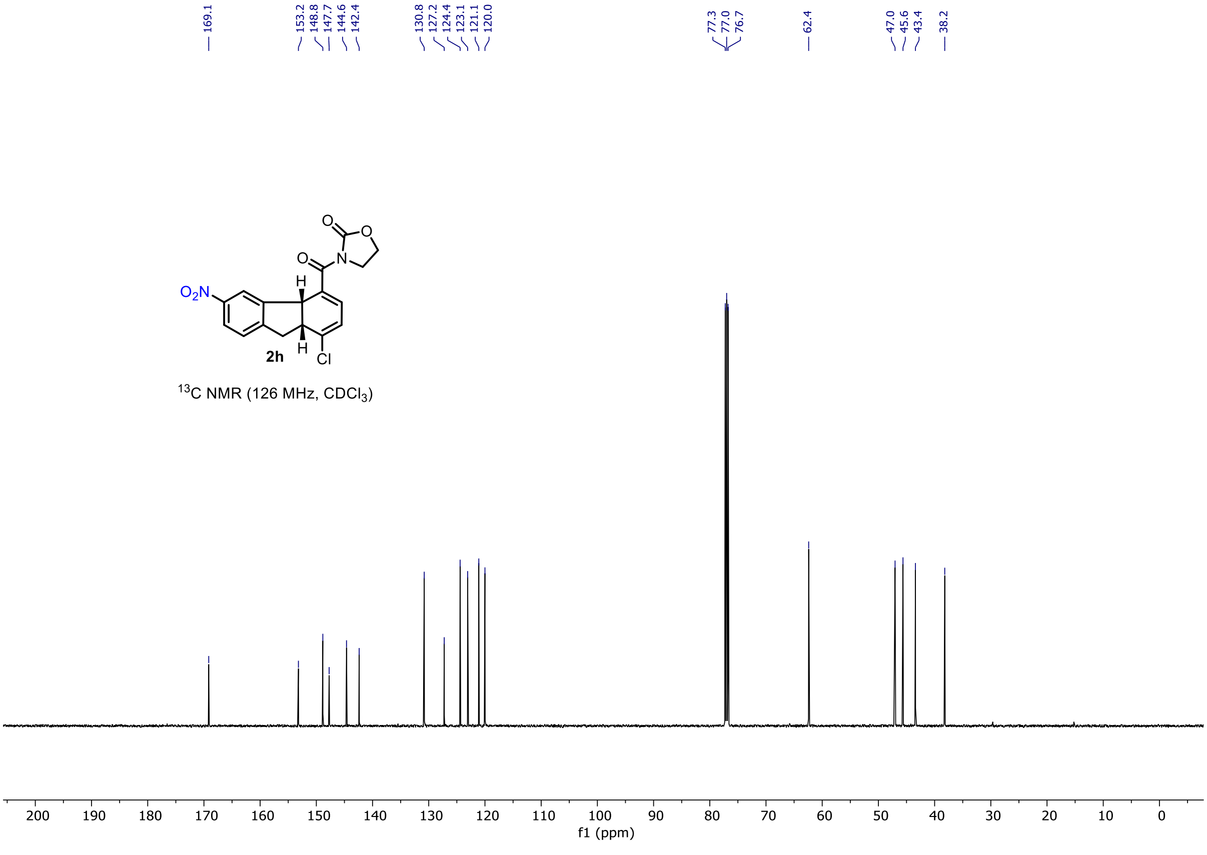

3-((4a*R*,9a*R*)-1-Chloro-7-(4-(trifluoromethyl)phenyl)-4a,9a-dihydro-9*H*-fluorene-4-carbonyl)oxazolidin-2-one, 2i

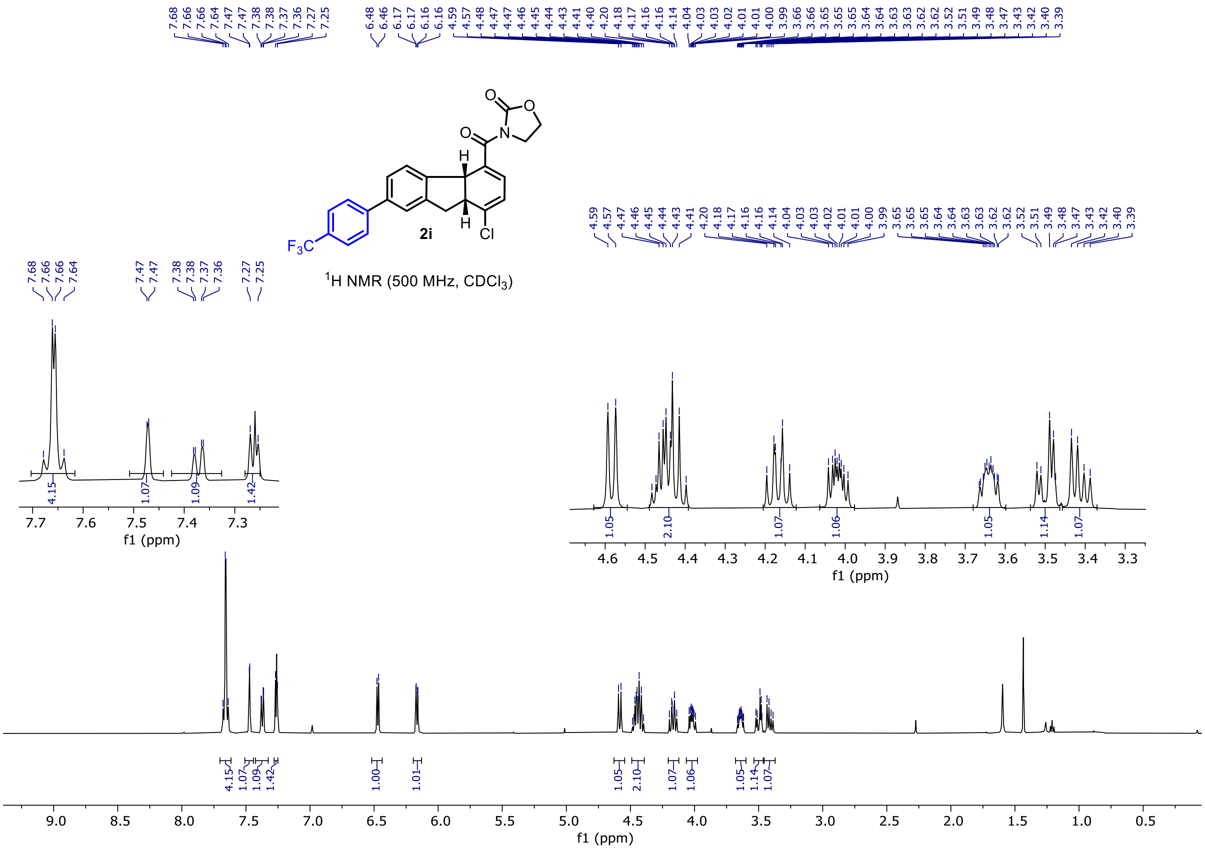

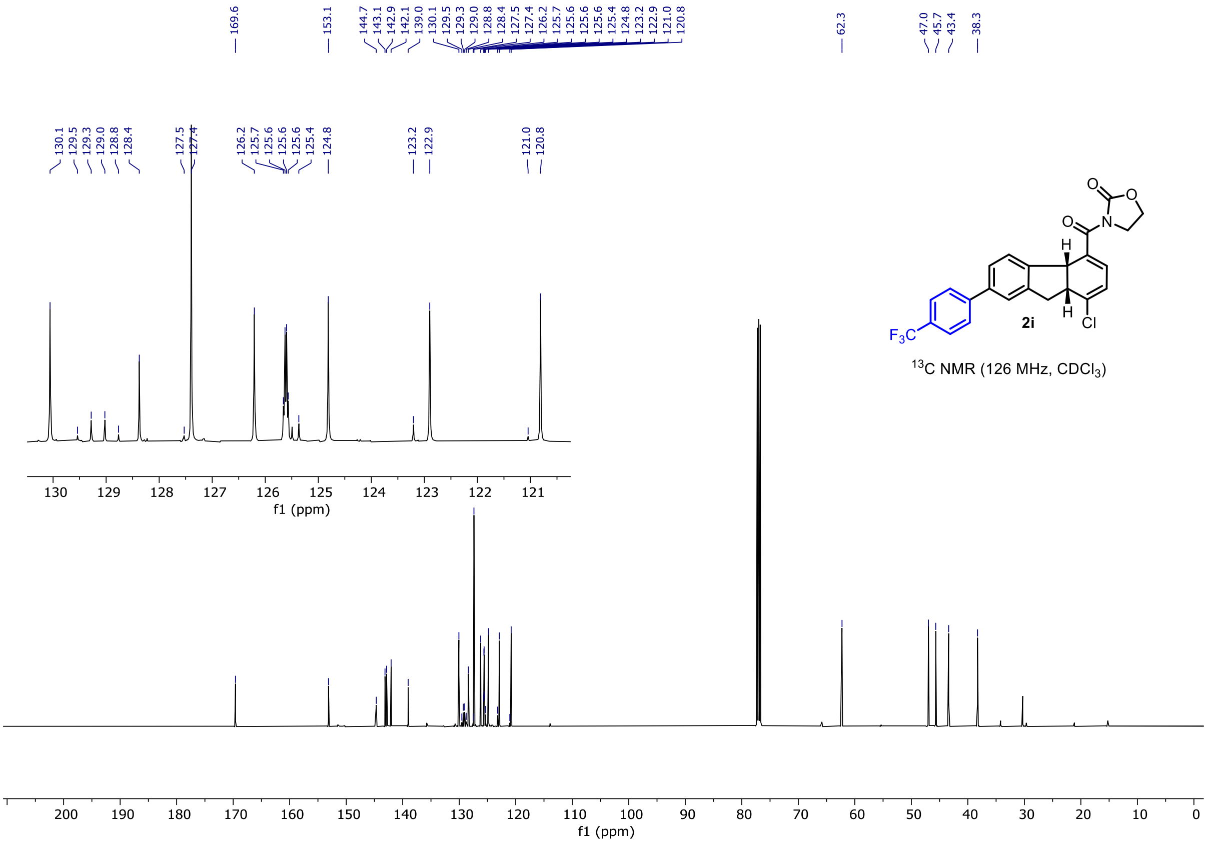

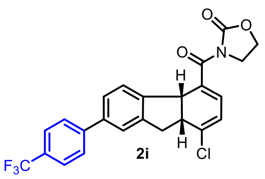

¹⁹F NMR (471 MHz, $CDCl_3$)

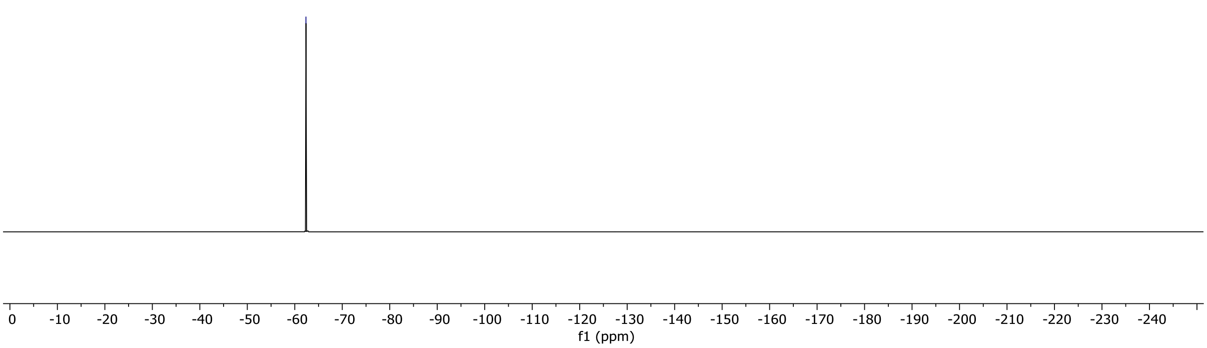

Methyl 4-((4b*R*,8a*R*)-8-chloro-5-(2-oxooxazolidine-3-carbonyl)-4b,8a-dihydro-9*H*-fluoren-2-yl)benzoate, 2j

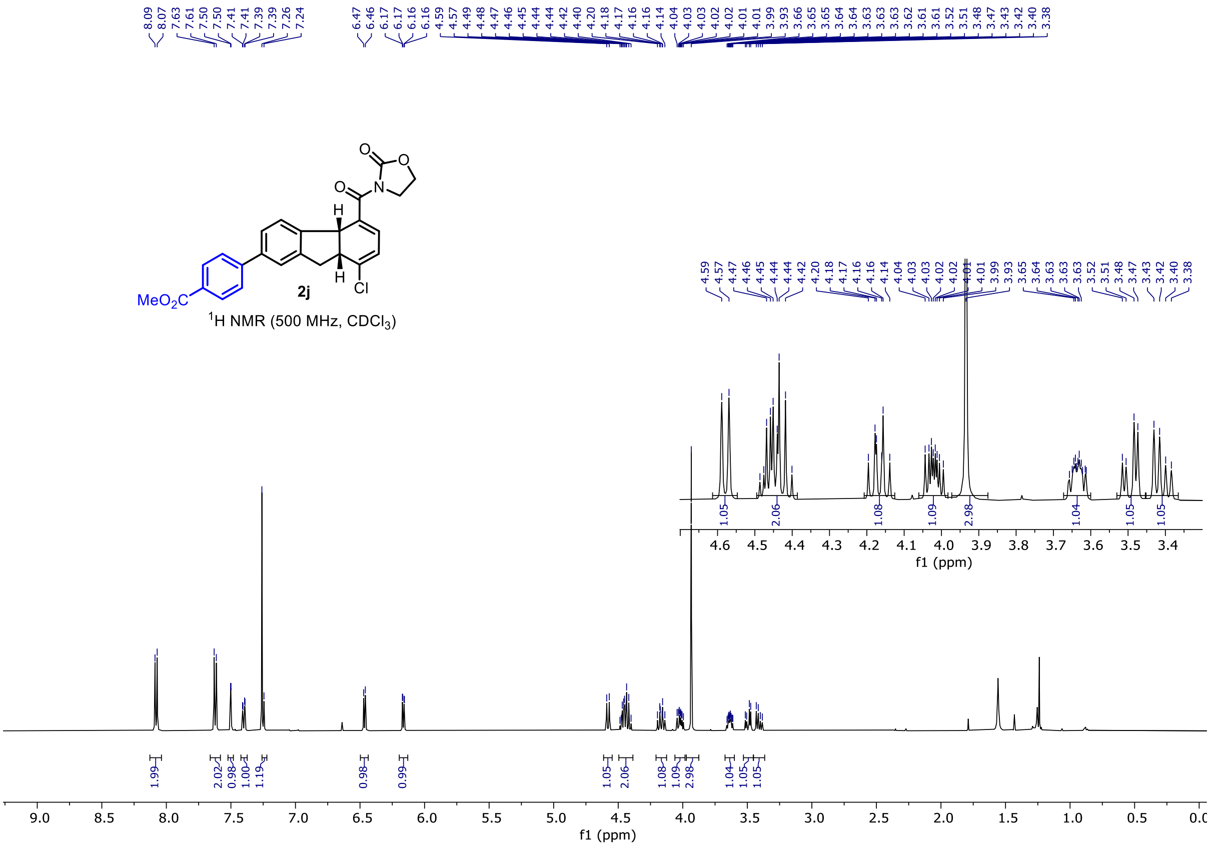

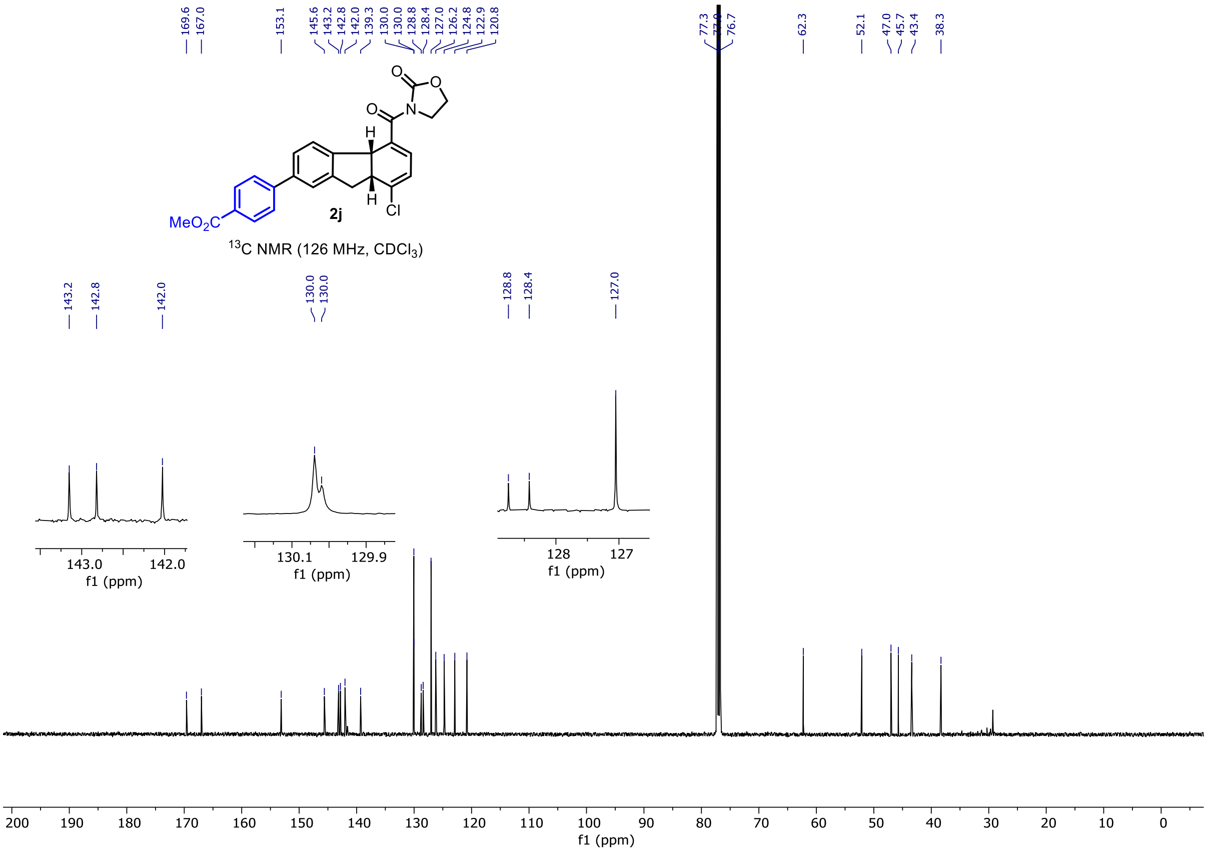

3-((4a*R*,9a*R*)-1-Chloro-6-methyl-4a,9a-dihydro-9*H*-fluorene-4-carbonyl)oxazolidin-2-one, 2k

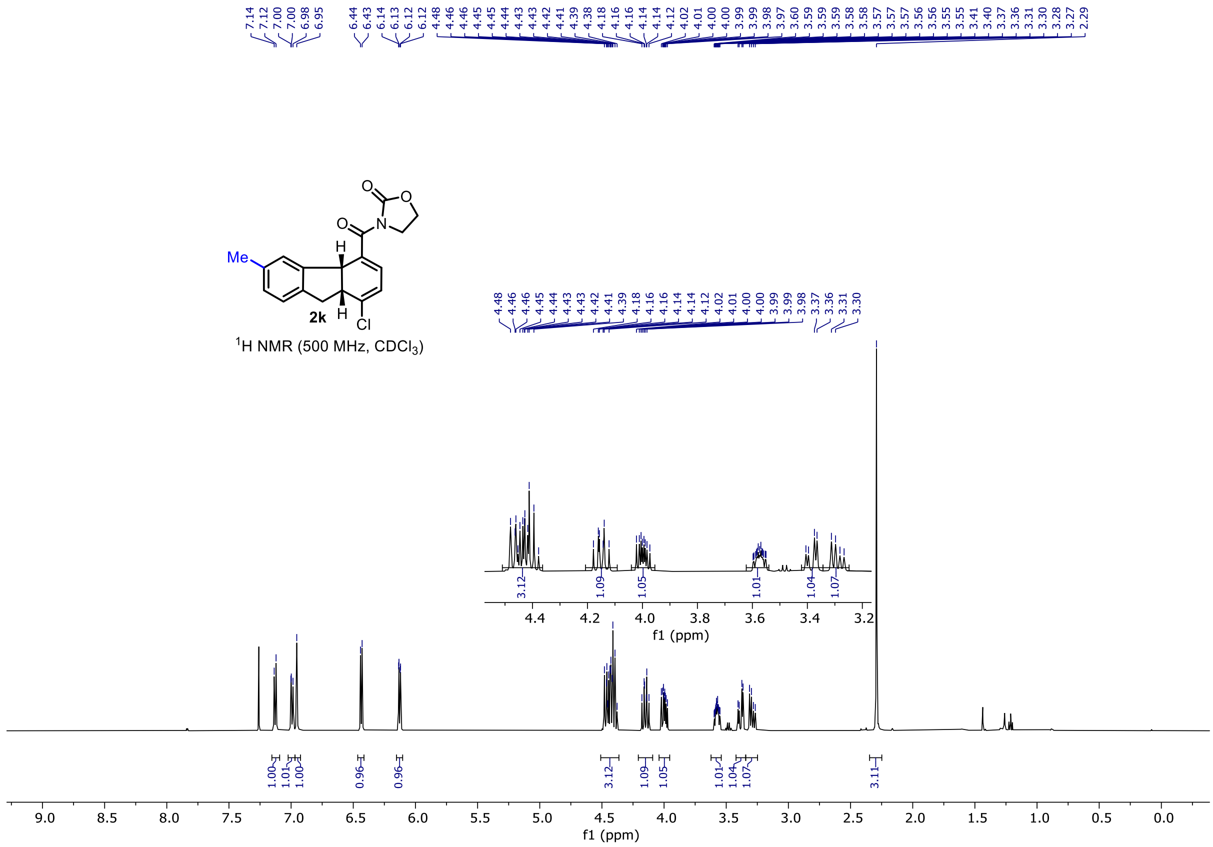

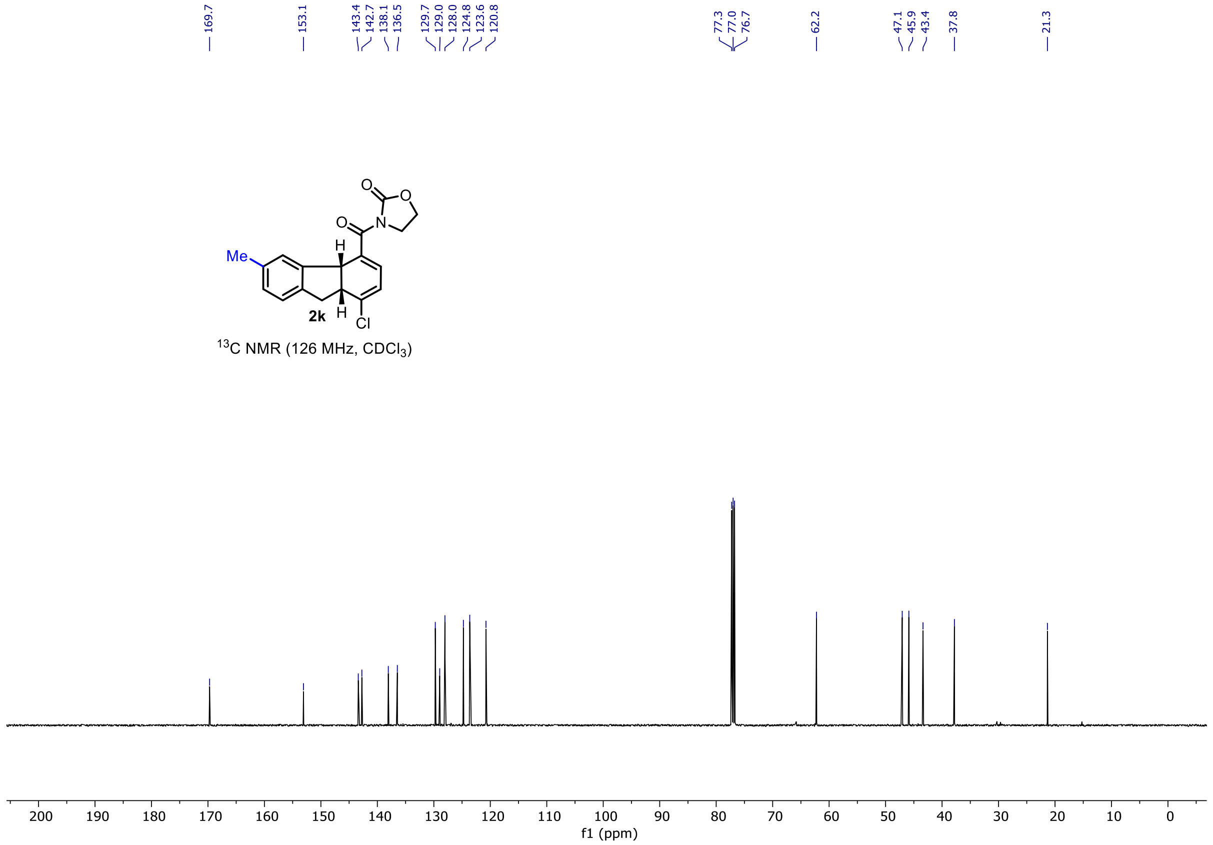

3-((4a*R*,9a*R*)-1-Chloro-8-methoxy-4a,9a-dihydro-9*H*-fluorene-4-carbonyl)oxazolidin-2-one, 2l

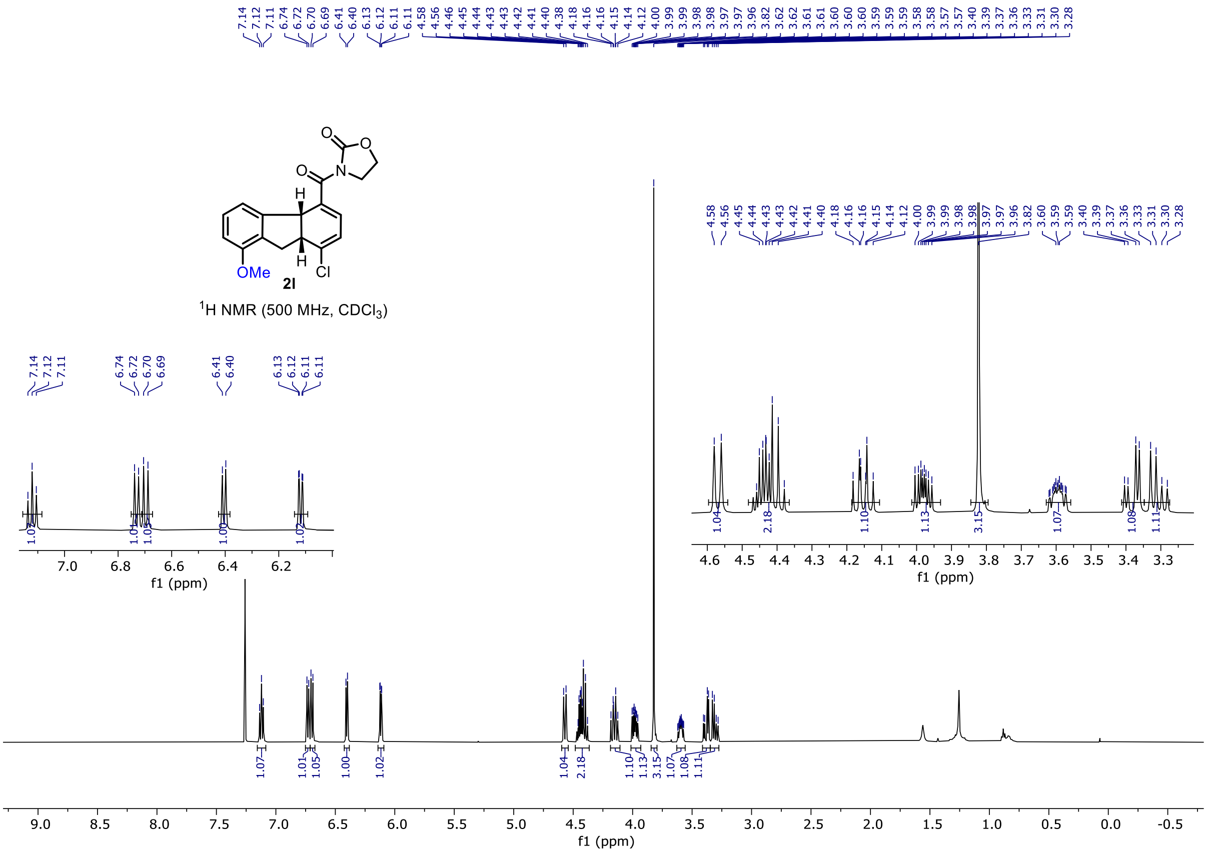

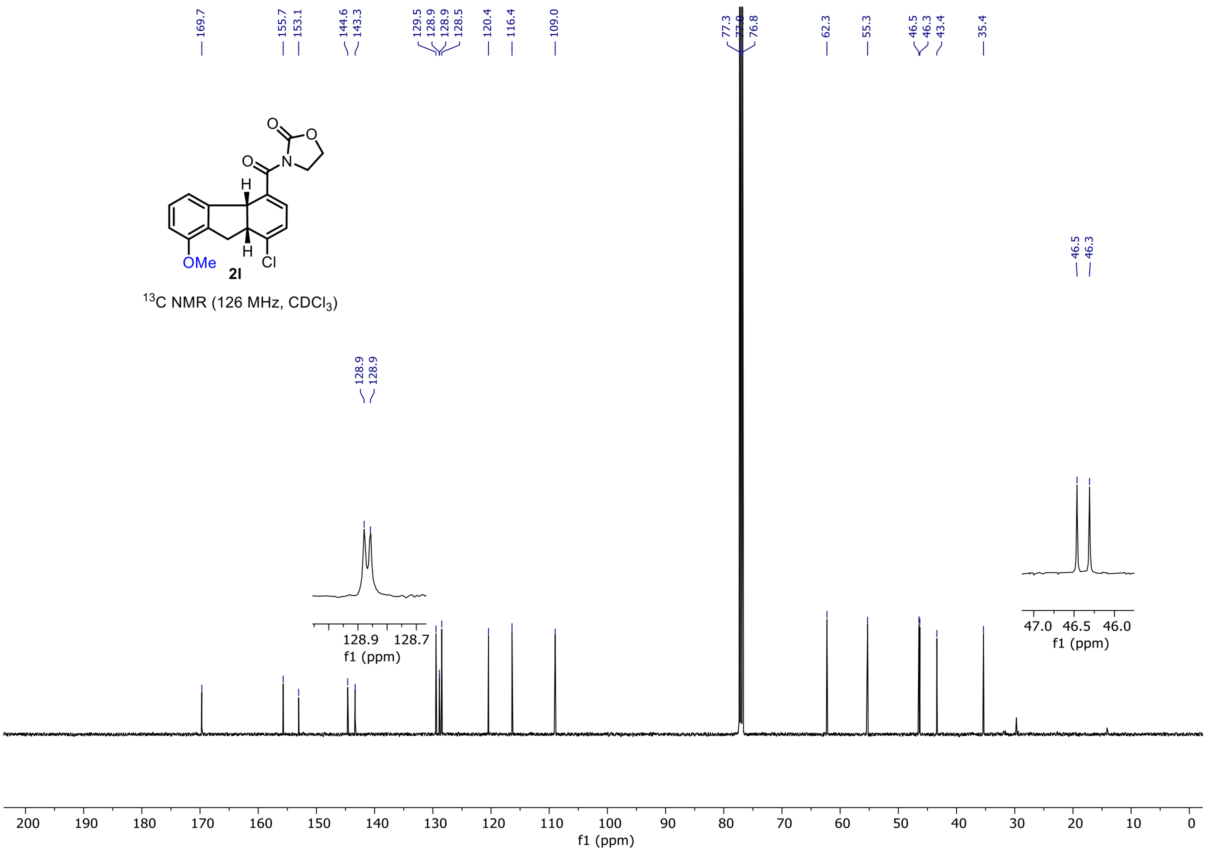

3-((4a*R*,9a*R*)-1-Chloro-8-(4,4,5,5-tetramethyl-1,3,2-dioxaborolan-2-yl)-4a,9a-dihydro-9*H*-fluorene-4-carbonyl)oxazolidin-2-one, 2m

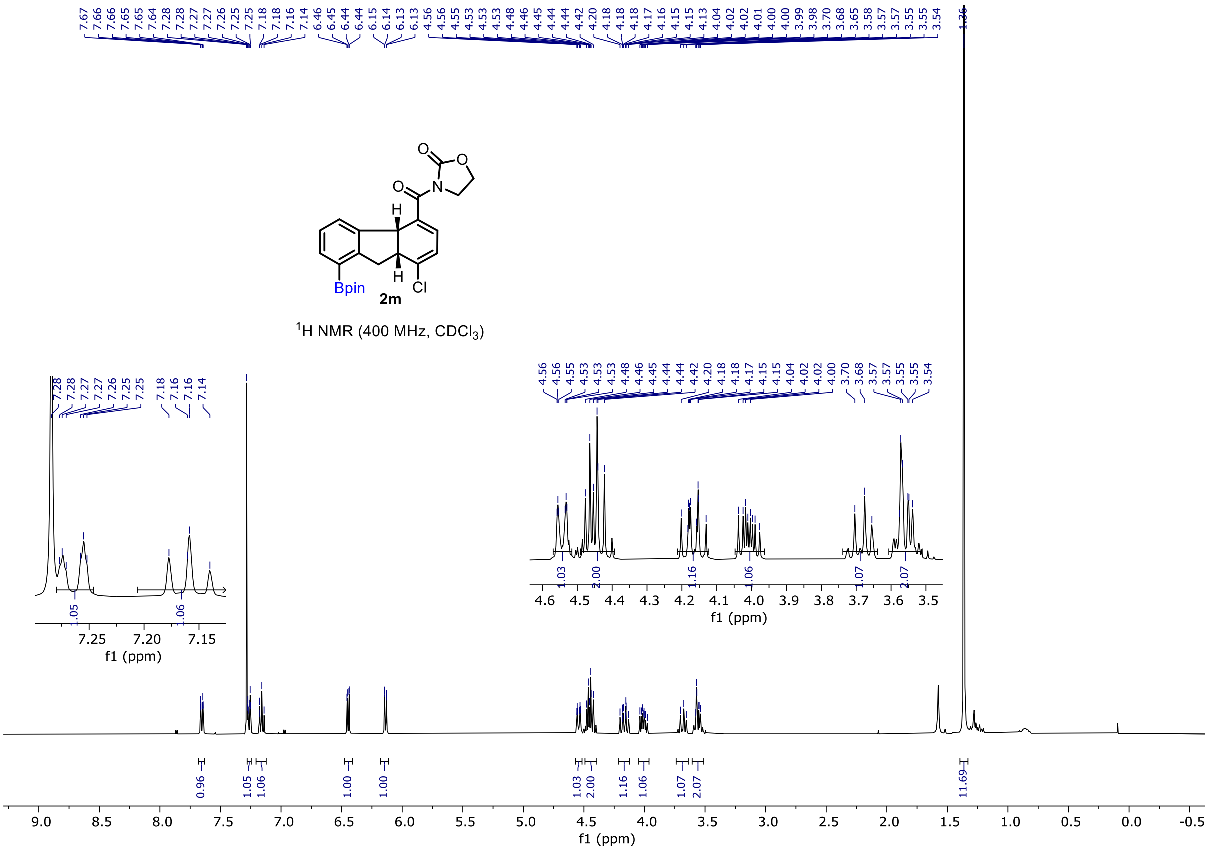

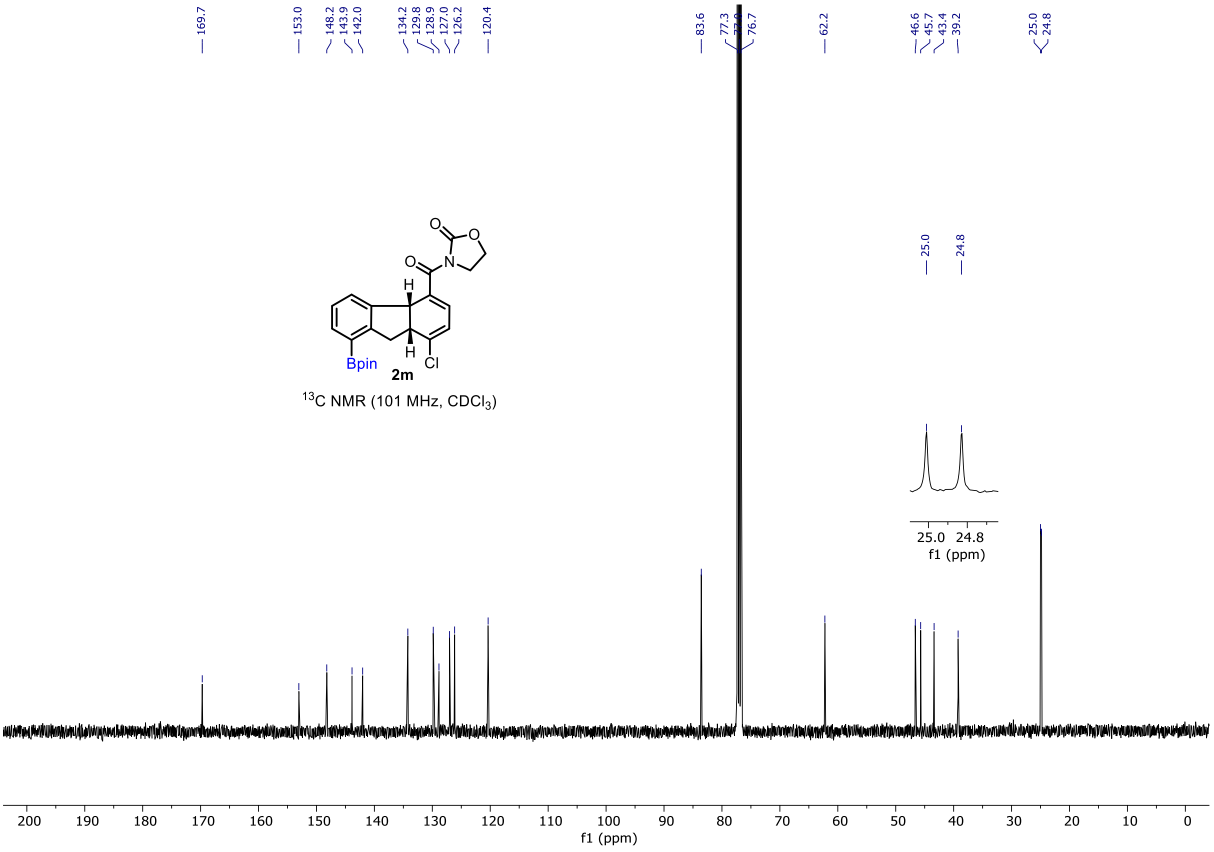

3-((4a*R*,9a*R*)-1-Chloro-6-phenyl-4a,9a-dihydro-9*H*-fluorene-4-carbonyl)oxazolidin-2-one, 2n

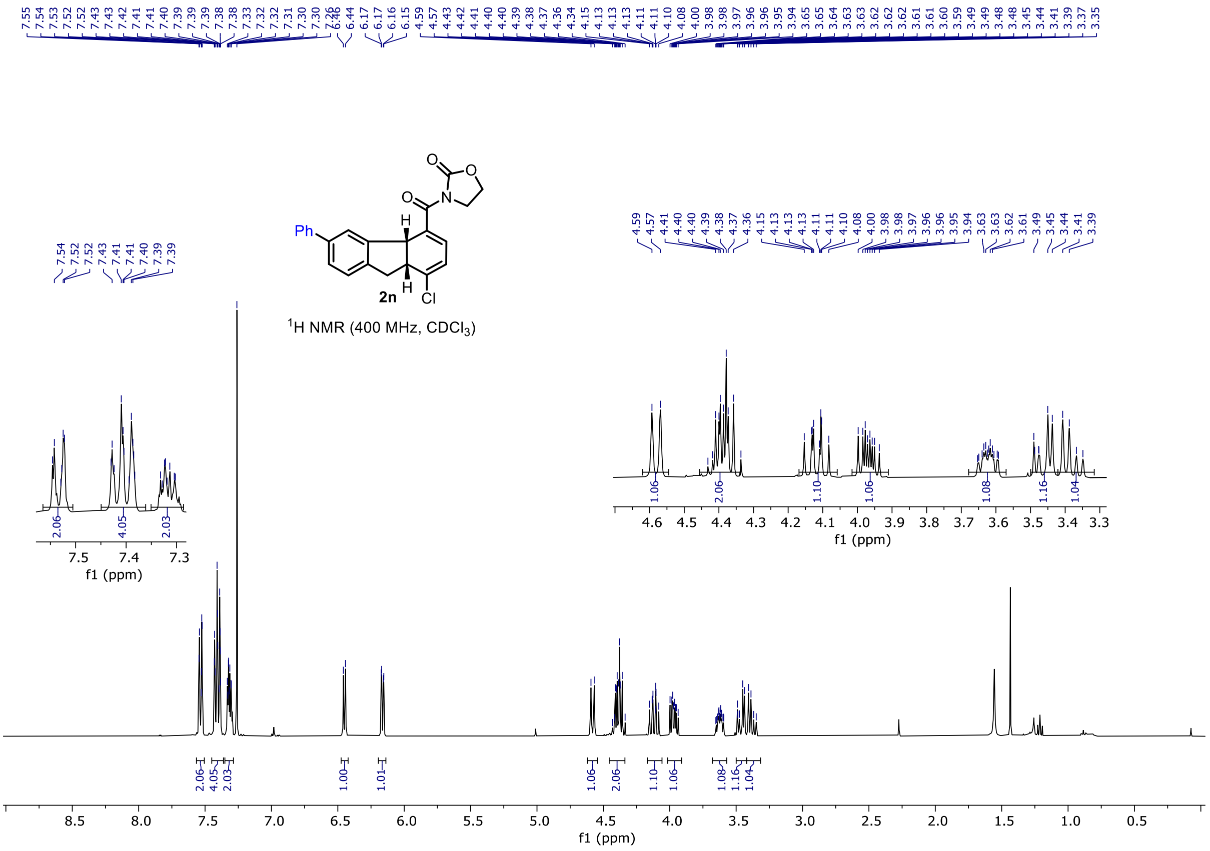

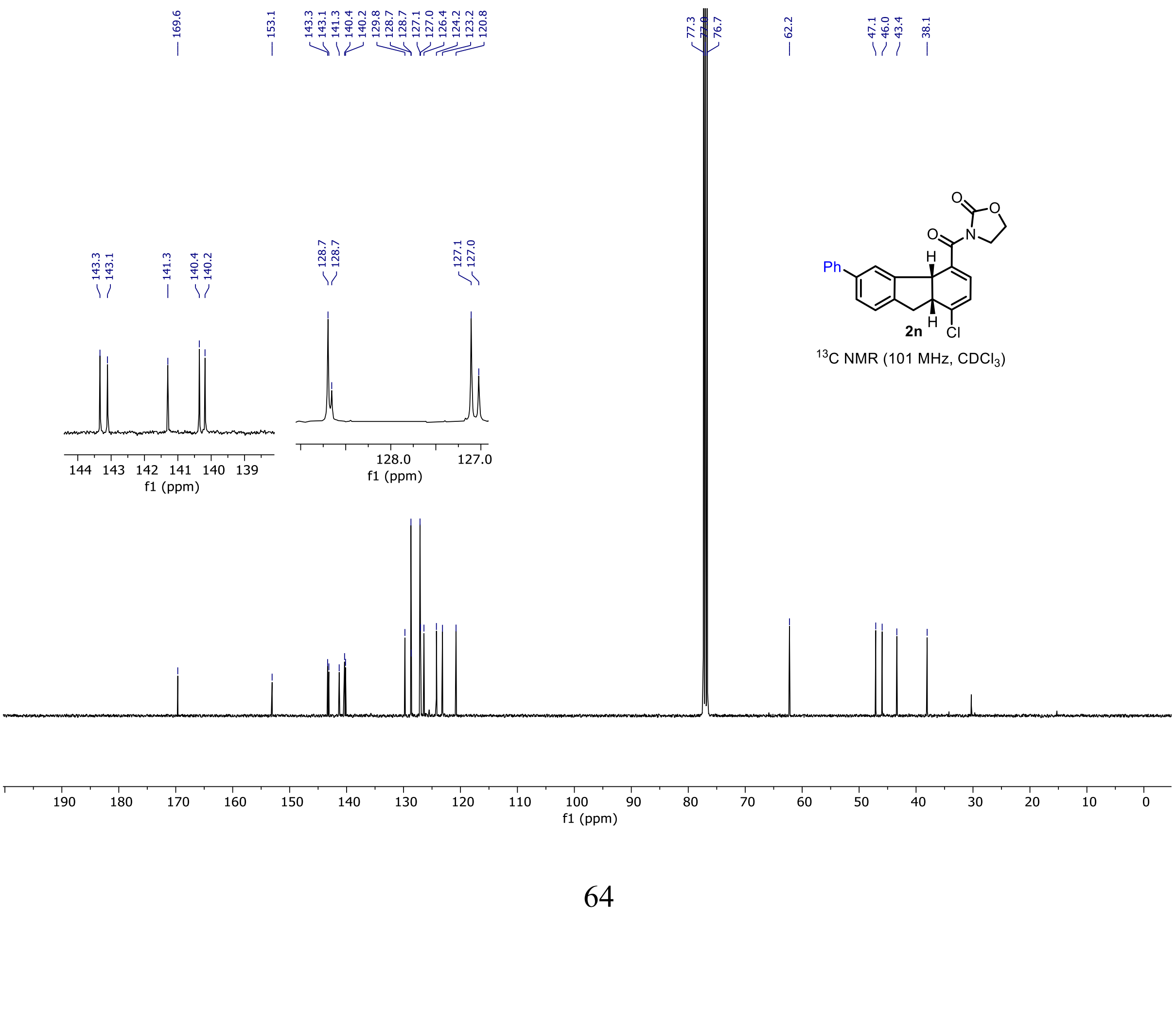

3-((7a*R*,11a*R*)-8-Chloro-7a,11a-dihydro-7*H*-benzo[*c*]fluorene-11-carbonyl)oxazolidin-2-one, 2o

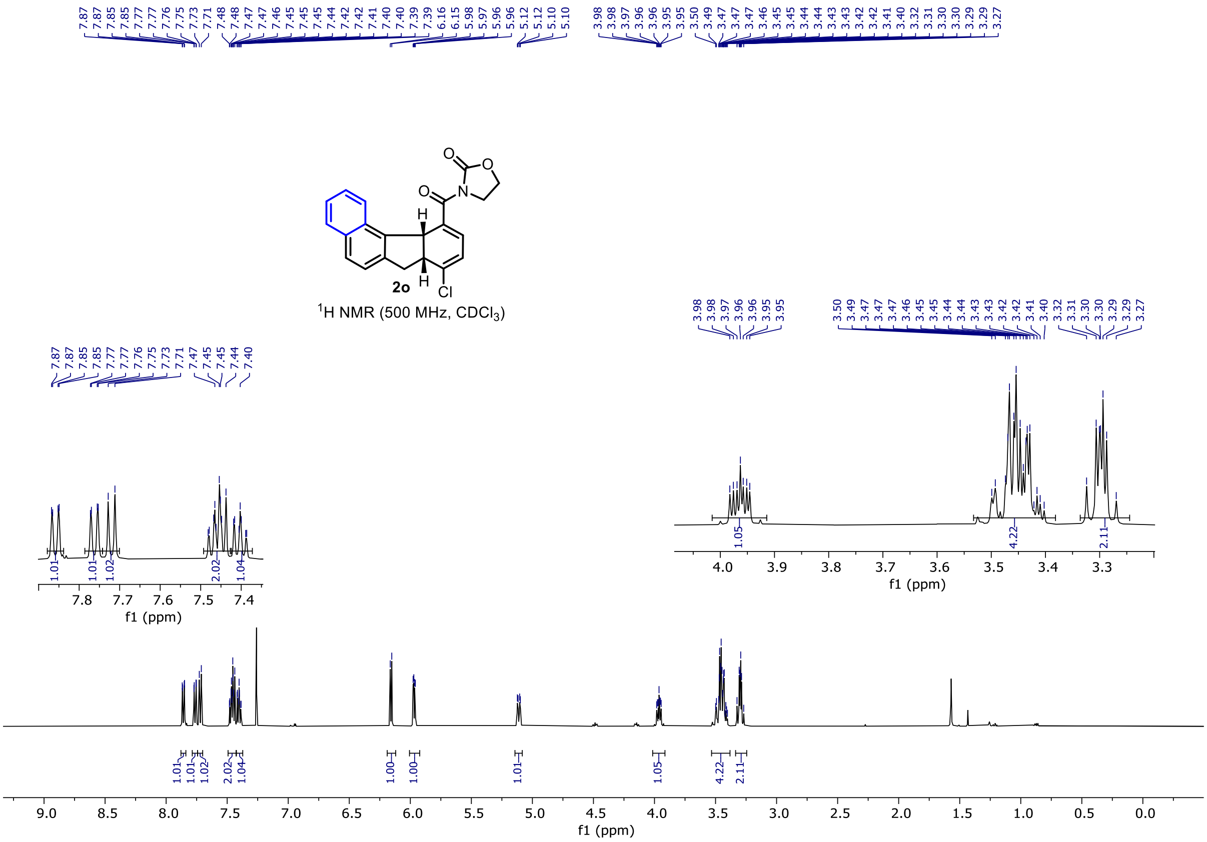

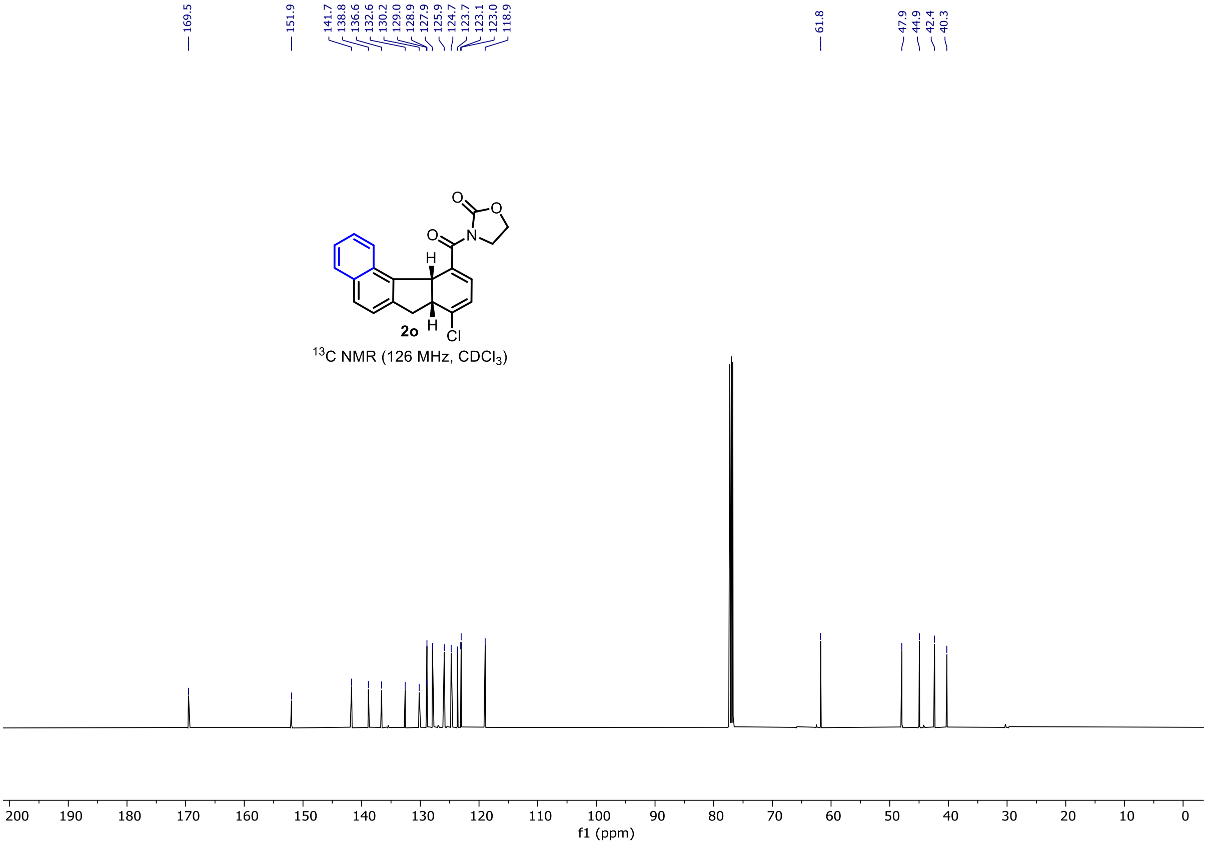

3-((6b*R*,10a*S*)-10-Chloro-6b,10a-dihydrofluoranthene-7-carbonyl)oxazolidin-2-one, 2p

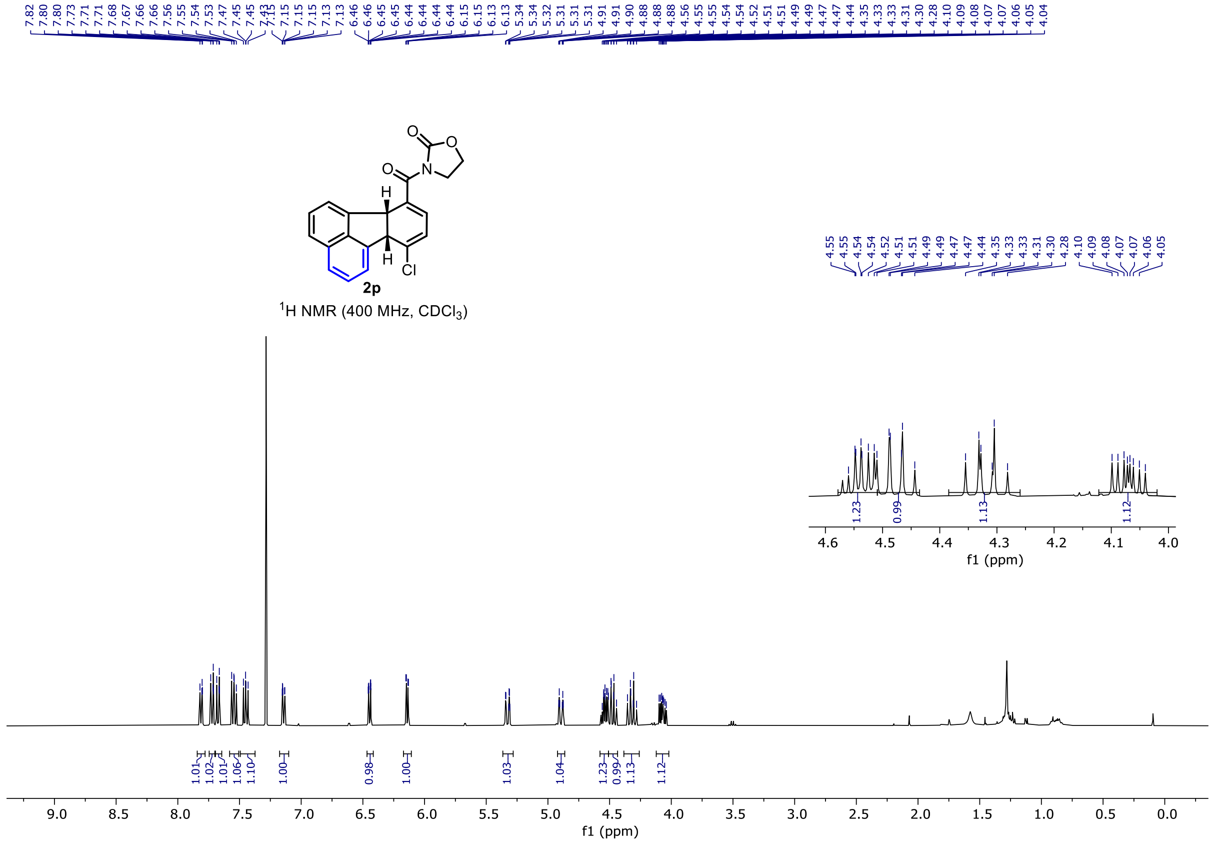

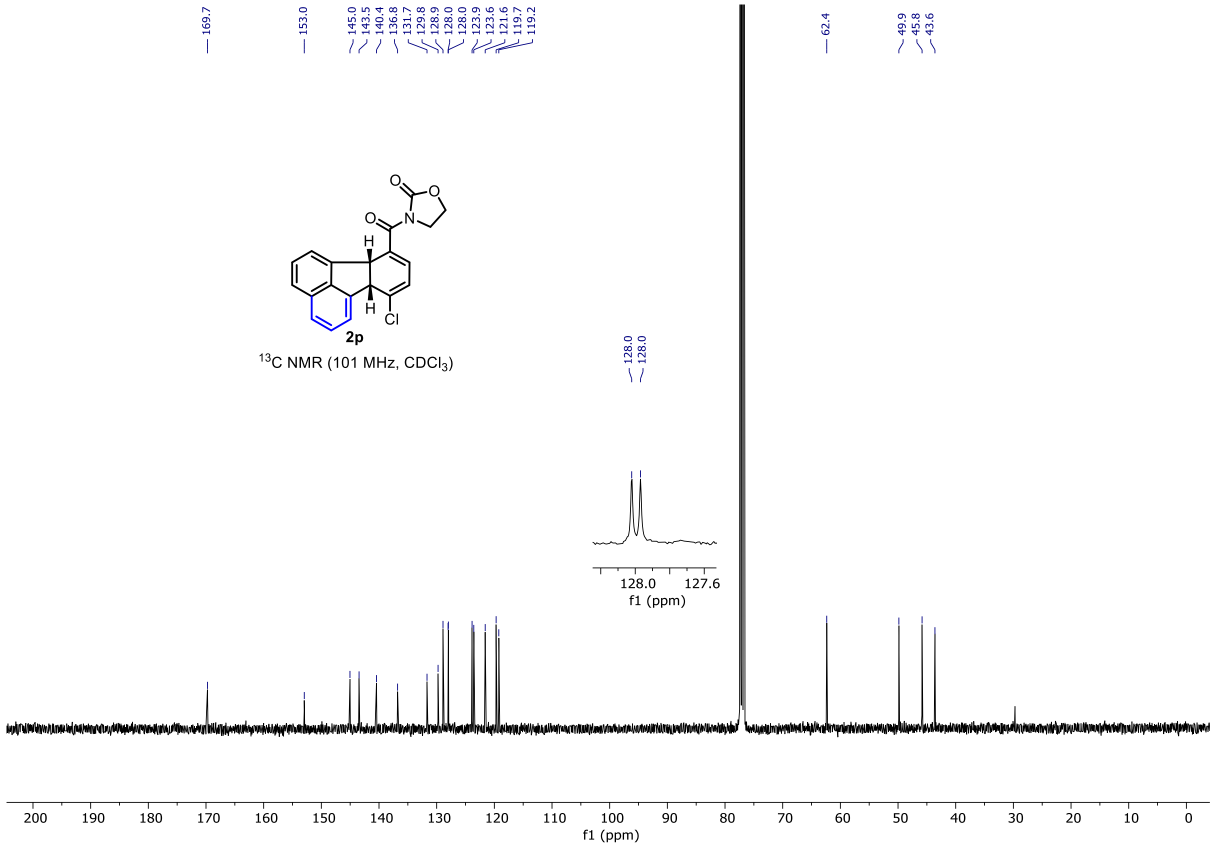

3-((4a*R*,9a*R*)-1-Methyl-4a,9a-dihydro-9*H*-fluorene-4-carbonyl)oxazolidin-2-one, 2q

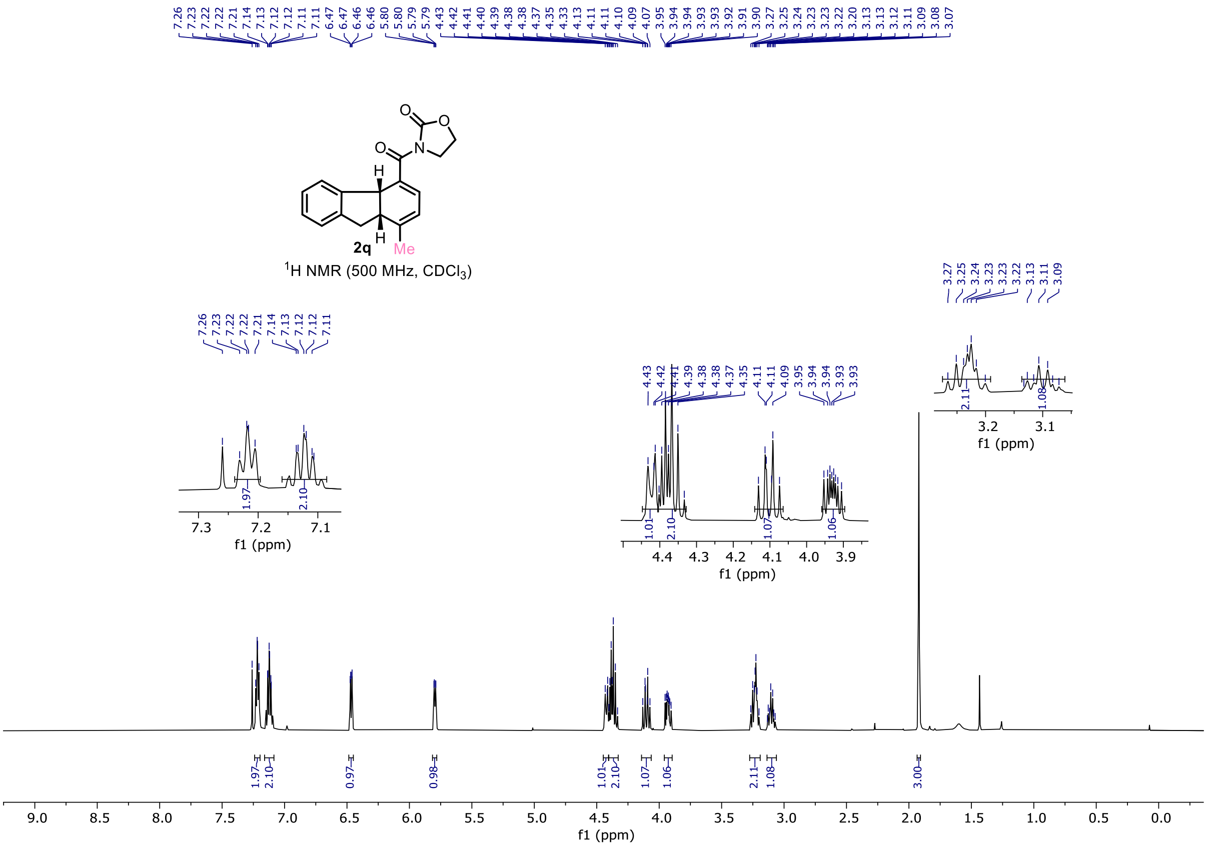

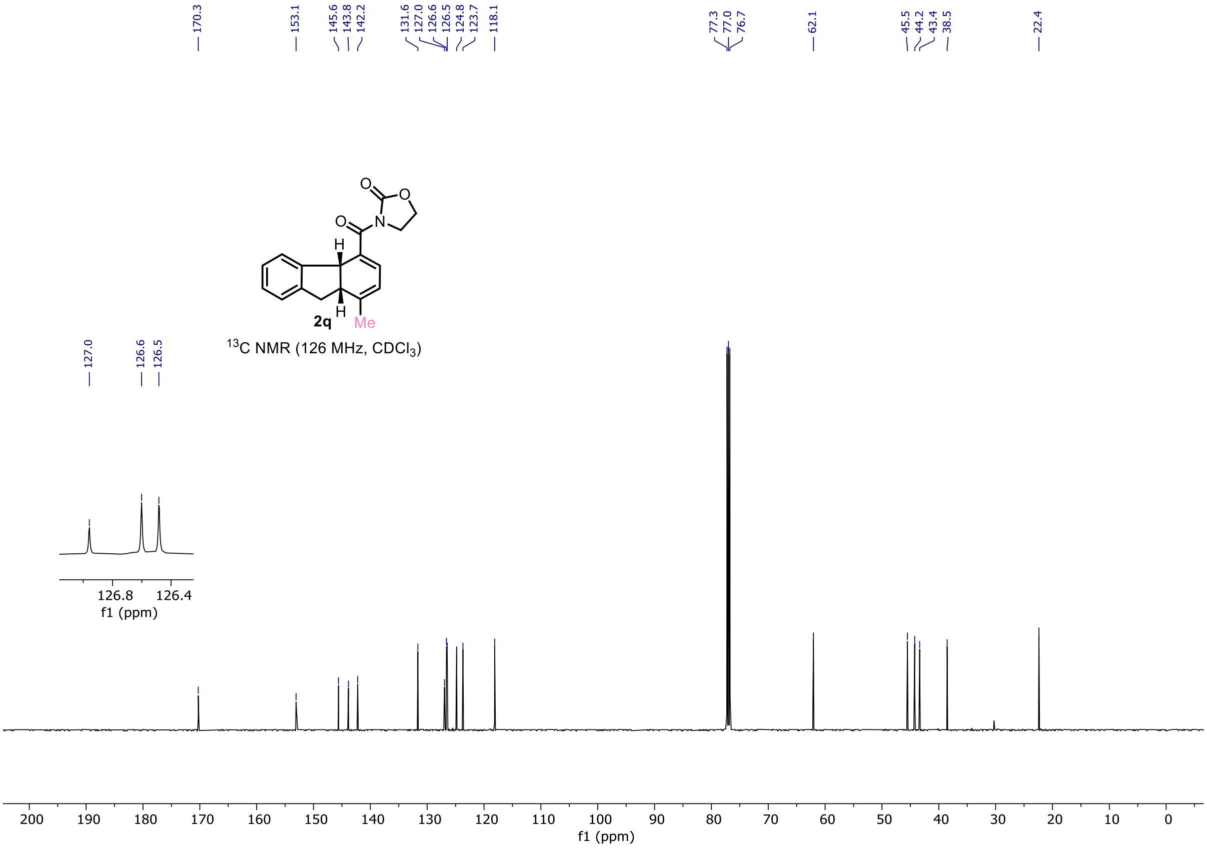

3-((4a*R*,9a*R*)-1-Bromo-4a,9a-dihydro-9*H*-fluorene-4-carbonyl)oxazolidin-2-one, 2r

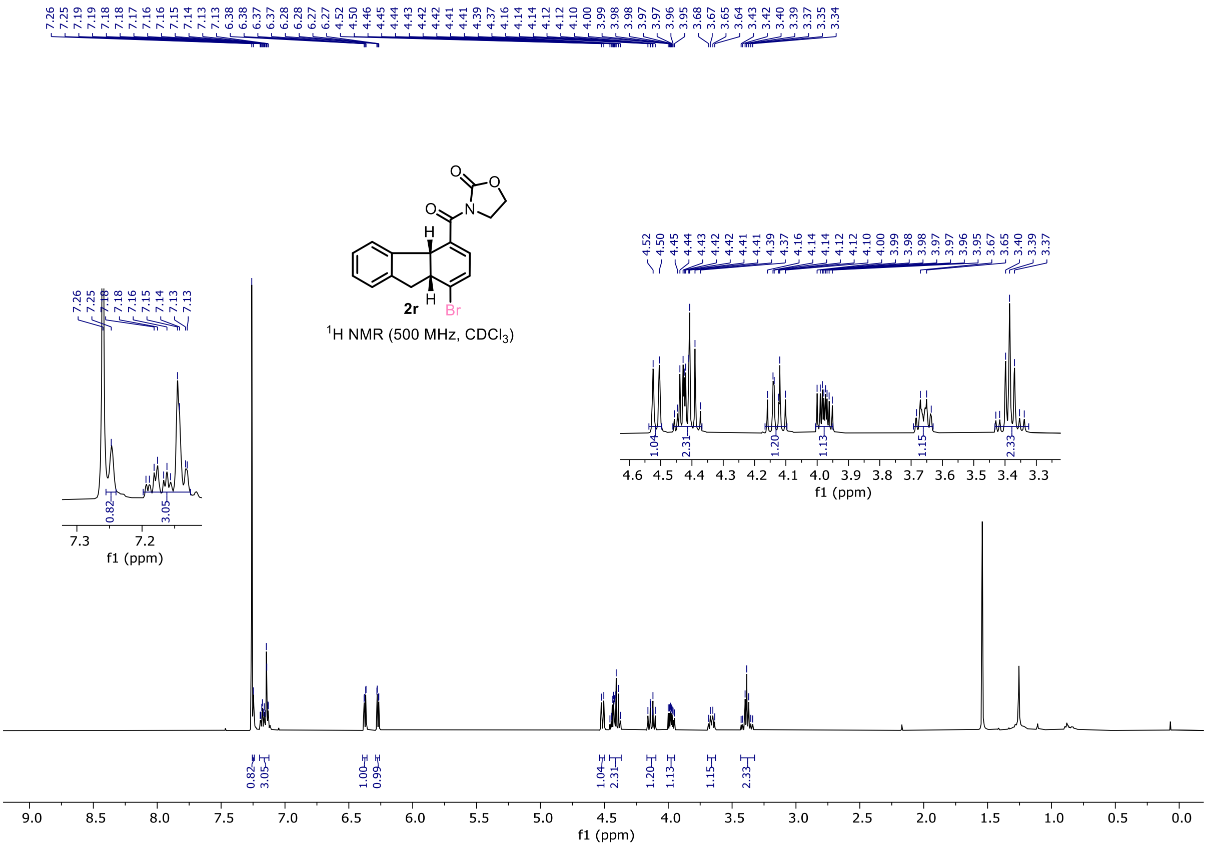

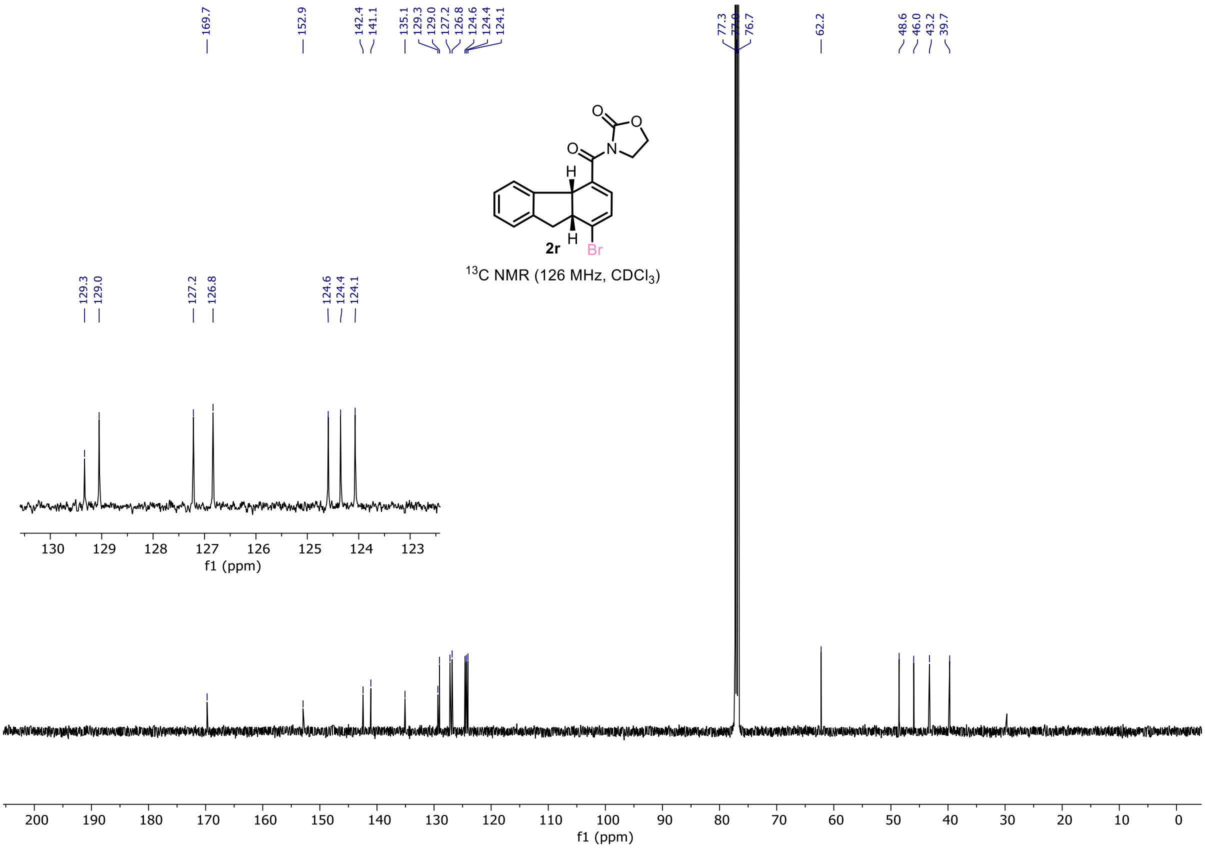

3-((5a*R*,10a*R*)-1,2,3,5a,10,10a-Hexahydrocyclopenta[*a*]fluorene-5-carbonyl)oxazolidin-2-one, 2s

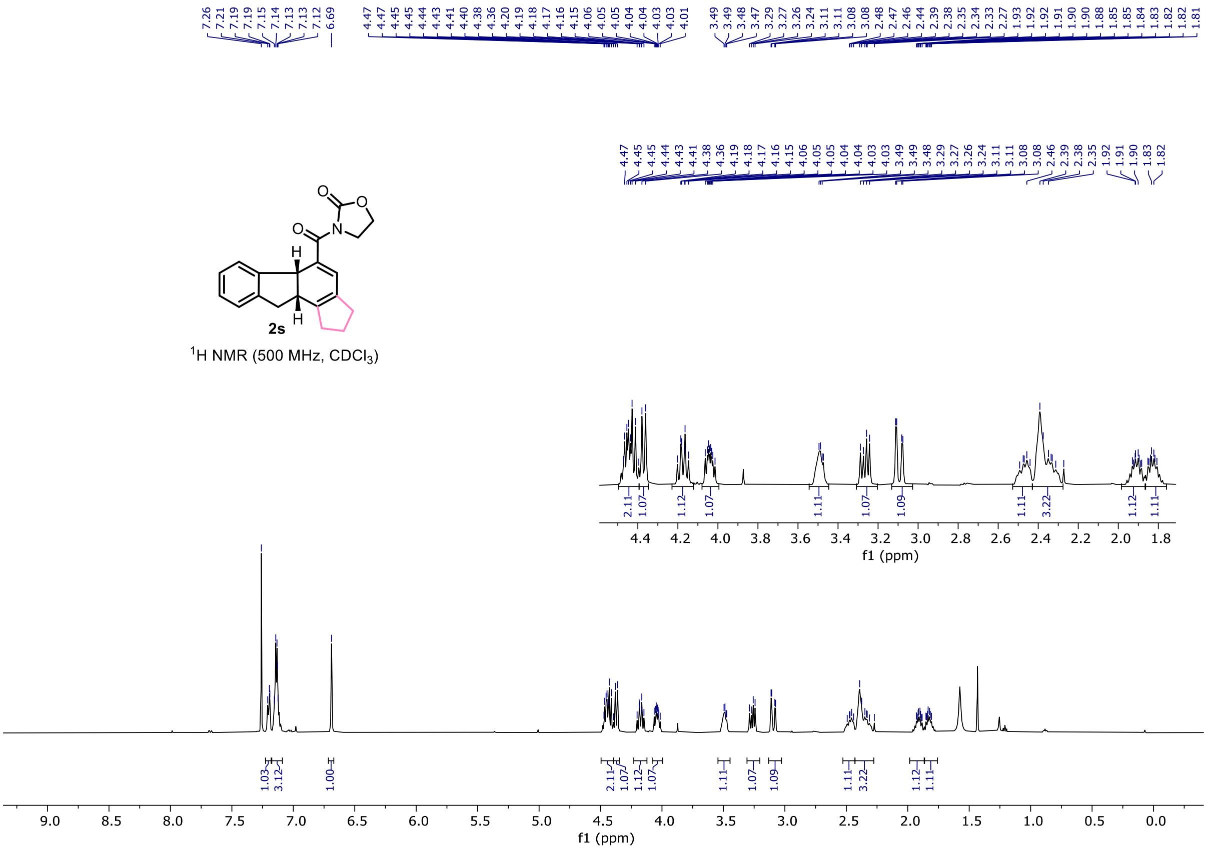

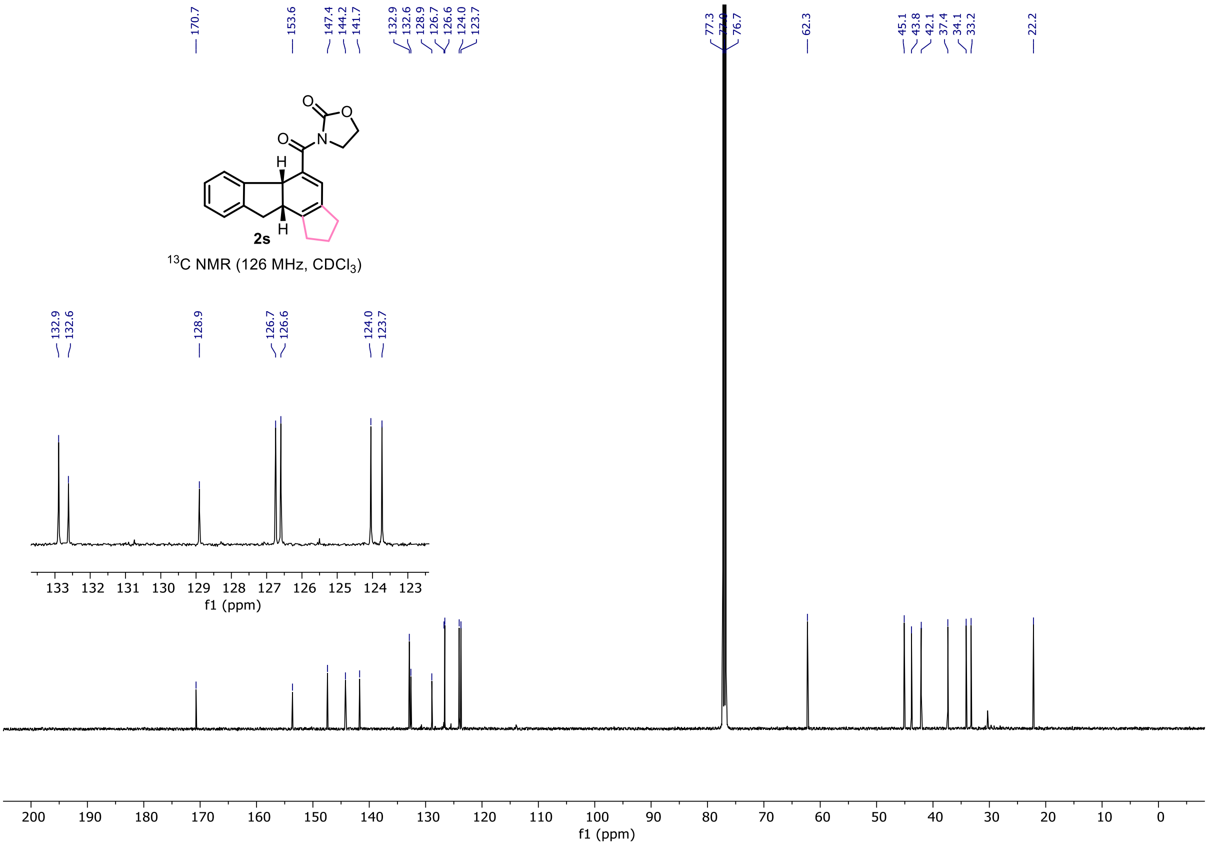

3-((4a*R*,9a*R*)-1-Bromo-2-methyl-4a,9a-dihydro-9*H*-fluorene-4-carbonyl)oxazolidin-2-one, 2t

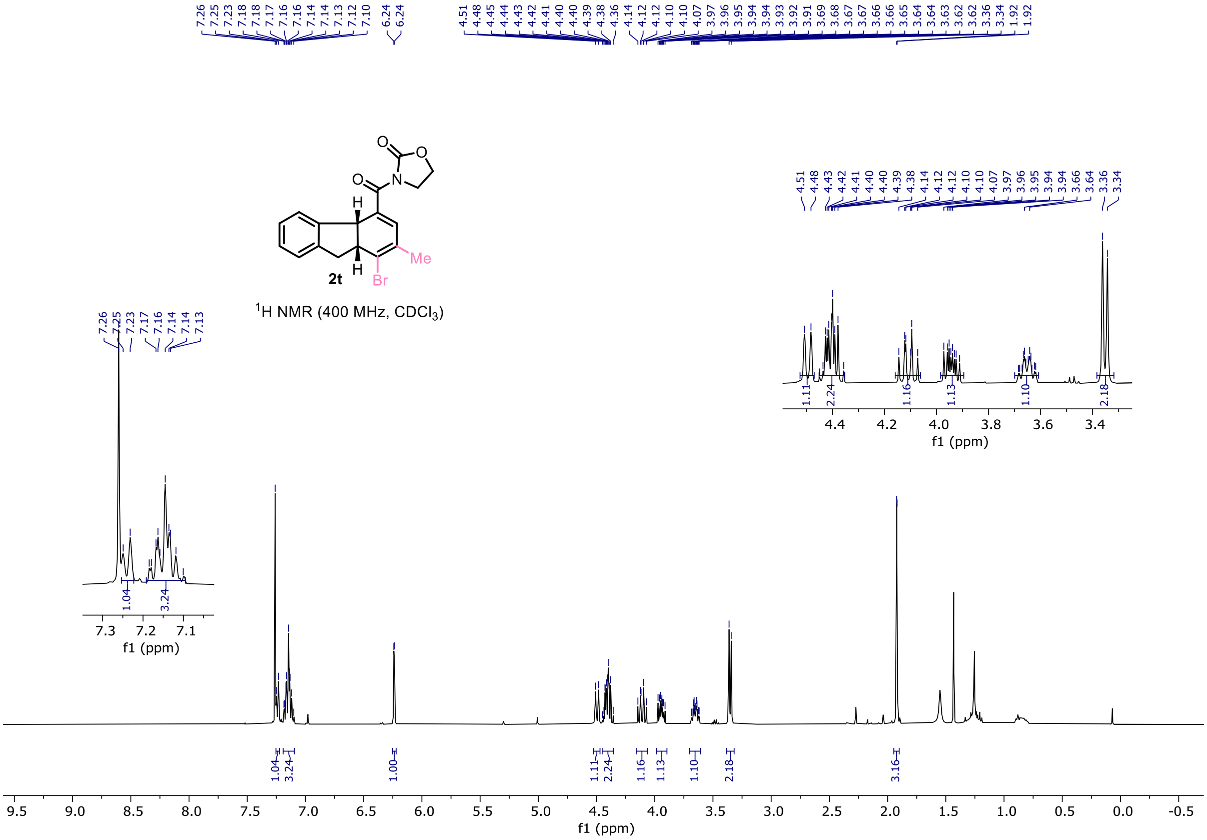

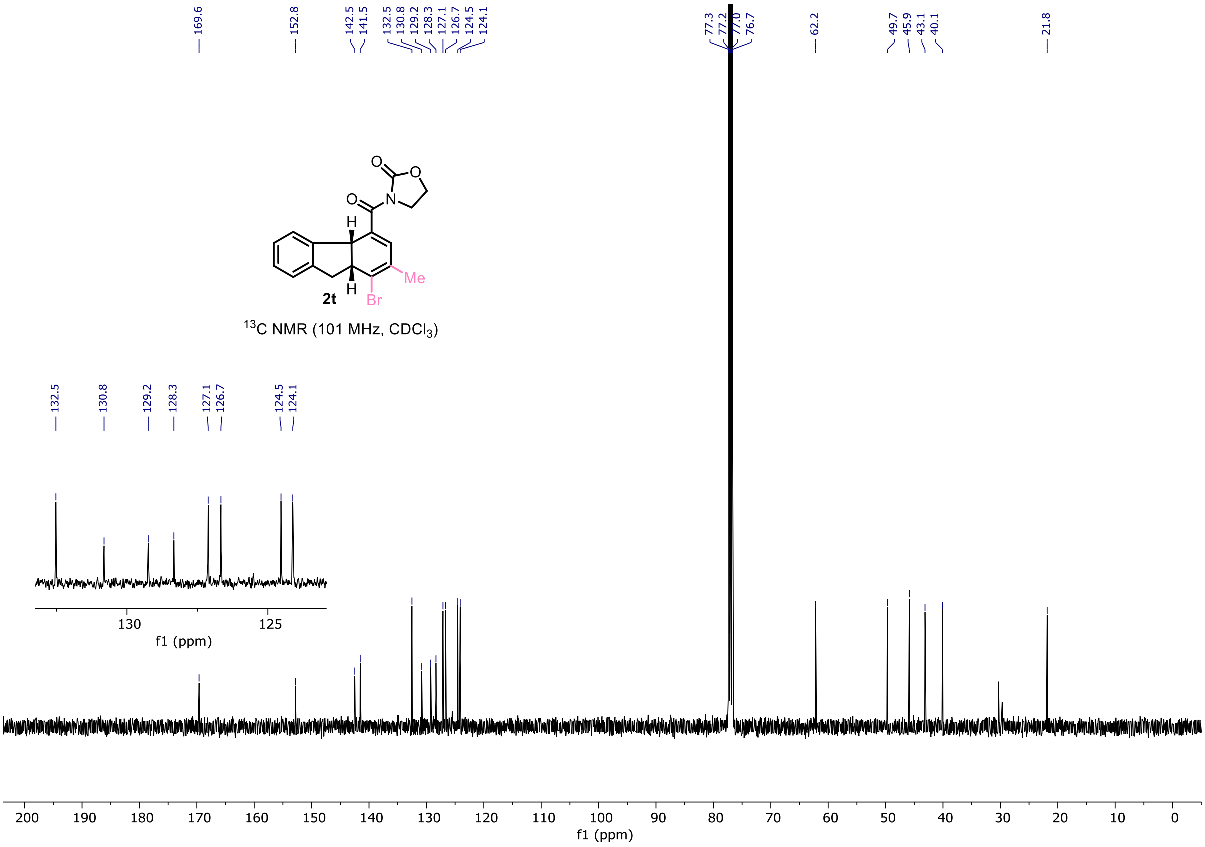

((4a*R*,9a*R*)-1-Chloro-4a,9a-dihydro-9*H*-fluoren-4-yl)methanol, 3

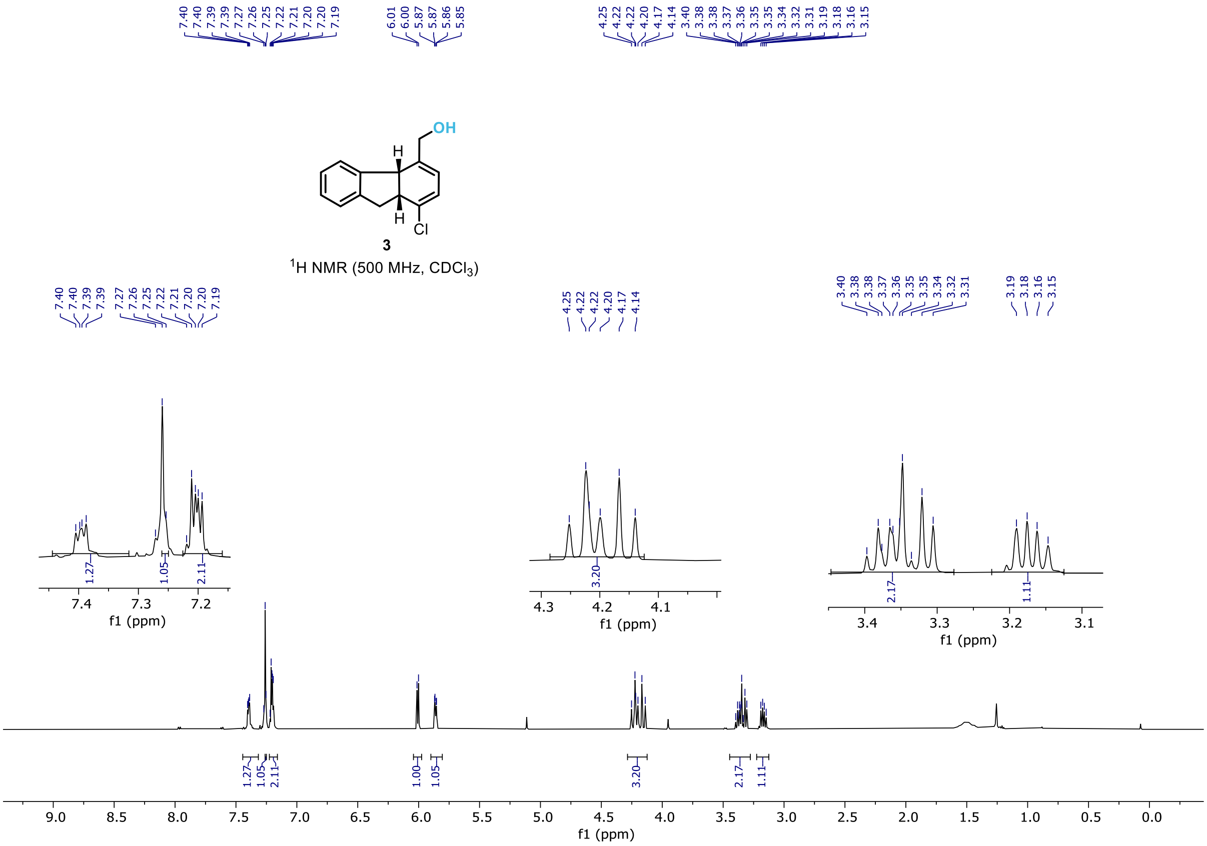

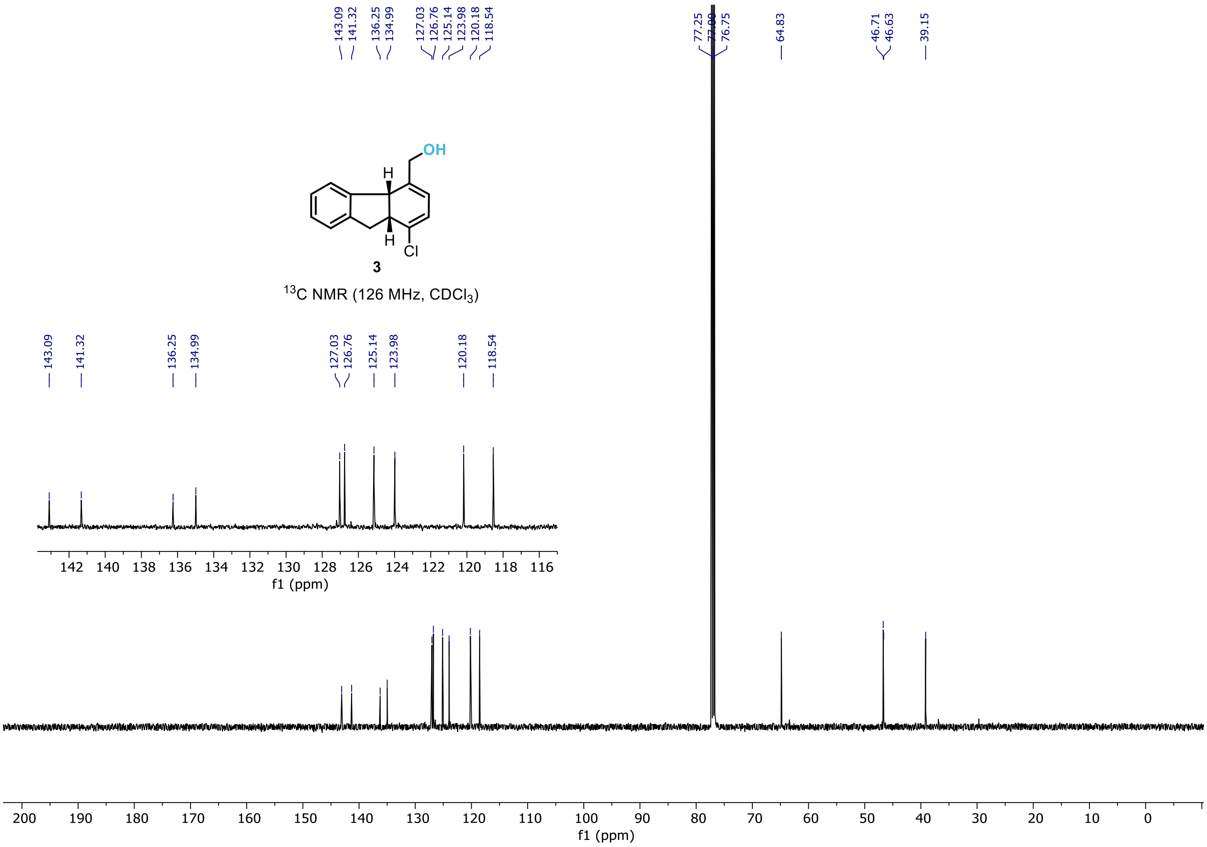

Methyl (4a*R*,9a*R*)-1-chloro-4a,9a-dihydro-9*H*-fluorene-4-carboxylate, 4

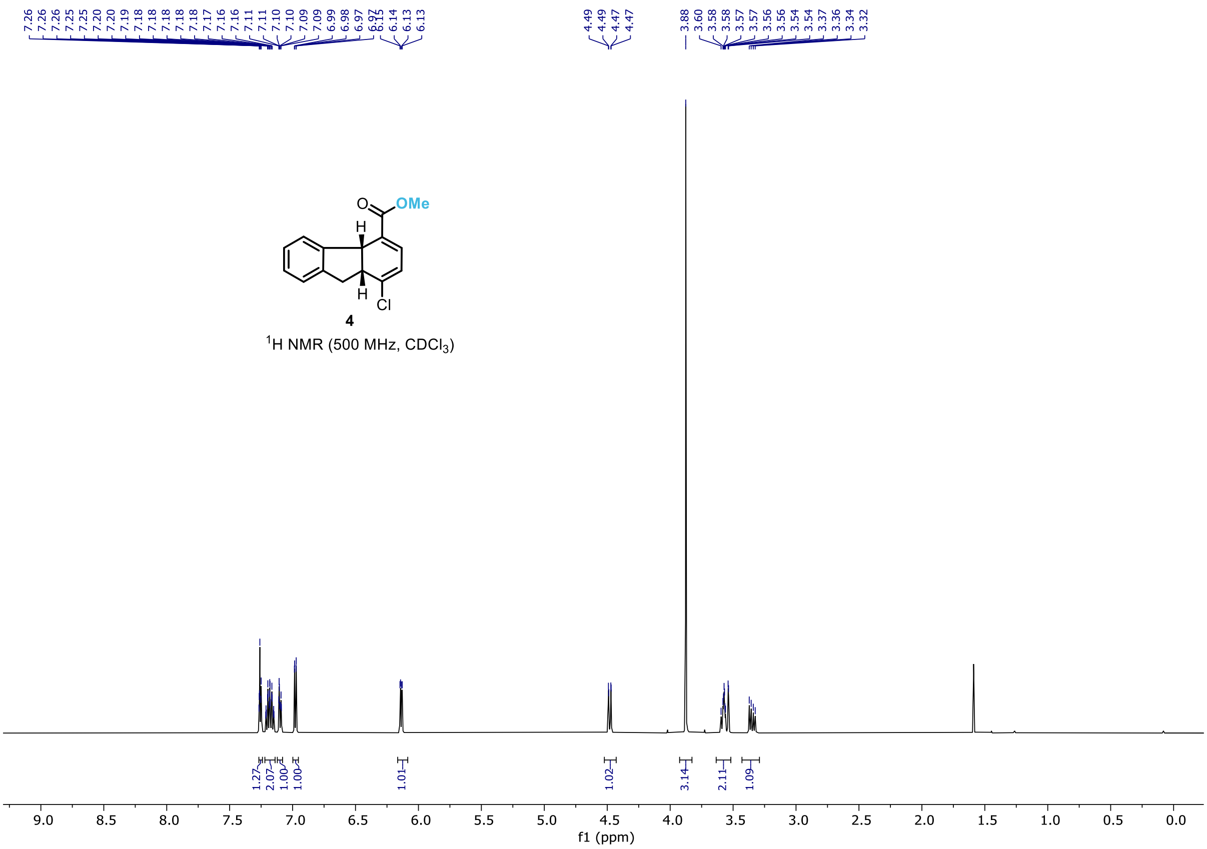

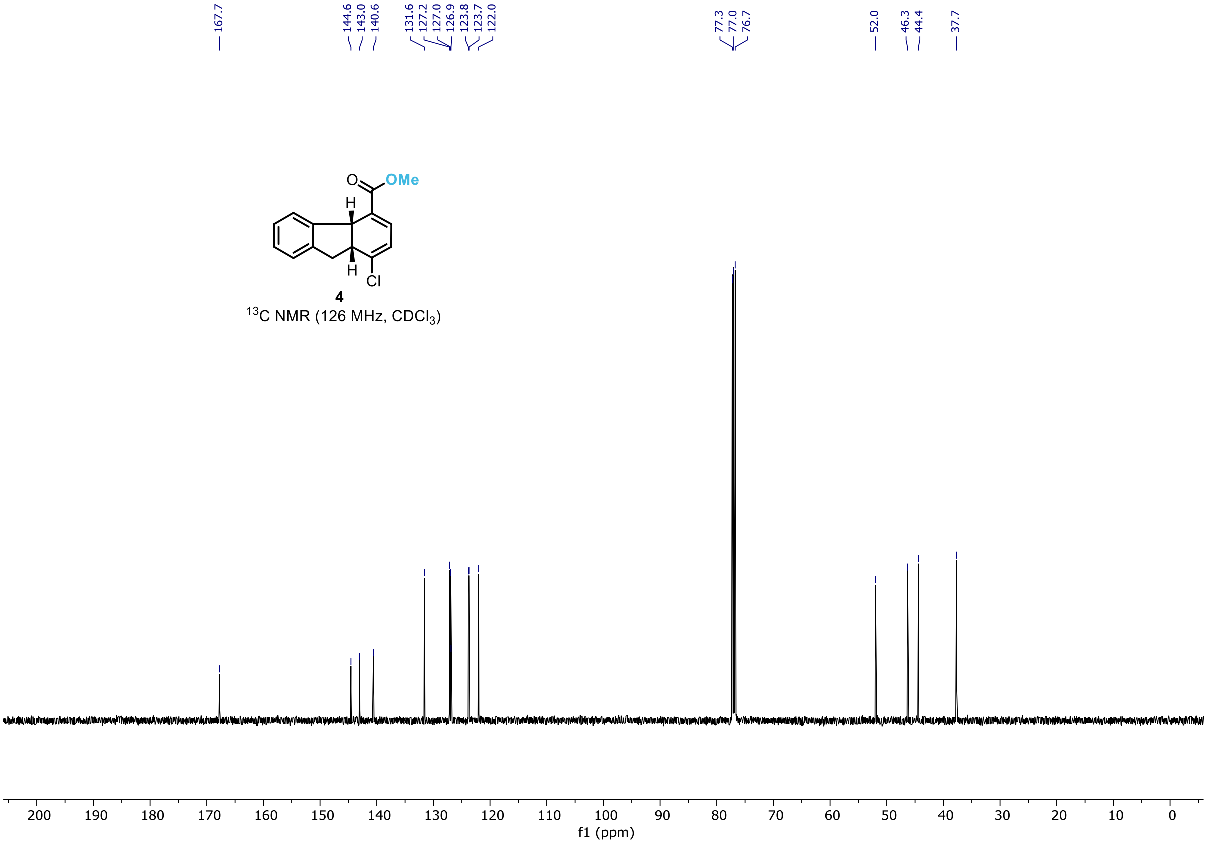

Methyl (4a*R*,9a*R*)-1-phenyl-4a,9a-dihydro-9*H*-fluorene-4-carboxylate, 5

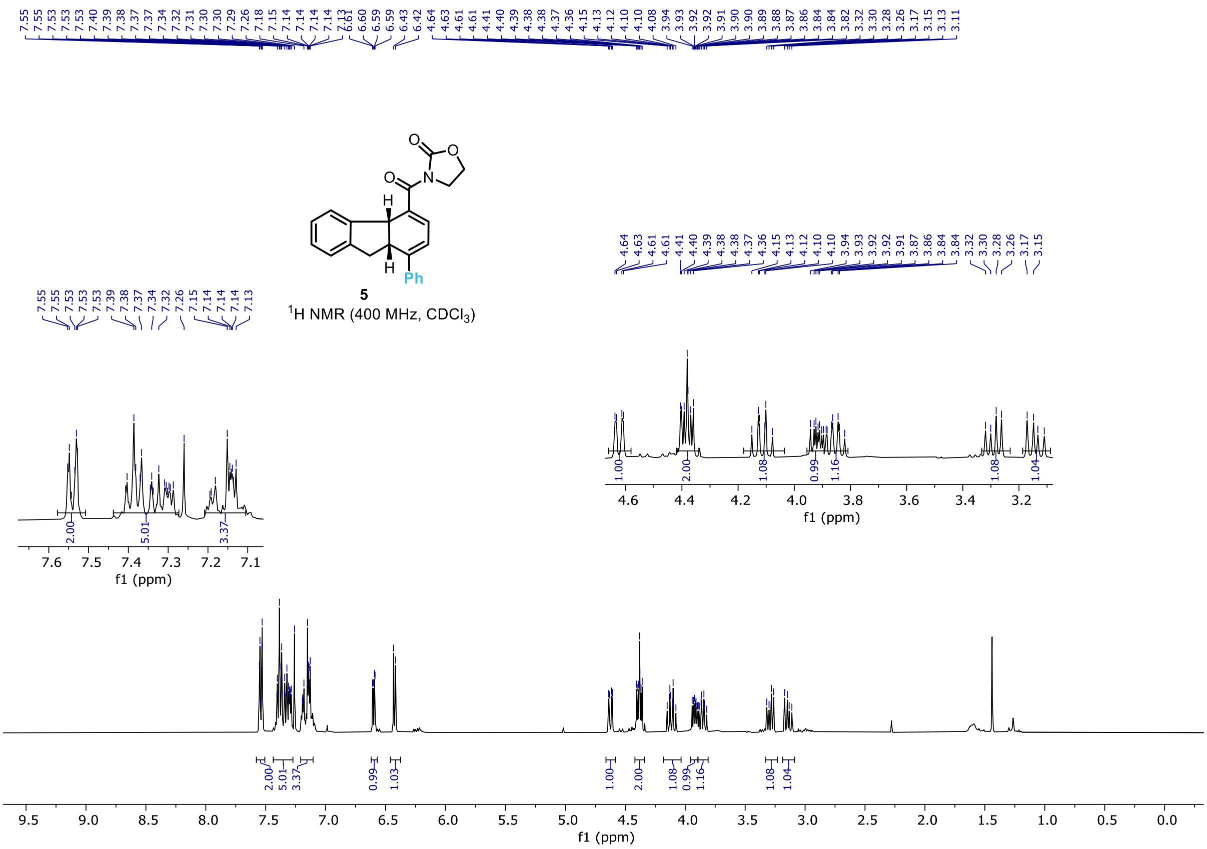

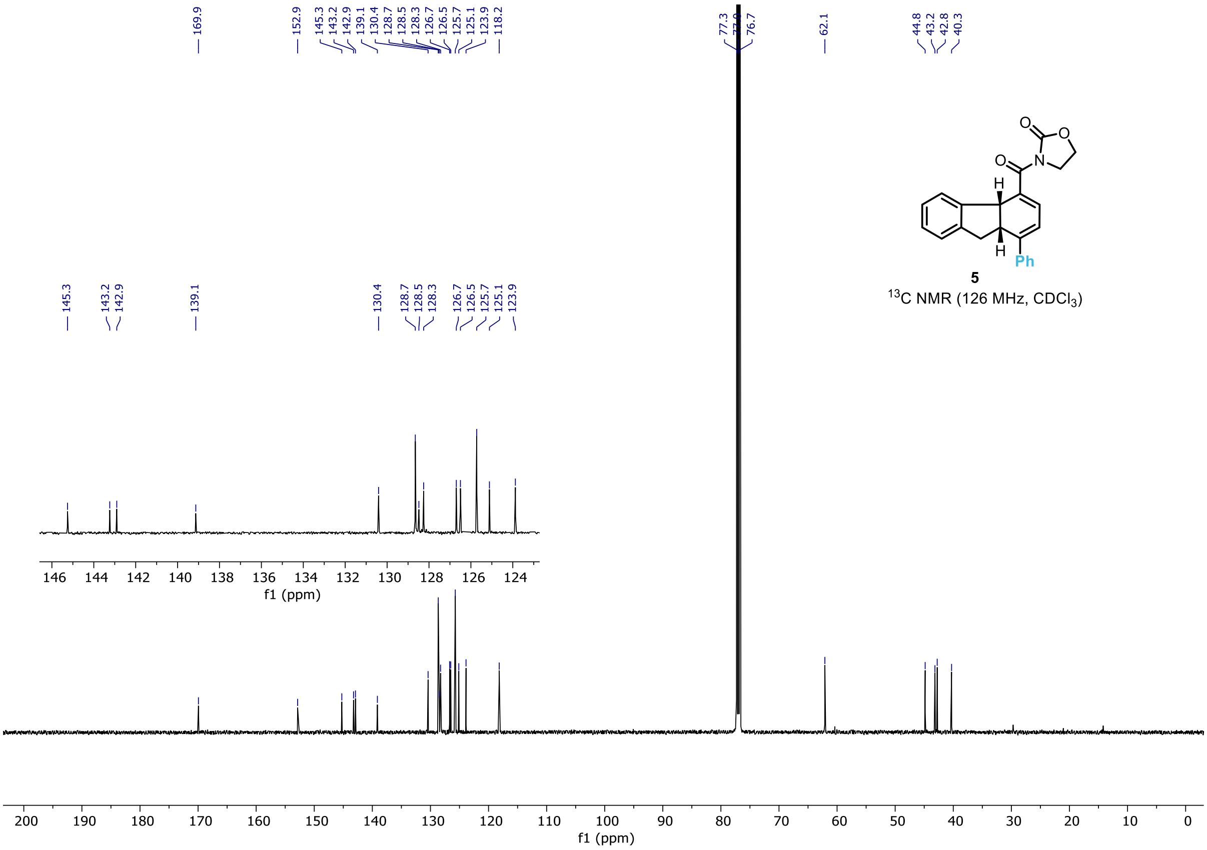

Methyl (4a*R*,9a*R*)-1-phenyl-4a,9a-dihydro-9*H*-fluorene-4-carboxylate, 6

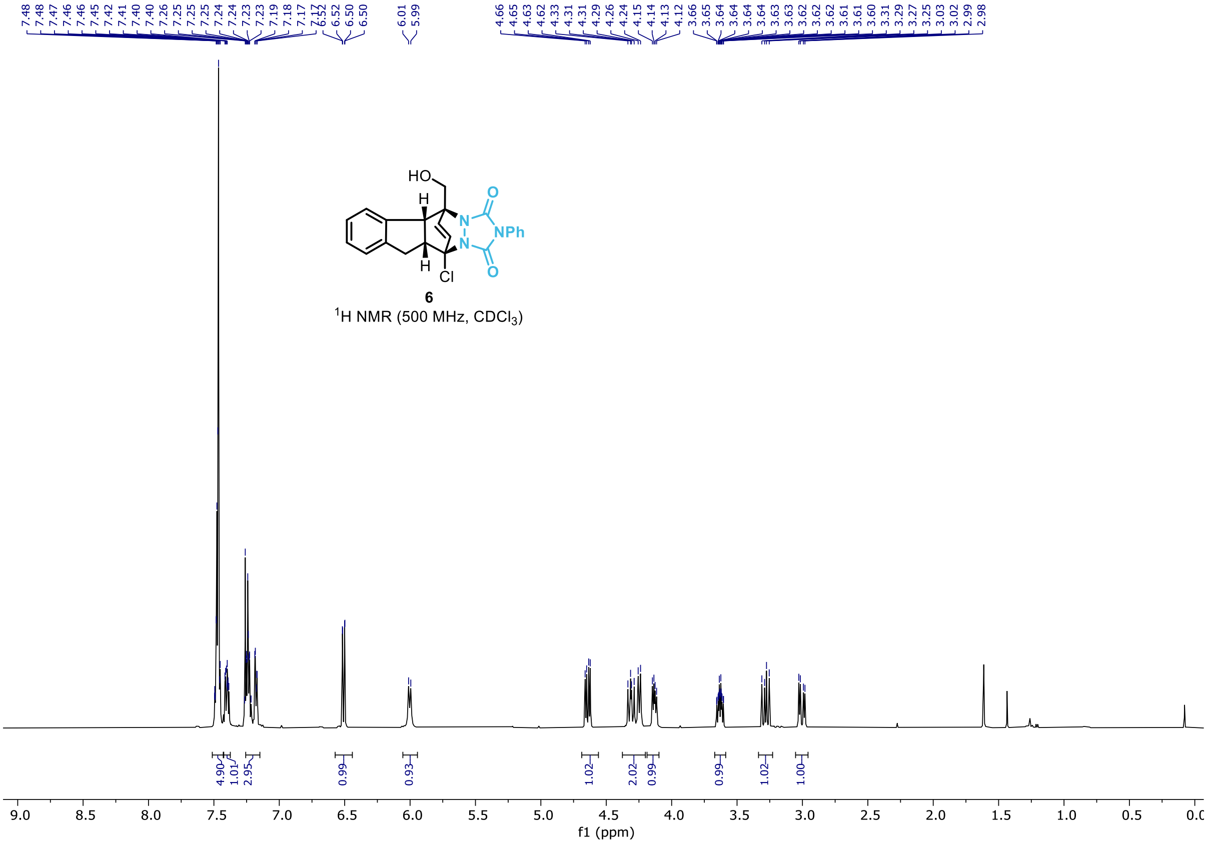

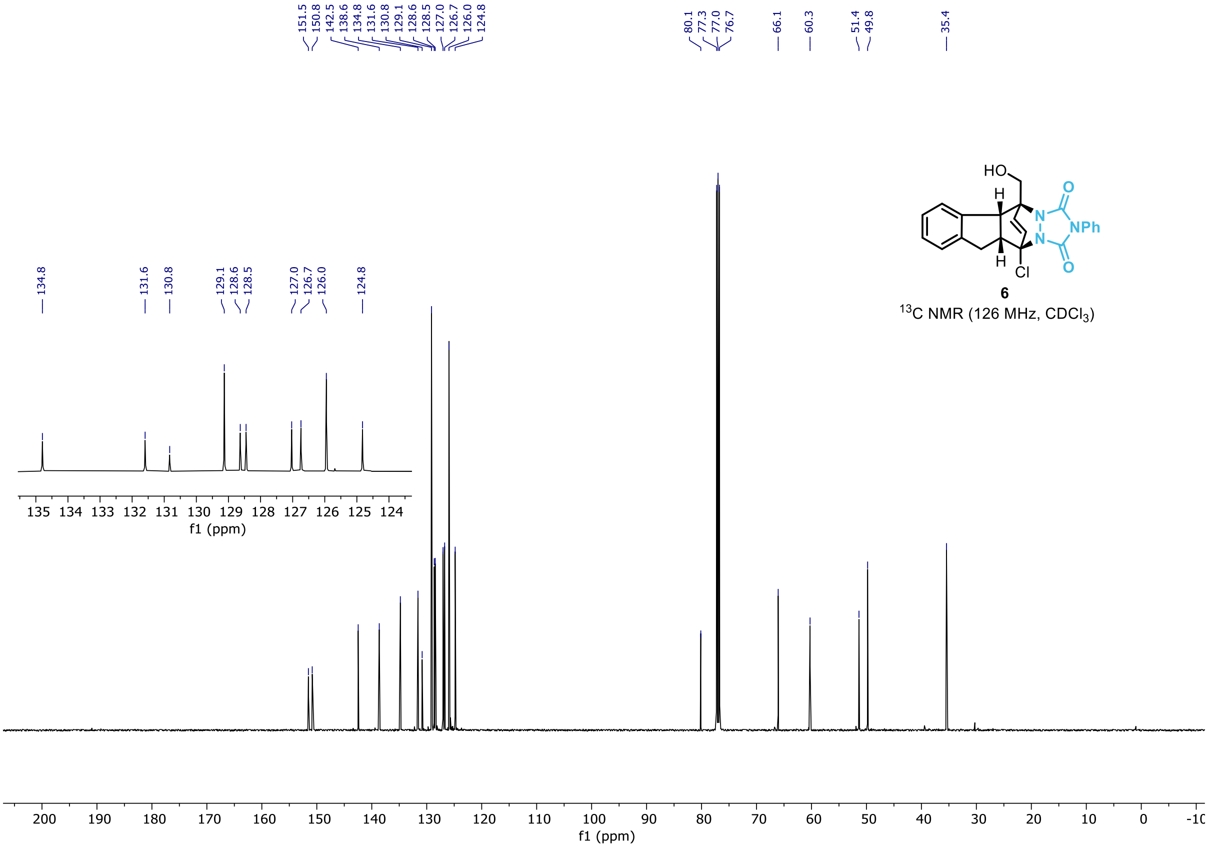

3-((4a*R*,8a*S*)-4-Chloro-4a,5,6,8a-tetrahydronaphthalene-1-carbonyl)oxazolidin-2-one, 7

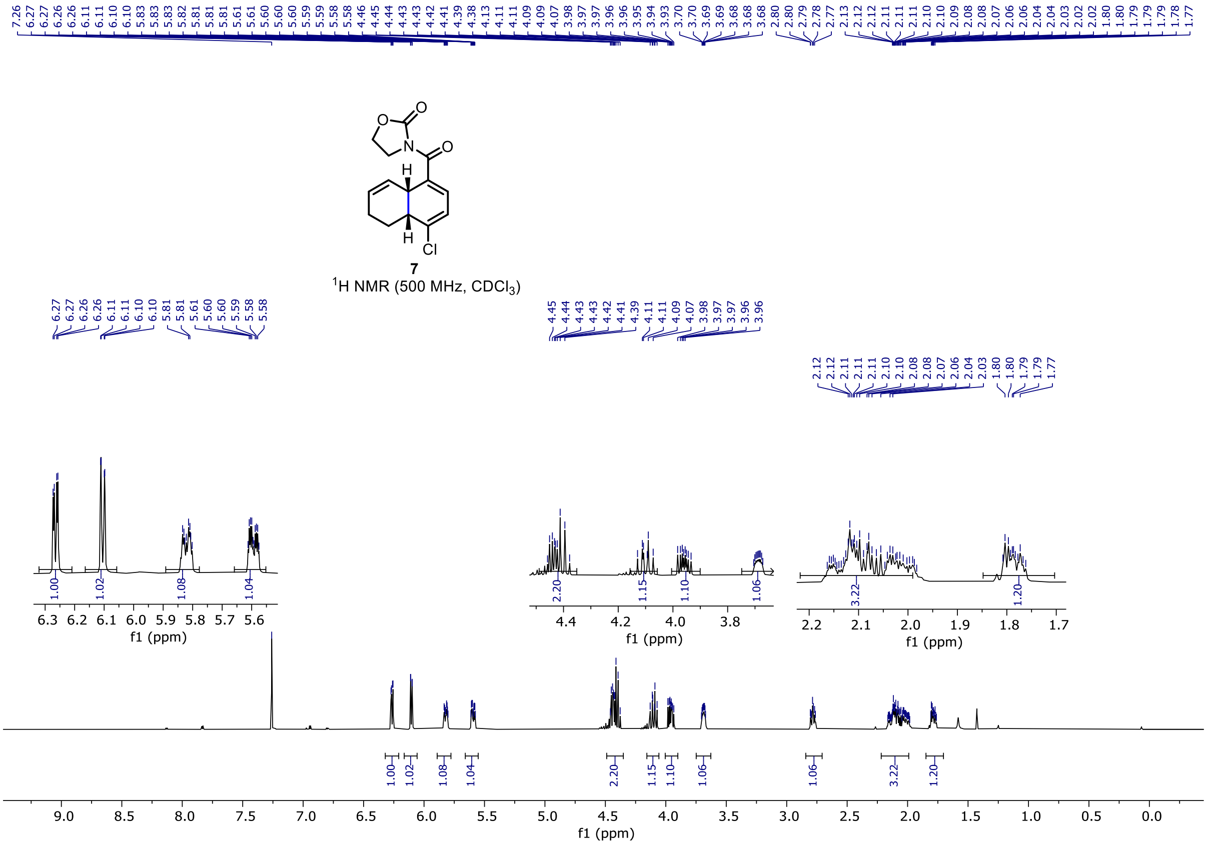

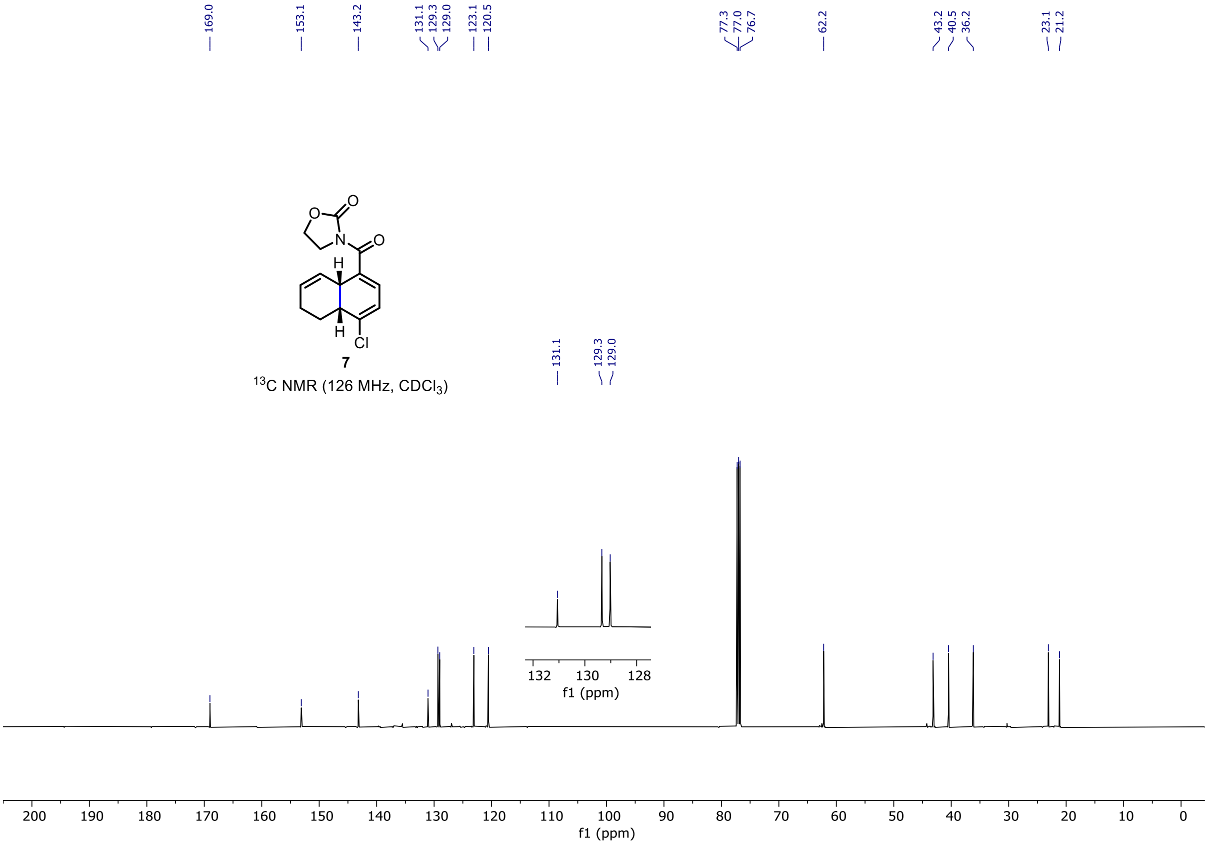

3-((4a*R*,9a*R*)-4-Chloro-4a,9-dimethyl-4a,9a-dihydro-9*H*-carbazole-1-carbonyl)oxazolidin-2-one, 9

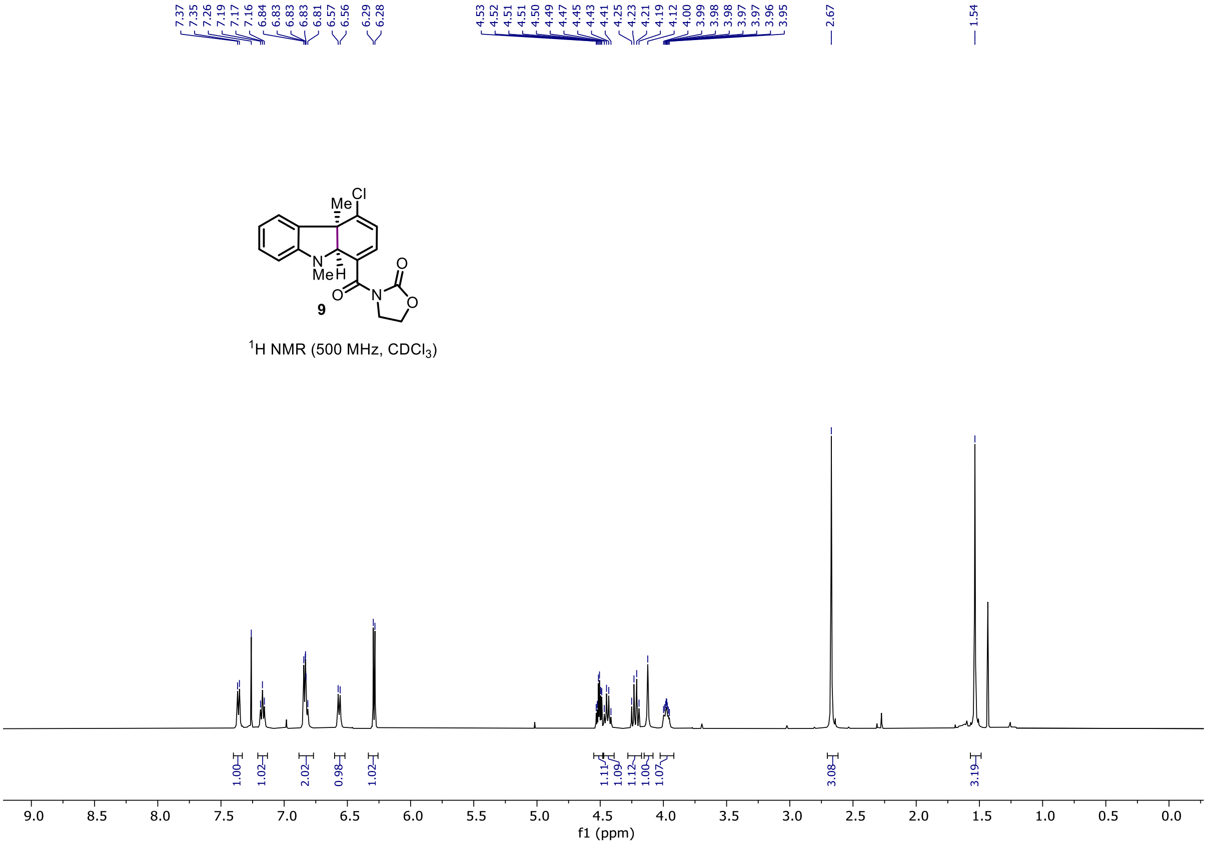

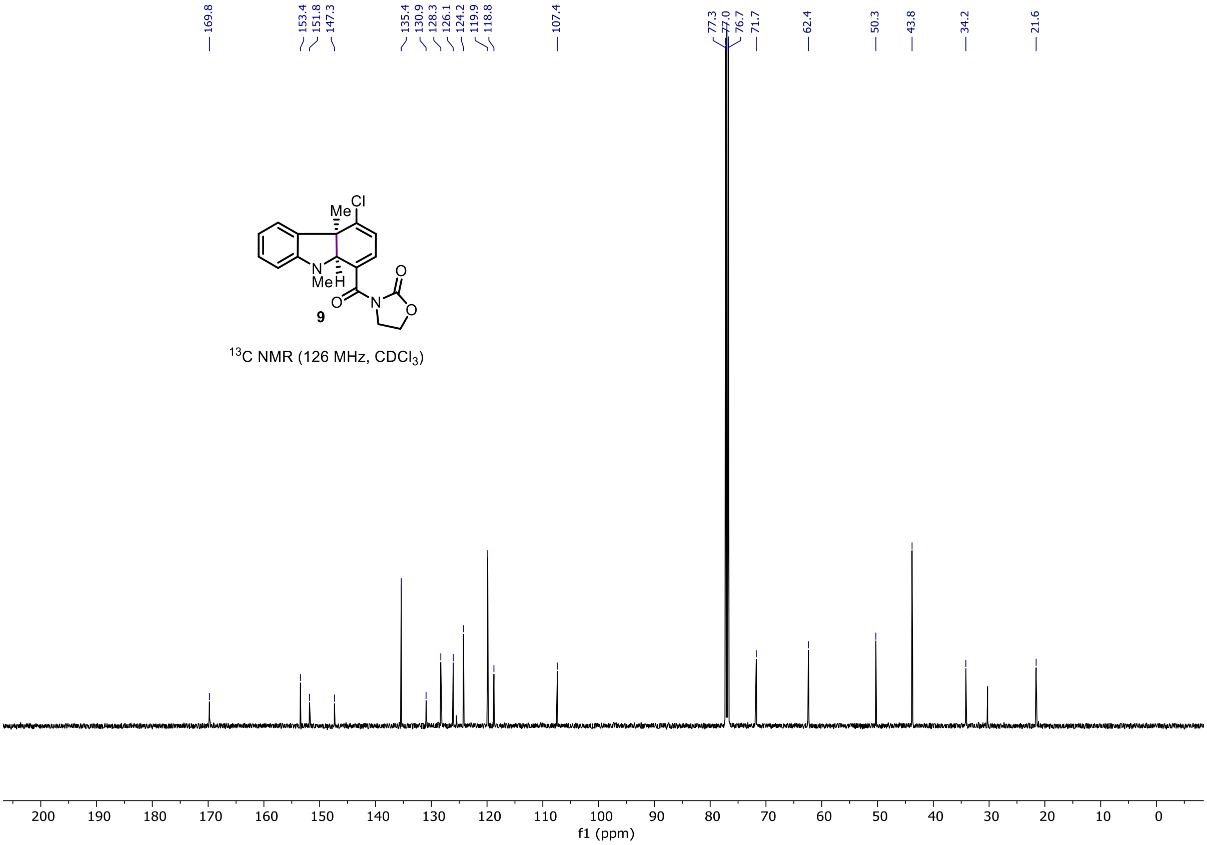

9. HPLC Traces

3-((4a*R*,9a*R*)-1-Chloro-4a,9a-dihydro-9*H*-fluorene-4-carbonyl)oxazolidin-2-one (2a)

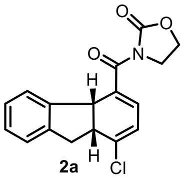

(CHIRALCEL® OD-H column, *n*-hexane/^{i}PrOH, 85:15 v/v, v = 1.0 mL/min, λ = 280 nm)

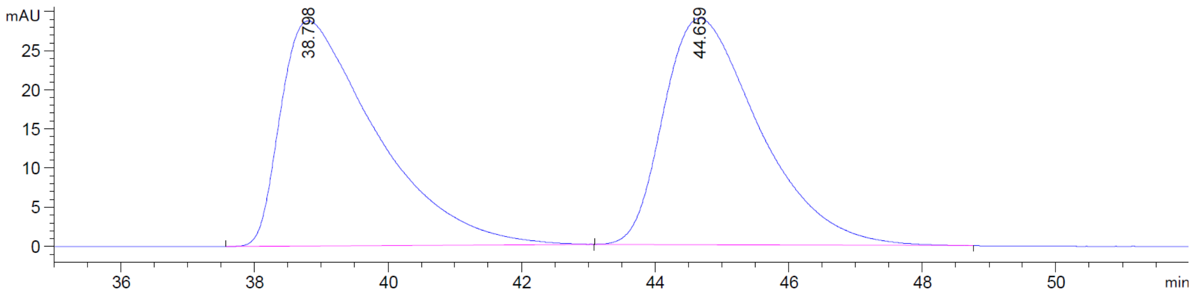

Peak #	RetTime [min]	Type	Width [min]	Area [mAU*s]	Height [mAU]	Area %
1	38.798	BB	1.3923	2862.41406	28.73277	49.7877
2	44.659	BB	1.4399	2886.82544	28.88660	50.2123

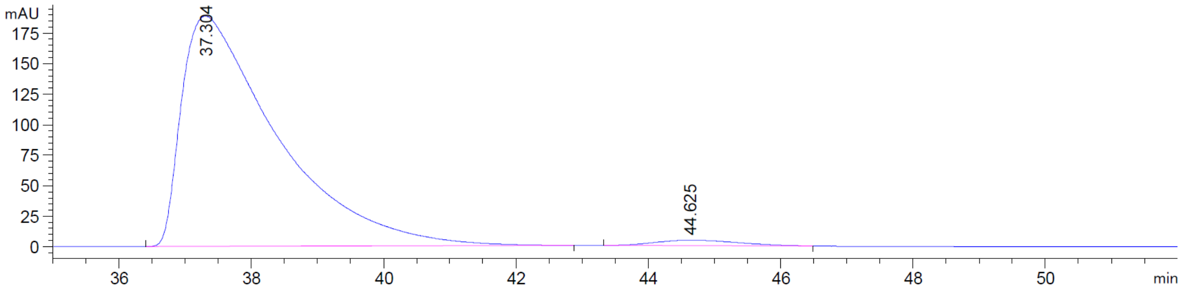

Peak #	RetTime [min]	Type	Width [min]	Area [mAU*s]	Height [mAU]	Area %
1	37.304	BB	1.3775	1.89479e4	188.70131	97.9323
2	44.625	BB	1.0229	400.05914	4.59877	2.0677

3-((4a*R*,9a*R*)-7-Bromo-1-chloro-4a,9a-dihydro-9*H*-fluorene-4-carbonyl)oxazolidin-2-one (2b)

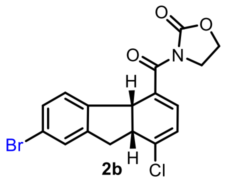

(CHIRALPAK IA column, *n*-hexane/^{i}PrOH, 85:15 v/v, v = 1.0 mL/min, λ = 230 nm)

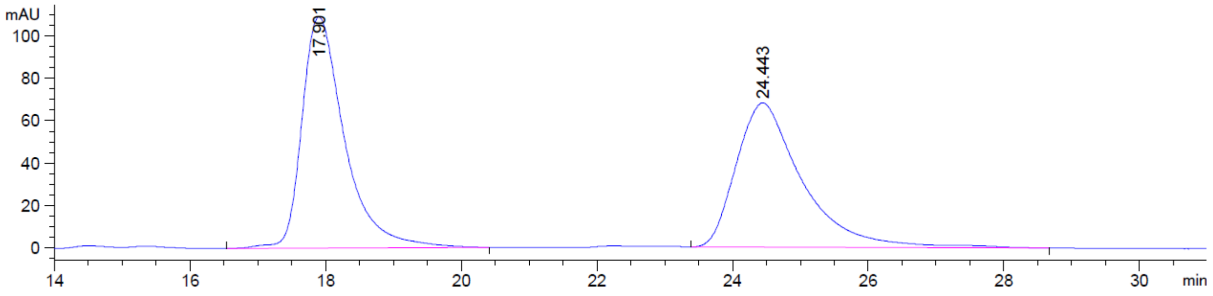

Peak #	RetTime [min]	Type	Width [min]	Area [mAU*s]	Height [mAU]	Area %
1	17.901	BB	0.6546	4781.53564	109.26334	50.7841
2	24.443	BB	0.9809	4633.88330	68.18298	49.2159

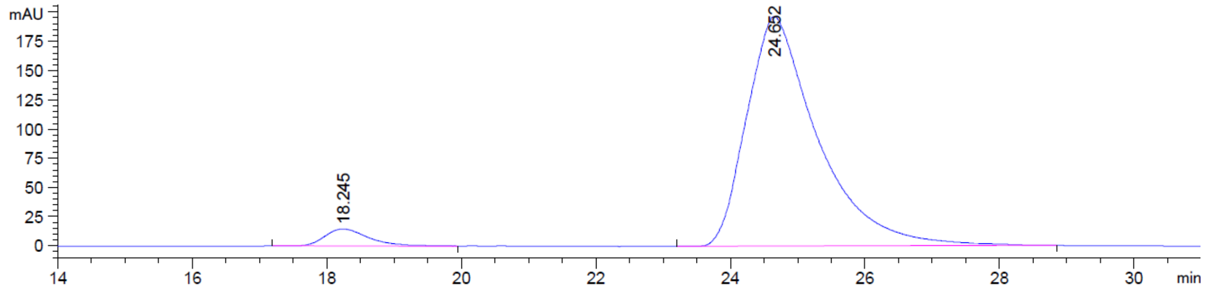

Peak #	RetTime [min]	Type	Width [min]	Area [mAU*s]	Height [mAU]	Area %
1	18.245	BB	0.6277	663.21924	14.60628	4.3958
2	24.652	BB	1.0680	1.44242e4	196.60190	95.6042

3-((4a*R*,9a*R*)-1-Chloro-7-fluoro-4a,9a-dihydro-9*H*-fluorene-4-carbonyl)oxazolidin-2-one (2c)

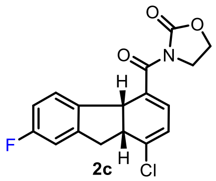

CHIRALPAK IA column, *n*-hexane/^{i}PrOH, 85:15 v/v, v = 1.0 mL/min, λ = 280 nm

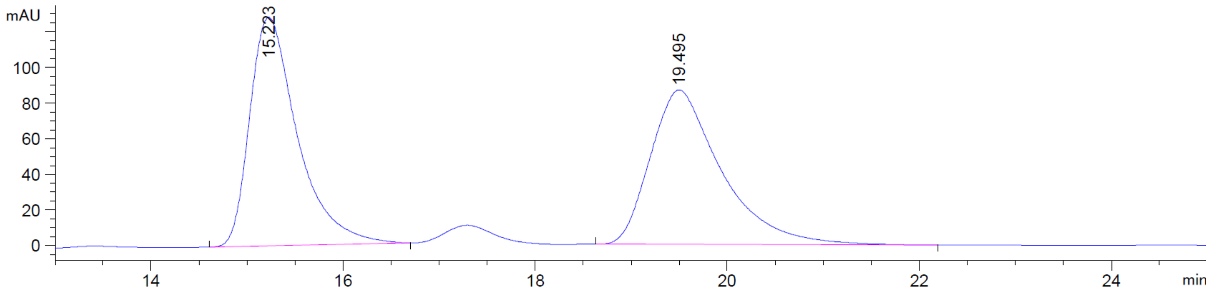

Peak #	RetTime [min]	Type	Width [min]	Area [mAU*s]	Height [mAU]	Area %
1	15.223	BB	0.5227	4425.83691	128.22649	49.8607
2	19.495	BB	0.7573	4450.57080	86.64338	50.1393

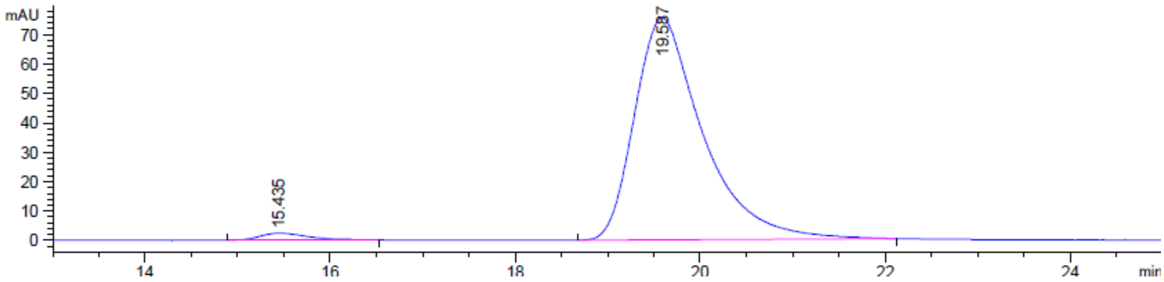

Peak #	RetTime [min]	Type	Width [min]	Area [mAU*s]	Height [mAU]	Area %
1	15.435	BB	0.4416	86.66978	2.40997	2.1865
2	19.587	BB	0.7555	3877.26978	75.70689	97.8135

3-((4a*R*,9a*R*)-8-Bromo-1-chloro-4a,9a-dihydro-9*H*-fluorene-4-carbonyl)oxazolidin-2-one (2d)

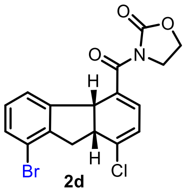

2d

(CHIRALPAK IA column, *n*-hexane/^{i}PrOH, 70:30 v/v, v = 1.0 mL/min, λ = 254 nm)

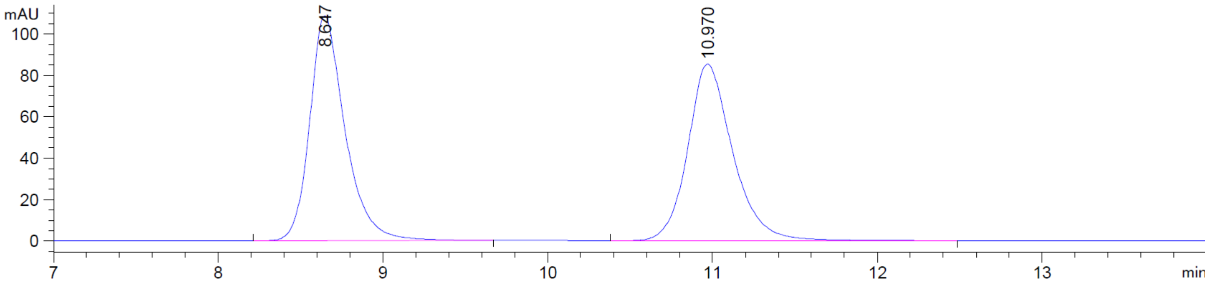

Peak #	RetTime [min]	Type	Width [min]	Area [mAU*s]	Height [mAU]	Area %
1	8.647	BB	0.2223	1621.85901	108.56036	49.9436
2	10.970	BB	0.2843	1625.52515	85.31735	50.0564

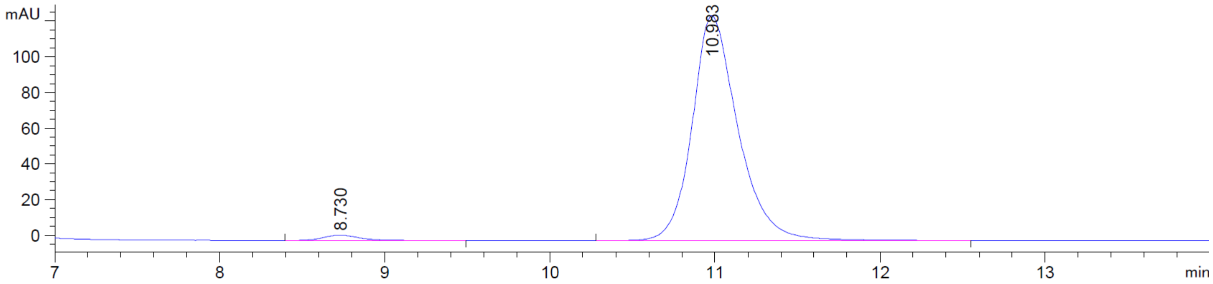

Peak #	RetTime [min]	Type	Width [min]	Area [mAU*s]	Height [mAU]	Area %
1	8.730	BB	0.2401	48.15359	3.01865	1.9352
2	10.983	BB	0.2876	2440.08496	126.16900	98.0648

(4b*R*,8a*R*)-8-Chloro-5-(2-oxooxazolidine-3-carbonyl)-4b,8a-dihydro-9*H*-fluorene-2-carbonitrile (2e)

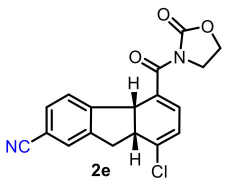

2e

(CHIRALPAK IA column, *n*-hexane/^{i}PrOH, 70:30 v/v, v = 1.0 mL/min, λ = 280 nm)

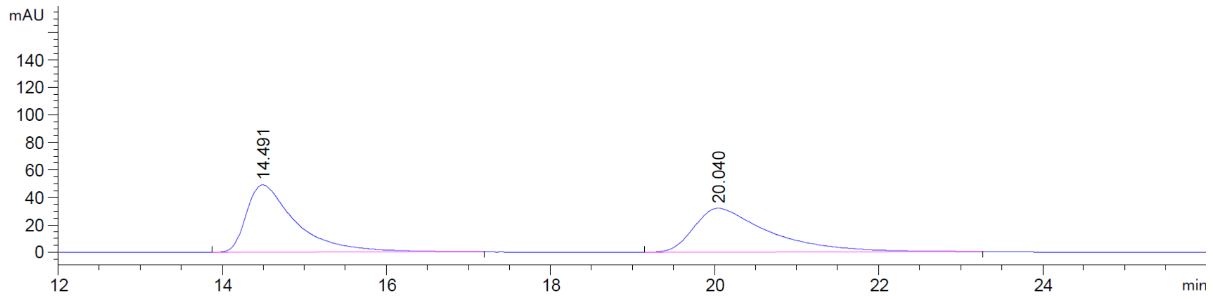

Peak #	RetTime [min]	Type	Width [min]	Area [mAU*s]	Height [mAU]	Area %
1	14.491	BB	0.6076	2055.43237	48.98053	50.3758
2	20.040	BB	0.8939	2024.76697	31.92839	49.6242

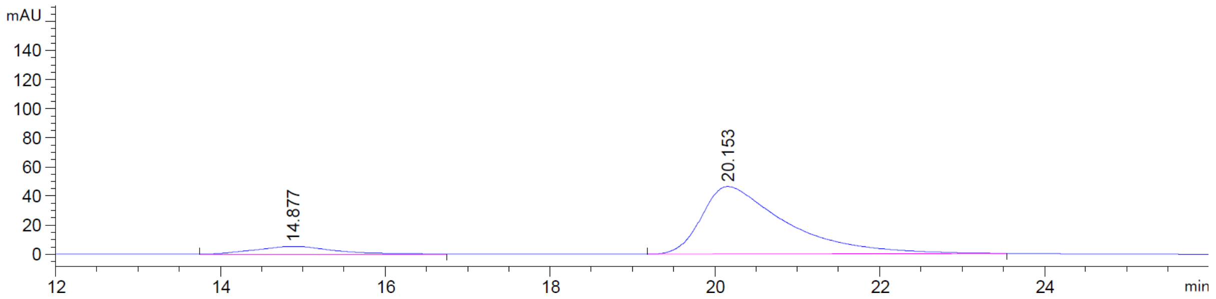

Peak #	RetTime [min]	Type	Width [min]	Area [mAU*s]	Height [mAU]	Area %
1	14.877	BB	0.7652	339.03006	5.27966	9.3369
2	20.153	BB	0.9822	3292.06274	46.37511	90.6631

3-((4a*R*,9a*R*)-1-Chloro-7-(trifluoromethyl)-4a,9a-dihydro-9*H*-fluorene-4-carbonyl) oxazolidin-2-one (2f)

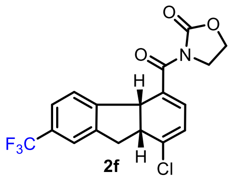

(CHIRALPAK IA column, *n*-hexane/^{i}PrOH, 70:30 v/v, v = 1.0 mL/min, λ = 254 nm)

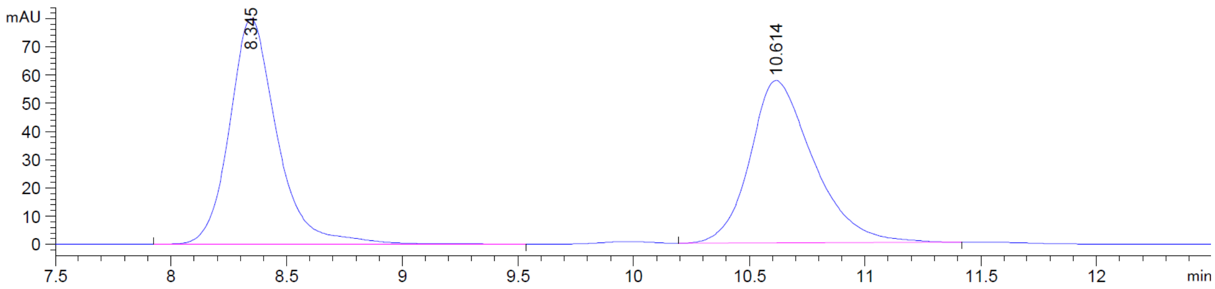

Peak #	RetTime [min]	Type	Width [min]	Area [mAU*s]	Height [mAU]	Area %
1	8.345	BB	0.2128	1143.33276	79.98671	50.9251
2	10.614	BB	0.2851	1101.79407	57.61125	49.0749

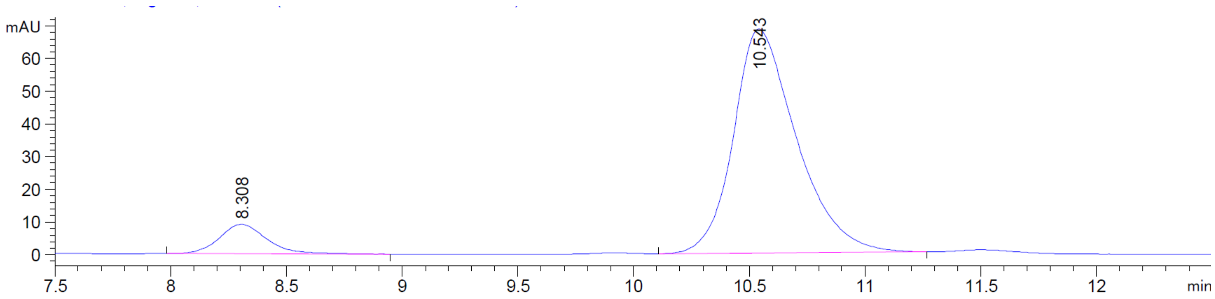

Peak #	RetTime [min]	Type	Width [min]	Area [mAU*s]	Height [mAU]	Area %
1	8.308	BB	0.2165	128.62129	9.01199	9.0066
2	10.543	BB	0.2811	1299.45093	68.55762	90.9934

Methyl (4b*R*,8a*R*)-8-chloro-5-(2-oxooxazolidine-3-carbonyl)-4b,8a-dihydro-9*H*-fluorene-2-carboxylate (2g)

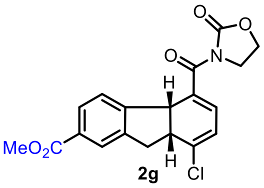

(CHIRALPAK IC column, *n*-hexane/^{i}PrOH, 60:40 v/v, v = 1.0 mL/min, λ = 280 nm)

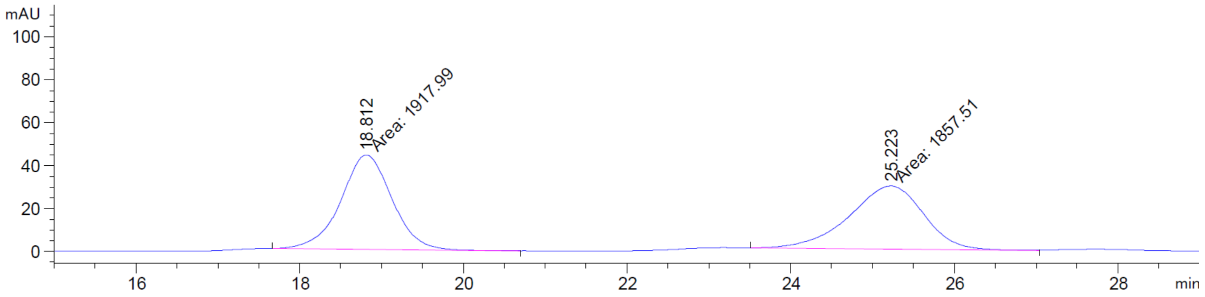

Peak #	RetTime [min]	Type	Width [min]	Area [mAU*s]	Height [mAU]	Area %
1	18.812	MM	0.7277	1917.99182	43.92873	50.8010
2	25.223	MM	1.0554	1857.50891	29.33231	49.1990

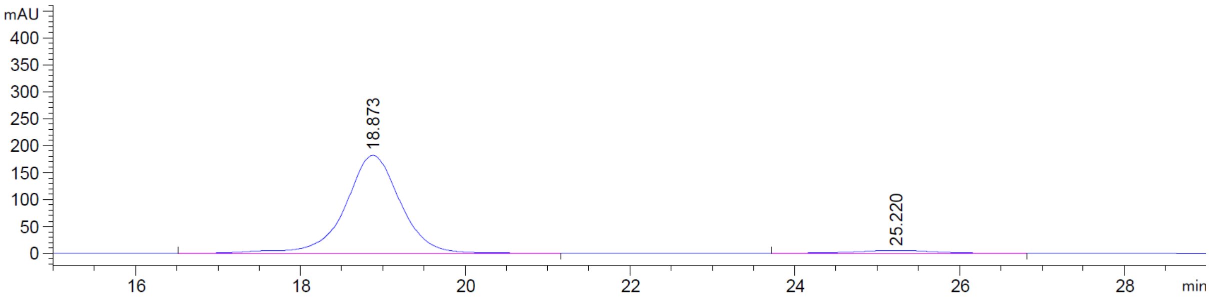

Peak #	RetTime [min]	Type	Width [min]	Area [mAU*s]	Height [mAU]	Area %
1	18.873	BB	0.7001	8472.69434	181.67311	96.1430
2	25.220	BB	0.7622	339.90518	5.35733	3.8570

3-((4a*R*,9a*R*)-1-Chloro-6-nitro-4a,9a-dihydro-9*H*-fluorene-4-carbonyl)oxazolidin-2-one (2h)

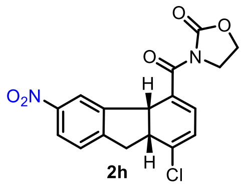

(CHIRALPAK IA column, *n*-hexane/^{i}PrOH, 70:30 v/v, v = 1.0 mL/min, λ = 254 nm)

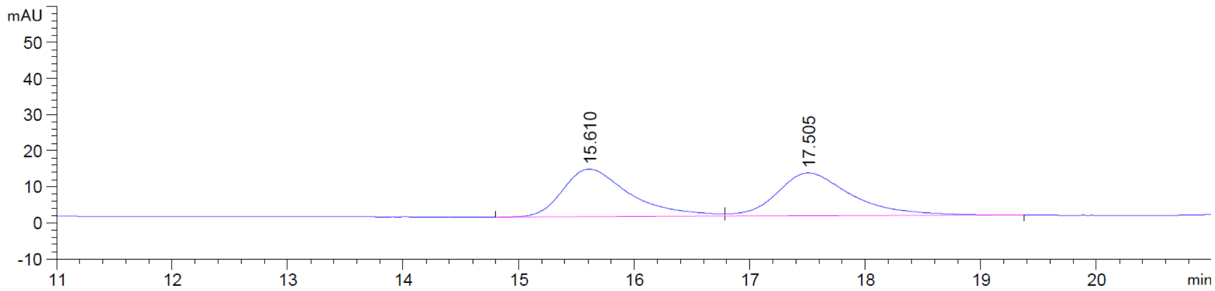

Peak #	RetTime [min]	Type	Width [min]	Area [mAU*s]	Height [mAU]	Area %
1	15.611	BV	0.5975	654.08569	16.11692	49.7512
2	17.504	VB	0.6650	660.62878	14.57003	50.2488

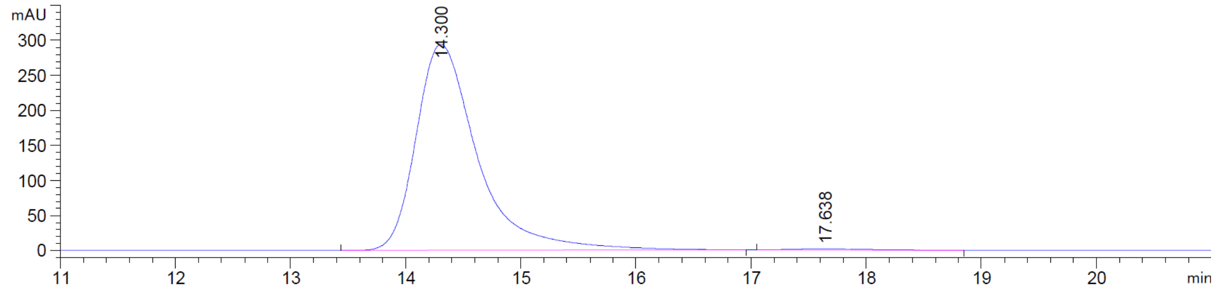

Peak #	RetTime [min]	Type	Width [min]	Area [mAU*s]	Height [mAU]	Area %
1	14.300	BB	0.5642	1.09482e4	294.15765	99.2918
2	17.638	BB	0.5076	78.09200	1.81645	0.7082

3-((4a*R*,9a*R*)-1-Chloro-7-(4-(trifluoromethyl)phenyl)-4a,9a-dihydro-9*H*-fluorene-4-carbonyl)oxazolidin-2-one (2i)

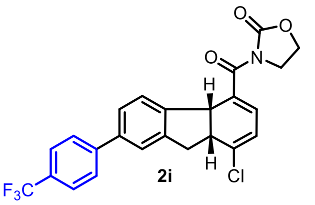

2i

(CHIRALPAK IA column, *n*-hexane/^{i}PrOH, 80:20 v/v, v = 1.0 mL/min, λ = 280 nm)

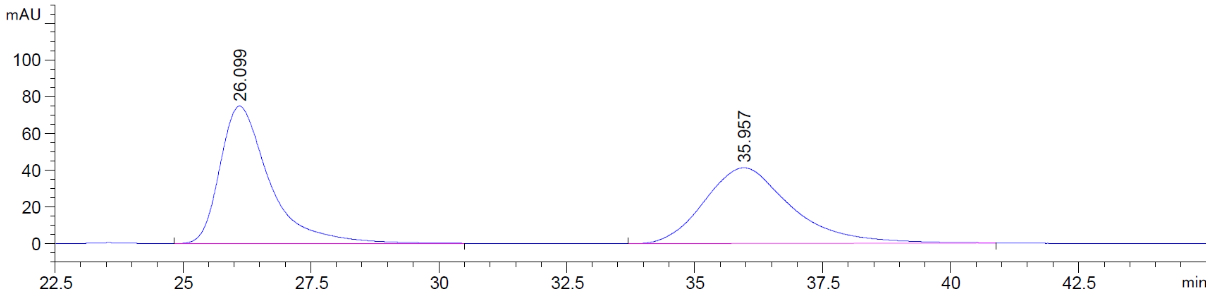

Peak #	RetTime [min]	Type	Width [min]	Area [mAU*s]	Height [mAU]	Area %
1	26.099	BB	1.0132	5052.39453	74.88021	50.9224
2	35.957	BB	1.6068	4869.35547	41.26066	49.0776

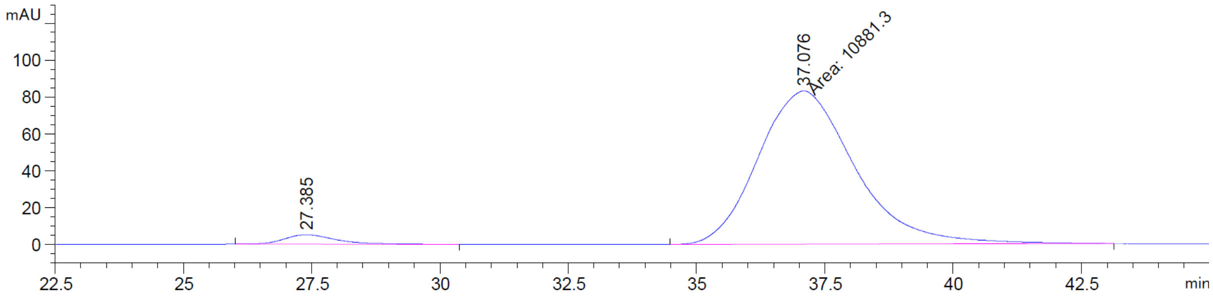

Peak #	RetTime [min]	Type	Width [min]	Area [mAU*s]	Height [mAU]	Area %
1	27.385	BB	0.8437	356.29922	5.09621	3.1026
2	37.076	MM	2.2163	1.11277e4	83.68168	96.8974

Methyl 4-((4b*R*,8a*R*)-8-chloro-5-(2-oxooxazolidine-3-carbonyl)-4b,8a-dihydro-9*H*-fluoren-2-yl)benzoate (2j)

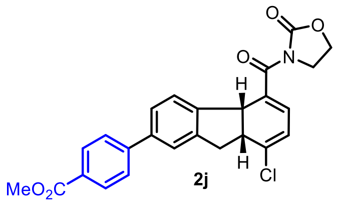

(CHIRALPAK IA column, *n*-hexane/^{i}PrOH, 80:20 v/v, v = 1.0 mL/min, λ = 280 nm)

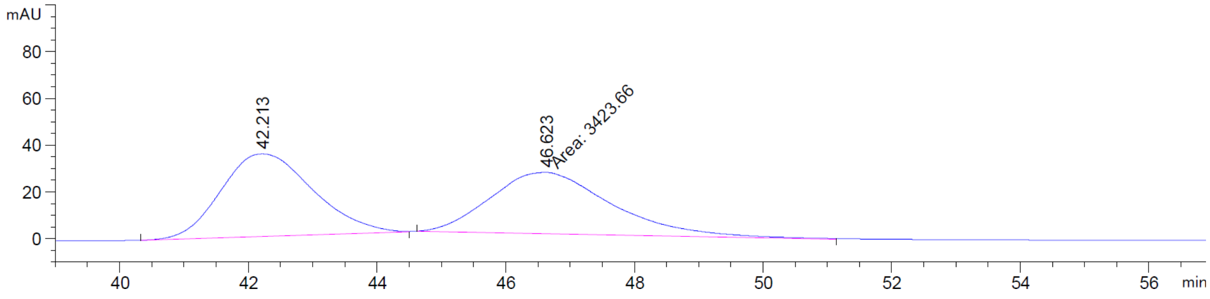

Peak #	RetTime [min]	Type	Width [min]	Area [mAU*s]	Height [mAU]	Area %
1	42.213	BB	1.3705	3488.23145	35.25090	50.7672
2	46.623	MM	2.1569	3382.80786	26.13955	49.2328

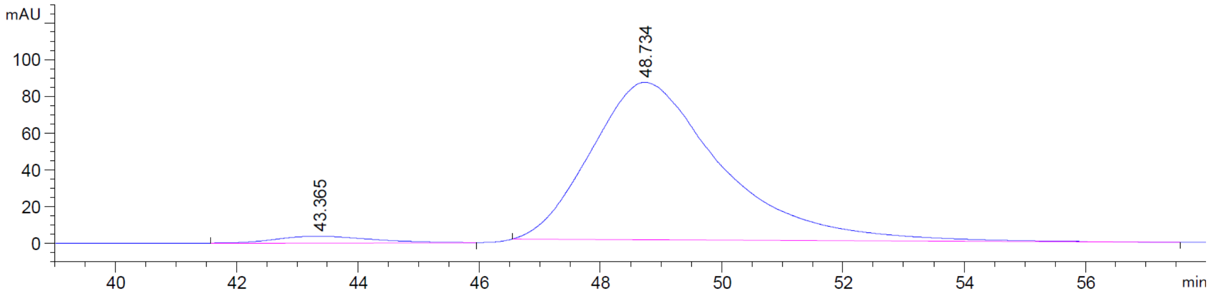

Peak #	RetTime [min]	Type	Width [min]	Area [mAU*s]	Height [mAU]	Area %
1	43.365	BB	1.2521	385.93320	3.63085	2.7883
2	48.734	MM	2.5704	1.34552e4	87.24401	97.2117

3-((4a*R*,9a*R*)-1-Chloro-6-methyl-4a,9a-dihydro-9*H*-fluorene-4-carbonyl)oxazolidin-2-one (2k)

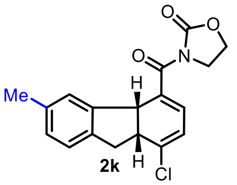

(CHIRALPAK IA column, *n*-hexane/^{i}PrOH, 90:10 v/v, v = 1.0 mL/min, λ = 280 nm)

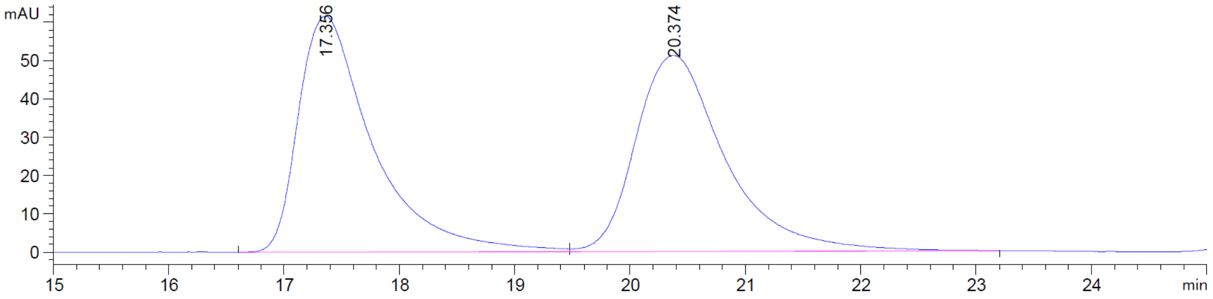

Peak #	RetTime [min]	Type	Width [min]	Area [mAU*s]	Height [mAU]	Area %
1	17.356	BV	0.6634	2763.80762	61.60146	50.0311
2	20.374	VB	0.8113	2760.37305	51.17186	49.9689

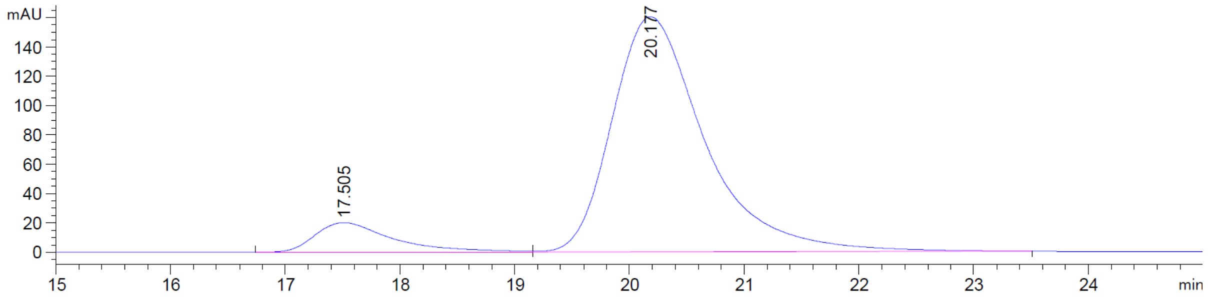

Peak #	RetTime [min]	Type	Width [min]	Area [mAU*s]	Height [mAU]	Area %
1	17.505	BV	0.6849	939.12225	20.03241	9.4637
2	20.177	VB	0.8543	8984.31152	160.20148	90.5363

3-((4a*R*,9a*R*)-1-Chloro-8-methoxy-4a,9a-dihydro-9*H*-fluorene-4-carbonyl)oxazolidin-2-one, 2l:

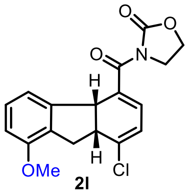

2l

(CHIRALPAK IA column, *n*-hexane/^{i}PrOH, 85:15 v/v, v = 1.0 mL/min, λ = 280 nm)

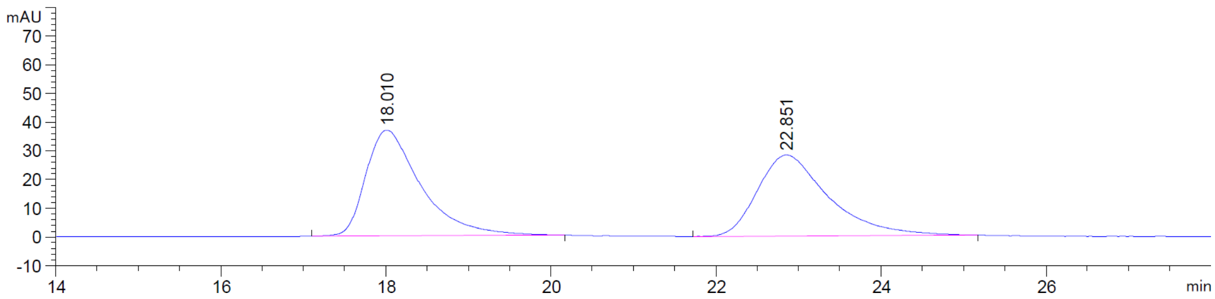

Peak #	RetTime [min]	Type	Width [min]	Area [mAU*s]	Height [mAU]	Area %
1	18.010	BB	0.6970	1714.40967	36.83637	50.2892
2	22.851	BB	0.8457	1694.69385	28.25762	49.7108

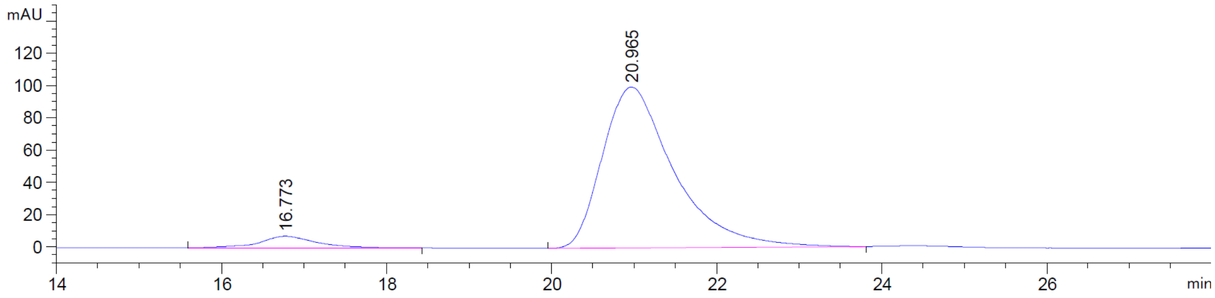

Peak #	RetTime [min]	Type	Width [min]	Area [mAU*s]	Height [mAU]	Area %
1	16.773	BB	0.6476	355.37616	7.27940	5.5857
2	20.965	BB	0.8995	6006.91309	99.61276	94.4143

3-((4a*R*,9a*R*)-1-Chloro-8-(4,4,5,5-tetramethyl-1,3,2-dioxaborolan-2-yl)-4a,9a-dihydro-9*H*-fluorene-4-carbonyl)oxazolidin-2-one (2m)

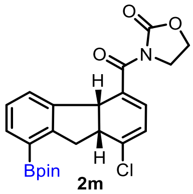

2m

(CHIRALPAK IA column, *n*-hexane/^{i}PrOH, 85:15 v/v, v = 1.0 mL/min, λ = 280 nm)

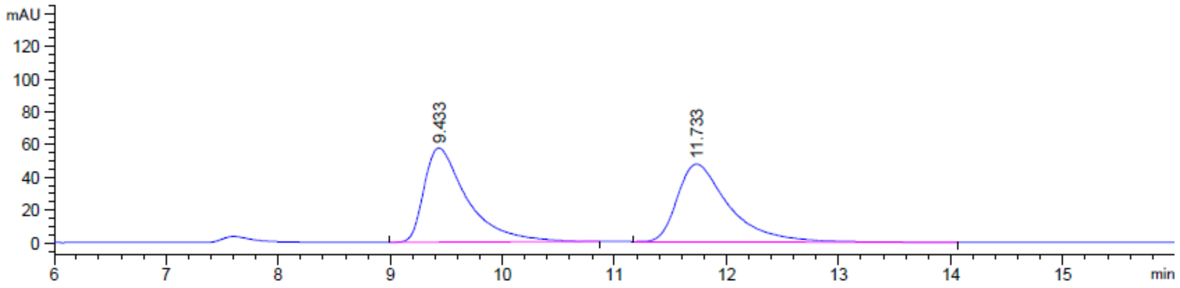

Peak #	RetTime [min]	Type	Width [min]	Area [mAU*s]	Height [mAU]	Area %
1	9.433	BB	0.3967	1543.76978	57.59274	50.3323
2	11.733	BB	0.4722	1523.38843	47.50164	49.6677

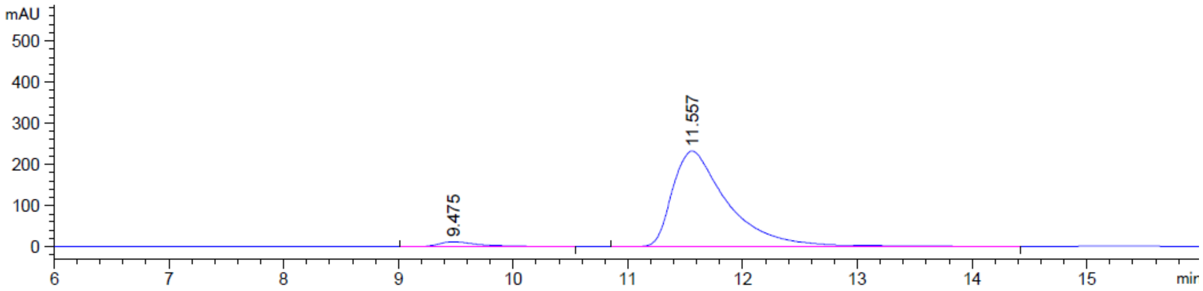

Peak #	RetTime [min]	Type	Width [min]	Area [mAU*s]	Height [mAU]	Area %
1	9.475	BV	0.3762	298.53268	11.67936	3.7074
2	11.557	BB	0.4963	7753.87402	231.77615	96.2926

3-((4a*R*,9a*R*)-1-Chloro-6-phenyl-4a,9a-dihydro-9*H*-fluorene-4-carbonyl)oxazolidin-2-one, 2n:

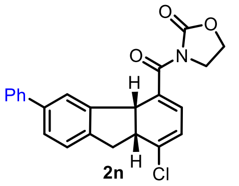

(CHIRALPAK IB column, *n*-hexane/^{i}PrOH, 90:10 v/v, v = 1.0 mL/min, λ = 254 nm)

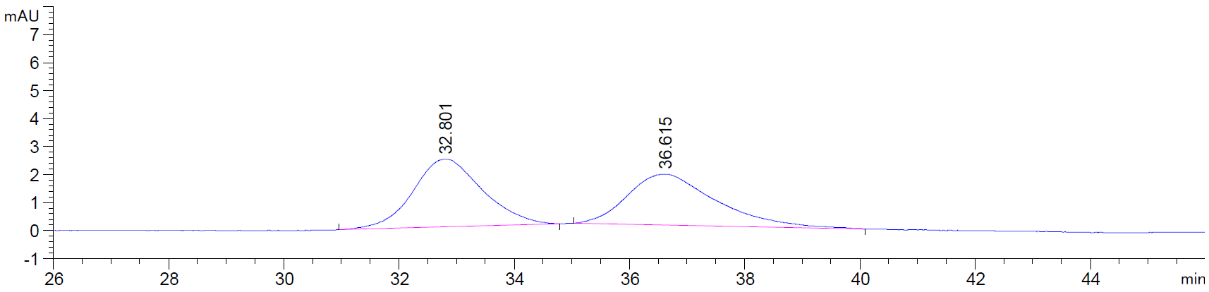

Peak #	RetTime [min]	Type	Width [min]	Area [mAU*s]	Height [mAU]	Area %
1	32.801	BB	0.9891	203.00980	2.41904	51.2260
2	36.615	BB	1.2457	193.29262	1.81910	48.7740

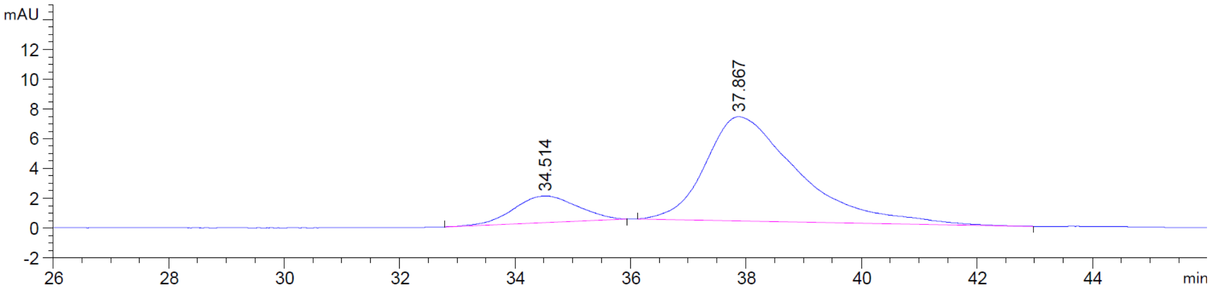

Peak #	RetTime [min]	Type	Width [min]	Area [mAU*s]	Height [mAU]	Area %
1	34.514	BB	0.9054	137.75940	1.79501	14.9288
2	37.867	BB	1.3249	785.01514	6.99686	85.0712

3-((7a*R*,11a*R*)-8-Chloro-7a,11a-dihydro-7*H*-benzo[*c*]fluorene-11-carbonyl)oxazolidin-2-one (2o)

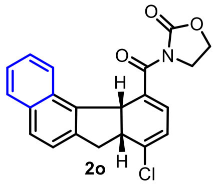

(CHIRALCEL® OD-H column, *n*-hexane/^{i}PrOH, 85:15 v/v, v = 1.0 mL/min, λ = 280 nm)

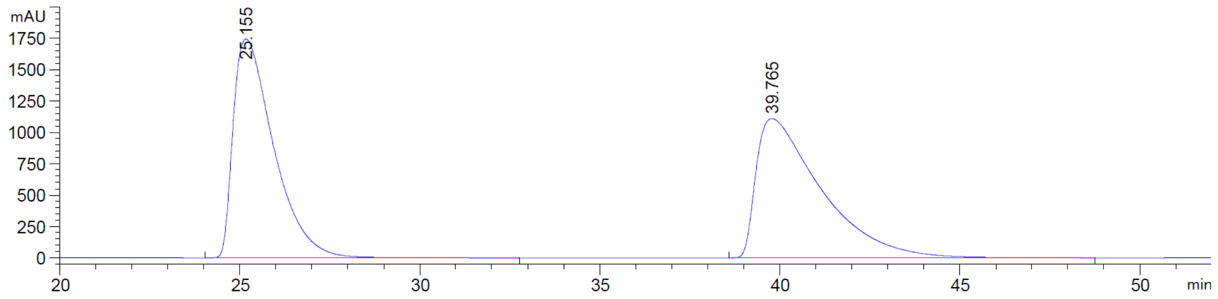

Peak #	RetTime [min]	Type	Width [min]	Area [mAU*s]	Height [mAU]	Area %
1	25.155	BB	1.1719	1.36255e5	1743.18518	49.4393
2	39.765	BB	1.7537	1.39345e5	1109.13379	50.5607

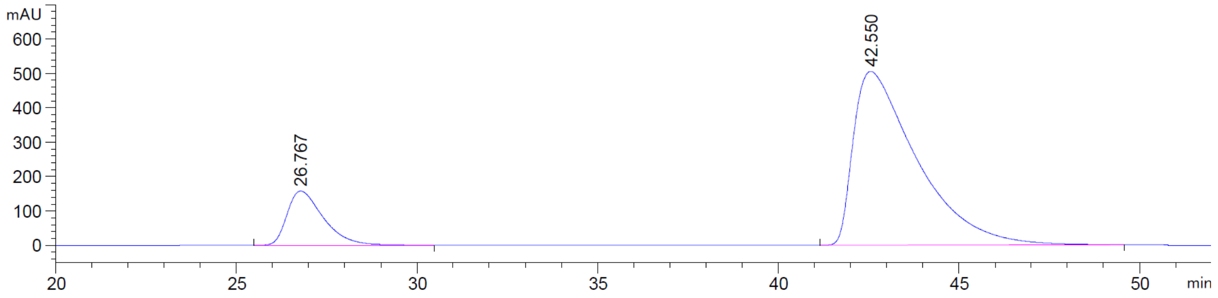

Peak #	RetTime [min]	Type	Width [min]	Area [mAU*s]	Height [mAU]	Area %
1	26.767	BB	1.0499	1.09923e4	157.56133	15.0729
2	42.550	BB	1.7410	6.19350e4	505.63669	84.9271

3-((6b*R*,10a*S*)-10-Chloro-6b,10a-dihydrofluoranthene-7-carbonyl)oxazolidin-2-one (2p)

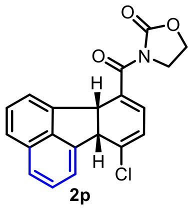

2p

(CHIRALPAK IC column, *n*-hexane/^{i}PrOH, 80:20 v/v, v = 1.0 mL/min, λ = 280 nm)

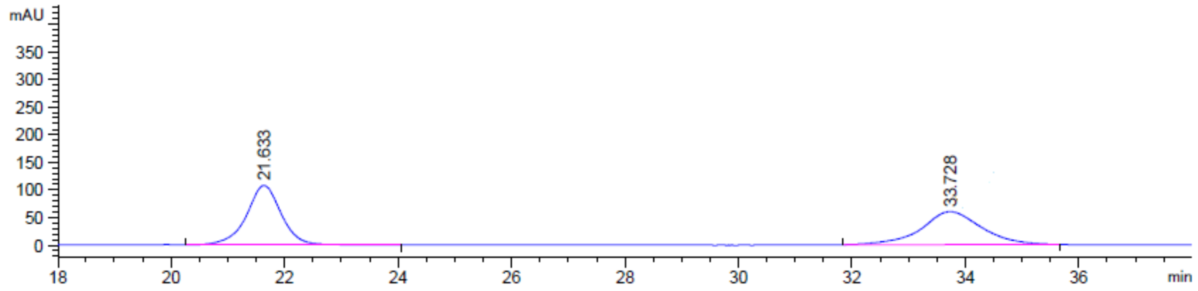

Peak #	RetTime [min]	Type	Width [min]	Area [mAU*s]	Height [mAU]	Area %
1	21.633	BB	0.6399	4635.49463	107.75072	51.2965
2	33.728	MM	1.2318	4401.16797	59.54695	48.7035

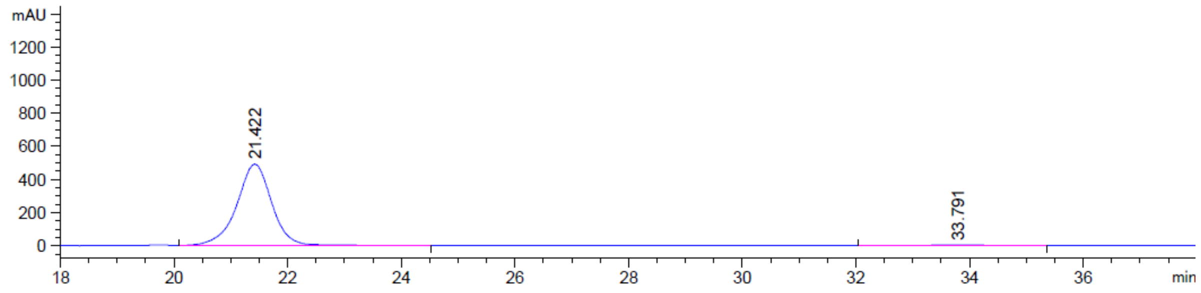

Peak #	RetTime [min]	Type	Width [min]	Area [mAU*s]	Height [mAU]	Area %
1	21.422	VV	0.6476	2.13289e4	492.13434	98.3476
2	33.791	BB	0.8640	358.36298	4.90730	1.6524

3-((4a*R*,9a*R*)-1-Methyl-4a,9a-dihydro-9*H*-fluorene-4-carbonyl)oxazolidin-2-one (2q)

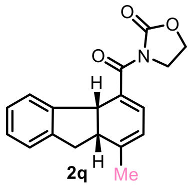

(CHIRALCEL® OD-H column, *n*-hexane/^{i}PrOH, 90:10 v/v, v = 1.0 mL/min, λ = 280 nm)

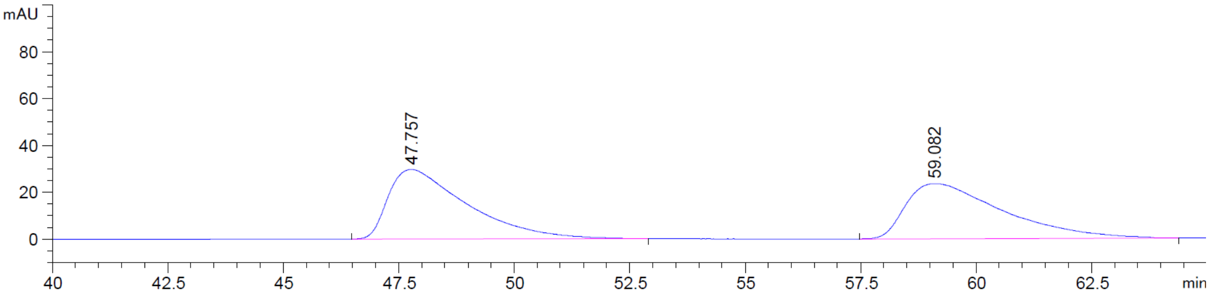

Peak #	RetTime [min]	Type	Width [min]	Area [mAU*s]	Height [mAU]	Area %
1	47.757	BB	1.4735	3521.32690	29.73764	50.5965
2	59.082	BB	1.7208	3438.30005	23.47449	49.4035

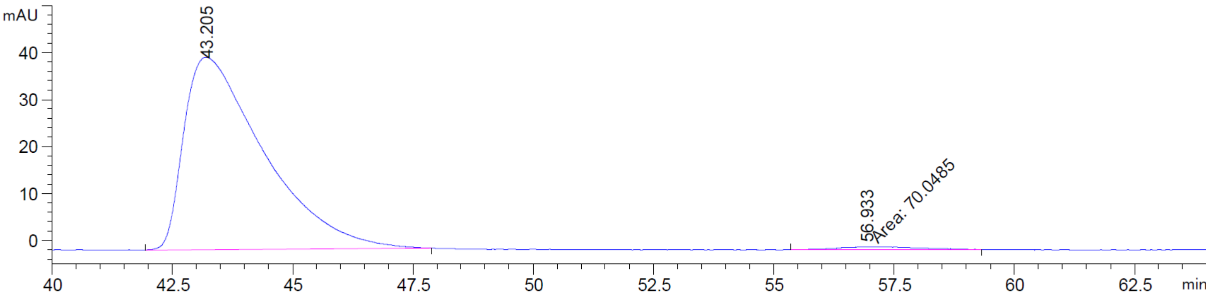

Peak #	RetTime [min]	Type	Width [min]	Area [mAU*s]	Height [mAU]	Area %
1	43.205	MM	1.9478	4794.95068	41.02773	98.8121
2	56.933	MM	1.7772	57.64234	5.40573e-1	1.1879

3-((4a*R*,9a*R*)-1-Bromo-4a,9a-dihydro-9*H*-fluorene-4-carbonyl)oxazolidin-2-one (2r)

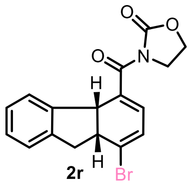

2r

(CHIRALPAK IA column, *n*-hexane/^{i}PrOH, 85:15 v/v, v = 1.0 mL/min, λ = 280 nm)

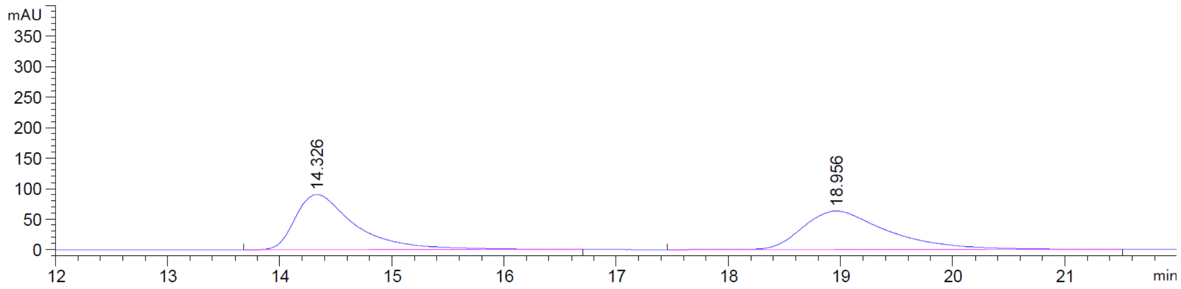

Peak #	RetTime [min]	Type	Width [min]	Area [mAU*s]	Height [mAU]	Area %
1	14.326	BB	0.5503	3364.47803	90.38505	50.1928
2	18.956	BB	0.7759	3338.63403	63.24023	49.8072

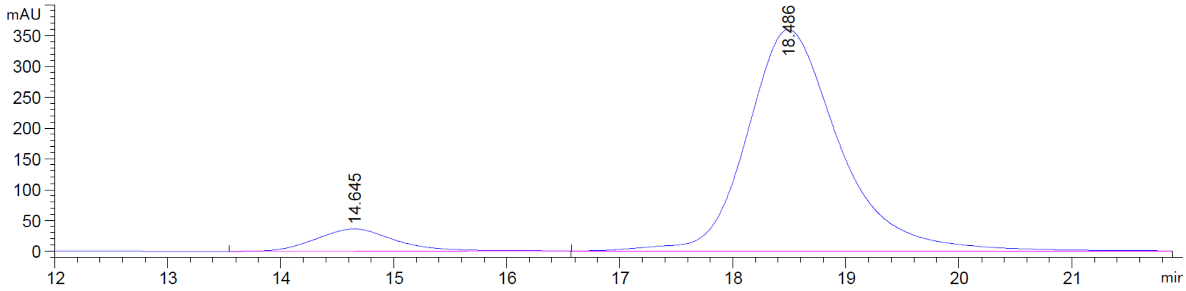

Peak #	RetTime [min]	Type	Width [min]	Area [mAU*s]	Height [mAU]	Area %
1	14.645	BB	0.7325	1731.09363	35.77567	7.8631
2	18.486	BBA	0.8546	2.02844e4	359.30988	92.1369

3-((5a*R*,10a*R*)-1,2,3,5a,10,10a-Hexahydrocyclopenta[*a*]fluorene-5-carbonyl)oxazolidin-2-one (2s)

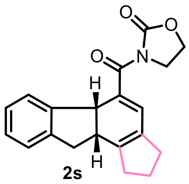

(CHIRALPAK IC column, *n*-hexane/^{i}PrOH, 80:20 v/v, v = 1.0 mL/min, λ = 210 nm)

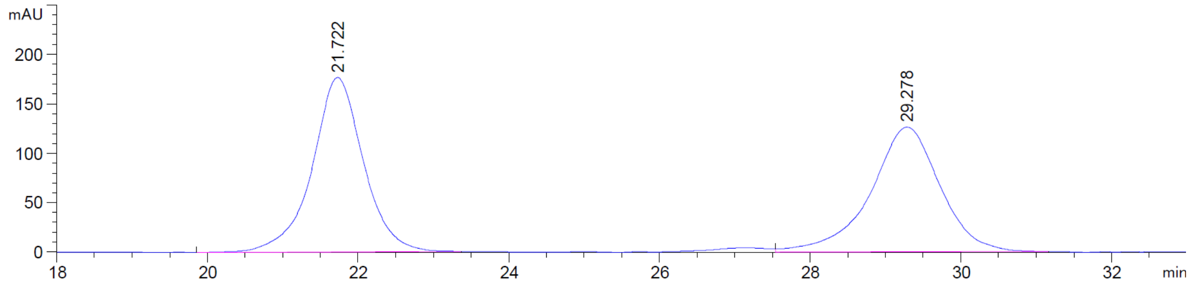

Peak #	RetTime [min]	Type	Width [min]	Area [mAU*s]	Height [mAU]	Area %
1	21.722	BB	0.7041	8391.60254	176.67111	50.9849
2	29.278	VB	0.9379	8067.38477	126.18296	49.0151

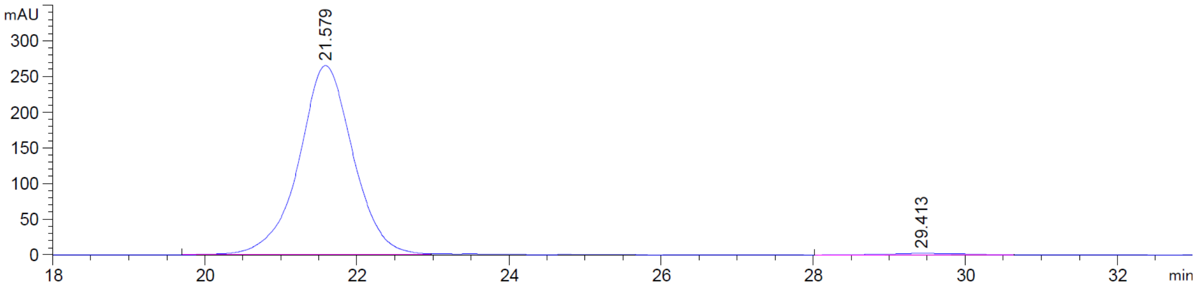

Peak #	RetTime [min]	Type	Width [min]	Area [mAU*s]	Height [mAU]	Area %
1	21.579	BV	0.7440	1.32020e4	265.56317	98.6842
2	29.413	BV	0.8434	176.02686	2.50019	1.3158

3-((4a*R*,9a*R*)-1-Bromo-2-methyl-4a,9a-dihydro-9*H*-fluorene-4-carbonyl)oxazolidin-2-one (2t)

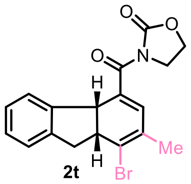

(CHIRALCEL® OD-H, *n*-hexane/^{i}PrOH, 90:10 v/v, v = 1.0 mL/min, λ = 280 nm)

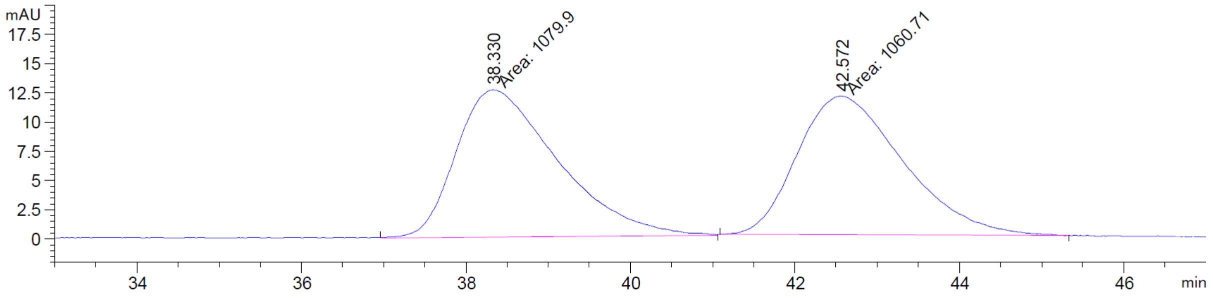

Peak #	RetTime [min]	Type	Width [min]	Area [mAU*s]	Height [mAU]	Area %
1	38.330	MM T	1.4144	1079.90076	12.58352	50.4482
2	42.572	MM T	1.4916	1060.71277	11.85187	49.5518

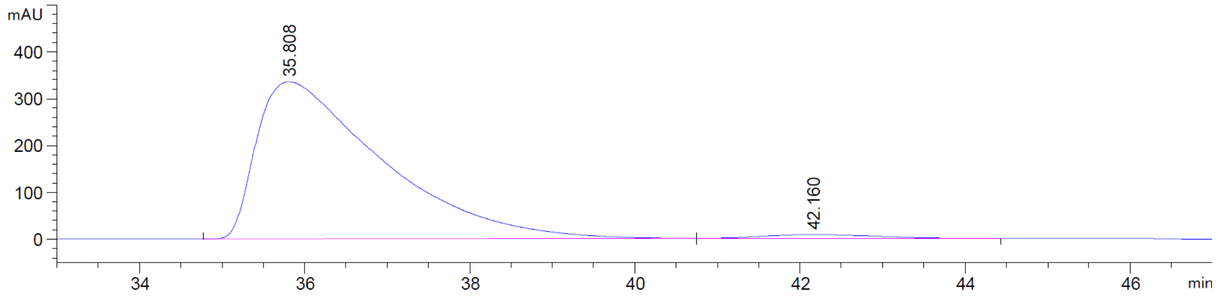

Peak #	RetTime [min]	Type	Width [min]	Area [mAU*s]	Height [mAU]	Area %
1	35.808	BB	1.5409	3.53835e4	335.37955	97.8558
2	42.160	BB	1.1202	775.31555	8.29676	2.1442

((4a*R*,9a*R*)-1-Chloro-4a,9a-dihydro-9*H*-fluoren-4-yl)methanol (3)

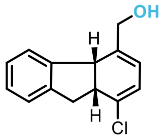

3a

(CHIRALCEL® OD-H column, *n*-hexane/^{i}PrOH, 92:8 v/v, v = 1.0 mL/min, λ = 280 nm)

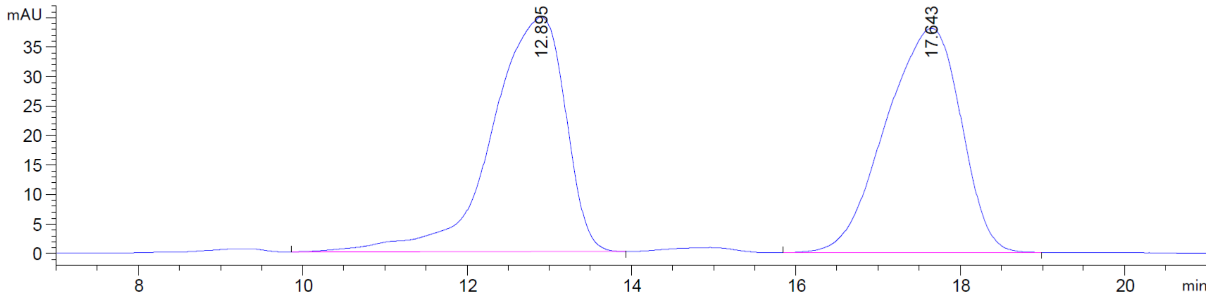

Peak #	RetTime [min]	Type	Width [min]	Area [mAU*s]	Height [mAU]	Area %
1	12.895	BB	0.9886	2476.45313	39.76455	50.6371
2	17.643	BB	0.9327	2414.13501	38.02625	49.3629

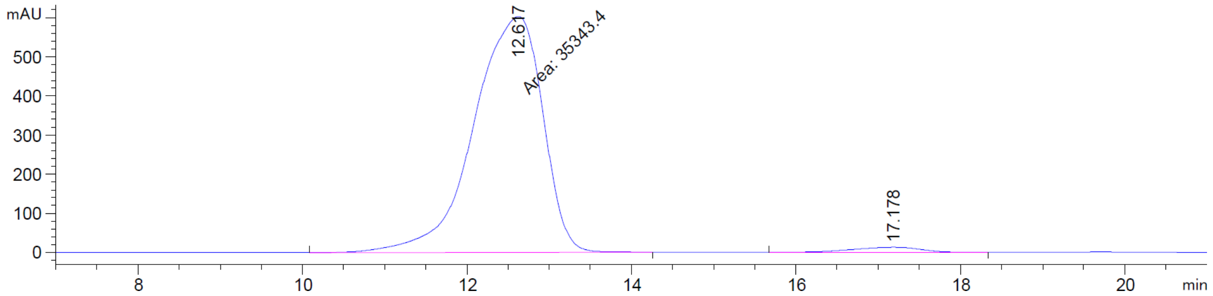

Peak #	RetTime [min]	Type	Width [min]	Area [mAU*s]	Height [mAU]	Area %
1	12.617	MM	0.9758	3.53434e4	603.66486	97.9621
2	17.178	BB	0.8037	735.23962	13.00251	2.0379

Methyl (4a*R*,9a*R*)-1-chloro-4a,9a-dihydro-9*H*-fluorene-4-carboxylate (4)

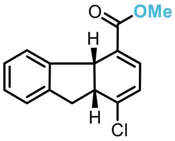

3b

(CHIRALCEL® OD-H column, *n*-hexane/^{i}PrOH, 95:5 v/v, v = 1.0 mL/min, λ = 280 nm)

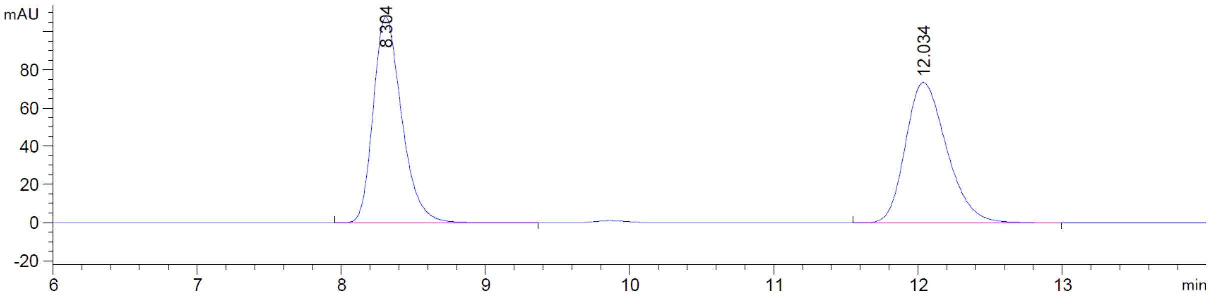

Peak #	RetTime [min]	Type	Width [min]	Area [mAU*s]	Height [mAU]	Area %
1	8.304	BB	0.2097	1475.37439	107.80419	50.3348
2	12.034	BB	0.3073	1455.74805	73.41798	49.6652

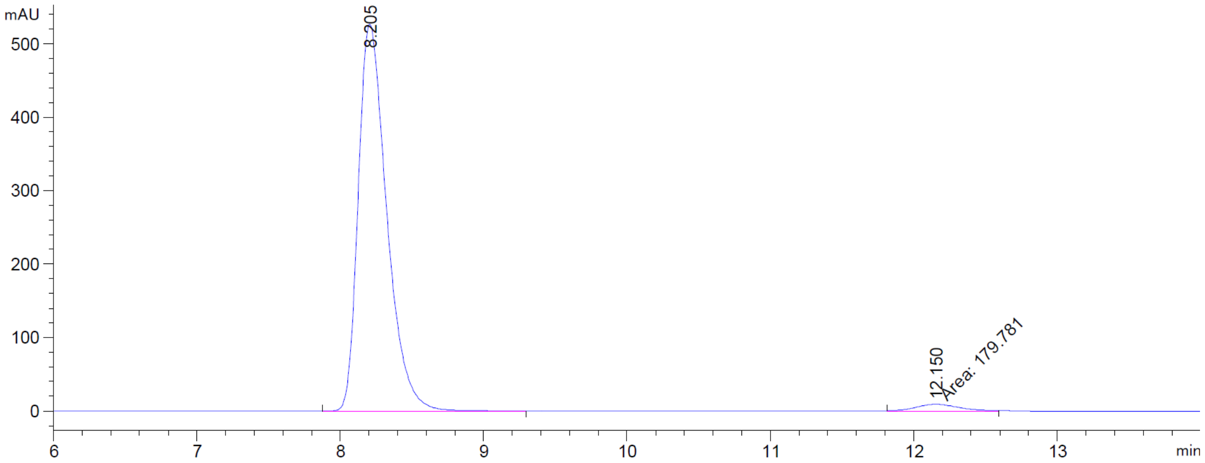

Peak #	RetTime [min]	Type	Width [min]	Area [mAU*s]	Height [mAU]	Area %
1	8.205	BB	0.2108	7258.74170	526.64368	97.5831
2	12.150	MM	0.3342	179.78069	8.96683	2.4169

Methyl (4a*R*,9a*R*)-1-phenyl-4a,9a-dihydro-9*H*-fluorene-4-carboxylate (5)

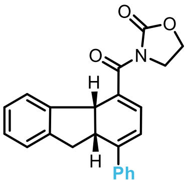

3c

(CHIRALCEL® OD-H column, *n*-hexane/^{i}PrOH, 85:15 v/v, v = 1.0 mL/min, λ = 360 nm)

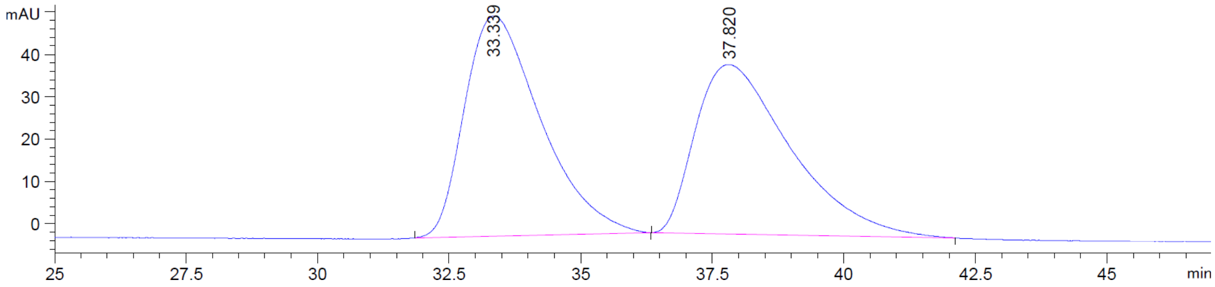

Peak #	RetTime [min]	Type	Width [min]	Area [mAU*s]	Height [mAU]	Area %
1	33.339	BB	1.2449	5249.04443	52.03534	51.0043
2	37.820	BB	1.4833	5042.32715	40.09137	48.9957

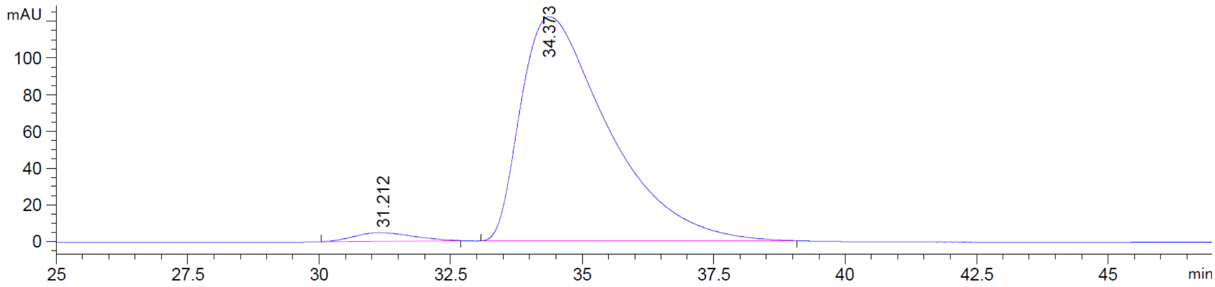

Peak #	RetTime [min]	Type	Width [min]	Area [mAU*s]	Height [mAU]	Area %
1	31.212	BB	0.9276	361.39755	4.61484	2.4915
2	34.373	BB	1.4225	1.41440e4	122.18690	97.5085

Methyl (4a*R*,9a*R*)-1-phenyl-4a,9a-dihydro-9*H*-fluorene-4-carboxylate (6)

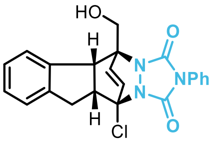

3d

(CHIRALPAK IB column, *n*-hexane/^{i}PrOH, 60:40 v/v, v = 1.0 mL/min, λ = 254 nm)

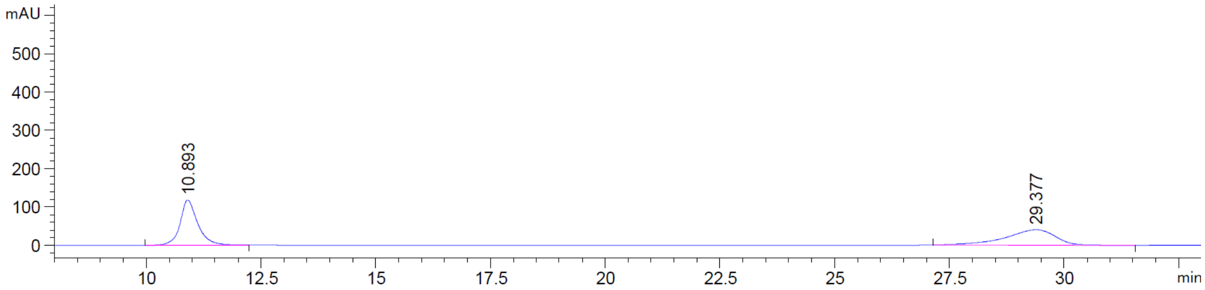

Peak #	RetTime [min]	Type	Width [min]	Area [mAU*s]	Height [mAU]	Area %
1	10.893	BV	0.3956	3233.96216	118.79745	51.5271
2	29.377	BB	1.1055	3042.27539	40.35559	48.4729

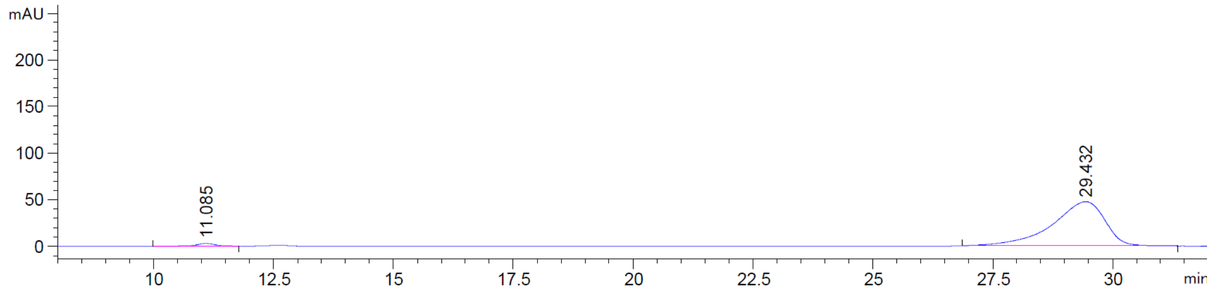

Peak #	RetTime [min]	Type	Width [min]	Area [mAU*s]	Height [mAU]	Area %
1	11.085	BB	0.4032	92.03707	3.14697	2.4778
2	29.432	BB	1.1225	3622.43677	47.45884	97.5222

3-((4a*R*,8a*S*)-4-Chloro-4a,5,6,8a-tetrahydronaphthalene-1-carbonyl)oxazolidin-2-one (7)

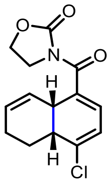

3e

(CHIRALCEL® OD-H column, *n*-hexane/^{i}PrOH, 75:25 v/v, v = 1.0 mL/min, λ = 280 nm)

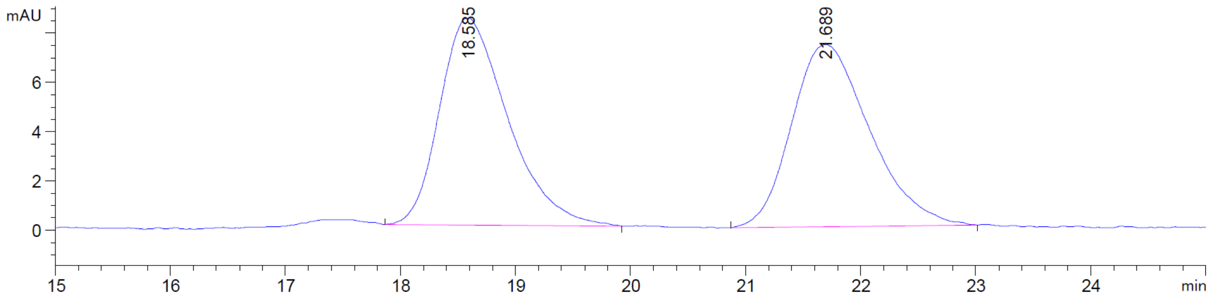

Peak #	RetTime [min]	Type	Width [min]	Area [mAU*s]	Height [mAU]	Area %
1	18.585	BB	0.5989	346.90741	8.41607	49.8547
2	21.689	BB	0.5659	348.92899	7.40253	50.1453

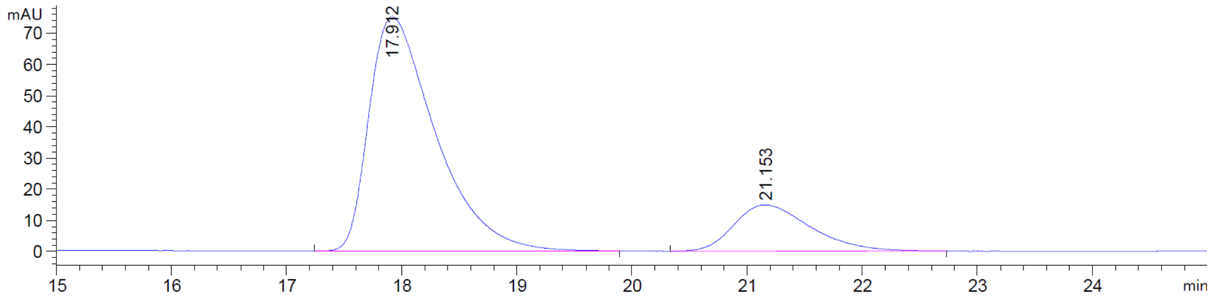

Peak #	RetTime [min]	Type	Width [min]	Area [mAU*s]	Height [mAU]	Area %
1	17.912	BB	0.6041	3011.93262	75.06451	81.5059
2	21.153	BB	0.6713	683.42145	14.78412	18.4941

3-((4a*R*,9a*R*)-4-Chloro-4a,9-dimethyl-4a,9a-dihydro-9*H*-carbazole-1-carbonyl)oxazolidin-2-one (9)

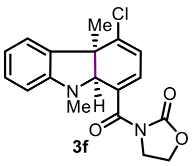

3f

(CHIRALPAK IB column, *n*-hexane/^{i}PrOH, 80:20 v/v, v = 1.0 mL/min, λ = 254 nm)

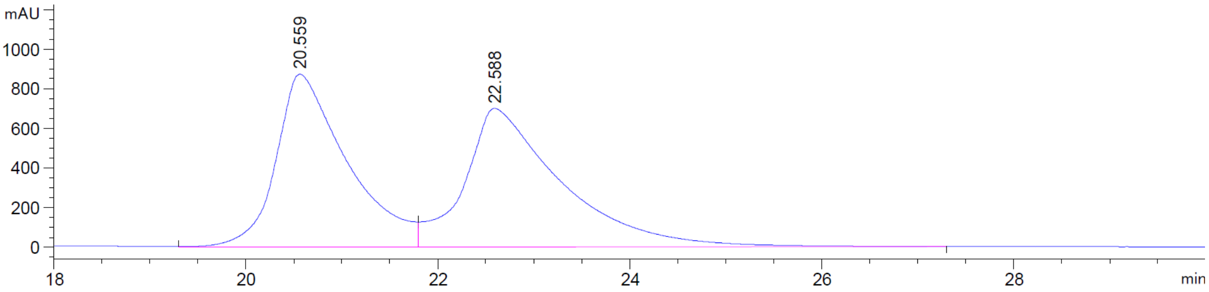

Peak #	RetTime [min]	Type	Width [min]	Area [mAU*s]	Height [mAU]	Area %
1	20.559	VV	0.7185	4.49286e4	872.85388	47.8195
2	22.588	VV	0.9560	4.90259e4	699.25568	52.1805

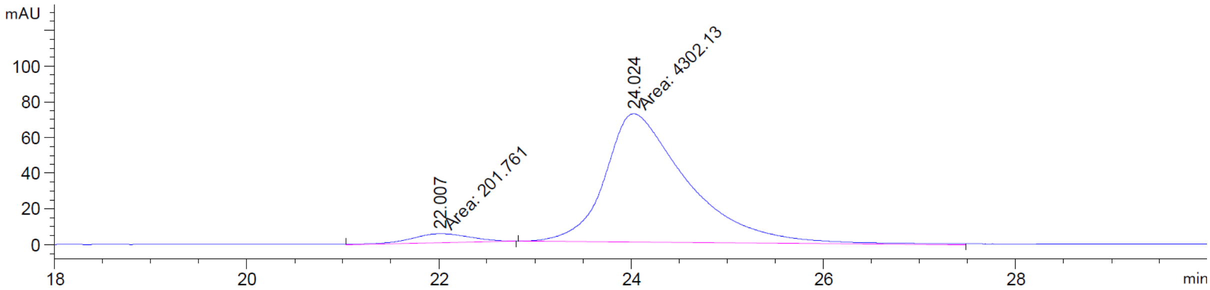

Peak #	RetTime [min]	Type	Width [min]	Area [mAU*s]	Height [mAU]	Area %
1	22.007	MM	0.6802	201.76062	4.94371	4.4797
2	24.024	MM	0.9973	4302.12500	71.89961	95.5203

10. References

(1) Lu, Y.; Xu, M. M.; Zhang, Z. M.; Zhang, J.; Cai, Q. Catalytic Asymmetric Inverse-Electron-Demand Diels-Alder Reactions of 2-Pyrones with Indenes: Total Syntheses of Cephanolides A and B. *Angew. Chem. Int. Ed.* **2021**, *60*, 26610–26615.
(2) Wu, H.; Wang, Q.; Zhu, J. P. Copper-Catalyzed Enantioselective Domino Arylation/Semipinacol Rearrangement of Allylic Alcohols with Diaryliodonium Salts. *Chem. Eur. J.* **2017**, *23*, 13037–13041.
(3) Xue, C. B.; Chen, L. H.; Cao, G. F.; Zhang, K.; Wang, A. L.; Meloni, D.; Glenn, J.; Anand, R.; Xia, M.; Kong, L.; et al. Discovery of INCB9471, a Potent, Selective, and Orally Bioavailable CCR5 Antagonist with Potent Anti-HIV-1 Activity. *ACS Med. Chem. Lett.* **2010**, *1*, 483–487.
(4) Niwa, T.; Uetake, Y.; Isoda, M.; Takimoto, T.; Nakaoka, M.; Hashizume, D.; Sakurai, H.; Hosoya, T. Lewis acid-mediated Suzuki-Miyaura cross-coupling reaction. *Nat. Catal.* **2021**, *4*, 1080–1088.
(5) Hou, M.; Xu, M. M.; Yang, B. C.; He, H. B.; Gao, S. H. Construction of polycyclic structures with vicinal all-carbon quaternary stereocenters an enantioselective photoenolization/Diels-Alder reaction. *Chem. Sci.* **2021**, *12*, 7575–7582.
(6) Narasaka, K.; Iwasawa, N.; Inoue, M.; Yamada, T.; Nakashima, M.; Sugimori, J. Asymmetric Diels-Alder Reaction Catalyzed by a Chiral Titanium Reagent. *J. Am. Chem. Soc.* **1989**, *111*, 5340–5345.
(7) Varga, B.; Vincze, D.; Peto, H.; Buna, L.; Pauló, J.; Holczbauer, T.; Mátravölgyi, B.; Hegedus, L.; Fogassy, E.; Keglevich, G.; et al. Resolution of aryl-H-phosphinates applied in the synthesis of P-stereogenic compounds including a Bronsted acid NMR solvating agent. *Org. Chem. Front.* **2022**, *9*, 2797–2807.
(8) Du, H. F.; Zhao, D. B.; Ding, K. L. Enantioselective catalysis of the hetero-Diels-Alder reaction between Brassard's diene and aldehydes by hydrogen-bonding activation: A one-step synthesis of (+)-Dihydrokawain. *Chem. Eur. J.* **2004**, *10*, 5964–5970.
(9) Cuenca, A.; Medio-Simón, M.; Aguilar, G. A.; Weibel, D.; Beck, A. K.; Seebach, D. Highly enantioselective protonation of the 3,4-dihydro-2-methylnaphthalen-1(2)-one Li-enolate by TADDOLs. *Helv. Chim. Acta* **2000**, *83*, 3153–3162.
(10) Cosier, J.; Glazer, A. M. A Nitrogen-Gas-Stream Cryostat for General X-Ray Diffraction Studies. *J. Appl. Crystallogr.* **1986**, *19*, 105–107.
(11) Sheldrick, G. M. SHELXT – Integrated Space-Group and Crystal-Structure Determination. *Acta Crystallogr. Sect. Found. Adv.* **2015**, *71*, 3–8.
(12) Palatinus, L.; Chapuis, G. SUPERFLIP – a Computer Program for the Solution of Crystal Structures by Charge Flipping in Arbitrary Dimensions. *J. Appl. Crystallogr.* **2007**, *40*, 786–790.
(13) Betteridge, P. W.; Carruthers, J. R.; Cooper, R. I.; Prout, K.; Watkin, D. J. CRYSTALS Version 12: Software for Guided Crystal Structure Analysis. *J. Appl. Crystallogr.* **2003**, *36*, 1487–1487.
(14) Parois, P.; Cooper, R. I.; Thompson, A. L. Crystal Structures of Increasingly Large Molecules: Meeting the Challenges with CRYSTALS Software. *Chem. Cent. J.* **2015**, *9*, 30.
